# ﻿New species of the genus *Chimarra* Stephens from Africa (Trichoptera, Philopotamidae) and characterization of the African groups and subgroups of the genus

**DOI:** 10.3897/zookeys.1111.77586

**Published:** 2022-07-11

**Authors:** Roger Blahnik, Trond Andersen

**Affiliations:** 1 Department of Entomology, University of Minnesota, 1980 Folwell Ave., 219 Hodson Hall, St. Paul, Minnesota, 55108, USA University of Minnesota St. Paul United States of America; 2 Department of Natural History, University Museum of Bergen, University of Bergen, PO Box 7800, NO-5020 Bergen, Norway University of Bergen Bergen Norway

**Keywords:** Democratic Republic of the Congo, Ghana, new synonyms, South Africa, species groups, species subgroups, Tanzania

## Abstract

This paper is primarily based on collections in Tanzania and Ghana in 1990–1991 and 1991–1994, respectively. In all, 46 species of *Chimarra* were collected, 31 of them new species. All these species are illustrated or re-illustrated and described in the paper. Additionally, five species from Africa from collections in Illinois and Minnesota, four of them new, are included. This provided the incentive to review the species of *Chimarra* from the African subregion and assign the majority of them to species groups and subgroups. In the process, several species were synonymized. In all 147 valid species are recognized, of which 51 are treated in this paper. Two major species groups are recognized for Africa, the *marginata* Group and the *georgensis* Group. The former is based on the type species for the genus; this is the first formal characterization of this group, as distinct from other species groups in the subgenus. Mainland African species in the *marginata* Group mostly fall into four large species-diverse subgroups, but a number of smaller subgroups are also recognized. Membership in these subgroups is specified for the majority of African species and characters defining the subgroups informally discussed. The *georgensis* Group includes a single Asian species and nine previously described African species. They are assigned to two subgroups, one newly recognized in this paper. Several additional species were considered unassigned to subgroup within the *georgensis* Group. The majority of the new species described in this paper belong to the *georgensis* Group.

## ﻿Introduction

*Chimarra* is currently the largest genus in the order Trichoptera, with approximately 950 species, or just under 6% of the diversity for the entire order. It is distributed on all major continents, except Antarctica, and divided into four subgenera, three of them endemic to the New World. New World subgenera were comprehensively treated in several relatively recent revisions (Blahnik 1997, 2002; Flint 1998). The fourth and nominate subgenus is found throughout the Old World, including Africa, Asia, and the Australian Region, including many of the Pacific islands, and also has a substantial radiation in the New World in both North and South America, except for the Chilean subregion. The type species, *Chimarramarginata* (Linnaeus), is the only species of the genus widely distributed in Europe, and also occurs in northern Africa. New World members of the subgenus were treated in a revision by Blahnik (1998). Old World species have not been comprehensively revised, but several valuable resources are available for identification of species in various regional faunas [southeast Asia: Malicky (2010); Australia and New Guinea: Cartwright (2002, 2020); the Pacific islands: Johanson and Espeland (2010), Johanson et al. (2011), Johanson and Oláh (2012)]. Also of inestimable value is a searchable online catalogue of described species for the entire order Trichoptera (Morse 2021). Confirmation for the general monophyly of the subgenera, including the nominate subgenus, was provided by two independent molecular analyses (Kjer et al. 2014; Wahlberg and Johanson 2014).

Currently, 115 species of *Chimarra* are described from Africa and Madagascar. Of these, three are brought into synonymization in the current work and an additional 35 new species are described. Undoubtedly, many additional species remain to be described. Most of the known species were described one or a few a time, in a number of papers, some obscure and difficult to obtain. The general quality of the descriptions and illustrations for many of the species is not high; many species lack comparative diagnoses and identification may be difficult or uncertain. The most useful compilation of the literature, not only for *Chimarra*, but for the entire African fauna of Trichoptera, was provided by Tobias and Tobias (2008), as an online resource that is no longer updated, but is archived at (http://trichoptera.senckenberg.de/Trichoptera%20africana/introduction.htm). This mostly contains species illustrations without the accompanying descriptions, but literature references are included, as well as comments on possible synonyms. Several recent papers treating the African fauna have begun revisionary work on selected groups within the genus *Chimarra*, or enumeration of related taxa (Gibon 2015, 2017, 2018), but the majority of African and Madagascan taxa have not been so treated. A more comprehensive, but still preliminary, contribution to this endeavor can be found in Table 1. However, some species are only known from females or have illustrations or descriptions too incomplete to be useful in making assessments; they are included at the end of Table 1.

**Table 1.** African *Chimarra* species.

**The *marginata* Group**
**The *apiconigra* subgroup**
*Chimarraapiconigra* Johanson, 2010 [Madagascar]
*Chimarragassa* Johanson, 2010 [Madagascar]
**The *cara* subgroup**
*Chimarrabispinosa Gibbs, 1973 [Ghana, Ivory Coast]
*Chimarracara* Mosely, 1936 (*Chimarrha*) [Cameroon]
**The *fallax* subgroup**
?*Chimarrabettinae* Marlier & Marlier, 1982 (proposed by Gibon 2018) [Réunion]
*Chimarracalundoensis Marlier, 1965 [Angola, DR of the Congo, Ghana]
*Chimarradybowskina Navás, 1931 (*Chimarrha*) [Burkino Faso, Cape Verde, DR of the Congo,
Ghana, Guinea, Ivory Coast, Madagascar, Mali, Togo]
Syn.: *C.caboverdensis* Nybom, 1960, syn. nov. [Cape Verde]
Syn.: *C.divergena* Gibbs, 1973, syn. nov. [Ghana]
*Chimarraelga Mosely, 1939 (*Chimarrha*) [DR of the Congo, Kenya]
*Chimarrafalcifera* Jacquemart, 1966 [DR of the Congo]
*Chimarrafallax Ulmer, 1912 (*Chimarrha*) [Cameroon, DR of the Congo, Ghana, Madagascar, Sao Tomé]
Syn.: *C.lukawei* Jacquemart, 1961 (*Chimarrha*), syn. nov. [DR of the Congo]
*Chimarrajacquemarti sp. nov. [Ghana]
*Chimarralanceolata sp. nov. [Ghana]
*Chimarralejea* Mosely, 1948 (*Chimarrha*) [Ethiopia, Yemen]
*Chimarramauritania* Jacquemart, 1960 (*Chimarrha*) [Mauritius]
*Chimarrarobynsi (Jacquemart, 1967), comb. nov. (*Chimarrhafra*) [DR of the Congo, Tanzania]
*Chimarratogoana (Ulmer, 1907) (*Wormaldia*) [Ghana, Togo]
*Chimarratravei* Jacquemart, 1963 [Mauritius]
**The *kenyana* subgroup**
*Chimarraakana Gibbs, 1973 [Ghana, Ivory Coast]
*Chimarraambulans* Barnard, 1934 (*Chimarrha*) [South Africa]
*Chimarrabaculifera* Marlier, 1965 [Angola]
*Chimarracamerunensis* Marlier, 1980 [Cameroon]
*Chimarrachicapa* Marlier, 1965 [Angola]
*Chimarraeshowensis sp. nov. [South Africa]
*Chimarraflaviseta* Wahlberg, Espeland & Johanson, 2014 [Malawi]
*Chimarraintermedia* Jacquemart, 1961 [DR of the Congo]
*Chimarrakenyana* Ulmer, 1931 (*Chimarrha*) [DR of the Congo, Kenya, South Africa, Zaire]
Syn.: *C.wittei* Jacquemart, 1961 (*Chimarrha*) (proposed by Marlier 1980) [Zaire]
*Chimarrakrugeri Jacquemart, 1963 [South Africa, Tanzania]
*Chimarralongistylis* Jacquemart & Statzner, 1981 [DR of the Congo]
*Chimarramorogoroensis sp. nov. [Tanzania]
*Chimarramulanjae* Wahlberg, Espeland & Johanson, 2014 [Malawi]
*Chimarramushuvae* Marlier, 1951 (*Chimarrha*) [DR of the Congo]
*Chimarrapedaliotus sp. nov. [Ghana]
*Chimarrapsittacus* Wahlberg, Espeland & Johanson, 2014 [Malawi]
*Chimarraquadrispinosa* Jacquemart & Statzner, 1981 [DR of the Congo]
*Chimarrarhodesi* Kimmins, 1957 [DR of the Congo, Zimbabwe]
*Chimarrasaudia* Malicky, 1986 [Yemen]
*Chimarrasomereni* Marlier, 1951 (*Chimarrha*) [Kenya]
*Chimarraszunyoghyi Oláh, 1986 [Tanzania]
*Chimarratanzaniensis sp. nov. [Tanzania]
*Chimarratriangularis* Kimmins, 1963 [Ethiopia]
*Chimarratriangularisoccidentalis Gibon, 1985 [Ghana, Ivory Coast]
*Chimarratrispina* Jacquemart, 1961 (*Chimarrha*) [DR of the Congo]
*Chimarrauvirana* Marlier, 1951 (*Chimarrha*) [DR of the Congo, Zambia]
*Chimarrawaensis Gibon, 1985 [Ghana, Ivory Coast]
*Chimarrazombaensis* Wahlberg, Espeland & Johanson, 2014 [Malawi]
**The *lehibemavo* subgroup**
*Chimarracebegepi* Gibon, 2017 [Madagascar]
*Chimarrafenoevo* Gibon, 2017 [Madagascar]
*Chimarrafotobohitra* Gibon, 2017 [Madagascar]
*Chimarraforcellinii* Gibon, 2017 [Madagascar]
*Chimarragattolliati* Gibon, 2017 [Madagascar]
*Chimarragensonae* Gibon, 2017 [Madagascar]
*Chimarrahamatra* Gibon, 2017 [Madagascar]
*Chimarrajejyorum* Gibon, 2017 [Madagascar]
*Chimarralehibemavo* Gibon, 2017 [Madagascar]
*Chimarramakiorum* Gibon, 2017 [Madagascar]
*Chimarramoramanga* Gibon, 2017 [Madagascar]
*Chimarrasaha* Gibon, 2017 [Madagascar]
*Chimarratamara* Gibon, 2017 [Madagascar]
**The *leta* subgroup**
*Chimarraamakyei sp. nov. [Ghana]
*Chimarraleta* Mosely, 1936 (*Chimarrha*) [Cameroon]
**The *marginata* subgroup**
*Chimarramarginata* (Linnaeus, 1767) (*Phryganea*) [Algeria, Morocco, Tunisia]
**The *mazumbai* subgroup**
*Chimarramazumbai sp. nov. [Tanzania]
*Chimarrausambara sp. nov. [Tanzania]
*Chimarrawliensis sp. nov. [Ghana]
**The *minima* subgroup**
*Chimarraambaja* Mosely, 1939 (*Chimarrha*) [Cameroon, DR of the Congo]
*Chimarraangolensis* Marlier, 1965 [Angola]
*Chimarraantsymeloka* Gibon, 2015 [Madagascar]
*Chimarraassambae* Gibon, 2015 [Cameroon]
*Chimarrabertrandi* Scott, 1974 [Zimbabwe]
*Chimarracallasae Gibon, 1982 [Ghana, Guinea, Mali, Sierra-Leone]
*Chimarracereris* Barnard, 1934 (*Chimarrha*) [Zimbabwe]
*Chimarracognata* Kimmins, 1957 [Angola, Namibia, Zimbabwe]
*Chimarraintexta Mosely, 1931 (*Chimarrha*) [Ghana, Guinea, Ivory Coast, Sierra-Leone]
*Chimarrakoualeensis* Johanson & Mary, 2009 [Mayotte Island]
*Chimarraloffae* Gibon, 2015 [Cameroon, Guinea]
*Chimarralufirae* Jacquemart, 1961 (*Chimarrha*) [DR of the Congo, South Africa, Zimbabwe]
*Chimarraminima Ulmer, 1907 (*Chimarrha*) [Benin, Burkino Faso, Cameroon, Ghana, Guinea,
Ivory Coast, Mali, Togo]
Syn.: *C.petri* Gibbs, 1973 (proposed by Gibon 2015) [Ghana]
Syn.: *C.voltae* Marlier, 1978 (proposed by Gibon 1985) [Burkino Faso]
*Chimarraprodhoni* Gibon, 1985 [Burkino Faso, Guinea, Ivory Coast]
*Chimarrasanagae* Gibon, 2015 [Cameroon]
*Chimarrasassandrae Gibon, 1982 [Cameroon, Ghana, Guinea, Ivory Coast Mali, Togo]
*Chimarratoubaensis* Gibon, 1985 [Guinea, Ivory Coast]
*Chimarravulgaris* Gibon, 2015 [Madagascar]
**The *pondoensis* subgroup**
*Chimarracrocifera* Morse, 1974 [South Africa]
*Chimarrapondoensis* Barnard, 1941 (*Chimarrha*) [South Africa]
**The *ruficeps* subgroup**
*Chimarrachechewa* Wahlberg, Espeland & Johanson, 2014 [Malawi]
*Chimarracircumverta* Wahlberg, Espeland & Johanson, 2014 [Malawi]
*Chimarraclara* Mosely, 1939 (*Chimarrha*) [Uganda]
*Chimarracornuta* Jacquemart & Statzner, 1981 (hom. of *C.cornuta* Ross, 1959) [DR of the Congo]
*Chimarradulensis sp. nov. [Tanzania]
*Chimarrafuscipes* Kimmins, 1958 [Mozambique, South Africa]
*Chimarrakibiensis sp. nov. [Ghana]
*Chimarralwirona* Statzner, 1976 [DR of the Congo]
*Chimarraminacis sp. nov. [Ghana]
*Chimarraruficeps* Ulmer, 1914 (*Chimarrha*) [South Africa]
*Chimarratangaensis sp. nov. [Tanzania]
*Chimarrauncata* Morse, 1974 [South Africa]
**The *georgensis* Group**
**The *georgensis* subgroup**
*Chimarraankylis sp. nov. [Tanzania]
*Chimarraaurita sp. nov. [Ghana]
*Chimarracrescentis sp. nov. [Tanzania]
*Chimarrafurcata* Jacquemart, 1961 [DR of the Congo]
*Chimarrageorgensis* Barnard, 1934 (*Chimarrha*) [South Africa]
*Chimarrahoogstraali* Ross, 1956 [Sudan]
*Chimarraindicis sp. nov. [Ghana]
*Chimarrakabashana* (Marlier, 1943) (*Chimarrhafra*) [DR of the Congo]
*Chimarralatidentis sp. nov. [Tanzania]
*Chimarraleptodactylus sp. nov. [Tanzania]
*Chimarraobuncata sp. nov. [Ghana]
*Chimarrapolycentropoides sp. nov. [DR of the Congo]
*Chimarraralphi sp. nov. [Ghana]
*Chimarraserrella sp. nov. [Ghana]
*Chimarratriramosa sp. nov. [Ghana]
*Chimarrauncinata sp. nov. [Ghana]
*Chimarravermitergata sp. nov. [Tanzania]
*Chimarrazombitsei* Gibon, 2018 [Madagascar]
**The *evoluta* subgroup**
*Chimarraaciculata* Morse, 1974 [South Africa]
*Chimarraevoluta* Kimmins, 1957 [Zimbabwe]
*Chimarrafoliata* Kimmins, 1959 [Uganda]
*Chimarragiboni sp. nov. [Ghana]
*Chimarralobulata sp. nov. [Ghana]
*Chimarramgwashi sp. nov. [Tanzania]
*Chimarraparafoliata sp. nov. [Ghana]
*Chimarrapectinella sp. nov. [Ghana]
**Not assigned to subgroup**
*Chimarraagumatsa sp. nov. [Ghana]
*Chimarraino* Marlier, 1981 [Zambia]
*Chimarrakjaerandseni sp. nov. [Ghana]
**Unassigned
*Chimarraabyssinica* Banks, 1913 (*Chimarrha*) [Ethiopia]
*Chimarraafricana* Enderlein, 1929 (*Chimarrha*) [♀, Tanzania]
*Chimarraarmata* Jacquemart, 1961 (*Chimarrha*) (hom. of *C.armata* Navás) [DR of the Congo]
*Chimarraauripilis* Navás, 1933 (*Chimarrha*) [Niger]
*Chimarraberghei* Marlier, 1951 (*Chimarrha*) [DR of the Congo]
*Chimarrabeylaensis* Gibon, 1986 [Guinea]
*Chimarrablahniki* Johanson, 2010 [Madagascar]
*Chimarracalidopectoris* Wahlberg, Espeland & Johanson, 2014 [Malawi]
*Chimarradeksamensis* Malicky, 1999 [Yemen]
*Chimarradioni* Gibon, 1986 [Guinea]
*Chimarragoedefroitae* Gibon, 2016 [Madagascar]
*Chimarraisbal* Malicky, 2015b [Madagascar, Nosy Be]
*Chimarralacroixi* Navás, 1921 (*Chimarrha*) [♀, Madagascar]
*Chimarralomor* Malicky, 2015b [Madagascar, Nosy Be]
*Chimarralupialae* Jacquemart, 1961 (*Chimarrha*) (the *fallax* subgroup?) [DR of the Congo]
*Chimarramayottensis* Johanson & Mary, 2009 [Madagascar, Mayotte Island]
*Chimarramultisensillata sp. nov. [Tanzania]
*Chimarraphilipponi* Gibon, 1986 [Guinea]
*Chimarrasaganeitina* Navás, 1932 (*Chimarrha*) [Ethiopia]
*Chimarrasylvestris* Gibon, 1985 (the *georgensis* subgroup?) [Ivory Coast]
*Chimarratamsi* Mosely, 1936 (*Chimarrha*) [Sao Tome]
*Chimarrazoria* Mosely, 1939 (*Chimarrha*) [Uganda]

*Described or redescribed in this article. **Species unassigned to subgroup are either based on females, inadequately illustrated, morphologically isolated from other species, known only from Madagascar or other islands, or some combination of above.

## ﻿Background

**Figure 1.**
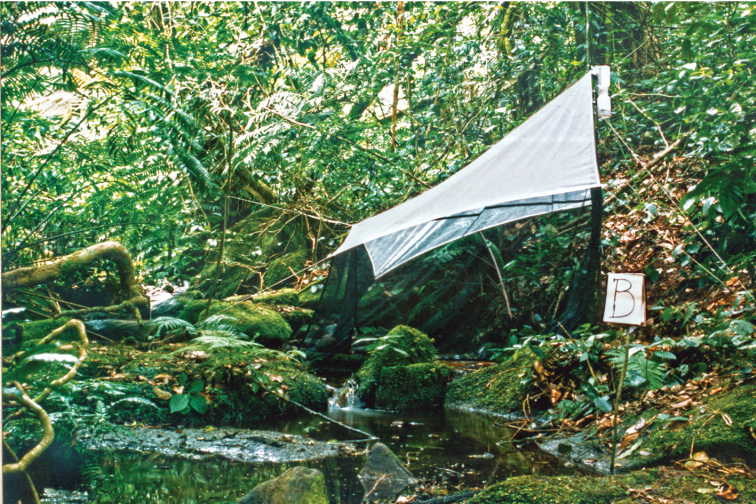
Malaise trap across Kaputu Stream at 1535 m altitude in the Mazumbai Forest Reserve in the West Usambara Mountains, northeastern Tanzania (Photo: Trond Andersen).

Most of the material on which this paper is based was collected during two projects by the Department of Natural History, University Museum of Bergen, Norway (former Museum of Zoology, University of Bergen). The field work during the first project targeted the fauna in the mountain rainforests in the West Usambara Mountains in northeastern Tanzania. The study area was located near the Mazumbai Forest Reserve, where caddisflies were collected in 1990 and 1991 along the Kaputu Stream (Andersen and Johanson 1993) (Fig. 1). The stream originates at 1860 m above sea level and runs down to a marshy area at ~ 1400 m altitude. Four relatively large waterfalls are located along the stream, but in most stretches the water current is moderate. The stream is surrounded by nearly undisturbed rain forest with trees that can reach a height of 50 m.

The West Usambara Mountains belong to the Eastern Arc, a chain of mountains that stretch from the Taita Hills in Kenya in the north, south to the Udzungwa and Mahenge Mountains in Tanzania. They were formed at least one hundred million years ago along a fault lying to the east of the East African Rift, which is a more recent structure. Approximately thirty million years ago, all this area was covered by extensive rainforest. During a period, some ten million years ago, when the climate was cooler and drier, the lowland forests were converted to savannah, leaving the mountain ranges as “islands” where the tropical forests continued to flourish, fed by moisture from the Indian Ocean. This isolation of each mountain range has led to a great deal of endemism, and a very diverse flora and fauna (Burgess et al. 2007; Mumbi et al. 2017).

Andersen and Johanson (1993) estimated that more than 50 species of Trichoptera were collected along the Kaputu Stream. Most unexpected was the first species of the family Beraeidae, *Notoernodesinornatus* Andersen & Kjærandsen, taken in the Afrotropical Region (Andersen and Kjærandsen 1997). The Hydroptilidae have been treated in several articles (Wells and Andersen 1995, 1996; Kjærandsen 2004) and the genus *Tangatrichia* Wells & Andersen was erected based on material from the project. Further some Lepidostomatidae (Weaver and Andersen 1995), Ecnomidae (Andersen and Kjærandsen 2005), and Helicopsychidae (Johanson 1993) have been treated.

During the second project, Trichoptera were collected in most provinces of Ghana from 1991 to 1998 (see Kjærandsen and Andersen 1997). In Ghana, three major vegetation zones are recognized, a belt of tropical rainforest along the southern coast, a transition zone in central Ghana, while northern Ghana is covered with savannah. The forest in southern Ghana belong to the Guinean forests of West Africa, a belt of tropical moist broadleaf forests stretching along the coast of West Africa from Sierra Leone and Guinea in the west to the Sanaga River in Cameroon in the east. The Dahomey Gap, a region of savannah and dry forest in Togo and Benin, divides the Guinean forests into the Upper Guinean forests and Lower Guinean forests. Hall and Swaine (1976) compiled a detailed vegetation map of the rainforest zone in Ghana, showing four main types of forest according to decreasing levels of precipitation from the coast moving inland.

**Figure 2.**
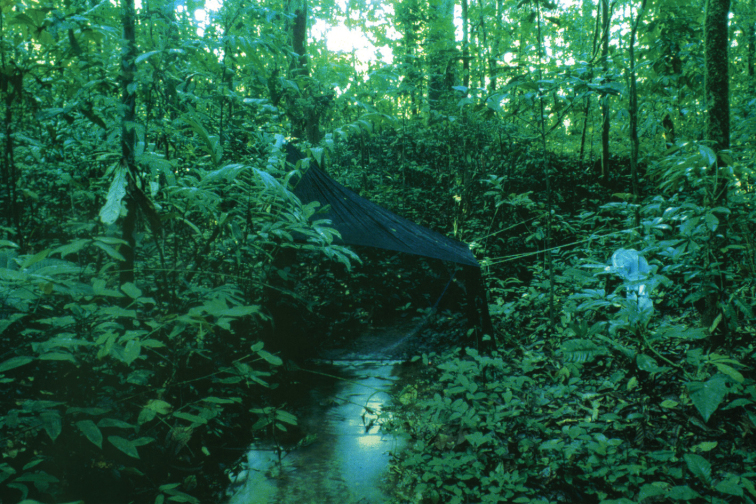
Malaise trap across a small tributary to Ankasa River in the wet evergreen forest in Ankasa Forest Reserve, southwestern Ghana (Photo: Jostein Kjærandsen).

Caddisflies were collected in several localities in the southern, forested part of Ghana. The Ankasa Conservation Area is situated in the Western Region in southwestern Ghana near the border to the Ivory Coast. The conservation area is ~ 500 square kilometers and incorporates the Nini Suhien National Park in the north and the Ankasa Forest Reserve in the south. The altitude varies from 35 m to 170 m and there are three larger rivers and many smaller streams in the area (Fig. 2). The forest is classified as wet evergreen forest (Hall and Swaine 1976) and is an ancient rainforest with the highest biodiversity in Ghana (UICN/PACO 2010).

The Kakum National Park is situated in the Central Region in southern Ghana. It was established as a reserve in 1931 and received the status as a national park in 1992. The Park covers 375 square kilometers and is generally flat with only a few undulating hills ranging 150–250 m above sea level. The forest is classified as moist semi-deciduous forest (Hall and Swaine 1976).

**Figure 3.**
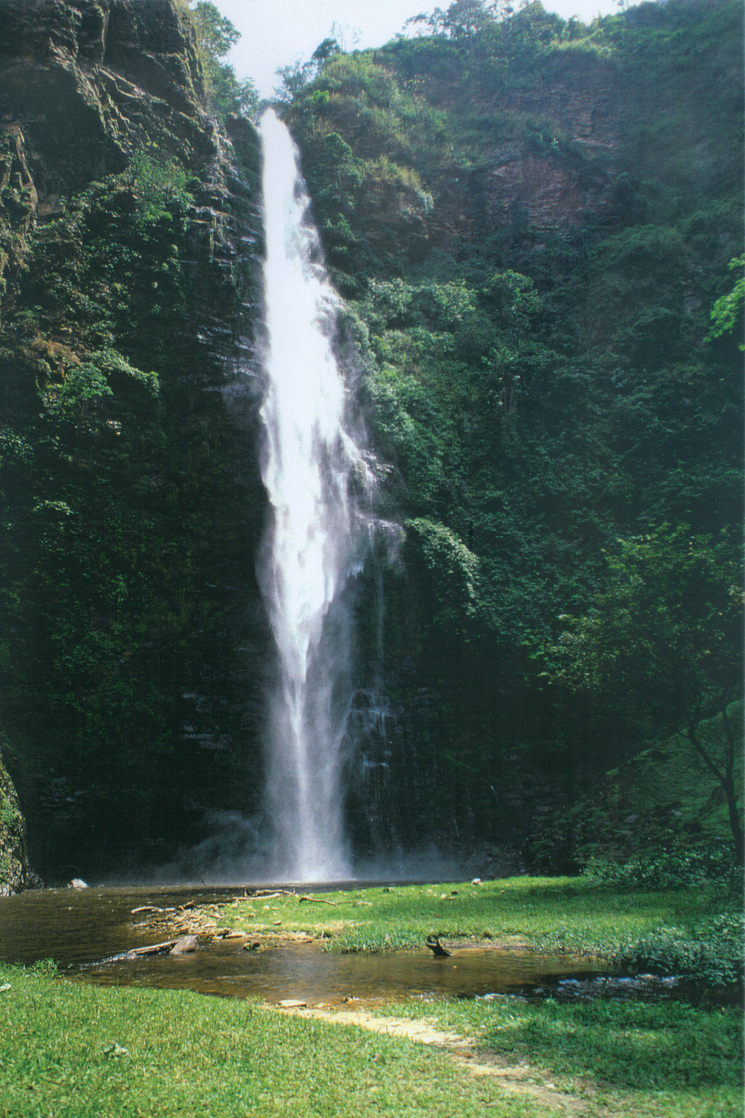
The lower waterfall of Agumatsa-Nubui headwater stream in the Agumatsa Wildlife Sanctuary, eastern Ghana (Photo: Jostein Kjæranden).

Most of the material treated here is from a study of the caddisfly community along a headwater stream in the Agumatsa Wildlife Sanctuary in the Volta Region, situated in the transition zone in the eastern part of Ghana (Andersen and Kjærandsen 2001; Kjærandsen 2005). The sanctuary lies in the southwestern part of the Togo Mountains where it embraces a ravine-riverine forest valley with rather steep sides. The Agumatsa-Nubui headwater stream runs through the valley originating at ~ 750 m altitude on the top of a mountain ridge and falls 250 m, mainly in two large waterfalls, into the bottom of the valley at ~ 350 m altitude (Fig. 3).

Northern Ghana is covered with savannah and is crossed by several large rivers flowing southwards. Caddisflies were mainly collected at two of these rivers, the Black Volta and Oti rivers. The Black Volta originates in Burkina Faso and in Ghana it forms the border with Ivory Coast before it joins the White Volta. The Oti River has its headwaters in Benin and Burkina Faso and flows through Benin and Togo before it joins the Volta River in Ghana.

Kjærandsen and Andersen (1997) listed eight *Chimarra* species from Ghana, today 34 species are known from the country (Table 1). Other taxa have also been described based on the material collected in Ghana during the project, among them the tribe Blyzophilini (Leptoceridae) based on *Blyzophilusdorsohamatus* Andersen & Kjærandsen collected in the Ankasa Conservation Area (Andersen et al. 1999). Of other Leptoceridae genera *Triaenodes* (Andersen and Holzenthal 2001, 2002a), *Adicella* (Andersen and Holzenthal 2002b), and *Tagalopsyche* (Holzenthal and Andersen 2007) have been treated. Of Hydroptilidae two new genera, *Wlitrichia* Kjærandsen and *Cyclopsiella* Kjærandsen were erected based on material from the Ankasa Conservation Area (Kjærandsen 1997), and new species of *Dahtrichia* (Kjærandsen 2004) and *Jabitrichia* (Kjærandsen and Andersen 2002) have been added. Further, new species of Polycentropodidae (Kjærandsen and Netland 1997), Ecnomidae (Andersen and Kjærandsen 2005), and Lepidostomatidae (Weaver and Andersen 1995) have also been described.

## ﻿Materials and methods

Because of the frequent use of species group names to refer to taxa in the subgenus Chimarra, in different regions where it occurs, we have adopted a more formal convention. Hopefully, it will not be found confusing. This includes the use of the species epithet of the first species described in a group or subgroup, followed by use of “Group” or “subgroup.” The difference in capitalization is used to diminish confusion between the two divisions, and because the same species name may be used to characterize both a species group and subgroup. Only formal “Group” names established in this paper are capitalized. In this paper, four major species groups are recognized within the subgenus Chimarra: the *marginata* Group, the *georgensis* Group, the *tsudai* Group, and the *minuta* Group. The last two groups are restricted to Asia. The *georgensis* Group is predominantly found in Africa; a single Asian species is currently assigned to the group (Blahnik et al. 2012), and it is possible that others may occur. The *marginata* Group is found worldwide, throughout the distribution of the subgenus. It encompasses most of the species group names previously proposed for the subgenus, from various parts of the world. These names would be considered subgroups within the *marginata* Group, as the group name is used here. A few taxa, in various regions, may be difficult to place within this structure. They should be considered as unassigned to species group, until their relationships are better established. Subgroup names, for the most part, are regionally restricted.

The fieldwork during the project in Tanzania targeted the fauna in the mountain rainforests in the West Usambara Mountains in northeastern Tanzania. The study area is located near the Mazumbai Forest Reserve, where caddisflies were collected along the Kaputu Stream (Andersen and Johanson 1993). Malaise traps were situated for shorter or longer periods at 11 sites along the stream between late October 1990 and early February 1991. The material was preserved directly in a container with ethylene-glycol and later transferred to 80% ethanol. In addition, caddisflies were collected with sweep nets, both in the Mazumbai Forest Reserve, as well as along other streams and rivers in the West Usambara Mountains. One species was also taken on the campus of the Teachers college in Morogoro.

During the second project, Trichoptera were collected in most provinces of Ghana from 1991 to 1998 (see Kjærandsen and Andersen 1997); only the material collected between 1991 and 1994 is included in the present study. The Ankasa Conservation Area was visited repeatedly in 1993 and we collected at several sites in the southern part of the reserve. The Kakum National Park was visited in the autumn of 1994, and we collected mainly near the main entrance. In the Agumatsa Wildlife Sanctuary caddis flies were collected at 12 sites along a 5 km stretch of the Agumatsa-Nubui headwater stream in spring and autumn 1993 (Kjærandsen 2005). In northern Ghana we collected at the Black Volta and Oti rivers in 1991 to 1993.

During the project in Ghana, caddisflies were mainly collected in light traps, Malaise traps, and with sweep nets and preserved in 80% ethanol. The females were tentatively associated with the males based on their co-occurrence with males and relative similarity. Because of this uncertainty, and because multiple species were collected at many sites, females of most new species are not listed in the paratype series, but instead included as additional material.

Illustrations were made using an ocular grid and inked in Adobe Illustrator. All figures are drawn of the left side or appendage, unless otherwise noted. Setation is generally omitted from the right side. Terminology used follows Blahnik (1998).

Type material is deposited in the collections of the University of Minnesota, St. Paul, Minnesota (**UMSP**), Department of Natural History, University Museum of Bergen (**ZMBN**), and the collection of the Illinois Natural History Survey, Champaign, Illinois (**INHS**), as indicated in the species descriptions.

## ﻿Results

### ﻿Taxonomic overview

**Figures 4, 5.**
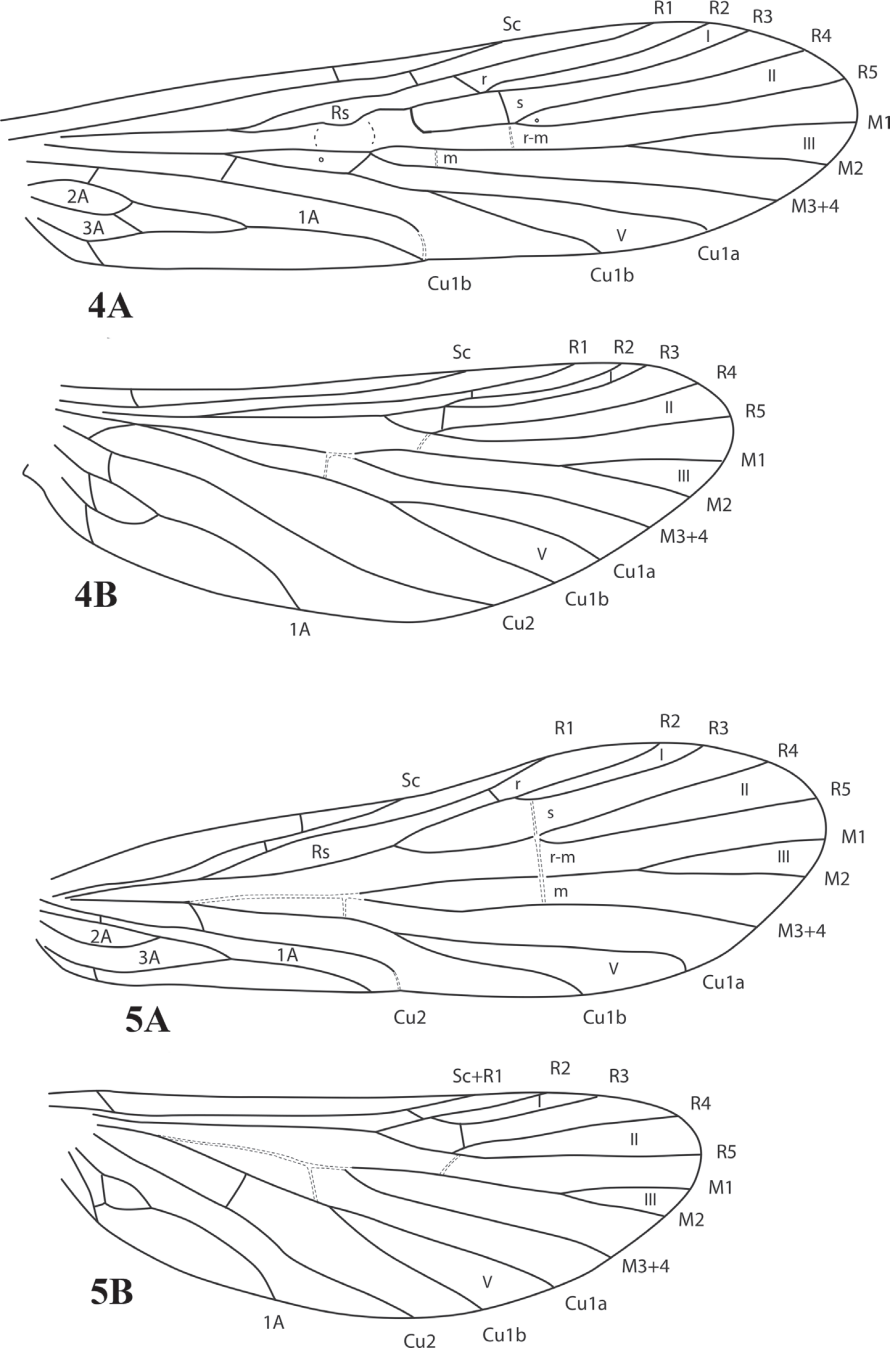
**4***Chimarramarginata* (Linnaeus), *marginata* Group, wings **A** forewing **B** hind wing **5***Chimarraankylis* sp. nov., *georgensis* Group, wings **A** forewing **B** hind wing.

The species described here can be placed into two well-defined species groups, the *marginata* Group and the *georgensis* Group. The *marginata* Group includes species previously placed in the *digitata* group (Blahnik et al. 2009, 2012), but is more broadly defined to include *Chimarramarginata*, the type species for the genus *Chimarra*, and additional species and lineages basal to this group, specifically including species in which the anal loops of the forewing have a crossvein so that the 2A vein appears to be “forked” apically (Fig. 4). In the *georgensis* Group, but also in the *minuta* and *tsudai* Groups of Asia and the subgenera *Curgia* and *Otarrha* of the Americas, the usual configuration is for both the 2A and 3A veins of the forewing to be looped to the 1A vein, that of the 3A vein distal to the 2A; thus, no crossvein is apparent (Fig. 5). The character state is usually easy to ascertain (with the removal of some of the setae covering the wing), because the anal veins are found on the dorsal margin of the wing, just behind the head, when the wings are folded over the body. This character state appears to be unique to the genus *Chimarra*; in taxa in other families in which a crossvein is absent between the anal veins of the forewing, the 3A vein is looped to the 2A vein. In some genera of Trichoptera, a crossvein may also occur between the 1A and 2A veins. This is ostensibly also the situation in the *marginata* Group, in which a crossvein occurs between the 1A and 2A veins, near the terminus of the 2A vein, often giving the vein the appearance of being “forked” apically. The significance of this difference, and the probability that the character state in the *marginata* Group represents a character reversal, was discussed in a previous paper (Blahnik et al. 2012). Unfortunately, even for described species in which the forewing is illustrated, the character state is often misrepresented, so it is prudent to be cautious about assigning species to these species groups without actually examining specimens. A few taxa may also be homoplasious for one or another character state, but this appears to be uncommon.

Most of the mainland African species of the *marginata* Group can be placed into four species diverse subgroups, the *fallax*, *kenyana*, *minima*, and *ruficeps* subgroups. The *fallax* and *minima* subgroups were proposed and populated by Gibon (2018 and 2015, respectively). The *fallax* subgroup is more completely treated in the current work and the *kenyana* and *ruficeps* subgroups are newly proposed here. A number of additional small subgroups from Africa with only one to several species also belong in the *marginata* Group. Other subgroups of the *marginata* Group are broadly represented in the Asian, Australian (including New Guinea and the Pacific Islands), and American faunas. Most of the various species groups recognized from these regions would be considered subgroups of the *marginata* Group, under the usage employed here.

The *georgensis* Group was recognized by Blahnik et al. (2012) in the description of a new species from Vietnam, without circumscribing the African members of the group beyond *Chimarrageorgensis* itself. *Chimarrageorgensis* Barnard is the designated type species for the genus *Chimarrhafra*Lestage (1936), which was synonymized under *Chimarra* by Ross (1956). The genus was specifically designated for species in which the R_1_ vein of the hind wing is lost (or fused to the subcosta), the stem of the Rs in the forewing is straight, and the chord of the forewing (composed of the *s*, *r-m*, and *m* crossveins) is unpigmented and linear (Fig. 5). Except for the first character, which exhibits a good deal of homoplasy within the genus *Chimarra*, the character states are probably plesiomorphic. African species of the *georgensis* Group were more completely listed by Gibon (2018), when describing a new species of the group from Madagascar. The list is expanded here to include two subgroups, the *georgensis* subgroup, including the species listed by Gibon (2018), and the *evoluta* subgroup, including three previously described species. An additional described taxon is recognized as belonging to the *georgensis* Group, but not assigned to subgroup. The 20 new species of the *georgensis* Group described in this paper greatly expand the known diversity of the two subgroups; the number includes two additional new species that were not assigned to subgroup.

In addition to new species described in this paper, other species from Africa and Madagascar were also assessed for their relationships. A list of the species and their assignment to subgroups within the major species groups is found in Table 1. The list is followed by taxa that were left unassigned to subgroup. The subgroups recognized are considered preliminary and provisional. A discussion of the subgroups, for new species described in this paper, precedes the species descriptions. Species subgroups not treated in this paper: The *marginata* subgroup includes only *C.marginata* itself. The species is widespread in Europe, but also occurs in the northern part of Africa. A molecular assessment of its placement within the genus by Wahlberg and Johanson (2014) supported its relationship to a subset of Asian taxa, which, collectively, were related to New World species in the subgenus Chimarra. In the study by Kjer et al. (2014), *C.marginata* was closest to African taxa, but there was no convincing support for this relationship. The species seems to be relatively isolated from other species of the subgenus occurring in Africa. The *apiconigra* subgroup includes two species from Madagascar that were acknowledged to be closely related at the time of their original descriptions. This subgroup is recognized to establish a nuclear subgroup to which other taxa may belong. *Chimarrablahniki* may belong here, as noted at the time of its description, but is not included because of its somewhat different venational attributes (less curved Rs vein of the forewing). *Chimarraapiconigra* was also compared to *C.cognata* at the time of its description, which is in the *minima* subgroup. An overall similarity to this subgroup is acknowledged, but the phallic spines in members of the *apiconigra* subgroup are relatively short and simple, rather than elongate and modified, and the overall shape of the inferior appendages is different from species in the *minima* subgroup. The *lehibemavo* subgroup, from Madagascar, was established and treated comprehensively by Gibon (2017). The *pondoensis* subgroup contains only two species from South Africa, both with elongate, narrow lateral lobes of tergum X and short curved phalli. Venational characters of the subgroup suggest its placement in the *marginata* Group, but possibly in a relatively basal position, since the curvature of the Rs vein of the forewing is minimal. Its relatively primitive characters may account for the original placement of *C.crocifera* within *Chimarrhafra*, considered by Morse (1974) a subgenus of *Chimarra*.

### ﻿Species descriptions

#### The *marginata* Group

The first characterization of evolutionary relationships in the genus *Chimarra* was provided by Ross (1956), who used exemplars at hand to characterize various lineages. One of the lineages characterized was the *digitata* type, based on a species collected from India. Characters of especial note were the reduction of the sensilla of the lateral lobes of tergum X to exactly two and the development of a membranous mesal lobe of tergum X. Ross noted that some ancestral form with the same character set invaded the New World to give rise to the many species lineages found there. Usage of the name, as the *digitata* lineage, was continued by Blahnik (1998) in a revision of New World species of the subgenus Chimarra, partly because of an agreement that American species of the subgenus were closely related to Asian species. The *digitata* group was more formally characterized and discussed by Blahnik et al. (2009, 2012), in describing new species of *Chimarra* from Borneo and Vietnam. However, specific membership in the group was deferred, except for the taxa directly treated. It was already realized that *Chimarramarginata*, as the type species for the subgenus Chimarra, might be a more appropriate species around which to characterize this group, since it also possesses many of its defining characters. In contrast, some species assigned to the subgenus Chimarra, both in Asia and Africa, have more plesiomorphic characters. This is the first formal recognition of the *marginata* Group as a lineage within the subgenus Chimarra, as distinct from more plesiomorphic lineages in the same subgenus, which are assigned to other species groups. The group name more or less replaces the *digitata* group, as previously characterized (Blahnik et al. 2009, 2012), although somewhat more broadly defined and encompassing a wider number of taxa from throughout Asia, Australia, Africa, and the New World. A number of these have been referred to various species groups in previous regional literature, which are here recognized as subgroups within the *marginata* Group.

The first, and most useful, character defining the *marginata* Group is that the 2A vein of forewing has a crossvein to 1A (2A apparently forked apically). This character would appear to be a primitive character state; the probability that it is a secondary and rederived character state was discussed by Blahnik et al. (2012). Outside of the *marginata* Group, it only regularly occurs in the subgenus Chimarrita of the New World. Other taxa within *Chimarra*, including the subgenera *Curgia* and *Otarrha*, but also the *tsudai*, *minuta*, and *georgensis* Groups of the subgenus Chimarra, have both the 2A and 3A veins looped to the 1A, that of the 3A distal to the 2A. Thus, no crossvein is apparent. Some homoplasy occurs, as might be expected for a character reversal, but appears to be very infrequent. An example of this may be the type species for the subgenus Curgia, the wings of which were illustrated by Flint (1998: fig. 5) and clearly have a crossvein present between the 1A and 2A veins of the forewing. However, an examination of species from all of the species groups of the subgenus recognized by Flint, including other species in the same species group as the type species for the subgenus, revealed no other examples of this.

A character usually used to define the subgenus Chimarra, and diagnostic when it occurs, is for the Rs vein of the forewing to be inflected or curved. The character state is found in *C.marginata*, in which it is particularly pronounced, and also commonly occurs in many other species of the group. However, it is generally acknowledged to be absent in species of the *georgensis* Group, which, partly on this basis, were assigned to the genus *Chimarrhafra* by Lestage (1936). The conclusion drawn from this is that an inflection of the Rs vein is not a synapomorphy for the entire subgenus but must have arisen within some lineages. Blahnik et al. (2009, 2012) used the presence of an inflection or loop in the Rs vein of the forewing as one character defining the *digitata* group, here reassigned to the *marginata* Group. The difficulty of applying this character was commented on by Wahlberg and Johanson (2014), who found a loop or inflection of the Rs vein of the forewing to be frequently absent in many of the taxa assigned to the *marginata* Group, based on the primary character defining the group (a crossvein between the anal veins), as discussed above. The discussion below is meant to address this issue.

The presence of an inflected Rs vein is often accompanied by other character state changes and can be useful for assessing relationships within the subgenus when the characters are considered in combination. Characters frequently occurring in the *marginata* Group, in addition to a sinuously inflected Rs vein, include a distinct sclerotized node, either at the point of inflection or extending into the cell below. The R_1_ and/or base of the M vein may also be sinuously inflected. Often, the basal fork of the discoidal cell also is thickened, and the fork loses its strict symmetry. Other character changes that are commonly associated with this character development include a change of the *s* crossvein to a character state in which it is pigmented and more evidently developed, rather than unpigmented or hyaline and weakly developed, and the movement of the *m* crossvein of the forewing to a position more proximal than the *s* and *r-m* crossveins, thus making the chord no longer linear. These associated character states are found in *C.marginata* (Fig. 4) and are also both common and widespread in other species of the *marginata* Group throughout its geographic distribution. However, in various taxa of the *marginata* Group, the Rs vein may be rather weakly inflected, or may even appear to be almost straight, either reflecting a more primitive state in those taxa, or a character reversal. Probably both explanations apply in different cases. Part of the difficulty, in this case, may be in the application or assignation of a character state, especially when the wing is mounted on a slide. At least a slight tendency for the Rs vein to be bowed outward from the plane of the wing seems to be inherent in the entirety of the subgenus Chimarra, including even species of the *georgensis* Group, in which the vein is generally characterized as being straight. The same is true of the *minuta* Group from Asia. In the *tsudai* Group of Asia, an inflection is generally noticeable, but is variable. Other associated characters states discussed above may sometimes occur, either within or among these groups. These are probably parallel developments.

Described species of the *marginata* Group have been placed into various subgroups, as listed below. A number of species from Africa are difficult to assign to subgroup, based on literature descriptions and illustrations. Most are probably members of the *marginata* Group. Some may represent additional subgroups not represented by species from Ghana and Tanzania.

#### The *cara* subgroup

**Included species.***Chimarrabispinosa* Gibbs, 1973; and *C.cara* Mosely, 1936.

Members of this subgroup have the same generalized features that characterize the *marginata* Group, but the *m* crossvein of the forewing is more or less continuous with the *r-m* and *s* crossveins, although strongly angled; thus, it is not displaced proximally as in most described species in the *marginata* Group as a whole. A similar character state occurs in the *leta* subgroup, discussed below. Like members of the *kenyana* subgroup, the species have a tergum X with lateral lobes that are simple (neither divided, nor with ventral periphallic processes) and have two sensilla at the apex of a digitate lobe that emerges either basally or midlaterally. Members of both of these groups also have a relatively short ventral process on sternum IX. The species of the *cara* subgroup are characterized by short rounded inferior appendages, with a pronounced cusp on the mesal surface, and also by an angular preapical projection on tergum X. The latter is a relatively common feature in various species of the subgenus Chimarra, also found in various Asian, American, and other African species, and may represent a primitive or plesiomorphic character for the *marginata* Group as a whole. If so, the character has been lost in many lineages. The species placed here have two small and more or less symmetrically placed endothecal spines, but the endotheca seems to lack a very distinctly textured region or tract with minute spines.

In the molecular analysis of species in the genus *Chimarra* by Wahlberg and Johanson (2014), *C.calidopetoris* Wahlberg, Espeland & Johanson was a close taxon to *C.bispinosa*, also included in their study. Although we do not question the relative placement of the taxa in their study, the characters presented in the description of the *C.calidopectoris* are not completely congruent with the definition of the *cara* subgroup, as defined here. We therefore prefer to defer placement of *C.calidopectoris*. Perhaps it would be better treated in a subgroup of its own.

##### ﻿ Chimarra bispinosa

Taxon classificationAnimaliaTrichopteraPhilopotamidae

Gibbs, 1973

25461EE0-6C4E-5360-B080-377A1AC45D95

Fig. 6A–F

Chimarra bispinosa Gibbs, 1973: 367, figs 8–10.
Chimarra bispinosa Gibbs: Gibon 1985: 28 (distribution: Ivory Coast).

###### Material examined.

Ghana – **Brong Ahafo Reg.** ● 2♂♂1♀; Kintampo, Saunders Waterfall; 8°05'23"N, 1°41'50"W; 19 June 1993; JS Amakye & J Kjærandsen leg.; light trap; ZMBN ● 1♂; same collection data as for preceding except 13 Feb. 1993; J Kjærandsen leg.; sweep net; ZMBN. – **Central Reg.** ● 4♂♂9♀♀; Kakum Forest Reserve; 5°21'N 1°22'W; 8–15 June 1994; T Andersen leg.; Malaise trap; ZMBN ● 1♂1♀; same collection data as for preceding except 8 Nov. 1994; light trap; ZMBN. – **Eastern Reg.** ● 1♂; Boti Falls; 6°11'40"N, 0°13'05"W; 24 Feb. 1993; JS Amakye & J Kjærandsen leg.; light trap; ZMBN ● 1♂; same collection data as for preceding; UMSP ● 1♂; Kibi, Subri stream; 6°10'N, 0°33'W; 5 Nov. 1993; J Kjærandsen leg.; light trap; ZMBN. – **Volta Reg.** ● 2♀♀; Wli, Agumatsa waterfall, station # 1^A^; 7°07'29"N, 0°35'31"E; 5–14 Mar. 1993; JS Amakye & J Kjærandsen leg.; Malaise trap; ZMBN ● 1♂6♀♀; same collection data as for preceding except 12–21 Nov. 1993; J Kjærandsen leg.; ZMBN ● 2♂♂; same collection data as for preceding except station # 1^B^; 11–14 Mar. 1993; JS Amakye & J Kjærandsen leg.; ZMBN ● 8♂♂; same collection data as for preceding except 12–21 Nov. 1993; J Kjærandsen leg.; ZMBN ● 3♂♂3♀♀; same collection data as for preceding except station # 3^A^; 4–13 Mar. 1993; JS Amakye & J Kjærandsen leg.; ZMBN ● 1♀; same collection data as for preceding except 11–20 Nov. 1993; J Kjærandsen leg.; ZMBN ● 1♂; same collection data as for preceding except station # 3^B^; 4–7 Mar. 1993; JS Amakye & J Kjærandsen leg.; UMSP ● 226♂♂95♀♀; same collection data as for preceding except station # 3; 10 Mar. 1993; JS Amakye & J Kjærandsen leg.; light trap; ZMBN ● 1♀; same collection data as for preceding; UMSP ● 113♂♂438♀♀; same collection data as for preceding except 17 Nov. 1993; J Kjærandsen leg.; ZMBN ● 1♂1♀; same collection data as for preceding except station # 6; 11 Mar. 1993; JS Amakye & J Kjærandsen leg.; ZMBN ● 2♀♀; same collection data as for preceding except 20 Nov. 1993; J Kjærandsen leg.; ZMBN.

###### Diagnosis.

**Figure 6.**
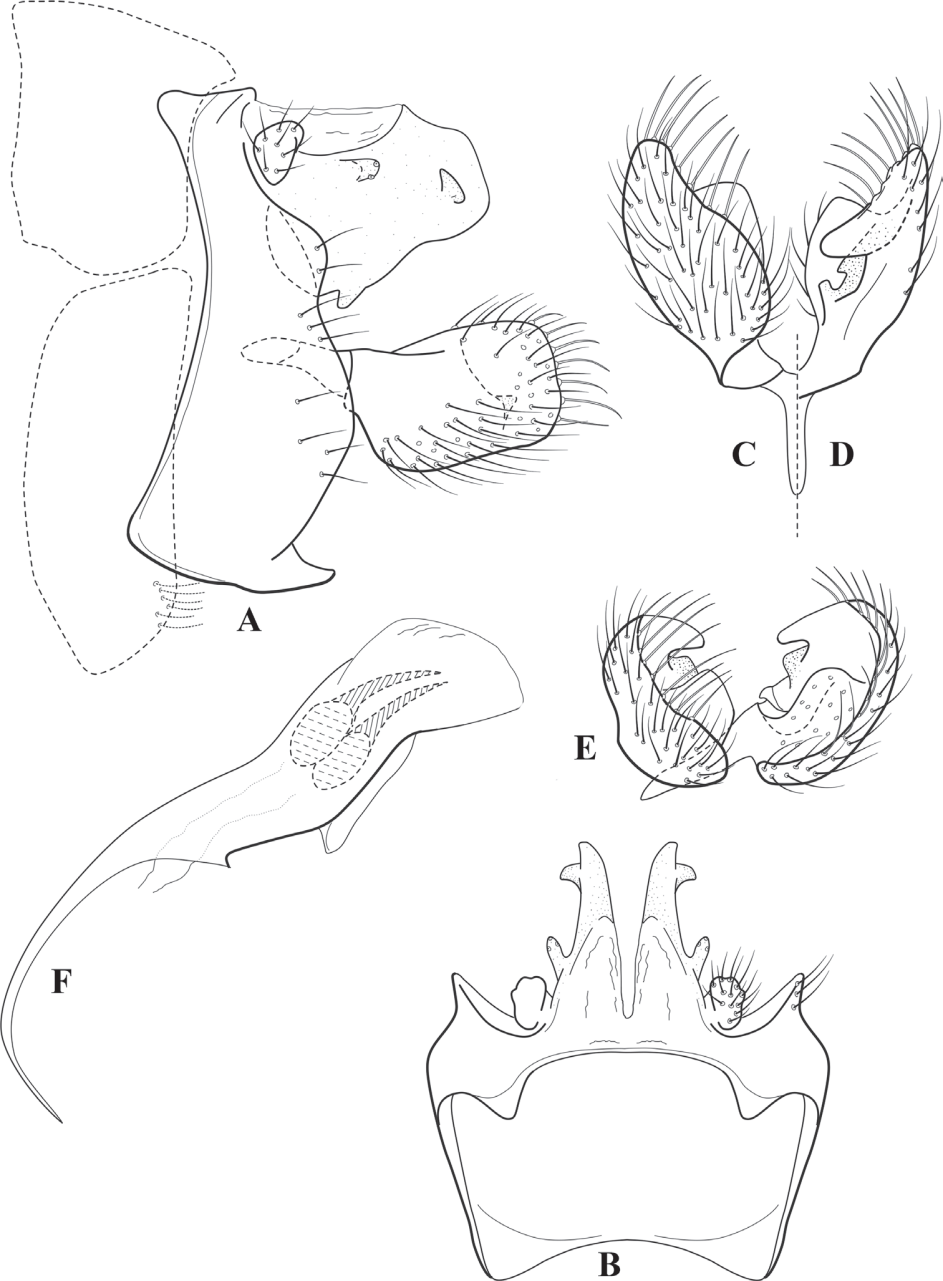
*Chimarrabispinosa* Gibbs, ♂ genitalia **A** lateral **B** dorsal, segments IX and X **C** inferior appendage, ventral **D** inferior appendage, dorsal **E** inferior appendage, oblique lateral **F** phallus, lateral.

The two species included in this subgroup are very similar. The differences, as evident from the original illustrations, lie mostly in the shape and length of the lateral lobes of tergum X, the relative shape of the apices of these lobes, as well as in the more bulbous bases of the phallic spines in *C.bispinosa*. It is also possible that the overall shape of the inferior appendages in *C.cara* are slightly more rounded. The specimen illustrated here (Fig. 6A–F) most closely conforms to *C.bispinosa* and we have identified it as such. Any minor differences from the original illustration should not be accorded significance, at least until the variation within the two known species of the subgroup is better assessed.

###### Redescription.

***Adult.*** Overall color (in alcohol) light brown, appendages paler, head and prothorax slightly darker (anterior and posterior setal warts pale and contrasting). Head short and rounded (postocular parietal sclerite short). Palps relatively short; maxillary palp with 1^st^ segment very short (approximately as long as wide), 2^nd^ segment short (slightly > 2× 1^st^), apex with cluster of ~ 12 stiff setae, 3^rd^ segment moderately elongate, distinctly longer than 2^nd^, 4^th^ segment short (shorter than 2^nd^), 5^th^ segment slightly longer than 3^rd^. Forewing length: male, 5.5–7.0 mm; female, 6.5–7.5 mm. Fore- and hind wings with forks I, II, III, and V present. Forewing with R_1_ somewhat sinuous, stem of Rs inflected at past midlength, with distinct node at inflection, extending into cell below, basal fork of discoidal cell distinctly enlarged, fork asymmetric, length of cell slightly > 2× width, fork I subsessile, fork II sessile, *r* crossvein diagonal, intersecting discoidal cell at fork I, chord nearly linear (*s* and *r-m*, crossveins linear, *m* crossvein diagonal, continuous with *r-m*), *s* pigmented (like wing), *r-m* and *m* hyaline, 2A with crossvein, (apparently forked apically to 1A and 3A). Hind wing with R_1_ narrowly parallel to subcosta, forks I and II subsessile, fork III distal and relatively wide, anal loop small and incomplete (not joining 1A). Forelegs with apical tibial spur short; male with foretarsi unmodified, claws small and symmetrical.

***Male genitalia*.** Segment VIII relatively short, tergum wider dorsally, sternum without posteroventral projection. Segment IX, in lateral view, short, with anteroventral margin weakly, angularly inflected in basal 1/4, concavely narrowing dorsally; tergum very short dorsally, with short anterior apodemes; dorsal margin very narrow, but continuous mesally between apodemes; posterior margin with inferior appendages mounted relatively high on segment, below mid-height, margin very weakly produced below preanal appendages, basally with short, subtriangular, posteriorly projecting, ventral process; anterior margin of sternum subtruncate as viewed dorsally or ventrally, slightly concave mesally. Lateral lobes of tergum X short and simple in form, without periphallic processes, each lobe with short, acute, preapical lateral projection, basally with very short, digitate process with 2 apical sensilla; mesal lobe of tergum X membranous and divided mesally, continuous with sclerotized lateral lobes. Preanal appendages short and knob-like, slightly flattened, membranous basally. Inferior appendage very short and rounded apically, with weak basal inflection, marginally with elongate setae; as viewed ventrally or caudally, with distinct cusp on mesal surface and very short, mesally directed, basodorsal process. Phallic apparatus relatively short, phallobase tubular, with usual basodorsal expansion, ventral margin slightly bulging, with weakly sclerotized ventral projection; endotheca with two short and nearly symmetrically positioned spines, each with enlarged base, membrane not or only weakly textured, phallotremal sclerite complex not evident.

###### Distribution.

Ghana, Ivory Coast.

#### The *fallax* subgroup

**Included species.** ?*Chimarrabettinae* Marlier & Marlier, 1982; *C.calundoensis* Marlier, 1965; *C.dybowskina* Navás, 1931; *C.elga* Mosely, 1939; *C.falcifera* Jacquemart, 1966; *C.fallax* (Ulmer, 1912); *C.jacquemarti* sp. nov.; *C.lanceolata* sp. nov.; *C.lejea* Mosely, 1948; *C.mauritania* Jacquemart, 1967; *C.robynsi* (Jacquemart, 1967); *C.togoana* (Ulmer, 1907); and *C.travei* Jacquemart, 1963.

The *fallax* subgroup of *Chimarra* was first proposed by Gibon (2018), who also gave a preliminary list of species. The list of included species is somewhat enlarged here, and several species synonymized. A number of described species, particularly those with an upturned, acute, sclerotized tergum X and ventral periphallic processes, are very similar to one another. Available illustrations and descriptions make it difficult to identify the species confidently, since the species were never treated comparatively, nor were the characters useful in diagnosing similar species discussed. This is complicated by what seems to be a certain degree of intraspecies variability. The subgroup is obviously in need of a revision, including examination of material from throughout Africa and also holotype specimens. Ideally, the two should be done in conjunction with one another, but this is outside the scope of the current paper. The following taxonomic treatment of species from Ghana and Tanzania, which includes many of the morphological forms represented in the literature, is offered in lieu of a formal revisionary treatment and to simplify the identification of known species. In the process, we have synonymized some of the described species. It is possible that a future comprehensive revisionary study will reveal that one or more of these forms should be resurrected. As it stands, however, existing names serve little purpose in allowing taxonomists to identify species. The interim taxonomy presented here is meant to lead to a more stable and meaningful use of names.

Species of the *fallax* subgroup are characterized by an elongate ventral process on segment IX, which is distinctly flattened apically so that the apex appears more or less acute in lateral view and rounded in ventral view. The ventral surface of the apex is often roughened and more or less file-like or rasp-like. Most species have the dorsal margin of segment IX obsolete (membranous), as in the *minima* subgroup. Also, the posteroventral margin of sternum VIII is distinctly projecting, in at least the majority of species, and the length of this segment is quite short. The ventral projection of sternum VIII should probably not be interpreted as a ventral process. This character was used to assign some species to this subgroup, rather than to the *ruficeps* subgroup, which seems to be closely related. The lateral lobes of tergum X, in many of the species, are formed into curved, usually upright, spine-like, and distinctly sclerotized lobes. In at least some instances, it is obvious that these lobes bear the pair of sensilla usually found on the lateral lobes of species of the *marginata* Group of *Chimarra*. However, in addition to these spine-like lobes, there are paired ventral processes, referred to here as periphallic processes, but whether they originated by a lateral division of the lateral lobes of tergum X, or represent de novo outgrowths from the periphallic membrane is uncertain; in some species these are almost completely ventral to the phallic apparatus and fused basally, but usually with the apices separated, thus providing a ventral source of support for phallus. The prominent development of these processes in some species of the subgroup seems to have a correlation with the greatly projecting ventral apex of the phallobase, which also characterizes some species. In the species from Mauritius, *C.mauritania* Jacquemart and *C.travei* Jacquemart, the periphallic processes are less developed, but distinct, and in *C.togoana* (Ulmer) and *C.lanceolata* sp. nov., and also in species of the *ruficeps* subgroup, inferred to be the sister taxon of the *fallax* subgroup, tergum X is divided from the posterior margin into dorsal and ventral lobes, possibly suggesting the origin of the periphallic processes.

The subgroup is probably most closely related to the *C.ruficeps* subgroup and the placement of individual species in one group or the other may be equivocal in some cases. The *ruficeps* subgroup also has species with an elongate ventral process on segment IX, but the apex of the process is broadened, as viewed laterally, and usually has its ventral margin formed into a pad of short, stiff setae or spines. Most described members of the *ruficeps* subgroup have a similar coloration, with a yellowish or orangish colored head and thorax, contrasting with darker wings. However, color attributes are difficult to ascertain in specimens that have been in alcohol for some time and may not be consistent for all members of the group. Species of the *ruficeps* subgroup also lack a ventral projection from sternum VIII.

In the study by Wahlberg and Johanson (2014), species that we have assigned to the two subgroups placed among species of *Chimarra* from Australia and the Pacific Islands, as separate clades in the parsimony analysis and as sister clades in the Bayesian analysis. Additional undetermined or undescribed species of the clades were also included in their study. The species from Australia and the Pacific Islands otherwise constituted a monophyletic group. Despite the overall support for this placement in their Bayesian analysis, the inclusion of these two African clades within the Australian lineage should probably be considered a hypothesis requiring further confirmation. Nevertheless, the relative proximity of the two clades in the analysis can probably be taken as an indication of their relationship to one another.

##### ﻿ Chimarra calundoensis

Taxon classificationAnimaliaTrichopteraPhilopotamidae

Marlier, 1965

7C210339-B685-5FB4-9E01-76EE6DE8B920

Fig. 7A–E

Chimarra calundoensis Marlier, 1965: 26, fig. 1.

###### Material examined.

Ghana – **Central Reg.** ● 1♂; Kakum Forest Reserve; 5°21'N 1°22'W; 8–15 Nov. 1994; T Andersen leg.; Malaise trap; UMSP.

###### Diagnosis.

Phallobase with ventral apex greatly produced and strongly bent, apex rounded; phallic spines both rather short; inferior appendage tapered, bent, acute apically, cusps of ventromesal margin not evident in lateral view.

*Chimarracalundoensis* is most similar to and most likely to be confused with either *C.dybowskina* or *C.falcifera*. However, diagnoses of other species in the subgroup should be considered to eliminate other possibilities. *Chimarracalundoensis* resembles *C.dybowskina* in having the apicoventral lobe of the phallobase strongly bent and in having the dorsal lobe of the inferior appendages at least somewhat bent. It differs in that the ventral apex of the phallobase is rounded, rather than subtruncate, and the dorsal lobes of the inferior appendages are more tapering and less distinctly bent. Additionally, the phallic spines are slightly shorter than in *C.dybowskina*. We considered synonymizing *C.falcifera* Jacquemart with *C.calundoensis*; it seems to differ primarily in having the ventral apex of the phallobase less distinctly bent. However, the illustration of the hind wing of *C.falcifera* provided by Jacquemart (1966b: fig. 7C) indicates an absence of fork III. This is not the case in *C.calundoensis*, or any other species of the *fallax* group investigated. Individual, sometimes unilateral, variations in venational forking are not particularly unusual. The matter should probably be investigated before a synonymy is made.

**Figure 7.**
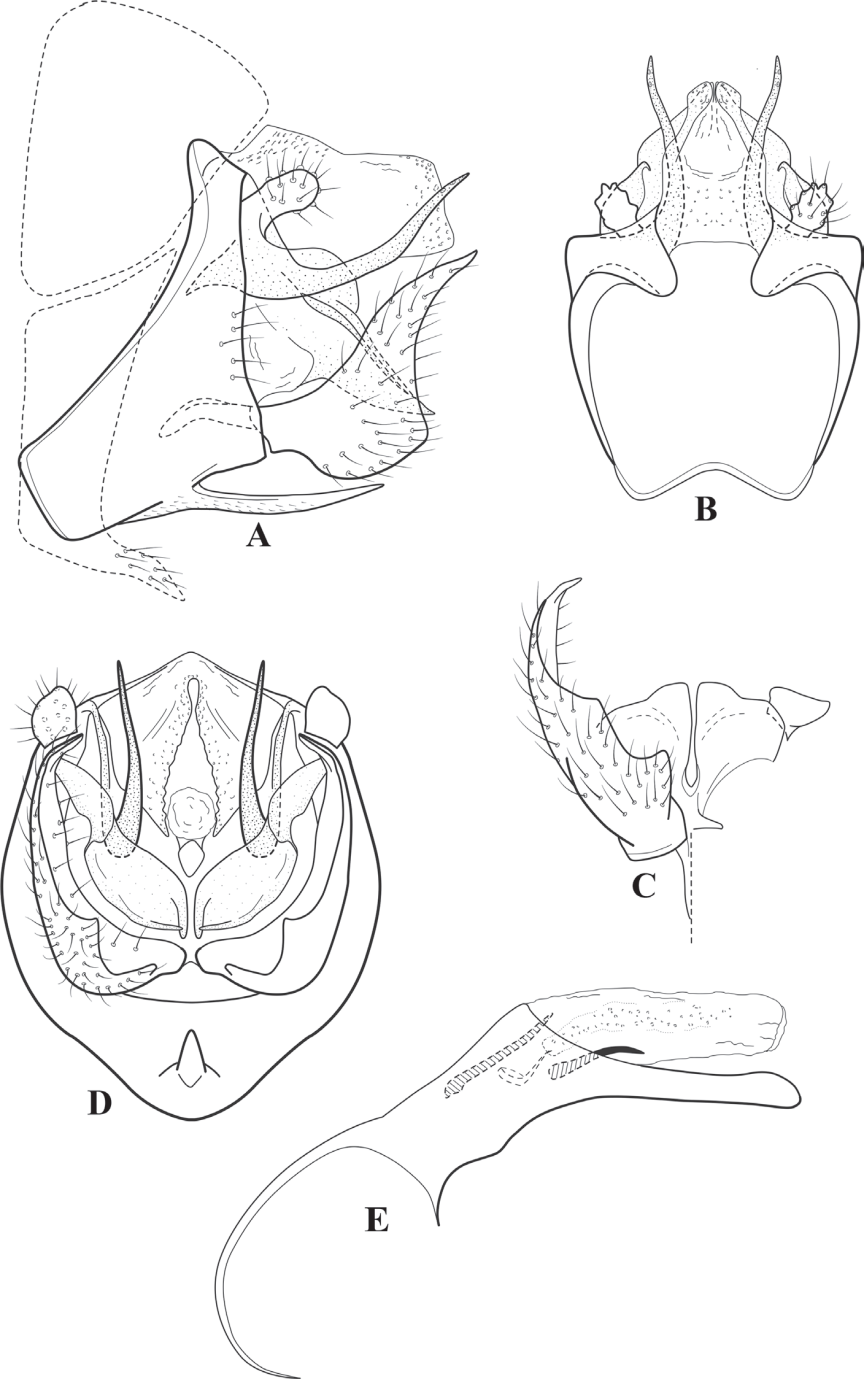
*Chimarracalundoensis* Marlier, ♂ genitalia **A** lateral **B** dorsal, segments IX and X **C** inferior appendage and periphallic processes, caudal **D** caudal **E** phallus, lateral.

###### Redescription.

***Adult.*** Color of head, prothorax, and appendages (in alcohol) yellowish, mesothorax, body, and spurs yellowish brown. Head relatively short (postocular parietal sclerite < 1/2 diameter of eye). Palps relatively short; maxillary palp with 1^st^ segment very short (approximately as long as wide), 2^nd^ segment relatively short (< 3× 1^st^), apex with small cluster of stiff setae, 3^rd^ segment only slightly longer than 2^nd^, 4^th^ segment very short (shorter than 2^nd^), 5^th^ segment subequal to 3^rd^. Forewing length: male, 5.4 mm. Fore- and hind wings with forks I, II, III, and V present. Forewing with R_1_ sinuous, stem of Rs inflected at past midlength (with distinct small node at inflection), basal fork of discoidal cell distinctly enlarged, fork asymmetric, length of cell ~ 2× width, fork I strongly subsessile, fork II sessile, *r* crossvein diagonal, intersecting discoidal cell at past midlength, just before fork I, *s* and *r-m*, crossveins linear, *m* crossvein more proximal, *s* pigmented (like wing), *r-m* and *m* crossveins hyaline, 2A with crossvein (apparently forked apically to 1A and 3A). Hind wing with R_1_ narrowly parallel to subcosta, forks I and fork II subsessile, fork III distal and relatively wide, anal loop small. Forelegs with apical tibial spur short; male with tarsi unmodified, claws small and symmetrical.

***Male genitalia*.** Segment VIII with sternum relatively short, ventrally with distinct projection from posterior margin, tergum wider, expanded dorsally (~ 2× width of sternum at base). Segment IX, in lateral view, with anteroventral margin moderately produced, anterior margin with angular inflection in ventral ¼, weakly concave and narrowing dorsally; tergum very short dorsally, with short anterior apodemes, obsolete mesally between apodemes; posterior margin nearly linear; ventral margin sloping, more or less linear, with elongate, narrow ventral process at approximately midlength, apex of process acute as viewed laterally, rounded as viewed ventrally, apicoventral surface of ventral process roughened and file-like; anterior margin of sternum, as viewed dorsally or ventrally, subtruncate, slightly concave mesally. Lateral lobes of tergum X formed into dorsally curved, sclerotized, spine-like processes, bearing two preapical sensilla; mesal lobe of tergum X membranous, moderately elongate; ventrally with strongly projecting, paired, sclerotized, periphallic processes, subtending phallic apparatus. Preanal appendages short and knob-like, constricted basally, membranous basally, but fused laterally to periphallic processes. Inferior appendage with pronounced basal inflection, apex dorsally inflected and strongly narrowing, somewhat posteriorly curved, apex acute; as viewed ventrally, with weakly sclerotized, angular projections or cusps near base and before midlength, projections not or scarcely evident in lateral view; mesal surface without projections or ridges. Phallic apparatus with phallobase tubular, with usual basodorsal expansion, apicoventral margin very strongly projecting, sclerotized, strongly ventrally deflected, apex of ventral projection more or less evenly rounded, as viewed laterally; endotheca with two relatively short and asymmetrically positioned spines, membrane textured with small spines, phallotremal sclerite complex composed of short rod and ring structure.

###### Distribution.

Angola, Democratic Republic of the Congo, Ghana.

##### ﻿ Chimarra dybowskina

Taxon classificationAnimaliaTrichopteraPhilopotamidae

Navás, 1931

05FDEDBA-20B2-57D8-AA7C-12B5E24BCC03

Fig. 8A–E

Chimarrha dybowskina Navás, 1931: 123–124, fig. 61.
Chimarra dybowskina Navás: Fischer 1961: 59; Malicky 2015b: 41 (text), 44 (figure) (distribution: Madagascar); Gibon 2018: 121–122, figs 3A, 5, (distribution: Burkina Faso, Guinea, Ivory Coast, Madagascar, Mali, Togo).
Chimarra divergena Gibbs, 1973: 367–369, figs 5–7. Syn. nov.
Chimarra caboverdensis Nybom, 1960: 1–3, figs A–G. Syn. nov.

###### Material examined.

Cape Verde ● 1♂; Brava, Fajâ d’ Agua; 100 m a.s.l.; 17 Feb. 2007; E Aistleitner leg.; UMSP. Ghana – **Central Reg.** ● 1♂; Kakum Forest Reserve; 5°21'N, 1°22'W; 8 Nov. 1994; T Andersen leg.; light trap; ZMBN. – **Eastern Reg.** ● 1♂1♀; Kibi, Subri stream; 6°10'N, 0°33'W; 5 Nov. 1993; J Kjærandsen leg.; light trap; ZMBN. – **Volta Reg.** ● 1♂; Hohoe, Matvin Hotel; 7°09'43"N, 0°28'31"E; 11 Nov. 1993; J Kjærandsen leg.; at light; ZMBN ● 3♀♀; Wli, Agumatsa waterfall, station # 3; 7°07'29"N, 0°35'31"E; 11–20 Nov. 1993; J Kjærandsen leg.; Malaise trap; ZMBN ● 1♀; same collection data as for preceding except station # 6; ZMBN ● 2♀♀; same collection data as for preceding except station # 7; ZMBN ● 1♀; same collection data as for preceding except station # 8; ZMBN ● 5♂♂15♀♀; same collection data as for preceding except station # 3; 17 Nov. 1993; light trap; ZMBN ● 1♀; same collection data as for preceding except station # 6; 11 Mar. 1993; JS Amakye & J Kjærandsen leg.; ZMBN ● 1♀; same collection data as for preceding except 20 Nov. 1993; J Kjærandsen leg.; ZMBN ● 1♂; same collection data as for preceding; UMSP ● 1♂3♀♀; same collection data as for preceding except station # 10; 19 Nov. 1993; ZMBN ● 3♂♂4♀♀; same collection data as for preceding except station # 12; 16 Nov. 1993; ZMBN ● 1♀; same collection data as for preceding; UMSP.

**Figure 8.**
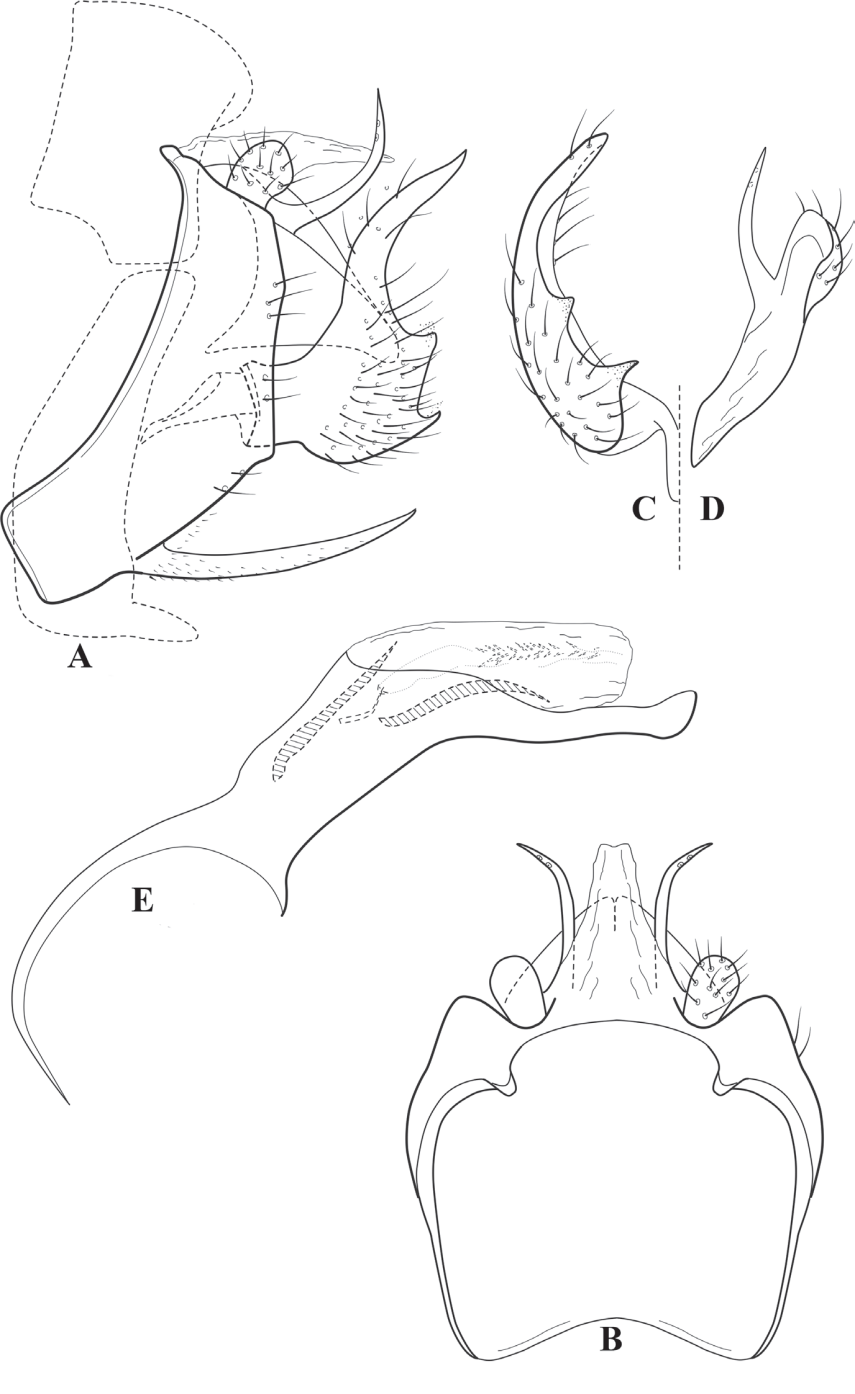
*Chimarradybowskina* Navás, ♂ genitalia **A** lateral **B** dorsal, segments IX and X **C** inferior appendage, caudal **D** tergum X and periphallic process, right caudal **E** phallus, lateral.

###### Diagnosis.

Phallobase with ventral margin greatly produced and strongly bent, apex enlarged and subtruncate, dorsal margin slightly upturned; phallic spines both moderately elongate and narrow; inferior appendage with dorsal projection abruptly narrowed, nearly uniform in width and distinctly bent, apex acute, ventromesal cusps of inferior appendage often both evident in lateral view (character possibly variable or inconsistent).

*Chimarradybowskina* is most similar to *C.calundoensis*, *C.falcifera*, and *C.jacquemarti* sp. nov. The species are best distinguished by differences in the shape and inflection of the dorsal lobe of the inferior appendages, and by the shape of the apex and inflection of the apicoventral projection of phallobase, as well as in the relative length of the phallic spines. Like both *C.calundoensis* and C. *jacquemarti*, the ventral apex of the phallobase is very strongly bent; the apex of the structure is more truncate than in *C.calundoensis*, but bent slightly upward, rather than downward, as in *C.jacquemarti*. The dorsal process of the inferior appendages is generally more uniform in width and more strongly bent in *C.dybowskina* than in the other species, and the cusps of the mesal surface are more likely to be evident in lateral view. The phallic spines are comparable in length to those of *C.jacquemarti*, but slightly longer than in either *C.calundoensis* or *C.falcifera*.

Our illustration closely matches that presented by Gibon (2018), which was based on the holotype of *C.dybowskina* Navás from the Democratic Republic of the Congo. We are less convinced that the illustration of *C.dybowskina* presented by Malicky (2015b) from Nosy Bé, Madagascar is the same species, although admittedly close. The inferior appendage in his illustration has the general shape and form of *C.dybowskina*, but the ventral projection of the phallobase is not as strongly deflexed and the apex is rounded, rather than truncate.

*Chimarradivergena* Gibbs, described from Ghana, has enough of the significant features of *C.dybowskina* to be considered a synonym, especially the strongly deflexed ventral projection of the phallobase, with its apex subtruncate and slightly upturned. There was no available illustration of *C.dybowskina* when it was published. The primary difference is that the mesal cusps of the inferior appendage are not apparent in lateral view in the illustration of *C.divergena*. The difference seems too minor to warrant species status. *Chimarracaboverdensis* is undoubtedly also a synonym, based on the structure of the ventral apex of the phallobase, which, in the illustration of the type, is clearly truncate apically, with the apex slightly upturned. Examination of a specimen collected from Cabo Verde revealed no distinctive differences from *C.dybowskina*. Pending further evidence of its species status, we prefer to consider *C.caboverdensis* Nybom to also be a synonym of *C.dybowskina* Navás.

###### Redescription.

***Adult.*** Overall color (in alcohol) yellowish brown, head and prothorax not lighter, spurs slightly darker. Head relatively short (postocular parietal sclerite ~ 1/2 diameter of eye). Palps relatively elongate; maxillary palp with 1^st^ segment very short (approximately as long as wide), 2^nd^ segment moderately elongate (~ 4× 1^st^), apex with small cluster of stiff setae, 3^rd^ segment slightly longer than 2^nd^, 4^th^ segment short (~ 1/2 length of 3^rd^), 5^th^ segment elongate, slightly shorter than 3^rd^ and 4^th^ combined. Forewing length: male, 5.0–6.0 mm; female 5.5–6.5 mm. Fore- and hind wings with forks I, II, III, and V present. Forewing with R_1_ very slightly sinuous, stem of Rs weakly inflected at past midlength (without distinct node at inflection), basal fork of discoidal cell slightly asymmetric, length of cell ~ 2× width, fork I slightly subsessile, fork II sessile, *r* crossvein diagonal, intersecting discoidal cell at past midlength, just before fork I, *s* and *r-m*, crossveins linear, *m* crossvein more proximal, *s* pigmented (like wing), *r-m* and *m* crossveins hyaline, 2A with crossvein (apparently forked apically to 1A and 3A). Hind wing with R_1_ narrowly parallel to subcosta, fork I and fork II subsessile, fork III relatively distal, anal loop small. Forelegs with tibial spur distinct; male with foretarsi unmodified, claws small and symmetrical.

***Male genitalia*.** Segment VIII with sternum short, ventrally with distinct ventral projection, tergum somewhat wider, expanded dorsally. Segment IX, in lateral view, with anteroventral margin moderately produced, anterior margin with angular inflection at approximately ventral ¼, concavely narrowing dorsally; tergum narrow dorsolaterally, with short anterior apodemes, obsolete mesally between apodemes; posterior margin nearly linear; ventral margin sloping, more or less linear, with elongate, narrow ventral process, apex of process acute as viewed laterally, rounded as viewed ventrally, apicoventral surface of ventral process roughened and file-like; anterior margin of sternum subtruncate as viewed dorsally or ventrally, slightly concave mesally. Lateral lobes of tergum X formed into dorsally curved, sclerotized, spine-like processes, bearing two preapical sensilla; dorsum of tergum X moderately elongate, membranous; tergum ventrally with strongly projecting, paired, sclerotized, periphallic processes, subtending phallic apparatus. Preanal appendages short and knob-like, constricted basally, fused laterally to periphallic processes. Inferior appendage with pronounced basal inflection, apex dorsally inflected and strongly narrowed, distinctly posteriorly curved, apex acute; as viewed ventrally, with distinct sclerotized projections near base and before midlength, generally evident in lateral view; mesal surface without projections or ridges. Phallic apparatus with phallobase tubular, with usual basodorsal expansion, apicoventral margin sclerotized, strongly deflexed and projecting, apex enlarged, subtruncate, with dorsal margin slightly upturned; endotheca with two relatively elongate, asymmetrically positioned spines, membrane textured with small spines, phallotremal sclerite complex composed of short rod and ring structure.

###### Distribution.

Burkina Faso, Cape Verde, Democratic Republic of the Congo, Ghana, Guinea, Ivory Coast, Madagascar, Mali, Togo.

##### ﻿ Chimarra elga

Taxon classificationAnimaliaTrichopteraPhilopotamidae

Mosely, 1939

CFC72F2D-3812-5C3B-83E5-2E0F369FA358

Fig. 9A–D

Chimarrha elga Mosely, 1939: 300–301, figs 20–23.
Chimarra elga Mosely: Fischer 1971: 209.

###### Material examined.

Democratic Republic Of The Congo ● 3♂♂; South Kivu, CRSN Lwiro, Kabindi, Guest House, Site 3; 2°14.270'S, 28°42.907'E; 1.668 m a.s.l.; 27 Sept. 2005; UMSP ● 1♂; same collection data as for preceding except 19 Apr. 2006; UMSP.

###### Diagnosis.

*Chimarraelga* is another species in the *fallax* subgroup belonging to the complex of species with spine-like dorsolateral lobes of tergum X and a phallobase with a projecting and deflexed ventral apex. Among these, *C.elga* is easily diagnosed by the relatively short, flexed dorsal process of its inferior appendage. It is included in the current paper mostly for comparative purposes.

**Figure 9.**
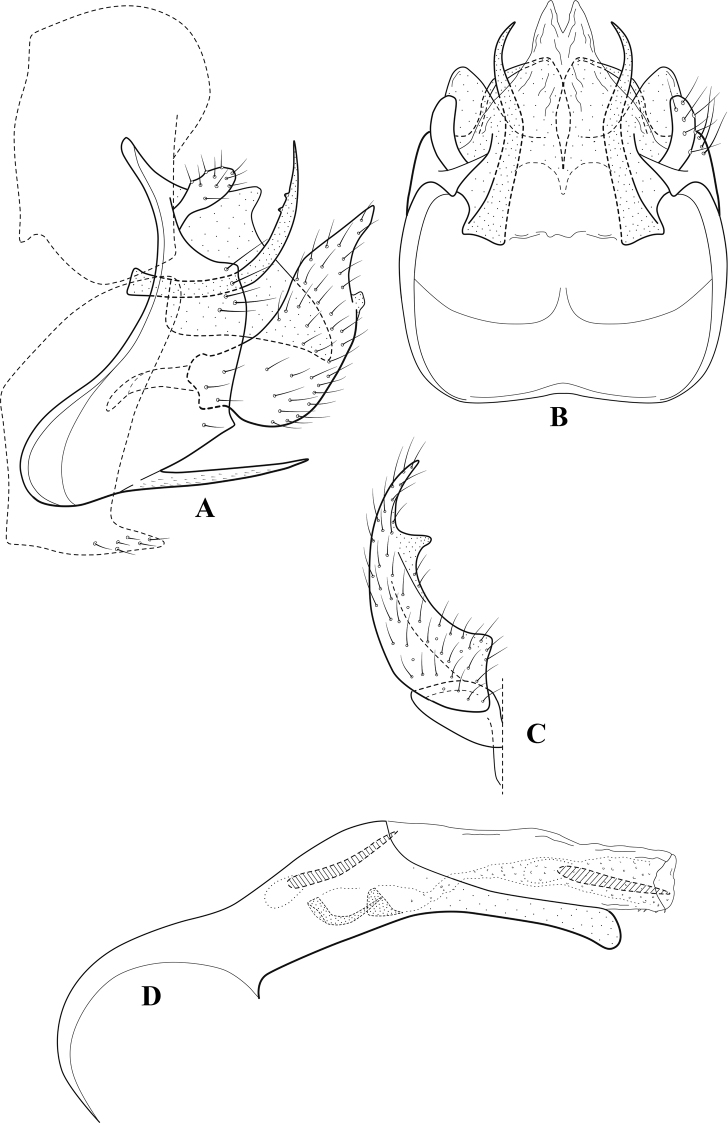
*Chimarraelga* Mosely, ♂ genitalia **A** lateral **B** dorsal, segments IX and X **C** inferior appendage, ventral/caudal **D** phallus, lateral.

###### Redescription.

***Adult.*** Overall color (in alcohol) yellowish brown, vertex of head darker than setal warts. Head relatively short (postocular parietal sclerite < 1/2 diameter of eye). Palps moderately elongate; maxillary palp with 1^st^ segment very short (approximately as long as wide), 2^nd^ segment moderately elongate (~ 3× 1^st^), apex with small cluster of stiff setae, 3^rd^ segment slightly longer than 2^nd^, 4^th^ segment short (~ 1/2 length of 3^rd^), 5^th^ segment subequal to 3^rd^. Forewing length: male, 5.7–6.6 mm. Fore- and hind wings with forks I, II, III, and V present. Forewing with R_1_ slightly sinuous, stem of Rs inflected at past midlength (with distinct node at inflection, not extending into cell below), basal fork of discoidal cell enlarged, slightly asymmetric, length of cell ~ 2 1/2× width, fork I distinctly subsessile, fork II sessile, *r* crossvein diagonal, intersecting discoidal cell at past midlength, just before fork I, *s* and *r-m*, crossveins linear, m crossvein more proximal, *s* pigmented (like wing), *r-m* and *m* crossveins hyaline, 2A with crossvein (apparently forked apically to 1A and 3A). Hind wing with R_1_ narrowly parallel to subcosta, forks I and II subsessile, fork III relatively distal, anal loop small. Forelegs with tibial spur distinct; male with foretarsi unmodified, claws small and symmetrical.

***Male genitalia*.** Segment VIII with sternum relatively short, ventrally with distinct ventral projection, tergum somewhat wider, expanded dorsally. Segment IX, in lateral view, with anteroventral margin moderately produced, anterior margin with rounded projection at approximately ventral ¼, concavely narrowing dorsally; tergum short dorsolaterally, with prominent anterior apodemes, obsolete mesally between apodemes; posterior margin widening below preanal appendages, nearly linear to ventral margin; ventral margin, with elongate, narrow ventral process, apex of process acute as viewed laterally, rounded as viewed ventrally, apicoventral surface of ventral process roughened and file-like; anterior margin of sternum subtruncate as viewed dorsally or ventrally, not or only slightly concave mesally. Lateral lobes of tergum X formed into dorsally curved, sclerotized, spine-like processes, bearing two preapical sensilla; dorsum of mesal lobe of tergum X moderately elongate, membranous; tergum ventrally with strongly projecting, paired, sclerotized, periphallic processes, subtending phallic apparatus. Preanal appendages short and knob-like, constricted basally. Inferior appendage with pronounced basal inflection, apex dorsally inflected, apex of inflection relatively short and strongly narrowed, distinctly posteriorly curved, apex acute; as viewed ventrally, with distinct sclerotized projections near base and before midlength, evident in ventral view, basal projection not evident in lateral view; base very strongly rounded and relatively short; mesal surface without projections or ridges. Phallic apparatus with phallobase tubular, with usual basodorsal expansion, apicoventral margin sclerotized, strongly deflexed and projecting, apex slight enlarged and rounded; endotheca with two moderately elongate, asymmetrically positioned spines, membrane textured with small spines, phallotremal sclerite complex composed of short rod and ring structure and small apical sclerite.

###### Distribution.

Democratic Republic of the Congo, Kenya.

##### ﻿ Chimarra fallax

Taxon classificationAnimaliaTrichopteraPhilopotamidae

(Ulmer, 1912)

EC00EAB7-3FE9-5194-94D6-D1E12EB7C761

Fig. 10A–E

Wormaldia fallax Ulmer, 1912: 84–85, figs 5–8a.
Chimarrha fallax (Ulmer): Marlier 1959 (distribution: Sao Tomé); Fischer 1961: 59; Fischer 1971: 210.
Chimarra lukawei Jacquemart, 1961a: 40, fig. 27a. Syn. nov.
Chimarra lukawei Jacquemart: Jacquemart, 1961b: 230; Jacquemart 1966a: 49, fig. 14A–B; Wahlberg and Johanson 2014: 437–439, figs 1–3; Gibon 2018: 123–124, figs 1B, 3B–D, 5 (distribution: Madagascar).
Chimarra lukawaei [sic] Jacquemart: Morse 2021 [also, many online taxonomic resources using the Trichoptera World Checklist as a source].
Chimarra sp. AK: Gibon & Elouard, 1996: 510.

###### Material examined.

Ghana – **Central Reg.** ● 28♂♂41♀♀; Kakum Forest Reserve; 5°21'N, 1°22'W; 8–15 Nov. 1994; T Andersen leg.; Malaise trap; ZMBN ● 1♂1♀; same collection data as for preceding; UMSP. – **Western Reg.** ● 5♀♀; Ankasa Game Production Reserve; 5°15'N, 2°37'W; 6–12 Dec. 1993; T Andersen & J Kjærandsen leg.; Malaise trap; ZMBN ● 1♂; same collection data as for preceding except 9 Dec. 1993; light trap; ZMBN.

**Figure 10.**
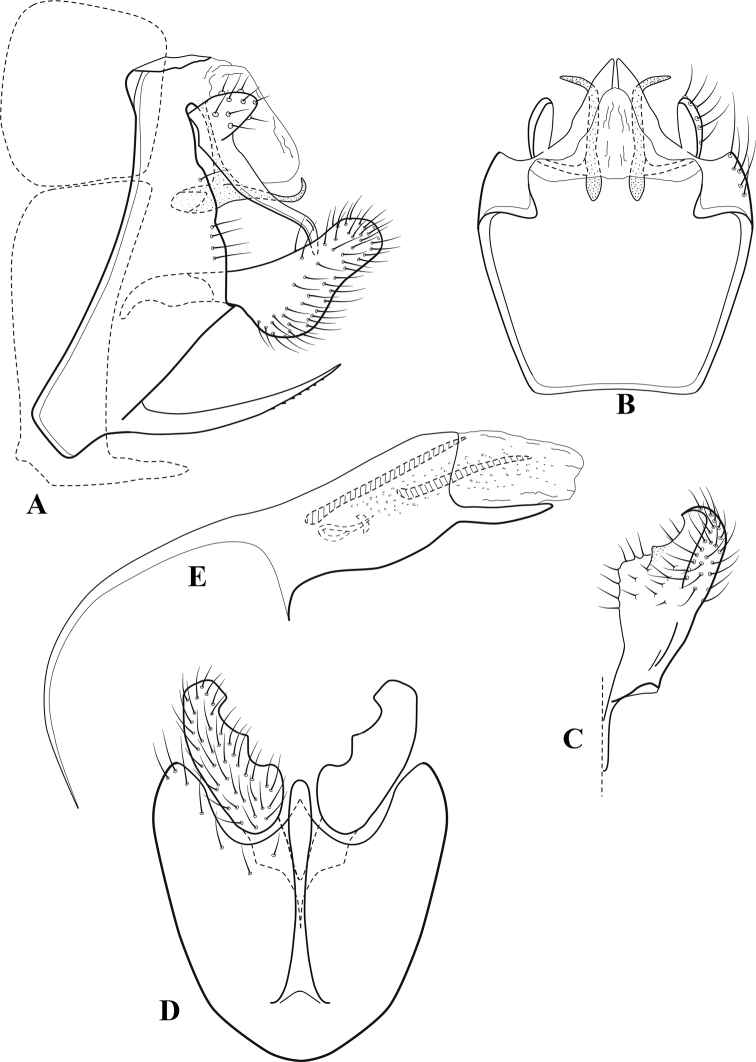
*Chimarrafallax* (Ulmer), ♂ genitalia **A** lateral **B** dorsal, segments IX and X **C** inferior appendage, dorsal **D** segment IX and inferior appendages, ventral **E** phallus, lateral.

###### Diagnosis.

Inferior appendage short and rounded apically; phallobase with ventral apex short and only weakly projecting, not enlarged apically; phallic spines both relatively elongate and narrow, differing in length; periphallic processes fused mesally, comparatively narrow and weakly developed; posteroventral margin of sternum VIII distinctly projecting.

Within the *fallax* subgroup, the distinguishing feature of this species is the relatively short inferior appendages with apices that appear rounded in lateral view. The only evident difference in the illustrations provided for *C.fallax* and that of *C.lukawei* is the more prominent dorsal spine-like projections of tergum X in *C.fallax*. The difference is minor, and we do not consider it to be of species-level significance.

Within the group of taxa assigned to the *fallax* subgroup with a spine-like modification to the lateral lobes of tergum X, it is the only species in which the ventral apex of the phallobase is weakly or only moderately projecting; the periphallic processes are also much less developed than in the other species. Thus, it probably represents a basal species of this clade.

###### Redescription.

***Adult.*** Overall color (in alcohol) yellowish brown, appendages slightly paler. Head relatively short (postocular parietal sclerite < 1/2 diameter of eye). Palps relatively short; maxillary palp with 1^st^ segment very short (approximately as long as wide), 2^nd^ segment moderately elongate, apex with small cluster of stiff setae, 3^rd^ segment subequal to segment 2, 4^th^ segment short (~ ½ length of segment 2), 5^th^ segment subequal to 2^nd^ or 3^rd^. Forewing length: male, 5.0–6.0 mm; female 5.5–6.5 mm. Fore- and hind wings with forks I, II, III, and V present. Forewing with R_1_ very sinuous, stem of Rs inflected at past midlength (with distinct small node at inflection), basal fork of discoidal cell distinctly enlarged, fork asymmetric, length of cell ~ 2× width, forks I and II both subsessile, *r* crossvein diagonal, intersecting discoidal cell at past midlength, *s* and *r-m*, crossveins linear, *m* crossvein more proximal, *s* pigmented (like wing), *r-m* and *m* crossveins hyaline, 2A with crossvein (apparently forked apically to 1A and 3A). Hind wing with R_1_ reduced, but evident, narrowly parallel to subcosta, forks I and II strongly subsessile, fork III distal and relatively wide, anal loop small. Forelegs with apical tibial spur short; male with tarsal claws unmodified, apical segments of tarsi narrow, claws small and symmetrical.

***Male genitalia*.** Segment VIII with sternum relatively short, ventrally with distinct projection from posterior margin, tergum slightly longer, expanded dorsally. Segment IX, in lateral view, with anteroventral margin weakly produced, anterior margin with angular inflection in ventral ¼, weakly concave and narrowing dorsally; tergum short dorsolaterally, with short, rounded apodemes, obsolete mesally between apodemes; posterior margin nearly linear; ventral margin sloping, more or less linear, with inferior appendages mounted high on segment (nearly midlaterally), basally with elongate, narrow ventral process near base, apex of process acute as viewed laterally, rounded as viewed ventrally, apicoventral surface of ventral process roughened and file-like; anterior margin of sternum subtruncate as viewed dorsally or ventrally. Lateral lobes of tergum X formed into short, narrow, sclerotized, dorsolaterally curved spine-like processes; dorsum of tergum X relatively short, membranous; tergum ventrally with relatively narrow, projecting, mesally fused, sclerotized, periphallic process, subtending phallic apparatus. Preanal appendages short and knob-like, distinctly flattened, membranous basally, but fused laterally to periphallic process. Inferior appendage relatively short, with pronounced basal inflection, apex dorsally inflected, broadly rounded, cupped (concave on mesal surface); as viewed ventrally, with weakly sclerotized projections near base and before midlength (projections not evident in lateral view); mesal surface without projections or ridges. Phallic apparatus with phallobase tubular, with usual basodorsal expansion, apicoventral margin not, or only weakly, projecting; endotheca with two asymmetrically positioned spines of moderate length, membrane textured with small spines, phallotremal sclerite complex composed of short rod and ring structure, with small preapical sclerite.

###### Distribution.

Cameroon, Democratic Republic of the Congo, Ghana, Madagascar, Sao Tomé.

##### ﻿ Chimarra jacquemarti sp. nov.

Taxon classificationAnimaliaTrichopteraPhilopotamidae

5951AE16-0542-5F24-A753-BDAAE0E11042

http://zoobank.org/3ADE8459-D153-46E2-B49B-33EA58E171EC

Fig. 11A–D

###### Type material.

***Holotype*.** Ghana – **Central Reg.** ● ♂ (in alcohol); Kakum Forest Reserve; 5°21'N, 1°22'W; 8–15 Nov. 1994; T Andersen leg.; Malaise trap; UMSP 000550005.

**Figure 11.**
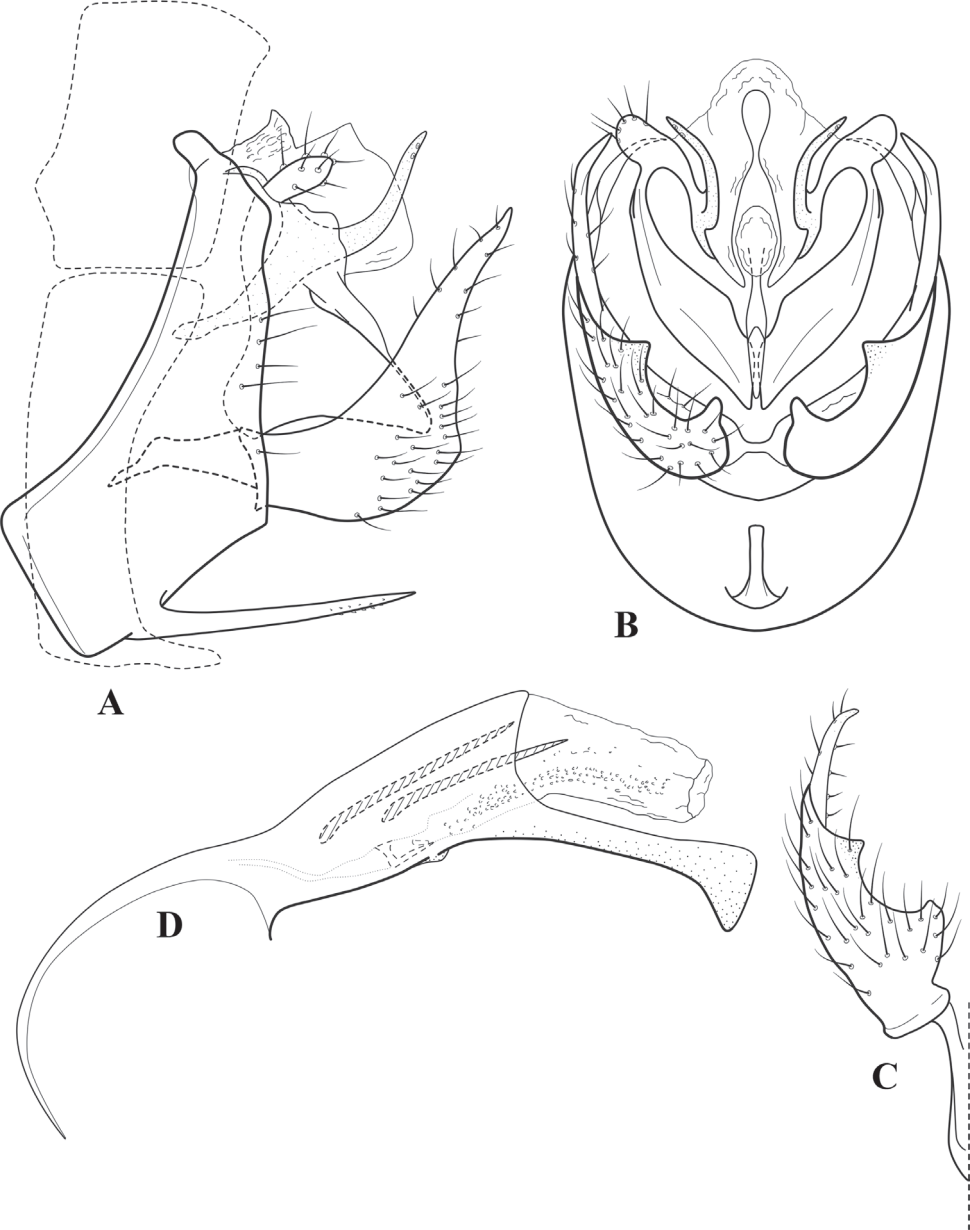
*Chimarrajacquemarti* sp. nov., ♂ genitalia **A** lateral **B** caudal **C** inferior appendage, ventral **D** phallus, lateral.

###### Diagnosis.

Phallobase with ventral apex produced and strongly bent, extreme apex enlarged and bent downward; both phallic spines narrow and elongate; inferior appendage with apex strongly narrowed and only weakly bent, cusps of ventromesal margin not evident in lateral view.

Although similar in overall morphology to other species in the *fallax* subgroup with acute dorsal processes on tergum X, it is the only species in which the ventral projection of the phallobase has its apex truncate, extending straight on its dorsal margin, but distinctly hooked downward on its ventral margin. This species is most similar to *C.dybowskina*. Both species are characterized by an elongate and strongly bent ventral projection of the phallobase, which is slightly expanded and subtruncate apically. However, as noted above, *C.jacquemarti* sp. nov. differs in that the apex of the projection, in lateral view, is strongly compressed and bent down, with the dorsal margin projecting straight, whereas *C.dybowskina* has the apex truncate or subtruncate, with the dorsal margin bent upward. Although the phallic spines are relatively elongate in both species, those in *C.jacquemarti* seem to be narrower and slightly more elongate. The general shape of the inferior appendages is also somewhat different.

###### Description.

***Adult.*** Color of head, prothorax, and appendages (in alcohol) yellowish, body and spurs yellowish brown. Head relatively short (postocular parietal sclerite < 1/2 diameter of eye). Palps relatively short; maxillary palp with 1^st^ segment very short (approximately as long as wide), 2^nd^ segment moderate in length (distinctly shorter than segment 3), apex with small cluster of stiff setae, 3^rd^ segment moderately elongate, 4^th^ segment very short (~ ½ length of segment 2), 5^th^ segment subequal to segment 3. Forewing length: male, 5.0 mm. Fore- and hind wings with forks I, II, III, and V present. Forewing with R_1_ very sinuous, stem of Rs inflected at past midlength (with distinct small node at inflection), basal fork of discoidal cell distinctly enlarged, fork asymmetric, length of cell ~ 2× width, fork I somewhat subsessile, fork II approximately sessile, *r* crossvein diagonal, intersecting discoidal cell at past midlength, just before fork I, *s* and *r-m*, crossveins linear, *m* crossvein more proximal, *s* pigmented (like wing), *r-m* and *m* crossveins hyaline, 2A with crossvein (apparently forked apically to 1A and 3A). Hind wing with R_1_ reduced, but evident, narrowly parallel to subcosta, forks I and II subsessile, fork III distal and relatively wide, anal loop moderate in size. Forelegs with apical tibial spur prominent; male with tarsal claws unmodified, apical segments of tarsi narrow, claws small and symmetrical.

***Male genitalia*.** Segment VIII with sternum relatively short, ventrally with distinct projection from posterior margin, tergum longer (~ 2× length of sternum at base). Segment IX, in lateral view, with anteroventral margin moderately produced, anterior margin with angular inflection at approximately ventral ¼, slightly concave and narrowing dorsally; tergum short dorsolaterally, with distinct apodemes, obsolete mesally between apodemes; posterior margin nearly linear; ventral margin sloping, more or less linear, with elongate, narrow ventral process near base, apex of process acute as viewed laterally, rounded as viewed ventrally, apicoventral surface of ventral process roughened and file-like; anterior margin of sternum subtruncate as viewed dorsally or ventrally, slightly concave mesally. Lateral lobes of tergum X formed into dorsally curved, sclerotized, spine-like processes, bearing two preapical sensilla; dorsum of tergum X relatively short, membranous; tergum ventrally with strongly projecting, paired, sclerotized, periphallic processes, subtending phallic apparatus. Preanal appendages short and knob-like, distinctly flattened, membranous basally, but fused laterally to periphallic processes. Inferior appendage with pronounced basal inflection, apex dorsally inflected and strongly narrowing, slightly posteriorly curved, apex acute; as viewed ventrally, with weakly sclerotized projection near base and more strongly sclerotized projection before midlength, projections not or scarcely evident in lateral view; mesal surface without projections or ridges. Phallic apparatus with phallobase tubular, with usual basodorsal expansion, somewhat flared apically, apicoventral margin very strongly projecting, sclerotized, and ventrally deflected, apex of ventral projection, as viewed laterally, expanded, dorsal margin extending almost straight, apex truncate, ventral margin distinctly downturned and acute, apex strongly compressed and flattened as viewed ventrally or caudally; endotheca with two elongate, narrow, and asymmetrically positioned spines, membrane textured with small spines, phallotremal sclerite complex composed of short rod and ring structure.

###### Etymology.

*Chimarrajacquemarti*, name used as a genitive, for S. Jacquemart, in recognition of his substantial contributions to the description of African caddisflies, including a number of species of *Chimarra*.

##### ﻿ Chimarra lanceolata sp. nov.

Taxon classificationAnimaliaTrichopteraPhilopotamidae

C3CBFB5F-43BC-5177-8A35-B30927036F7F

http://zoobank.org/8FA0E7BC-98AE-4F16-98E4-57550C3CEA6E

Fig. 12A–E

###### Type material.

***Holotype*.** Ghana – **Volta Reg.** ● ♂ (in alcohol); Wli, Agumatsa waterfall, station # 3; 7°07'29"N, 0°35'31"E; 17 Nov. 1993, J Kjærandsen leg.; light trap; UMSP 000550006. ***Paratypes*.** Ghana – **Volta Reg.** ● 2♂♂; same data as for holotype; ZMBN. – **Central Reg.** ● 1♂; Kakum Forest Reserve; 5°21'N, 1°22'W; 8–15 Nov. 1994; T. Andersen leg.; Malaise trap; ZMBN.

**Figure 12.**
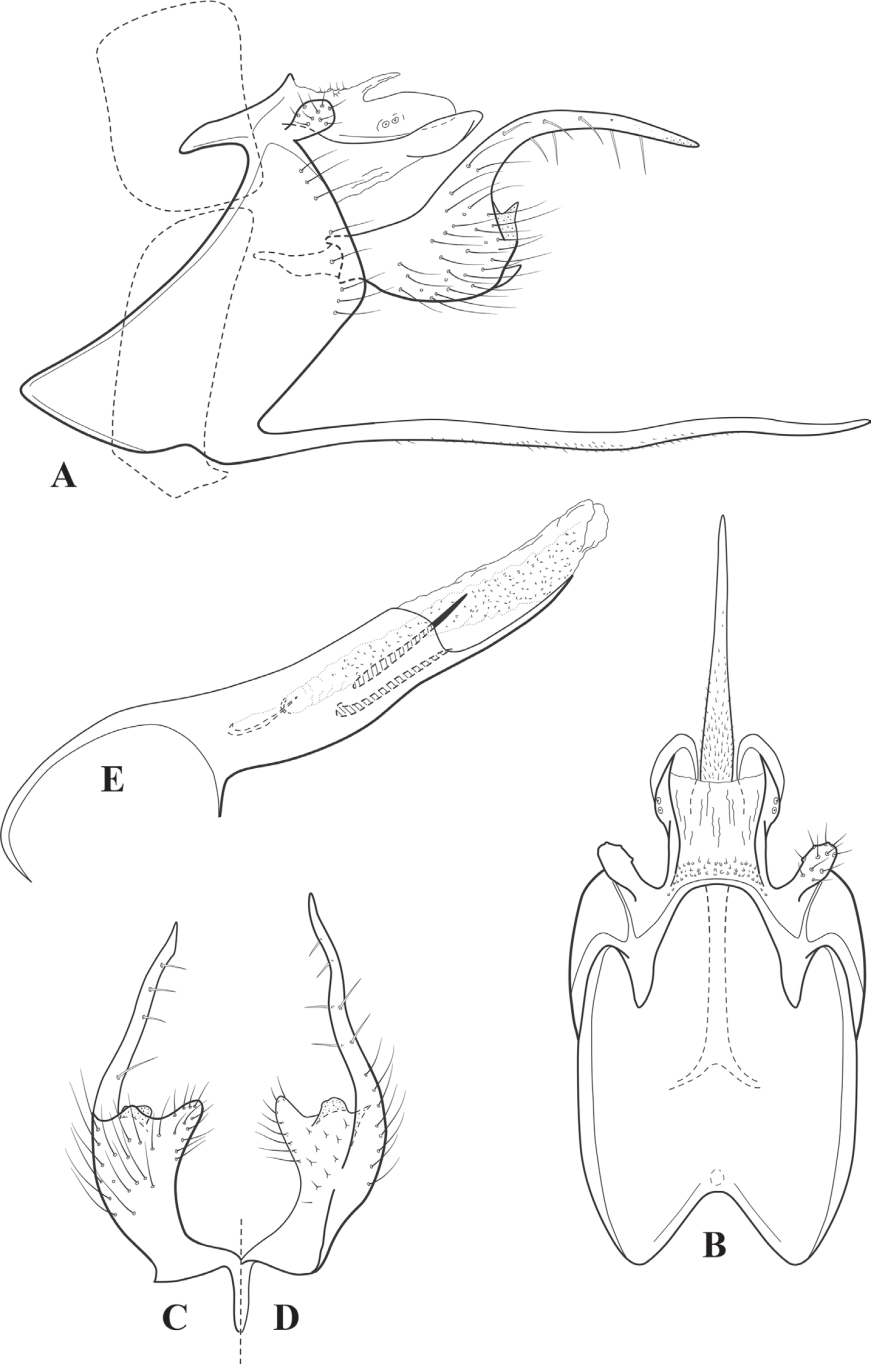
*Chimarralanceolata* sp. nov., ♂ genitalia **A** lateral **B** dorsal, segments IX and X **C** inferior appendage, ventral **D** inferior appendage, dorsal **E** phallus, lateral.

###### Additional material.

Ghana – **Central Reg.** ● 2♀♀; Kakum Forest Reserve; 5°21'N 1°22'W; 8–15 Nov. 1994; T. Andersen leg.; Malaise trap, ZMBN ● 1♀; same collection data as for preceding; UMSP. – **Volta Reg.** ● 1♀; Wli, Agumatsa waterfall, station # 5^C^; 7°07'29"N, 0°35'31"E; 12–15 Mar. 1993, JS Amakye & J Kjærandsen leg.; Malaise trap; ZMBN ● 1♀; same collection data as for preceding except station # 8^B^; 4–7 Mar. 1993; ZMBN ● 1♀; same collection data as for preceding except station # 3; 10 Mar. 1993; light trap; ZMBN ● 4♀♀; same collection data as for preceding except 18 Nov. 1993; J Kjærandsen leg.; ZMBN. – **Western Reg.** ● 1♀; Ankasa Game Production Reserve; 5°15'N 2°37'W; 6–12 Dec. 1993; T Andersen & J Kjærandsen leg.; Malaise trap; ZMBN.

###### Diagnosis.

Ventral process of segment IX incredibly elongate; tergum X without spine-like dorsal projection, ventral part completely divided laterally, but not strongly deflexed; inferior appendage mounted at approximately midheight on segment, far above ventral process, dorsal apex strongly narrowed, acute apically, and also very strongly posteriorly bent, ventral cusps visible in lateral view; phallobase with ventral apex extended and sclerotized, but not bent or excessively produced; phallic spines both moderately elongate and narrow; sternum VIII with posteroventral margin weakly produced.

*Chimarralanceolata* is most readily identified by the very elongate ventral process of segment IX; the character is so unusual that it would almost appear to be an aberrant or mutation, but there is no evidence of this. Other characters, especially the overall shape of the inferior appendages, are also distinctive for this species and thus it is unlikely to be confused with any other species.

###### Description.

***Adult.*** Overall color (in alcohol) yellowish brown, appendages paler, tibial spurs slightly darker. Head relatively short (postocular parietal sclerite ~ 1/2 length of eye). Palps moderately elongate; maxillary palp with 1^st^ segment very short (approximately as long as wide), 2^nd^ segment short (~ 2× 1^st^), apex with small cluster of stiff setae, 3^rd^ segment elongate, almost 2× 2^nd^, 4^th^ segment short (shorter than 2^nd^), 5^th^ relatively elongate (longer than 3^rd)^. Forewing length: male, 6.0–6.5 mm; female, 5.5–6.5 mm. Fore- and hind wings with forks I, II, III, and V present. Forewing with R_1_ somewhat sinuous, stem of Rs weakly inflected at past midlength, basal fork of discoidal cell distinctly enlarged, fork slightly asymmetric, length of cell > 2× width, fork I subsessile, fork II sessile, *r* crossvein diagonal, intersecting discoidal cell at past midlength, just before fork I, *s* and *r-m*, crossveins linear, *m* crossvein more proximal, *s* pigmented (like wing), *r-m* and *m* crossveins hyaline, 2A with crossvein (apparently forked apically to 1A and 3A). Hind wing with R_1_ narrowly parallel to subcosta, forks I and II subsessile, anal loop moderate. Forelegs with apical tibial spur distinct; male with foretarsi unmodified, claws small and symmetrical.

***Male genitalia*.** Segment VIII with sternum relatively short ventrally, with very short, posteriorly-projecting ventromesal projection, tergum slightly longer than sternum. Segment IX, in lateral view, with anteroventral margin distinctly produced, anterior margin with very angular inflection in ventral ¼, almost linearly narrowing dorsally; tergum very short dorsolaterally, with prominent apodemes, obsolete mesally between apodemes; posterior margin with obtuse angular projection at middle, at insertion of inferior appendages, ventral margin with extremely elongate, narrow, posteriorly-projecting, apically acute, ventral projection; anteroventral margin, as viewed dorsally or ventrally, with very distinct mesal invagination. Lateral lobes of tergum X relatively short, each divided midlaterally into short rounded dorsal lobe, with two sensilla at midlength, and somewhat longer ventral lobe; mesal lobe of tergum X short, membranous. Preanal appendages short and knob-like, constricted basally. Inferior appendage with basal inflection and dorsal process; ventral margin with apex acute, mesally curved, not strongly sclerotized; mesal margin below dorsal process with very strongly sclerotized cusp, at least partially visible in lateral view; dorsal projection elongated, narrow, apically acute, very strongly posteriorly bent. Phallic apparatus with phallobase tubular, with usual basodorsal expansion, apicoventral margin with acute, narrow, sclerotized projection, extending almost straight (not ventrally deflected). Endotheca with two asymmetrically positioned spines of moderate length, membrane textured with small spines, phallotremal sclerite complex composed of short rod and ring structure with short apical sclerite.

###### Etymology.

*Chimarralanceolata*, used as an adjective, from the Latin *lanceolatus*, meaning spear-like, and referring to lance-like ventral process of segment IX, reminiscent of the elongate lance used in medieval jousting tournaments.

##### ﻿ Chimarra robynsi

Taxon classificationAnimaliaTrichopteraPhilopotamidae

(Jacquemart, 1967)

9BE54006-E461-5112-84A1-061D310E0B81

Fig. 13A–E

Chimarrafra [sic] robynsi Jacquemart, 1967 (1966a): 49–51, fig. 15.
Chimarra robynsi (Jacquemart, 1967). Comb. nov.

###### Material examined.

Tanzania – **Tanga Reg.** ● 1♂; West Usambara Mt., Mazumbai, Kaputu Stream; 4°48'S, 38°30'E; 17–20 Nov. 1990; T Andersen leg.; Malaise trap; UMSP.

###### Diagnosis.

Phallobase with ventral apex greatly produced, but only weakly bent, apex slightly enlarged and more or less rounded apically; phallic spines both relatively short; inferior appendage relatively narrow overall, with dorsal projection narrow and tapering, not or only scarcely bent, cusps of ventromesal surface not evident in lateral view.

**Figure 13.**
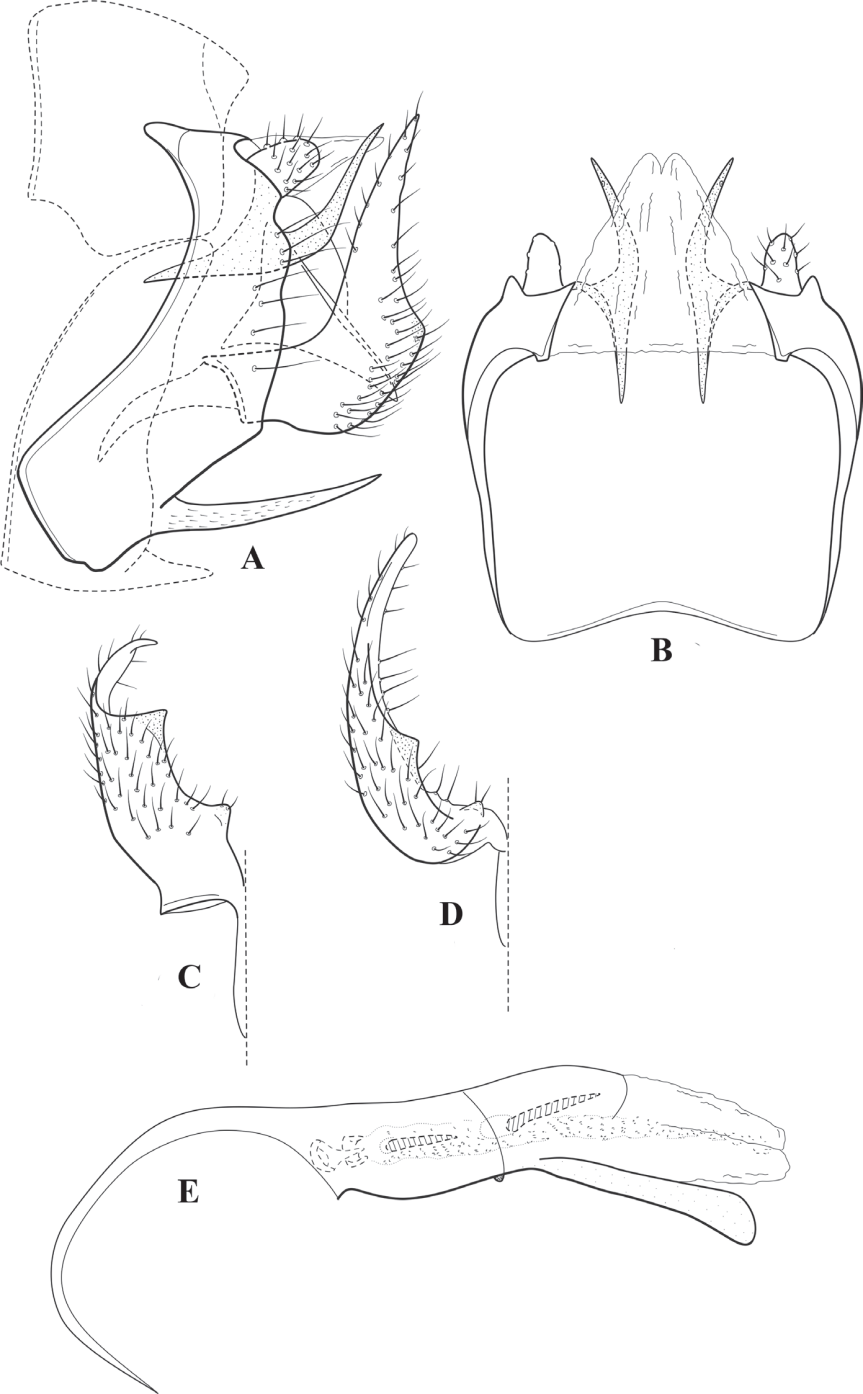
*Chimarrarobynsi* (Jacquemart), ♂ genitalia **A** lateral **B** dorsal, segments IX and X **C** inferior appendage, ventral **D** inferior appendage, caudal **E** phallus, lateral.

We are somewhat unsure of our attribution of the specimen illustrated (Fig. 13A–E) here to *C.robynsi*, especially considering their different provenance. However, among the species of the *fallax* complex, it has the most slender inferior appendage, with a very narrow dorsal process that is not, or scarcely, bent apically. The slightly narrower dorsal process of the inferior appendage in the illustration by Jacquemart can probably be attributed to a slight difference in the orientation of the specimen when illustrated, as suggested by slightly rotating the specimen. Among the species of the *fallax* subgroup with an elongate ventral apex to the phallobase, considered here, *C.robynsi* has the apex least ventrally flexed or bent, possibly similar in this respect to *C.falcifera*, which was not available for comparison. As noted in the description of *C.calundoensis*, the hind wing of *C.falcifera* was illustrated as lacking fork III (Jacquemart 1966b: fig. 7C) which is not true of the specimen illustrated here, in which the fork is prominent, as in Fig. 4B.

###### Redescription.

***Adult.*** Head, prothorax, and appendages (in alcohol) yellowish; mesothorax and body yellowish brown, spurs slightly darker. Head relatively short (postocular parietal sclerite ~ 1/2 diameter of eye). Palps moderately elongate; maxillary palp with 1^st^ segment slightly longer than wide, 2^nd^ segment moderately elongate (~ 3× 1^st^), apex with small cluster of stiff setae, 3^rd^ segment only slightly longer than 2^nd^, 4^th^ segment short (~ 1/2 length of 3^rd^), 5^th^ segment subequal to 3^rd^. Forewing length: male, 7.1 mm. Fore- and hind wings with forks I, II, III, and V present. Forewing with stem of Rs rather weakly inflected at past midlength, basal fork of discoidal cell distinctly enlarged, length of cell ~ 2× width, fork I slightly subsessile, fork II sessile, *r* crossvein diagonal, intersecting discoidal cell at past midlength, just before fork I, *s* and *r-m*, crossveins linear, *m* crossvein more proximal, *s* pigmented (like wing), *r-m* and *m* crossveins hyaline, 2A with crossvein (apparently forked apically to 1A and 3A). Hind wing with R_1_ narrowly parallel to subcosta, forks I and II subsessile, anal loop small. Forelegs with apical tibial spur distinct; male with foretarsi unmodified, claws small and symmetrical.

***Male genitalia*.** Segment VIII with sternum relatively short, ventrally with distinct projection from posterior margin, tergum slightly longer. Segment IX, in lateral view, with anteroventral margin moderately produced, anterior margin with angular inflection in ventral ¼, weakly concave and narrowing dorsally; tergum short dorsally, with short anterior apodemes, obsolete mesally between apodemes; posterior margin nearly linear; ventral margin sloping, more or less linear, with elongate, narrow ventral process, apex of process acute as viewed laterally, rounded as viewed ventrally, apicoventral surface of ventral process roughened and file-like; anterior margin of sternum, as viewed dorsally or ventrally, subtruncate, slightly concave mesally. Lateral lobes of tergum X formed into dorsally curved, sclerotized, spine-like processes, with one or two sensilla apically; mesal lobe of tergum X membranous, moderately elongate; ventrally with strongly projecting, paired, sclerotized, periphallic processes, subtending phallic apparatus. Preanal appendages short and knob-like, constricted basally, fused laterally to periphallic processes. Inferior appendage relatively slender and narrow, with pronounced basal inflection, apex dorsally inflected and strongly narrowing, nearly straight, apex acute; as viewed ventrally, with weakly sclerotized, angular projections or cusps near base and before midlength, projections not evident in lateral view; mesal surface without projections or ridges. Phallic apparatus with phallobase tubular, with usual basodorsal expansion, apicoventral margin very strongly projecting, sclerotized, weakly ventrally deflected, apex of ventral projection more or less rounded, as viewed laterally; endotheca with two relatively short and asymmetrically positioned spines, membrane textured with small spines, phallotremal sclerite complex composed of short rod and ring structure and small apical sclerite.

###### Distribution.

Democratic Republic of the Congo, Tanzania.

##### ﻿ Chimarra togoana

Taxon classificationAnimaliaTrichopteraPhilopotamidae

(Ulmer, 1907)

9E0D8C53-A654-5889-B555-D789B4633D05

Fig. 14A–E

Wormaldia togoana Ulmer, 1907: 42–43, figs 61–63.
Chimarrha togoana (Ulmer): Ulmer 1931: 3.
Chimarra togoana (Ulmer): Fischer 1961: 71; Gibbs 1973: 67 (distribution: Ghana).

###### Material examined.

Ghana – **Volta Reg.** ● 1♂ 2♀♀; Wli, Agumatsa waterfall, station # 3; 7°07'29"N, 0°35'31"E; 17 Nov. 1993; J Kjærandsen leg.; light trap; ZMBN ● 1♂; same collection data as for preceding; UMSP ● 3♀♀; same collection data as for preceding except station # 10; 11 Nov. 1993; ZMBN ● 1♀; same collection data as for preceding except 20 Nov. 1993; J Kjærandsen leg.; UMSP.

**Figure 14.**
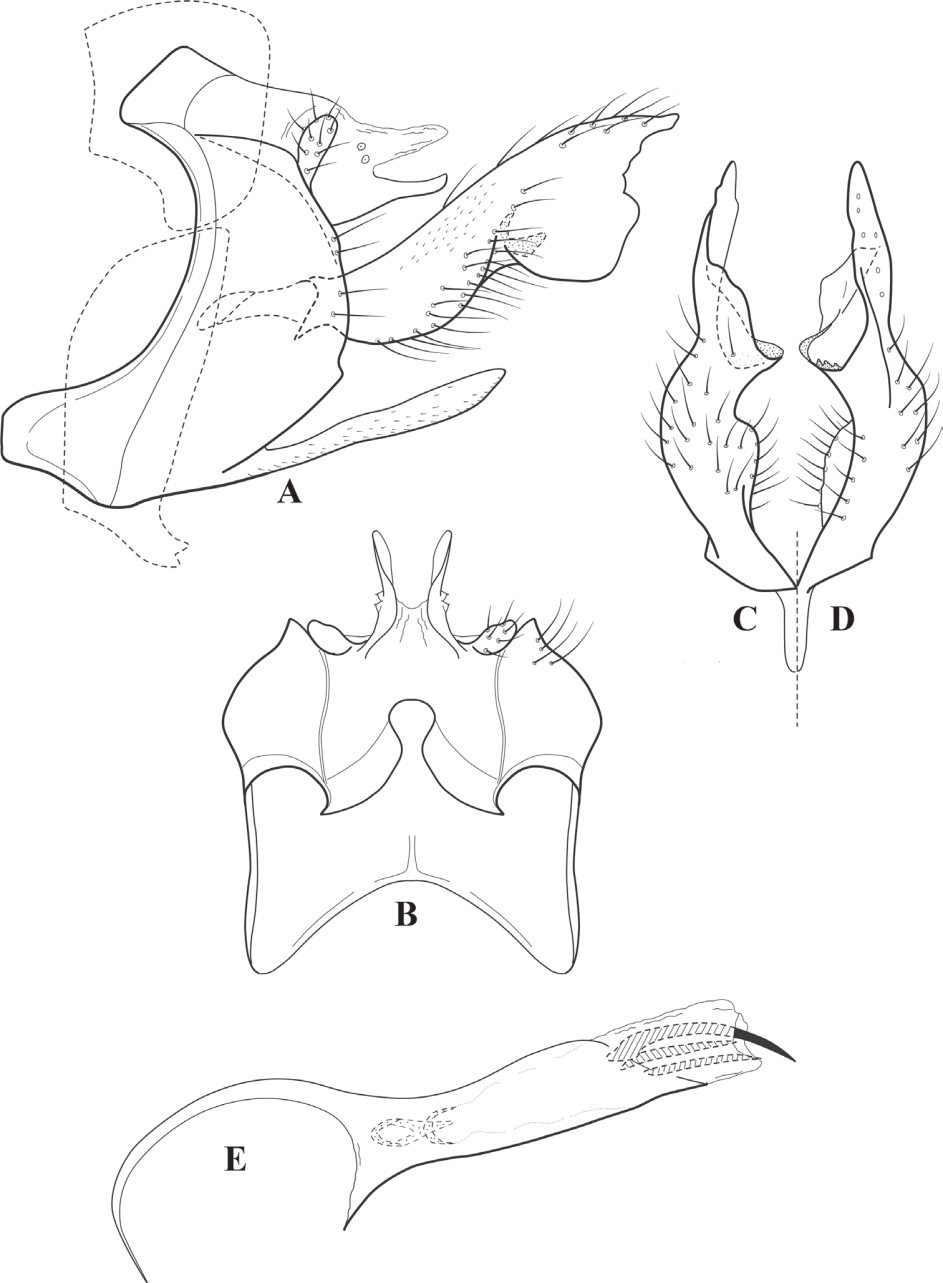
*Chimarratogoana* (Ulmer), ♂ genitalia **A** lateral **B** dorsal, segments IX and X **C** inferior appendage, ventral **D** inferior appendage, dorsal **E** phallus, lateral.

###### Diagnosis.

*Chimarratogoana* is a very distinctive species, readily identified by the elongate, apically flared shape of its inferior appendages, with a distinctive mesal cusp at approximately midlength, and the elongate ventral process of segment IX, which is somewhat inflated apically, but apparently lacks the cluster of apicoventral spines characteristic of species in the *ruficeps* subgroup. It is only provisionally placed in the *fallax* subgroup since some of its characters could equally well be used to place it in the *ruficeps* subgroup. Characters supporting the latter interpretation include the overall shape of segment IX, which is strongly produced anteroventrally and has its ventromesal margin concave, and the distinctly formed and enlarged dorsolateral apodemes of the same segment. Characters supporting its placement in the *fallax* subgroup include the posteroventral projection of segment VIII. It is also possible that it belongs to a lineage basal to both of those subgroups. The rather simple tergum X, with an apicolateral cleft on each of its lateral lobes, is probably a primitive character; it may be ancestral to both subgroups, if the periphallic processes of the *fallax* subgroup had their origin as a cleft in each of the lateral lobes of tergum X.

###### Redescription.

***Adult.*** Overall color (in alcohol) nearly uniformly yellowish brown. Head relatively short (postocular parietal sclerite ~ 1/2 diameter of eye). Palps relatively elongate; maxillary palp with 1^st^ segment very short (approximately as long as wide), 2^nd^ segment short (~ 3× 1^st^), apex with small cluster of stiff setae, 3^rd^ segment relatively elongate (nearly 2× 2^nd^), 4^th^ segment short (slightly shorter than 2^nd^), 5^th^ segment elongate (nearly as long as 3^rd^ and 4^th^ combined). Forewing length: male, 6.2–7.0 mm; female, 6.5–7.5 mm. Fore- and hind wings with forks I, II, III, and V present. Forewing with R_1_ somewhat sinuous, stem of Rs weakly inflected at past midlength, without node at inflection, basal fork of discoidal cell not enlarged, fork nearly symmetric, length of cell ~ 2× width, fork I slightly subsessile, fork II sessile, *r* crossvein diagonal, intersecting discoidal cell at past midlength, just before fork I, *s* and *r-m* crossveins linear, *m* crossvein very distinctly more proximal, *s* pigmented (like wing), *r-m* and *m* crossveins hyaline. 2A with crossvein (apparently forked apically to 1A and 3A). Hind wing with R_1_ narrowly parallel to subcosta, forks I and II strongly subsessile, fork III distal and relatively wide, anal loop small. Foreleg with apical tibial spur distinct; male with foretarsi unmodified, claws small and symmetrical.

***Male genitalia*.** Segment VIII relatively short, sternum with short, posteriorly projecting, ventromesal projection, tergum slightly longer than sternum. Segment IX, in lateral view, with anterior margin distinctly produced and rounded in ventral ¼, dorsolaterally with prominent rounded apodemes, margin strongly convex between apodemes; tergum continuous dorsally, forming deep, narrow emargination mesally between apodemes; posterior margin broadly convex; posteroventral margin with elongate, narrow, posteriorly-projecting, ventral process, apex of process slightly expanded. Segment IX, in dorsal or ventral views, with anteroventral margin strongly concave. Lateral lobes of tergum X short, each partially divided from posterior margin into dorsal and ventral lobes, dorsal lobe with two sensilla in basal half; mesal lobe of tergum X very short, membranous. Preanal appendages short and rounded, somewhat flattened, constricted basally. Inferior appendage, in lateral view, elongate, projecting, widened and flared apically, distal margin subtruncate; appendage with prominent, sclerotized mesal cusp at approximately midlength, visible in lateral view as notch on ventral margin. Phallic apparatus with phallobase tubular, with usual basodorsal expansion, apicoventral margin only weakly projecting, endotheca with three spines, one relatively elongate, curved, and strongly sclerotized, other two relatively short, asymmetrically positioned; phallotremal sclerite complex composed of short rod and ring structure with small apical sclerite.

###### Distribution.

Ghana, Togo.

#### The *kenyana* subgroup

**Included species.***Chimarraakana* Gibbs, 1973; *C.ambulans* Barnard, 1934; *C.baculifera* Marlier, 1965; *C.chicapa* Marlier, 1965; *C.flaviseta* Wahlberg, Espeland & Johanson, 2014; *C.intermedia* Jacquemart, 1961; *C.kenyana* Ulmer, 1931; *C.krugeri* Jacquemart, 1963; *C.longistylis* Jacquemart & Statzner, 1981; *C.morogoroensis* sp. nov.; *C.mulanjae* Wahlberg, Espeland & Johanson, 2014; *C.mushuvae* Marlier, 1951; *C.pedaliotus* sp. nov.; *C.psittacus* Wahlberg, Espeland & Johanson, 2014; *C.quaridspinosa* Jacquemart & Statzner, 1981; *C.rhodesi* Kimmins, 1957; *C.saudia* Malicky, 1986; *C.somereni* Marlier, 1951; *C.szunyoghyi* Oláh, 1986; *C.tanzaniensis* sp. nov.; *C.triangularis* Kimmins, 1963; *C.triangularisoccidentalis* Gibon, 1985; *C.trispina* Jacquenart, 1961; *C.uvirana* Marlier, 1951; and *C.zombaensis* Wahlberg, Espeland & Johanson, 2014.

General features of subgroup: tergum X with lateral lobes entire (or sometimes cleft apically), with digitate process near dorsal margin bearing two sensilla, process sometimes short. Inferior appendage with distinct basal inflection and mesal curvature, variable in length, but generally relatively narrow, with variably modified apex; mesal cusps absent. Ventral process of segment IX short, usually posteriorly projecting; the shape of the ventral process and genital capsule is variable among species and often usefully diagnostic. Phallus often with a pair of symmetrically positioned spines.

The number of known species assigned to this subgroup is large. Although there is considerable variation in the shape of the genital capsule, ventral process, phallic armature, etc., most species have a general similarity that readily allows them to be placed in the subgroup. Distinguishing differences between some species are relatively minor. A revision of the subgroup would be a useful contribution.

##### ﻿ Chimarra akana

Taxon classificationAnimaliaTrichopteraPhilopotamidae

Gibbs, 1973

FB7C70F5-2A31-5DE1-B915-A9BA4402A8D7

Fig. 15A–E

Chimarra akana Gibbs, 1973: 366–367, figs 14–16.
Chimarra akana Gibbs: Marlier 1980: 62 (as possible synonym of C.kenyana Ulmer); Gibon 1985: 25, figs 7, 12 (distribution: Ivory Coast).

###### Material examined.

Ghana – **Eastern Reg.** ● 4♂♂3♀♀; Kibi, Subri stream; 6°10'N, 0°33'W; 5 Nov. 1993; J Kjærandsen leg.; light trap; ZMBN. – **Volta Reg.** ● 1♂; Kute, River Menu; 7°22'N, 0°36'E; 11 Dec. 1990; JS Amakye leg.; light trap; ZMBN ● 1♂; Wli, Agumatsa waterfall, station # 2^A^; 7°07'29"N, 0°35'31"E; 8–11 Mar. 1993; JS Amakye & J Kjærandsen leg.; Malaise trap; ZMBN ● 15♂♂11♀♀; same collection data as for preceding except station # 3; 17 Nov. 1993; J Kjærandsen leg.; light trap; ZMBN ● 1♂; same collection data as for preceding except station # 6; 11 Mar. 1993; JS Amakye & J Kjærandsen leg.; ZMBN ● 1♂; same collection data as for preceding except station # 10; 19 Nov. 1993; J Kjærandsen leg.; UMSP ● 10♂♂2♀♀; same collection data as for preceding except station # 15; 5 Dec. 1993; ZMBN ● 1♀; same collection data as for preceding except station # 19; 9 Dec. 1993; ZMBN. – **Western Reg.** ● 1♂1♀; Ankasa Game Production Reserve; 5°15'N, 2°37'W; 6–12 Dec. 1993; T Andersen & J Kjærandsen leg.; Malaise trap; ZMBN ● 1♂; same collection data as for preceding except 11 Mar. 1993; J Kjærandsen leg.; light trap; ZMBN ● 10♂♂3♀♀; same collection data as for preceding except 5–9 Dec. 1993; T Andersen & J Kjærandsen leg.; ZMBN ● 1♀; same collection data as for preceding except 19 Dec. 1993; UMSP.

###### Diagnosis.

Characters, in combination, that confirm the identification and can be used to distinguish *C.akana* from other species in the subgroup include: the general shape and length of tergum X and position and length of digitate dorsal process; overall shape and length of inferior appendage and shape of apex in lateral view (orientation of appendage is slightly more bowed outward in specimen from Ghana); general shape of segment IX and length and shape of ventral process; details of phallus, especially the pair of curved ventral spines and upturned dorsal apex of phallobase.

**Figure 15.**
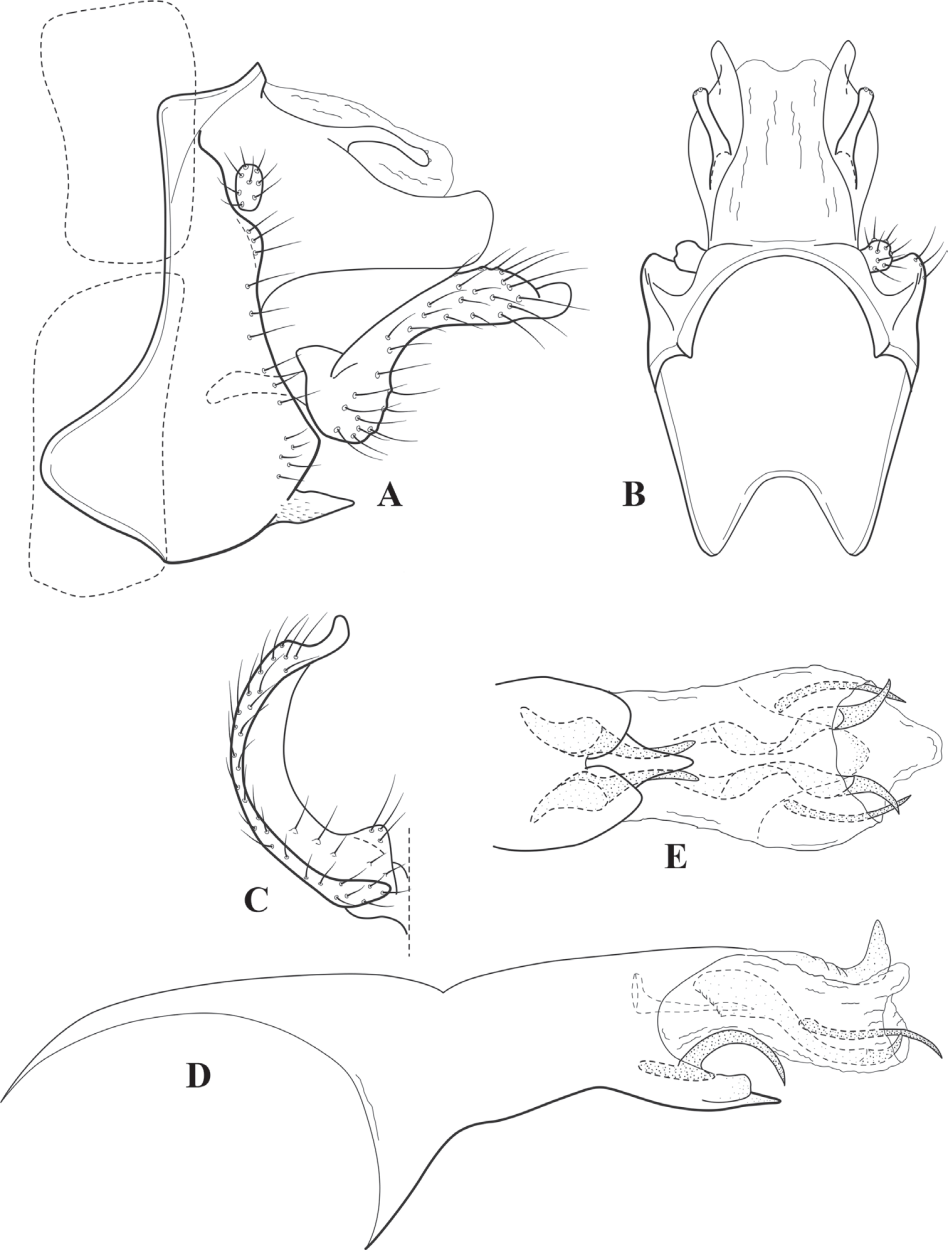
*Chimarraakana* Gibbs, ♂ genitalia **A** lateral **B** dorsal, segments IX and X **C** inferior appendage, caudal **D** phallus, lateral **E** phallus apex, ventral.

The form illustrated here (Fig. 15A–E) closely matches the illustration provided by Gibon (1985: figs 7–8). As compared to the illustration of the species provided by Gibbs (1973: fig.16), the apex of the inferior appendage appears to be more sinuate. This is probably a matter of the orientation of the structure when illustrated (more or less caudal in the specimen illustrated by Gibon (1985: fig. 8), and more ventral in the specimen illustrated by Gibbs (1973: fig.16). For now, we accept Gibon’s illustration as representing this species. Marlier (1980), who synonymized *C.wittei* Jacquemart with *C.kenyana* Ulmer, also suggested that *C.akana* may be synonym of this species. Both of these species would have name priority over *C.akana*. Unfortunately, the illustrations provided for the species do not provide enough details to make an informed conclusion. The issue, including the synonymy made by Marlier, should be addressed in a future revision of the subgroup.

Diagnostic features of *C.akana* include, in particular, the shape of the inferior appendages which are relatively narrow and strongly bowed, with the apex somewhat narrowed and upturned, as viewed caudally, with the relatively elongate dorsal sensilla-bearing lobes of tergum X and the phallic armature, which includes a pair of ventrally curved spines near the base of the endotheca and also a pair of very narrow spines apically, in addition to the phallotremal sclerite complex. The dorsal margin of the phallobase is also somewhat produced and upturned apically, but only weakly sclerotized, so the feature may not always be evident. As in all species of the *kenyana* subgroup, the shape of the genital capsule and ventral process of segment IX are also important considerations in making species determinations, even if these features are not absolutely consistent. Compared to other species in the subgroup, *C.akana* has a relatively short segment IX, with the anteroventral margin produced and a narrow, posteriorly projecting ventral process.

###### Redescription.

***Adult.*** Overall color (in alcohol) nearly uniformly yellowish brown. Head relatively short (postocular parietal sclerite < 1/2 diameter of eye). Palps moderately elongate; maxillary palp with 1^st^ segment very short (slightly longer than wide), 2^nd^ segment short (~ 2× 1^st^), apex with small cluster of stiff setae, 3^rd^ segment elongate (~ 1½ × 2^nd^), 4^th^ segment short (slightly shorter than 2^nd^), 5^th^ segment elongate (subequal to 3^rd^). Forewing length: male, 4.5–5.2 mm; female, 4.8–5.8 mm. Fore- and hind wings with forks I, II, III, and V present. Forewing with R_1_ somewhat sinuous, stem of Rs inflected at approximately midlength, with distinct node at inflection, extending into cell below, basal fork of discoidal cell enlarged, fork asymmetric, length of cell ~ 2× width, forks I and II slightly subsessile, *r* crossvein diagonal, intersecting discoidal cell at past midlength, just before fork I, *m* crossvein proximal to *s* and *r-m* crossveins, *s* pigmented (like wing), *r-m* and *m* crossveins hyaline, 2A with crossvein (apparently forked apically to 1A and 3A). Hind wing with R_1_ narrowly parallel to subcosta, forks I and II subsessile. Foreleg with apical tibial spur short; male with foretarsi modified, claws enlarged, outer claw twisted and asymmetrical.

***Male genitalia*.** Segment VIII relatively short, sternum and tergum subequal in length. Segment IX, in lateral view, relatively short, anterior margin distinctly produced and rounded in ventral ¼, dorsolaterally with broad, weakly developed, apodeme; tergum continuous dorsally, forming concave excavation between lateral apodemes; posterior margin very weakly produced below preanal appendage, widening ventrally to level of inferior appendage; ventral margin rounded between anteroventral production and inferior appendage, ventral process midway between, short, narrow, acute apically. Segment IX, in dorsal or ventral views, with anteroventral margin deeply concave mesally. Lateral lobes of tergum X relatively elongate, subtruncately rounded apically, with moderately elongate, digitate, sensilla-bearing process from dorsal margin in basal half; mesal lobe of tergum X elongate, membranous, somewhat shorter than lateral lobes. Preanal appendages small, rounded, constricted basally. Inferior appendage, in lateral view, relatively elongate, narrow, strongly dorsally flexed near base, apex somewhat narrowed, rounded as viewed laterally, with sinuous dorsal inflection as viewed caudally; appendage, in caudal view, very strongly mesally curved. Phallic apparatus with phallobase tubular, with usual basodorsal expansion, apicoventral margin weakly projecting, dorsal margin somewhat extended, weakly sclerotized, with apex slightly upturned; endotheca with pair of prominent, symmetrical, ventrally curved spines basoventrally, and pair of very narrow, needle-like spines apically; phallotremal sclerite complex composed of moderately elongate rod and ring structure, with relatively elongate, paired apicolateral sclerites, each terminating in a distinct short spine.

###### Distribution.

Ghana, Ivory Coast.

##### ﻿ Chimarra eshowensis sp. nov.

Taxon classificationAnimaliaTrichopteraPhilopotamidae

4524042B-C061-55D6-86A6-1A1EB20AB416

http://zoobank.org/AB398DFA-9761-4F81-8D07-8CDD8D876EB8

Fig. 16A–F

###### Type material.

***Holotype*.** South Africa ● ♂ (pinned); KwaZulu-Natal, Eshowe, Mpushini Falls; 28°54.529'S, 31°26.858'E; 9 Jan. 2000; KM Kjer & RJ Blahnik leg.; UMSP 000172258. ***Paratypes*.** South Africa ● 1♂3♀♀; same data as for holotype; UMSP.

###### Diagnosis.

*Chimarraeshowensis* sp. nov. is very similar to *C.chicapa* Marlier, described from Angola. Both species are distinctive in having relatively short inferior appendages, with their apices little modified and also in having paired, curved, dorsal spines near the base of the endotheca; both were also collected near waterfalls. The differences in the shape of segment IX, forming the genital capsule, length of the dorsal sensilla-bearing processes of tergum X, and particularly the overall shape of the inferior appendages and degree to which their apices are inturned apically, all suggest the two are different species. The assessment is admittedly subjective. The collection of specimens with intermediate character states from intervening areas might warrant a reassessment of their species status.

**Figure 16.**
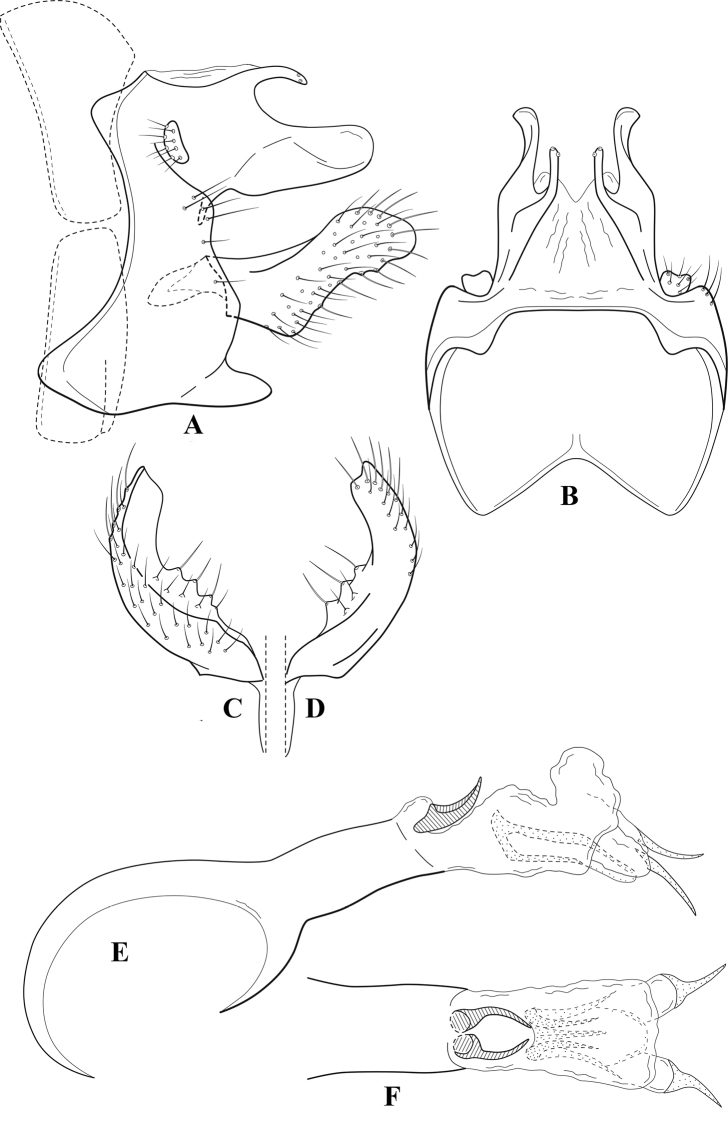
*Chimarraeshowensis* sp. nov., ♂ genitalia **A** lateral **B** dorsal, segments IX and X **C** inferior appendage, ventral **D** inferior appendage, dorsal **E** phallus, lateral **F** phallus apex, dorsal.

###### Description.

***Adult.*** Overall color dark brown, including appendages, femurs paler. Head relatively short and rounded (postocular parietal sclerite ~ 1/2 diameter of eye). Palps relatively short; maxillary palp with 1^st^ segment very short (approximately as long as wide), 2^nd^ segment relatively short (~ 3× 1^st^), apex with small cluster of stiff setae, 3^rd^ segment slightly longer than 2^nd^, 4^th^ segment short (~ 1/2 length of 3^rd^), 5^th^ segment subequal to 2^nd^. Forewing length: male, 4.5–4.8 mm; female, 4.5–5.0 mm. Fore- and hind wings with forks I, II, III, and V present. Forewing with R_1_ somewhat sinuous, stem of Rs weakly inflected at approximately midlength, with distinct node at inflection, extending into cell below, basal fork of discoidal cell enlarged, fork very asymmetric, length of cell ~ 2× width, fork I subsessile, fork II sessile, *r* crossvein diagonal, intersecting discoidal cell at past midlength, just before fork I, *m* crossvein proximal to *s* and *r-m* crossveins, approximately midway between basal fork of M and *r-m* crossvein, *s* pigmented (like wing), *r-m* and *m* crossveins hyaline, *m* very faint, 2A with crossvein (apparently forked apically to 1A and 3A). Hind wing with R_1_ narrowly parallel to subcosta, forks I and II subsessile. Foreleg with apical tibial spur distinct; male with foretarsi modified, claws enlarged, approximately symmetrical.

***Male genitalia*.** Segment VIII relatively short, tergum slightly longer dorsally. Segment IX, in lateral view, relatively short, anterior margin distinctly, subangularly produced in ventral ¼, dorsolaterally with broadly rounded, rather weakly developed, apodeme; tergum continuous dorsally, sclerotized region very short, nearly linear between lateral apodemes; posterior margin very weakly produced below preanal appendage, widening ventrally to level of inferior appendage; ventral margin with rather prominent, posteriorly projecting, ventral process, length greater than width at base, apex subangular. Segment IX, in dorsal or ventral views, with anteroventral margin moderately, angularly, invaginated mesally. Lateral lobes of tergum X, in lateral view, moderately elongate, apices rounded, weakly sclerotized, dorsal margin with moderately elongate, posteriorly curved, digitate, sensilla-bearing process at approximately midlength; mesal lobe of tergum X membranous, extending > 1/2 length of lateral lobes. Preanal appendages small, rounded, and somewhat flattened, constricted basally. Inferior appendage, in lateral view, relatively short, dorsally flexed near base, posteriorly recurved near apex, apex not or very little narrowed, rounded as viewed laterally, subtruncate as viewed dorsally or ventrally; appendage, in dorsal or ventral views, only moderately bowed or curved, apices not more so, basomesally with distinct setae. Phallic apparatus with phallobase relatively short, tubular, with usual basodorsal expansion, apicoventral margin not projecting; endotheca, basodorsally, with pair of prominent, symmetrical, dorsally curved spines; endotheca apically with paired membranous lobes, each terminating in tapering, moderately sclerotized spine; phallotremal sclerite complex composed of moderately elongate rod and ring structure, with elongate, paired, rather weakly sclerotized, dorsal sclerites.

###### Etymology.

*Chimarraeshowensis*, used as an adjective and meaning “from Eshowe” in reference to the place of origin of the holotype specimen.

##### ﻿ Chimarra krugeri

Taxon classificationAnimaliaTrichopteraPhilopotamidae

Jacquemart, 1963

EC811FDD-0F5A-50BA-875C-D523D37C92D3

Fig. 17A–E

Chimarra krugeri Jacquemart, 1963: 395–397, figs 48, 49.
Chimarra krugeri Jacquemart: Scott 1974: 244–245, figs 22–24.

###### Material examined.

Tanzania – **Morogoro Reg.** ● 1♂; Uluguru Mts, Kimboza Forest Reserve, Ruvu River; 7°2'S, 37°47'E; 20 Oct. 1990, T Andersen leg.; sweep net; UMSP.

###### Diagnosis.

The most diagnostic aspects of *Chimarrakrugeri*, in combination, include the very elongate lateral lobes of tergum X, with apices rounded and dorsal margin more strongly sclerotized, and with a very elongate, digitate, sensilla-bearing process basally; the single, very elongate phallic spine; the shape of segment IX, especially the subtriangular ventral process and prominent anterodorsal apodeme; and the general shape of the inferior appendages, whose apices are somewhat broadened or enlarged, as viewed laterally. The latter character will distinguish it from *C.waensis*, *C.baculifera*, and *C.camerunensis*, all of which also have a basally broad, subtriangular, ventral process on segment IX and elongate digitate processes on tergum X but have the inferior appendages more or less uniformly narrow. Among the species of the *kenyana* subgroup treated here, *C.krugeri* is unusual in having a very short discoidal cell in both the fore- and hind wings, with very elongate forks I and II. It is most similar, in this respect, to *C.waensis*, in which the characters are similar, but not quite as exaggerated.

**Figure 17.**
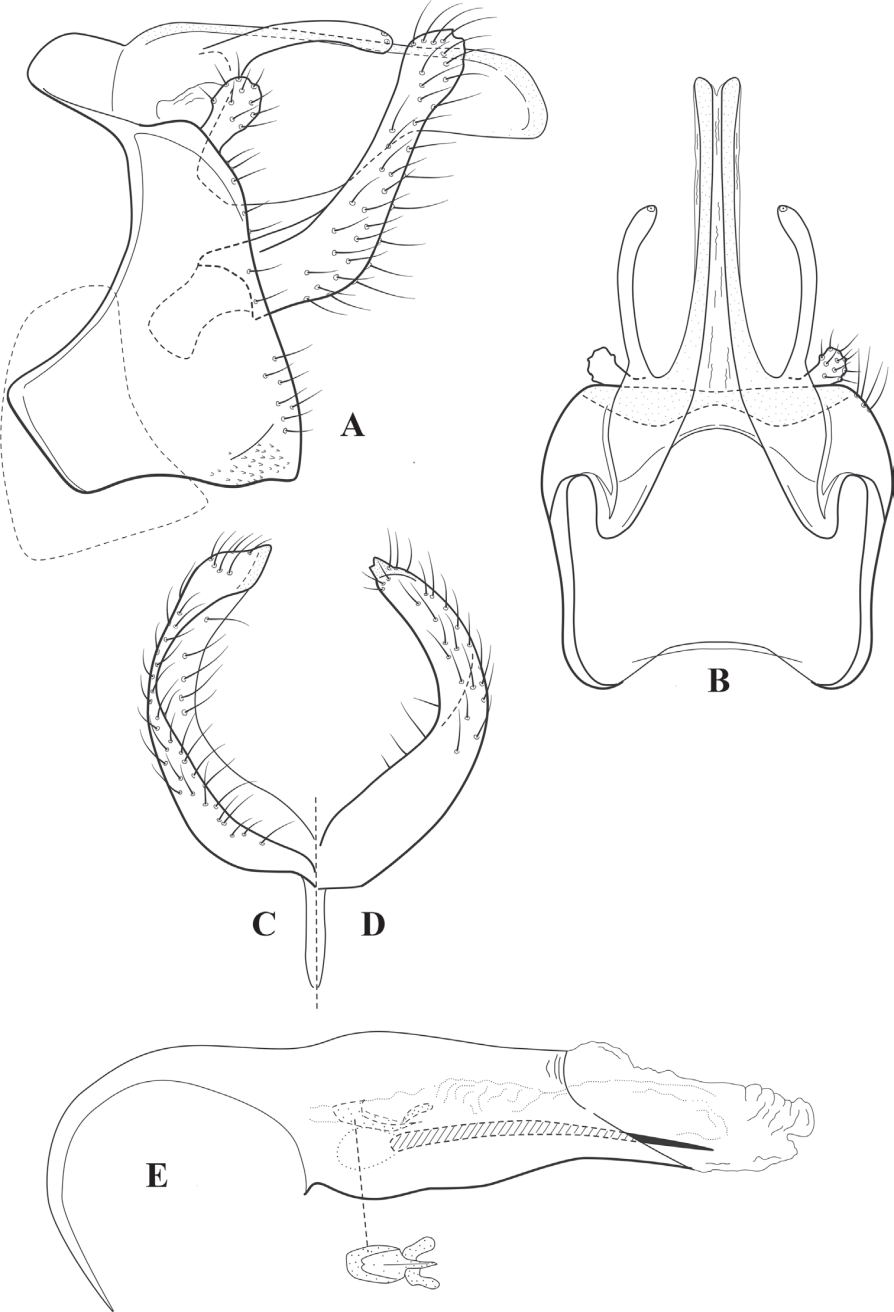
*Chimarrakrugeri* Jacquemart, ♂ genitalia **A** lateral **B** dorsal, segments IX and X **C** inferior appendage, ventral **D** inferior appendage, dorsal **E** phallus, lateral.

###### Redescription.

***Adult.*** Overall color (in alcohol) nearly uniformly yellowish brown. Head elongate (postocular parietal sclerite subequal in length to diameter of eye). Palps elongate; maxillary palp with 1^st^ segment very short (slightly longer than wide), 2^nd^ segment elongate (distinctly longer than 3^rd^), apex with cluster of stiff setae, 3^rd^ segment moderately elongate (normal), 4^th^ segment short (~ 2× 1^st^), 5^th^ segment elongate (subequal to 3^rd^). Forewing length: male, 5.9 mm. Fore- and hind wings with forks I, II, III, and V present. Forewing with R_1_ somewhat sinuous, stem of Rs short, inflected at approximately midlength, with small node extending into cell below, discoidal cell very short, width subequal to length, basal fork not enlarged, forks I and II (of both fore- and hind wings) very elongate, forks petiolate, *r* crossvein diagonal, intersecting discoidal cell at approximately midlength, *r-m* crossvein of forewing slightly proximal to *s*, *m* crossvein proximal to *r-m*, very near basal fork of M, 2A with crossvein (apparently forked apically to 1A and 3A). Hind wing with R_1_ narrowly parallel to subcosta, discoidal cell very small. Foreleg with apical tibial spur short; male with foretarsi modified, claws enlarged and symmetrical.

***Male genitalia*.** Segment VIII relatively short. Segment IX, in lateral view, relatively short, anterior margin distinctly, subangularly, produced in ventral ¼, dorsolaterally with very prominent, broadly rounded apodeme, margin concave between; tergum very short and narrowly sclerotized, but continuous dorsally, in dorsal view, forming concave excavation between lateral apodemes; posterior margin obliquely and linearly widened from preanal appendage to ventral process; ventral margin extended apically to form very basally wide, weakly projecting, subtriangular ventral process. Segment IX, in dorsal or ventral views, with anteroventral margin moderately concave mesally. Lateral lobes of tergum X very elongate, subtruncately rounded apically, dorsal margin distinctly sclerotized, basodorsally with elongate, posteriorly oriented, digitate, sensilla-bearing process; lateral lobes, in dorsal view, narrowly parallel; mesal lobe of tergum X membranous, nearly as long as lateral lobes, inconspicuous because of closely apposed lateral lobes. Preanal appendages short, constricted basally, knob-like. Inferior appendage, in lateral view, relatively elongate, narrow, strongly dorsally flexed near base, apex distinctly widened, extreme apex weakly notched or bifid, noticeable in some orientations; appendage, in dorsal or ventral views, more or less uniformly mesally curved. Phallic apparatus with phallobase broadly tubular, with usual basodorsal expansion, apicoventral margin moderately projecting; endotheca with single, very elongate spine, nearly as long as ventral margin of phallobase; phallotremal sclerite complex composed of very short rod and ring structure, with small, paired apicolateral sclerites.

###### Distribution.

Republic of South Africa, Tanzania.

##### ﻿ Chimarra morogoroensis sp. nov.

Taxon classificationAnimaliaTrichopteraPhilopotamidae

0E736ACC-C143-53AB-95D5-52D53AB6DDA5

http://zoobank.org/0B638A45-3548-4BFE-B932-00FEB3A6CA1C

Fig. 18A–E

###### Type material.

***Holotype*.** Tanzania – **Morogoro Reg.** ● ♂ (in alcohol); Morogoro, Teachers College; 6°49'S, 37°42'E; 12 Dec. 1990; T Andersen leg.; sweep net; UMSP 000550015. ***Paratype*.** Tanzania – **Morogoro Reg.** ● 1♂; same data as for holotype; ZMBN.

###### Diagnosis.

*Chimarramorogoroensis* is very similar to *C.szunyoghyi* Oláh, and we are not completely sure that it is a distinct species. The inferior appendages, tergum X, and the general form of segment IX and its ventral process are more or less identical in the two species. Like *C.szunyoghyi* and *C.tanzaniensis* sp. nov., the apex of the inferior appendages is very distinctly developed and acute, more distinctly so in both *C.morogoroensis* and *C.szunyoghyi* than in *C.tanzaniensis*, which usefully distinguishes them. Differences between *C.morogoroensis* and *C.szunyoghyi* are found mostly in phallic structures. The differences, however, are qualitative and involve characters that could function in isolating the two species. The primary differences are the elongate, extensible dorsal lobe on the endotheca in *C.szunyoghyi*, with two small apical spines, and the very much extended and sharply downturned ventral apex of the phallobase in *C.morogoroensis*. In *C.morogoroensis*, the dorsal phallic lobe appears to be relatively simple and much shorter, without apical spines, and in *C.szunyoghyi*, the ventral apex of the phallobase is only very weakly developed and projecting. We consider the differences significant enough to warrant the recognition of two species.

**Figure 18.**
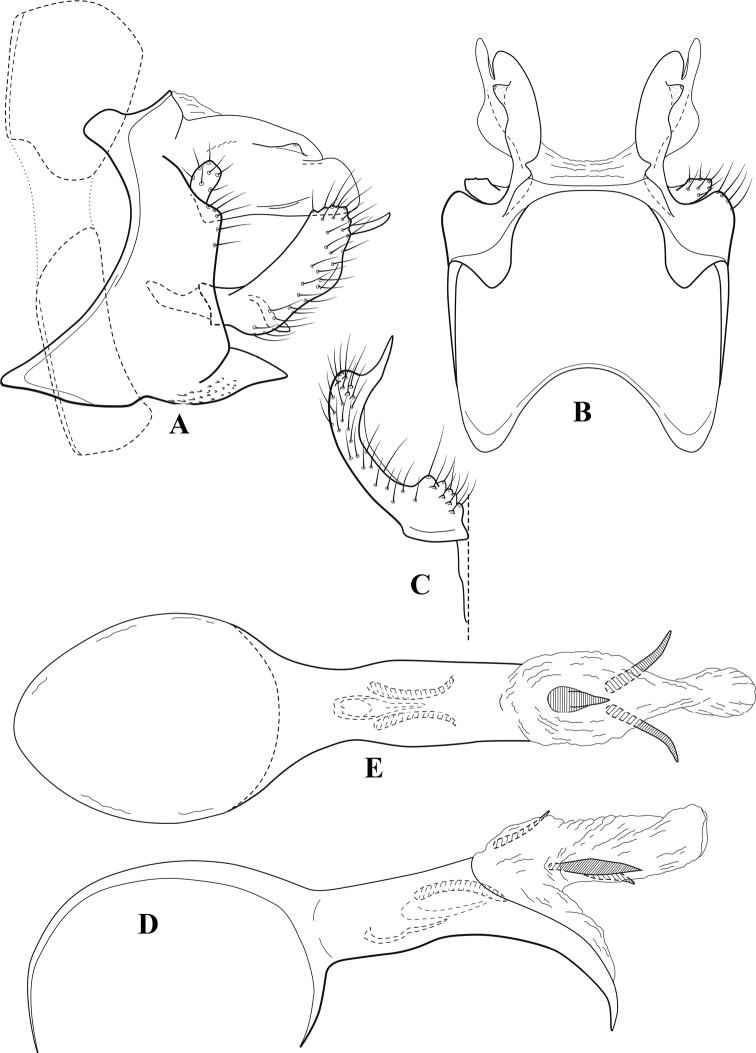
*Chimarramorogoroensis* sp. nov., ♂ genitalia **A** lateral **B** dorsal, segments IX and X **C** inferior appendage, ventral **D** phallus, lateral **E** phallus, dorsal.

###### Description.

***Adult.*** Overall color (in alcohol) medium brown, vertex of head darker than setal warts. Head elongate (postocular parietal sclerite nearly as long as diameter of eye). Palps relatively short, maxillary palp with 1^st^ segment short (approximately as long as wide), 2^nd^ segment short (~ 2× length of 1^st^), apex with small cluster of stiff setae, 3^rd^ elongate (almost 2× length of 2^nd^), 4^th^ segment short (shorter than 2^nd^), 5^th^ segment elongate (subequal to 3^rd^). Forewing length: male, 5.9 mm. Fore- and hind wings with forks I, II, III, and V present. Forewing with R_1_ somewhat sinuous, stem of Rs inflected at approximately midlength, with distinct node at inflection, extending into cell below, basal fork of discoidal cell enlarged, very asymmetric, discoidal cell elongate, length greater than two × width, forks I and II sessile, *r* crossvein diagonal, intersecting discoidal cell at past midlength, just before fork I, *r-m* crossvein nearly continuous with *s*, *m* crossvein proximal to *s* and *r-m* crossveins, slightly closer to *r-m* crossvein than basal fork of M, *s* pigmented (like wing), *r-m* and *m* crossveins hyaline, very weakly developed, 2A with crossvein (apparently forked apically to 1A and 3A). Hind wing with R_1_ narrowly parallel to subcosta, forks I and II approximately sessile. Foreleg with apical tibial spur short; male with foretarsi not modified.

***Male genitalia*.** Segment VIII relatively short, tergum slightly longer dorsally. Segment IX, in lateral view, moderate in length, anterior margin strongly, angularly produced ventrally, dorsolaterally with distinct rounded apodemes, margin strongly concave between; tergum, in dorsal view, continuous between apodemes, but very short, forming deeply concave excavation; posterior margin short dorsally, weakly, obtusely produced below preanal appendages, more or less linearly widening ventrally to ventral process; posteroventral margin with rather prominent, subtriangular, posteriorly projecting, ventral process, length greater than width at base, apex acute. Segment IX, in dorsal or ventral views, with anteroventral margin deeply concave mesally. Lateral lobes of tergum X moderate in length, rounded apically, with very short, rounded, sensilla-bearing process near dorsal margin at approximately midlength, ventrolaterally with compressed, rounded projection, hardly evident in lateral view, but forming distinct rounded projection, as viewed dorsally; mesal lobe of tergum X membranous, short, only at base of lateral lobes. Preanal appendages short, rounded, constricted basally. Inferior appendage, in lateral view, relatively narrow and short, dorsally flexed near base, with apex forming very distinct spine-like projection, readily visible in both lateral and ventral views; appendage, in dorsal or ventral views, moderately mesally curved, with short basomesal enlargement at basal inflection, apex very prominent and spine-like, somewhat mesally curved. Phallic apparatus with phallobase relatively short and tubular, with usual basodorsal expansion, apicoventral margin forming a very distinct and strongly ventrally curved projection, apex acute; endotheca membranous, without minute spines, but with three very distinct, moderate elongate spines, one dorsomesal and two lateral, symmetrically positioned; phallotremal sclerite complex composed of moderately elongate rod and ring structure, with pair of distinct, narrow, curved, dorsolateral sclerites.

###### Etymology.

*Chimarramorogoroensis*, used as an adjective and meaning “from Morogoro” in reference to the town in Tanzania where this species was collected.

##### ﻿ Chimarra pedaliotus sp. nov.

Taxon classificationAnimaliaTrichopteraPhilopotamidae

53D519A0-D155-5415-A87E-1AE9BE50B7E0

http://zoobank.org/E741B076-E7DD-46D8-88DB-29BAD6D1020D

Fig. 19A–G

###### Type material.

***Holotype*.** Ghana – **Volta Reg.** ● ♂ (in alcohol); Wli, Agumatsa waterfall, station # 12; 7°07'29"N, 0°35'31"E; 7 Mar. 1993; JS Amakye & J Kjærandsen leg.; light trap; UMSP 000550008. ***Paratypes*.** Ghana – **Volta Reg.** ● 1♂; same data as for holotype except station # 10^A^; 7–10 Mar. 1993; Malaise trap; ZMBN ● 1♂; same data as for holotype except station # 3; 10 Mar. 1993; light trap; ZMBN ● 4♂♂; same data as for holotype except 17 Nov. 1993; J Kjærandsen leg.; ZMBN ● 2♂♂; same data as for holotype except station # 6; 11 Mar. 1993; ZMBN ● 1♂; same data as for holotype except 20 Nov. 1993; J Kjærandsen leg.; ZMBN ● 1♂; same data as for holotype except station # 10; 8 Mar. 1993; ZMBN ● 1♂; same data as for holotype except station # 3; 12 Nov. 1993; J Kjærandsen leg.; sweep net; ZMBN. – **Eastern Reg.** ● 1♂; Boti Falls; 6°11'40"N, 0°13'05"W; 19 Nov. 1991; J Amakye leg.; light trap; ZMBN ● 1♂; Kibi, Subri stream; 6°10'N, 0°33'W; 5 Nov. 1993; J Kjærandsen leg.; light trap; ZMBN. Cameroon ● 20♂♂1♀; Muguka, Victoria Division; 24–29 June 1949; B Malkin leg.; INHS ● 2♂♂; same collection data as for preceding; UMSP ● 1♂; Victoria, British Cameroons; May 1949; B Malkin leg.; INHS. NIGERIA ● 1♂; Cross River State, Ikom, Igoja Prov.; 6 Jan. 1949; B Malkin leg.; INHS.

**Figure 19.**
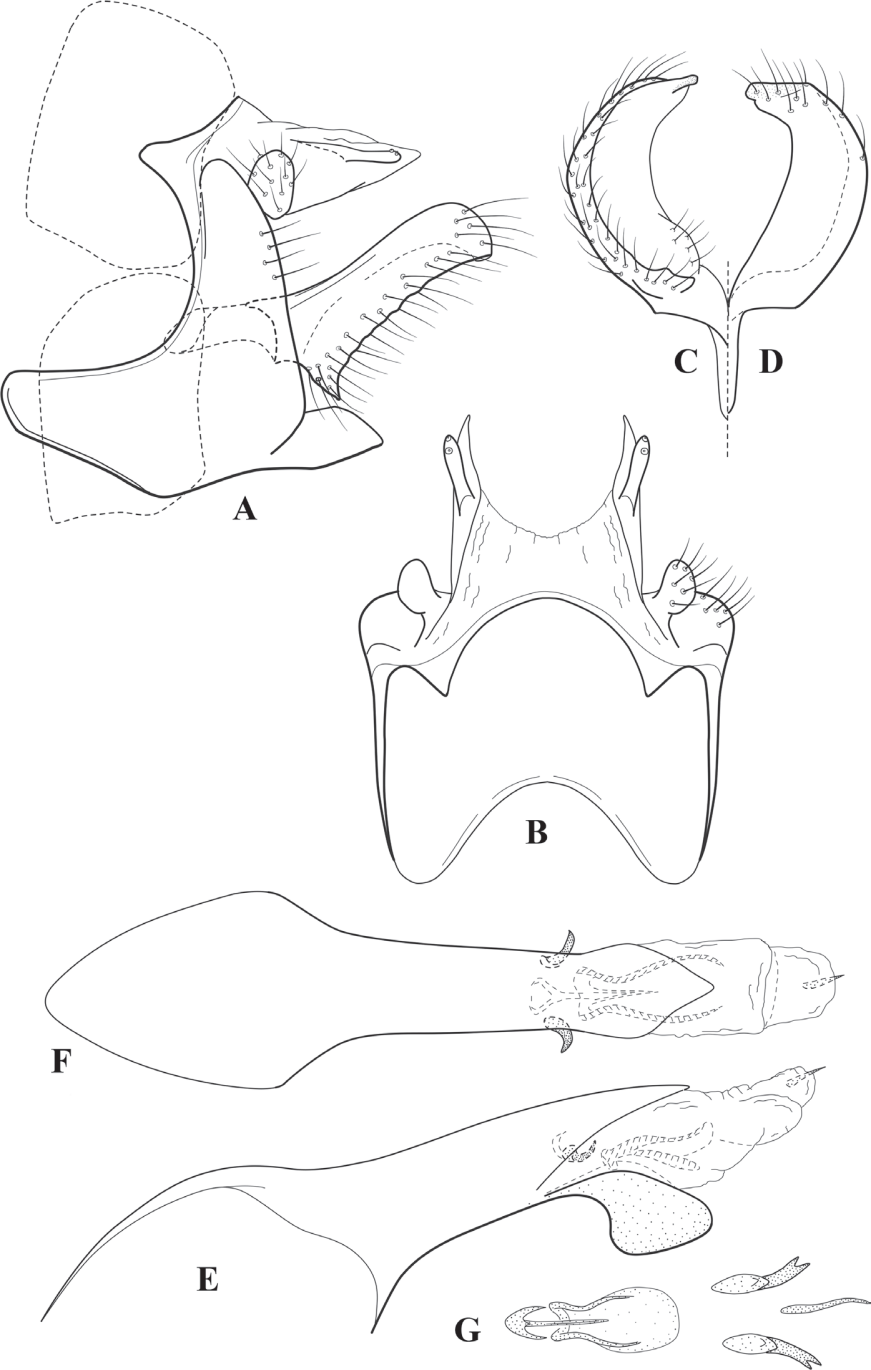
*Chimarrapedaliotus* sp. nov., ♂ genitalia **A** lateral **B** dorsal, segments IX and X **C** inferior appendage, ventral **D** inferior appendage, dorsal **E** phallus, lateral **F** phallus, dorsal **G** phallic armature, dorsal, variant from Cameroon.

###### Additional material.

Ghana – **Eastern Reg.** ● 2♀♀; Boti Falls; 6°11'40"N, 0°13'05"W; 19 Nov. 1991; JS Amakye leg.; light trap; ZMBN ● 1♀; same collection data as for preceding except 28 Oct.–4 Nov. 1994; T Andersen leg.; Malaise trap; ZMBN ● 8♀♀; Kibi, Subri stream; 6°10'N, 0°33'W; 5 Nov. 1993; J Kjærandsen leg.; light trap; ZMBN. – **Volta Reg.** ● 2♀♀; Hohoe, Matvin Hotel; 7°09'43"N, 0°28'31"E; 11 Nov. 1993; J Kjærandsen leg.; at light; ZMBN ● 1♂; Fodoma, Nubui stream; 7 June 1995; T Andersen & J Kjærandsen leg.; light trap; UMSP ● 1♀; Wli, Agumatsa waterfall, station # 3^B^; 7°07'29"N, 0°35'31"E; 4–7 Mar. 1993; JS Amakye & J Kjærandsen leg.; Malaise trap; ZMBN ● 1♀; same collection data as for preceding except station # 9^B^; UMSP ● 1♀; same collection data as for preceding except station # 4^B^; 7–10 Mar. 1993; ZMBN ● 5♀♀; same collection data as for preceding except station # 3; 10 Mar. 1993; light trap; ZMBN ● 6♀♀; same collection data as for preceding except 17 Nov. 1993; J Kjærandsen leg.; ZMBN ● 1♀; same collection data as for preceding except station # 6; 11 Mar. 1993; JS Amakye & J Kjærandsen leg.; ZMBN ● 2 ♀♀; same collection data as for preceding except 20.xi.1993; J Kjærandsen leg.; ZMBN ● 12♀♀; same collection data as for preceding except station # 10; 19 Nov. 1993; ZMBN ● 30♀♀; same collection data as for preceding except station # 12; 16 Nov. 1993; ZMBN.

###### Diagnosis.

Inferior appendage relatively short, uniform in width in lateral view, apex subacute in ventral view; ventral apex of phallobase expanded, laterally compressed, and ventrally deflexed; phallus with pair of small, curved, symmetrically placed spines; apex of lateral lobe of tergum X subacute.

*Chimarrapedaliotus* is similar to *C.occidentalis* Gibon but is easily diagnosed by the very distinctive apex of the phallobase, which is enlarged and rounded, as viewed laterally, and also strongly compressed. Specimens from Cameroon differed slightly in the armature of the phallus, including phallic spines that tended to be bifid apically and were also somewhat larger, and a more elongate apical spine. The differences are rather minor and somewhat variable even in the material examined; on the other hand, the overall similarity is significant. We do not consider the differences significant enough to warrant varietal or species status. Future collecting may require a reassessment of this conclusion.

###### Description.

***Adult.*** Overall color (in alcohol) yellowish brown. Head short and rounded (length of postocular parietal sclerite ~ 1/2 diameter of eye). Palps relatively short, maxillary palp with 1^st^ segment very short (length subequal to width), 2^nd^ segment relatively short (~ 3× 1^st^), apex with small cluster of stiff setae, 3^rd^ segment slightly longer than 2^nd^, 4^th^ segment short (~ 1/2 length of 3^rd^), 5^th^ segment short (subequal to 2^nd^). Forewing length: male, 3.8–5.0 mm; female, 4.5–5.5 mm. Fore- and hind wings with forks I, II, III, and V present. Forewing with R_1_ somewhat sinuous, stem of Rs weakly inflected at approximately midlength, with node at inflection, extending into cell below, basal fork of discoidal cell enlarged, fork very asymmetric, length of cell ~ 2× width, forks I and II slightly subsessile, *r* crossvein diagonal, intersecting discoidal cell at past midlength, just before fork I, *m* crossvein proximal to *s* and *r-m* crossveins, approximately midway between basal fork of M and *r-m* crossvein, *s* pigmented (like wing), *r-m* and *m* crossveins hyaline and very faint, 2A with crossvein (apparently forked apically to 1A and 3A). Hind wing with R_1_ narrowly parallel to subcosta, forks I and II slightly subsessile. Foreleg with apical tibial spur short; male with foretarsi modified, claws enlarged, outer claw twisted and asymmetric.

***Male genitalia*.** Segment VIII moderately elongate, sternum and tergum subequal in length. Segment IX, in lateral view, elongate, anterior margin strongly, subangularly produced in ventral 1/3, dorsolaterally with distinct rounded apodeme, margin strongly convex between; tergum, in dorsal view, continuous between apodemes, but very short, forming deeply concave excavation; posterior margin nearly linear, slightly widening ventrally; ventral margin with rather prominent, posteriorly projecting, ventral process, length greater than width at base, apex subacute. Segment IX, in dorsal or ventral views, with anteroventral margin deeply concave mesally. Lateral lobes of tergum X relatively short, subacute apically, with moderately elongate, posteriorly projecting, digitate, sensilla-bearing process near dorsal margin in basal half; mesal lobe of tergum X membranous, ~ 1/2 length of lateral lobes. Preanal appendages short, rounded, constricted basally. Inferior appendage, in lateral view, only moderately elongate, nearly uniform in width, dorsally flexed near base, with small angular ventral projection at point of inflection, apex incurved and forming short, rounded projection, not visible in lateral view; appendage, in dorsal or ventral views, uniformly and only moderately mesally curved. Phallic apparatus with phallobase tubular, with usual basodorsal expansion, dorsal margin tapering and acute apically, but only weakly sclerotized, apicoventral margin forming a broadly rounded and deflexed, laterally compressed and keel-like, sclerotized projection; endotheca relatively short and without minute spines, basally with pair of short, curved, symmetrical, laterally emergent spines; phallotremal sclerite complex composed of moderately elongate rod and ring structure, with narrow, paired, dorsolateral sclerites.

###### Etymology.

*Chimarrapedaliotus*, as an adjective from the Greek *pedaliotos*, meaning “with a rudder,” and referring to the keeled and somewhat rudder-like ventral apex of the phallobase in this species.

##### ﻿ Chimarra szunyoghyi

Taxon classificationAnimaliaTrichopteraPhilopotamidae

Oláh, 1986

4BB19C50-67CA-59B5-919D-ECCABC8D90C3

Fig. 20A–E

Chimarra szunyoghyi Oláh, 1986: 141–143, fig. 1A–D.

###### Material examined.

Tanzania – **Tanga Reg.** ● 1♂; West Usambara Mts, Mazumbai, Kaputu Stream; 4°48'S, 38°30'E; 26 Nov. 1990; T Andersen leg.; Malaise trap; UMSP ● 1♂; INHS.

###### Diagnosis.

*Chimarraszunyoghyi* is very similar to *C.morogoroensis* sp. nov., as discussed in the diagnosis for that species. Both species have general features of the inferior appendages, tergum X, and the general shape of segment IX, including its ventral process, nearly identical. The elongate, acute apical projection on the inferior appendages is usefully diagnostic for both species, and differs from the similar, but shorter, projection found in *C.tanzaniensis* sp. nov. The primary differences separating *C.szunyoghyi* from *C.morogoroensis* are found in structures of the phallic apparatus and include, especially, the elongate, extensible dorsal lobe on the endotheca, found in *C.szunyoghyi*, which has a pair of small apical spines, and the very elongate and strongly downturned ventral apex of the phallobase found in *C.morogoroensis*. The dorsal lobe on the endotheca in *C.morogoroensis* is simpler, much shorter, and lacks apical spines, and the ventral apex of the phallobase in *C.szunyoghyi* is much shorter. The differences, while minor, are distinctive.

**Figure 20.**
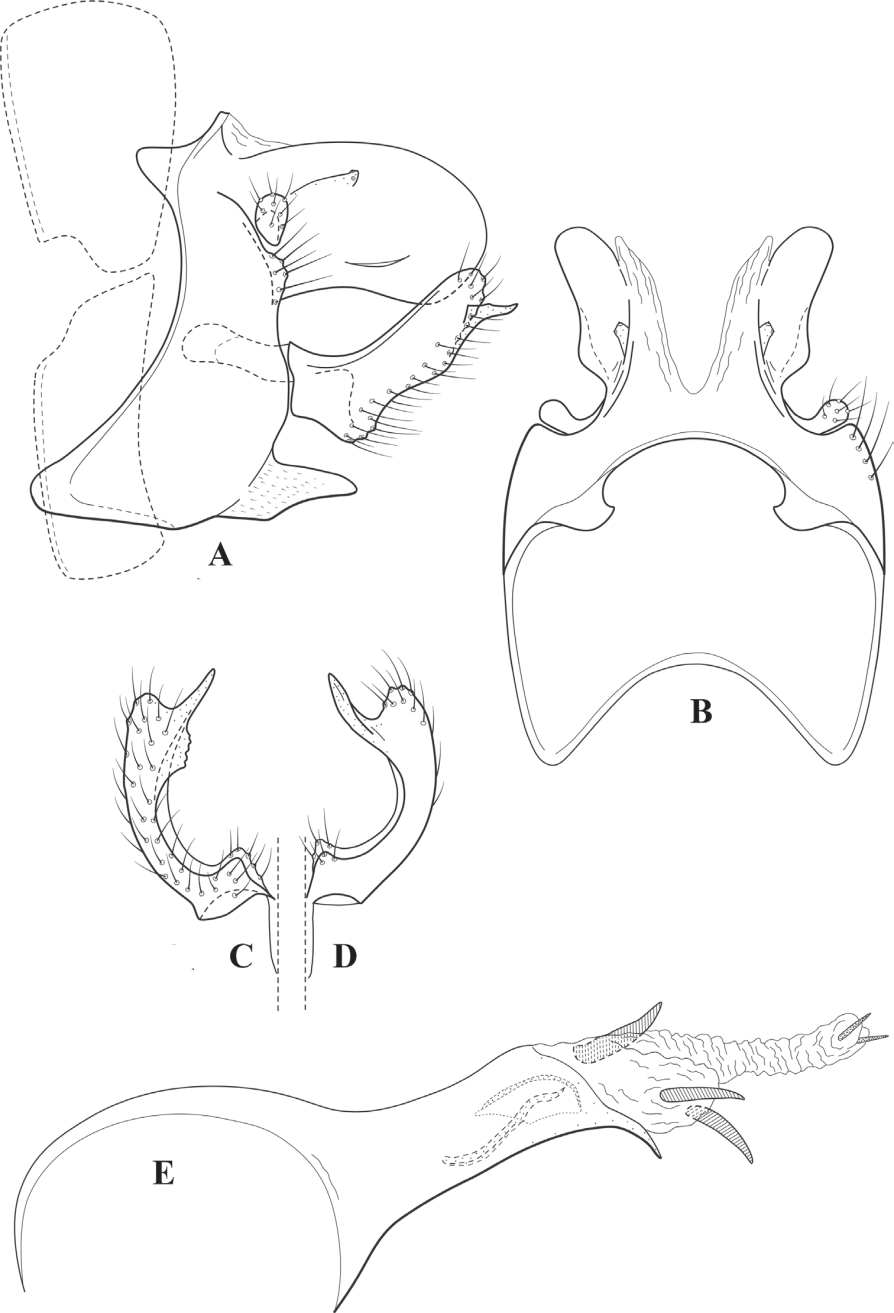
*Chimarraszunyoghyi* Oláh, ♂ genitalia **A** lateral **B** dorsal, segments IX and X **C** inferior appendage, ventral **D** inferior appendage, dorsal **E** phallus, lateral.

###### Redescription.

***Adult.*** Overall color (in alcohol) medium brown, vertex of head darker than setal warts. Head elongate (postocular parietal sclerite nearly as long as diameter of eye). Palps relatively short, maxillary palp with 1^st^ segment short (approximately as long as wide), 2^nd^ segment short (~ 2× length of 1^st^), apex with small cluster of stiff setae, 3^rd^ elongate (almost 2× length of 2^nd^), 4^th^ segment short (shorter than 2^nd^), 5^th^ segment elongate (slightly longer than 3^rd^). Forewing length: male, 5.1 mm. Fore- and hind wings with forks I, II, III, and V present. Forewing with R_1_ somewhat sinuous, stem of Rs inflected at approximately midlength, with distinct node at inflection, extending into cell below, basal fork of discoidal cell enlarged, very asymmetric, discoidal cell moderately elongate, length ~ 2× width, forks I and II slightly subsessile, *r* crossvein diagonal, intersecting discoidal cell at past midlength, just before fork I, *r-m* crossvein nearly continuous with *s*, *m* crossvein proximal to *s* and *r-m* crossveins, approximately midway between basal fork of M and *r-m* crossvein, *s* pigmented (like wing), *r-m* and *m* crossveins hyaline, very weakly developed, 2A with crossvein (apparently forked apically to 1A and 3A). Hind wing with R_1_ narrowly parallel to subcosta, forks I and II subsessile. Foreleg with apical tibial spur short; male with foretarsi apparently weakly modified, claws symmetric, slightly enlarged.

***Male genitalia*.** Segment VIII relatively short, tergum slightly longer dorsally. Segment IX, in lateral view, moderate in length, anterior margin strongly, angularly produced ventrally, dorsolaterally with distinct rounded apodeme, margin strongly convex between; tergum, in dorsal view, continuous between apodemes, but very short, forming deeply concave excavation; posterior margin short dorsally, weakly, obtusely produced below preanal appendages, more or less linearly widening ventrally to ventral process; posteroventral margin with rather prominent, subtriangular, posteriorly projecting, ventral process, length greater than width at base, apex acute. Segment IX, in dorsal or ventral views, with anteroventral margin deeply concave mesally. Lateral lobes of tergum X moderate in length, rounded apically, with very short, rounded, sensilla-bearing process near dorsal margin in basal half, ventrolaterally with compressed, rounded projection, hardly evident in lateral view, but forming distinct rounded projection, as viewed dorsally; mesal lobe of tergum X membranous, short, and divided mesally, more extended laterally. Preanal appendages short, rounded, constricted basally. Inferior appendage, in lateral view, relatively narrow and short, dorsally flexed near base, with apex forming very distinct spine-like projection, readily visible in both lateral and ventral views; appendage, in dorsal or ventral views, moderately mesally curved, with short basomesal enlargement at basal inflection, apex very prominent and spine-like, somewhat mesally curved. Phallic apparatus with phallobase relatively short and tubular, with usual basodorsal expansion, apicoventral projection short, acute, only weakly projecting and deflexed; endotheca membranous, without minute spines, but with very elongate, pleated, extensible, membranous lobe with two short apical spines, endotheca also with three very distinct, moderately long spines, one dorsomesal and two lateral, symmetrically positioned, extensible lobe, when extended, as long or longer than ventral margin of phallobase; phallotremal sclerite complex composed of moderately elongate rod and ring structure, with pair of distinct, narrow, curved, dorsolateral sclerites.

###### Distribution.

Tanzania.

##### ﻿ Chimarra tanzaniensis sp. nov.

Taxon classificationAnimaliaTrichopteraPhilopotamidae

D45D19D1-B15E-50DD-BFB7-C5818E18631C

http://zoobank.org/C728A834-7039-47DE-AF50-D22EA22BCF22

Fig. 21A–E

###### Type material.

***Holotype*.** Tanzania – **Tanga Reg.** ● ♂ (in alcohol); West Usambara Mts, Mazumbai, Kaputu Stream; 4°48'S, 38°30'E; 30 Oct. 1990–12 Feb. 1991; T Andersen leg.; Malaise trap; UMSP 000550066. ***Paratypes*.** Tanzania – **Tanga Reg.** ● 11♂♂; same data as for holotype; ZMBN ● 2♂♂; same data as for holotype except 4–12 Feb.1991; UMSP ● 2♂♂; same data as for holotype except 5 Nov. 1990; sweep net; ZMBN.

###### Diagnosis.

*Chimarratanzaniensis* probably has its overall closest similarity to *C.quadrispinosa* Jacquemart & Statzner, particularly in the overall shape of segment IX and inferior appendages, which have a very similar shape and acute, spine-like apices. The apices of the inferior appendages also resemble *C.szunyoghyi* Oláh, but are not quite so pronounced as in that species. Differences from *C.quadrispinosa* include a less produced posteroventral margin of segment IX, absence of distinct basomesal projections on the inferior appendages, and a different armature of the phallus. The four spines of *C.quadrispinosa*, based on its illustration, seem to include two prominent, symmetrically placed dorsal spines, which are common among various species of the *kenyana* group, and two apical spines, possibly elements of the phallotremal sclerite complex. The phallotremal sclerite complex of *C.tanzaniensis* also has elongate lateral sclerites, but the dorsal spines in this species are very small and occur at the end of a narrow membranous projection, much as that found in *C.szunyoghyi*. The overall differences are significant enough to warrant the recognition of a new species.

**Figure 21.**
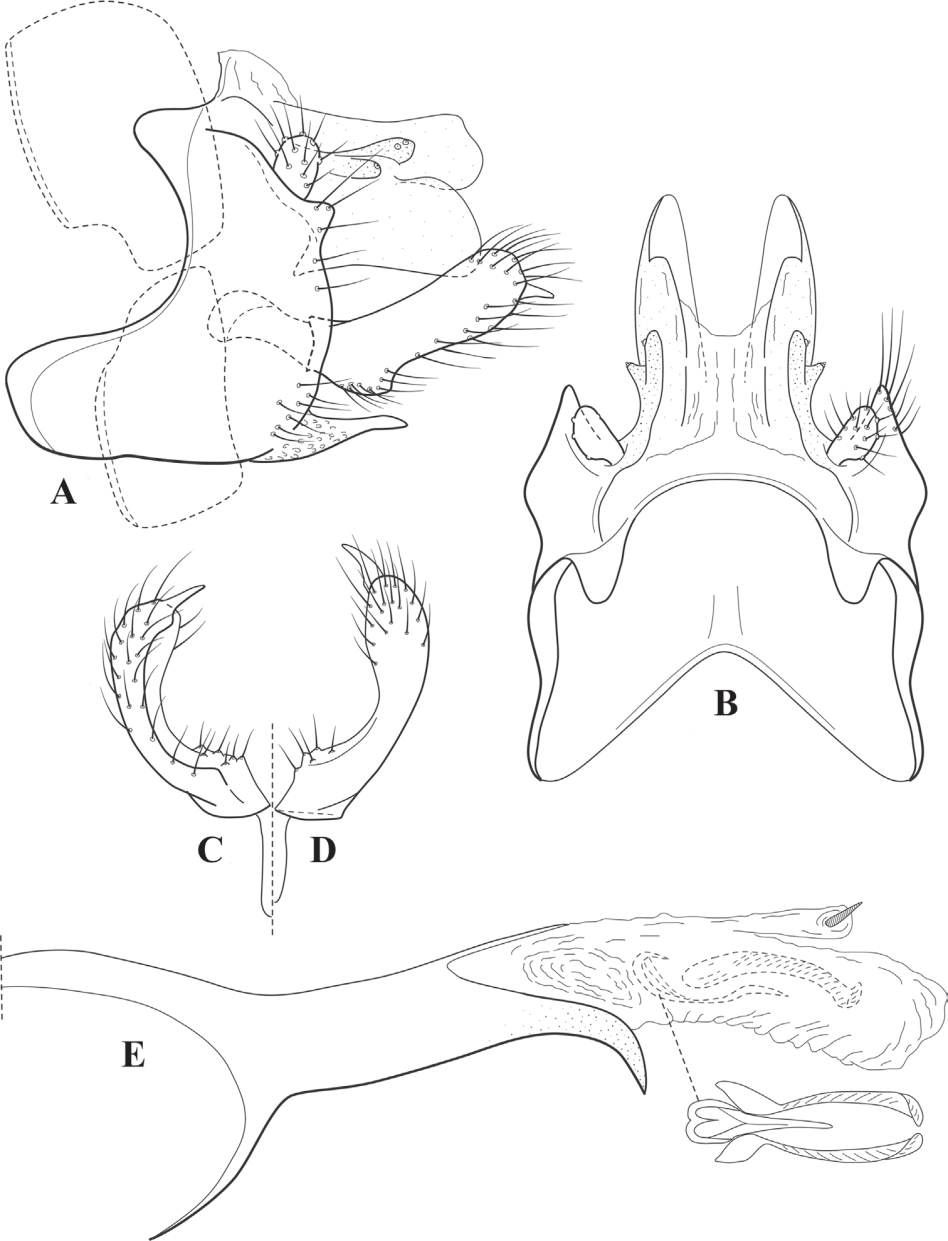
*Chimarratanzaniensis* sp. nov., ♂ genitalia **A** lateral **B** dorsal, segments IX and X **C** inferior appendage, ventral **D** inferior appendage, dorsal **E** phallus, lateral, with dorsal detail of phallotremal sclerite complex.

###### Description.

***Adult.*** Overall color (in alcohol) dark brown. Head relatively short (postocular parietal sclerite ~1/2 diameter of eye). Palps moderately elongate, maxillary palp with 1^st^ segment short (length slightly greater than width), 2^nd^ segment short (~ 2× length of 1^st^), apex with small cluster of stiff setae, 3^rd^ elongate (~ 2× as long as 2^nd^), 4^th^ segment short (shorter than 2^nd^), 5^th^ segment elongate (subequal to 3^rd^). Forewing length: male, 6.0–7.5 mm. Fore- and hind wings with forks I, II, III, and V present. Forewing with R_1_ somewhat sinuous, stem of Rs inflected at approximately midlength, with distinct node at inflection, extending into cell below, basal fork of discoidal cell enlarged, fork asymmetric, discoidal cell elongate, length > 2× its width, forks I and II sessile, *r* crossvein diagonal, intersecting discoidal cell at past midlength, just before fork I, *r-m* crossvein diagonal, continuous with *s*, *m* crossvein proximal to *s* and *r-m* crossveins, approximately midway between basal fork of M and *r-m* crossvein, *s* pigmented (like wing), *r-m* and *m* crossveins hyaline, 2A with crossvein (apparently forked apically to 1A and 3A). Hind wing with R_1_ narrowly parallel to subcosta, forks I and II subsessile. Foreleg with apical tibial spur short; male with foretarsi unmodified, or nearly so, claws small and symmetrical.

***Male genitalia*.** Segment VIII moderate in length, tergum slightly longer dorsally. Segment IX, in lateral view, relatively elongate, anterior margin very strongly produced ventrally, forming rounded lateral projection in ventral 1/3, dorsolaterally with distinct rounded apodeme, margin strongly concave between; tergum, in dorsal view, continuous between apodemes, but very short, forming deeply concave excavation; posterior margin short dorsally, weakly produced below preanal appendages, more or less linear to ventral process; posteroventral margin with prominent, moderately elongate, posteriorly projecting, ventral process, length > 2× width at base, apex acute. Segment IX, in dorsal or ventral views, with anteroventral margin deeply, angularly, concave mesally. Lateral lobes of tergum X moderate in length, relatively wide, with apex partially divided into rounded dorsal and ventral lobes, dorsal lobe with very short, rounded, sensilla-bearing process in basal half; mesal lobe of tergum X membranous, extending ~ 1/2 length of lateral lobes. Preanal appendages short, rounded, constricted basally. Inferior appendage, in lateral view, relatively narrow and short, dorsally flexed near base, with apex forming distinct, short, spine-like projection, visible in both lateral and ventral views; appendage, in dorsal or ventral views, moderately mesally curved, with distinct basomesal enlargement at basal inflection, apex narrowed and spine-like, curvature more or less continuous with lateral margin of appendage. Phallic apparatus with phallobase moderate in length and tubular, with usual basodorsal expansion, apicoventral margin with distinct, ventrally curved projection, apex acute; endotheca membranous, without minute spines, but with narrow membranous dorsal lobe, with small apical spine; phallotremal sclerite complex composed of moderately elongate rod and ring structure, with pair of distinct, narrow, curved, dorsolateral sclerites.

###### Etymology.

*Chimarratanzaniensis*, used as an adjective and meaning “from Tanzania,” in reference to the country of origin of the holotype specimen.

##### ﻿ Chimarra triangularis occidentalis

Taxon classificationAnimaliaTrichopteraPhilopotamidae

Gibon, 1985

3474BA08-6594-544C-8D10-7BC2E4824A6A

Fig. 22A–G

Chimarra triangularis occidentalis Gibon, 1985: 27, figs 11–12.

###### Marterial examined.

Ghana – **Western Reg.** ● 1♂; Ankasa Game Production Reserve; 5°15'N, 2°37'W; 11 Dec. 1993; T Andersen & J Kjærandsen leg.; light trap; UMSP ● 12♂♂9♀♀; same collection data as for preceding except 5–9 Dec. 1993; ZMBN ● 1♀; same collection data as for preceding; UMSP ● 3♂♂; same collection data as for preceding except 31 Apr. 1993; J Kjærandsen leg.; ZMBN ● 2♂♂; same collection data as for preceding except 6–12 Dec. 1993; Malaise trap; ZMBN.

###### Diagnosis.

Characters, in combination, that confirm the identification and can be used to distinguish *C.triangularisoccidentalis* from the nominate subspecies and other species in the subgroup include: the general shape of inferior appendages, shape of segment IX and ventral process, relatively short tergum X and length of sensilla-bearing process, and presence of large apical phallic spine.

**Figure 22.**
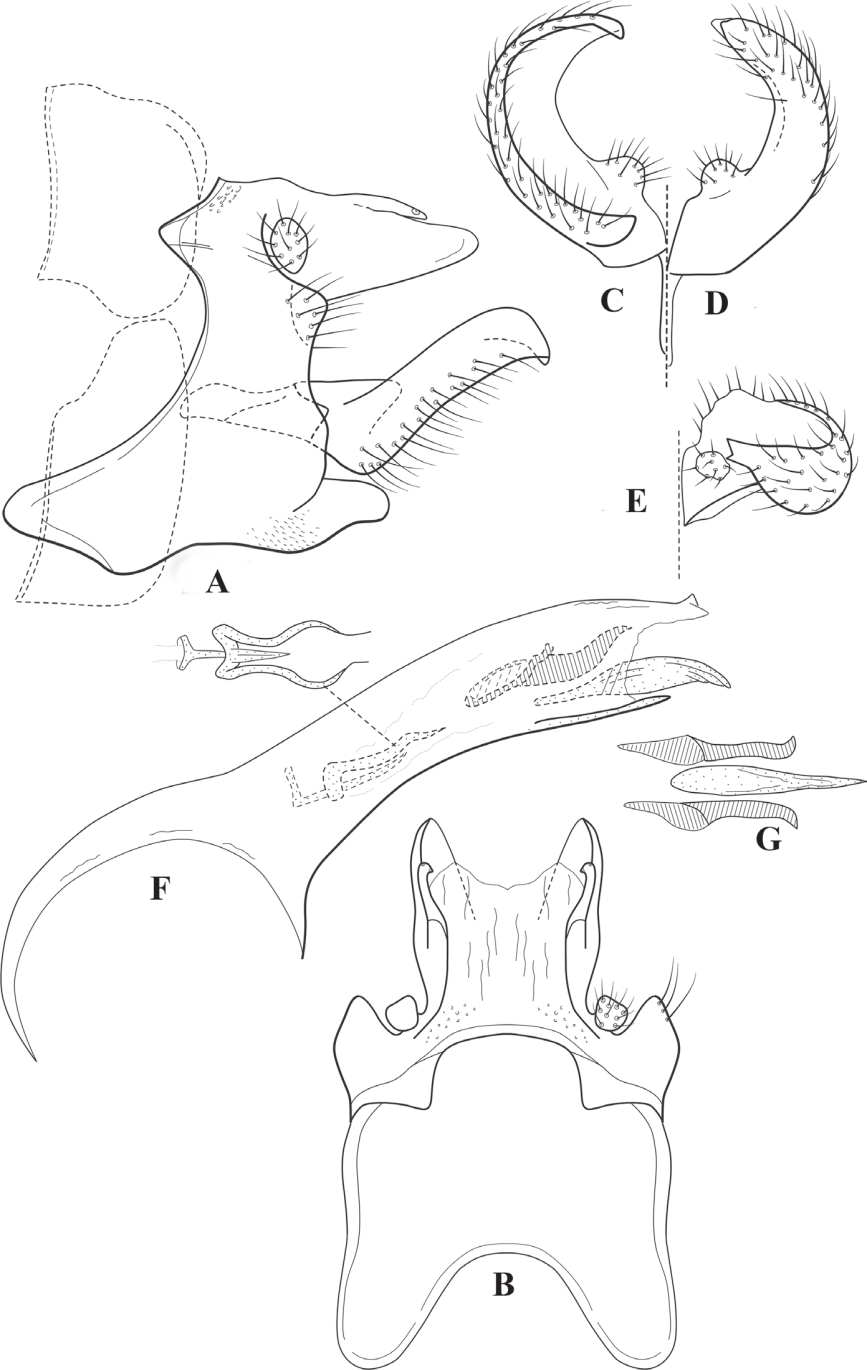
*Chimarratriangularisoccidentalis* Gibon, ♂ genitalia **A** lateral **B** dorsal, segments IX and X **C** inferior appendage, ventral **D** inferior appendage, dorsal **E** inferior appendage, oblique dorsal **F** phallus, lateral, with dorsal detail of phallotremal sclerite complex **G** phallic spines, dorsal.

*Chimarratriangularisoccidentalis* was considered a subspecies of *C.triangularis* Kimmins when described by Gibon, probably because of the overall similarity between the two forms in the shape of the inferior appendages, length of tergum X, and similarity of its phallic armature. Kimmins described *C.triangularis* as having two sets of paired inclusions in the phallus and two single, unpaired inclusions. One of the sets of paired inclusions and one of the unpaired inclusions seem to be elements of the phallotremal sclerite complex, including a central rod and ring structure and paired lateral sclerites. The other inclusions include a set of small, paired spines, common in members of the *kenyana* subgroup, and an unpaired apical spine-like sclerite. Gibon described the subspecies based mainly on differences in the phallic armature, including a larger unpaired spine than that found in the nominate form. We have used this as the basis for identifying the form illustrated here as *C.triangularisoccidentalis* (Fig. 22A–G), in addition to its relatively proximate geographic location. The nominotypical form was described from Ethiopia, on the other side of the African continent. The apical spine is somewhat unusual, very lightly sclerotized, and appearing somewhat feathered or striated. Its apical part appears wider in lateral view than in dorsal view, suggesting that it is somewhat blade-like. We are uncertain about the species or varietal status of this form, as distinct from the form described by Kimmins.

*Chimarratriangularisoccidentalis* is most diagnostically recognized by the overall shape of its inferior appendages, with its short tergum X and very short basal sensilla-bearing process. Among species treated here it is probably most similar in these regards to *C.pedaliotus* sp. nov., which is easily diagnosed by the very enlarged and compressed ventral apex of its phallobase.

###### Description.

***Adult.*** Overall color (in alcohol) pale yellowish brown. Head relatively elongate (length of postocular parietal sclerite nearly diameter of eye). Palps relatively short, maxillary palp with 1^st^ segment very short (length subequal to width), 2^nd^ segment short (~ 3× 1^st^), apex with small cluster of stiff setae, 3^rd^ segment relatively short (slightly longer than 2^nd^), 4^th^ segment short (shorter than 2^nd^), 5^th^ segment short (subequal to 2^nd^). Forewing length: male, 3.8–4.5 mm; female, 4.7–5.2 mm. Fore- and hind wings with forks I, II, III, and V present. Forewing with R_1_ somewhat sinuous, stem of Rs inflected at approximately midlength, with distinct node at inflection, extending into cell below, basal fork of discoidal cell enlarged, fork asymmetric, length of cell ~ 2× width, forks I and II slightly subsessile, *r* crossvein diagonal, intersecting discoidal cell at past midlength, just before fork I, *m* crossvein proximal to *s* and *r-m* crossveins, *s* pigmented (like wing), *r-m* and *m* crossveins hyaline and very faint, 2A with crossvein (apparently forked apically to 1A and 3A). Hind wing with R_1_ narrowly parallel to subcosta, forks I and II subsessile, fork III relatively terminal. Foreleg with apical tibial spur short; male with foretarsi modified, claws enlarged, outer claw twisted and asymmetric.

***Male genitalia*.** Segment VIII moderately elongate, sternum and tergum subequal in length. Segment IX, in lateral view, elongate, anterior margin greatly, subangularly produced in ventral ¼, dorsolaterally with short rounded apodeme, margin strongly concave between; tergum, in dorsal view, continuous between apodemes, forming concave excavation; posterior margin weakly produced below preanal appendages, extending more or less linearly to ventral process; ventral process prominent, posteriorly projecting, length greater than width at base, apex rounded. Segment IX, in dorsal or ventral views, with anteroventral margin deeply concave mesally. Lateral lobes of tergum X moderate in length, rounded apically, each with moderately elongate, posteriorly projecting, digitate, sensilla-bearing process on dorsal margin at approximately midlength; mesal lobe of tergum X membranous, somewhat shorter than the lateral lobes. Preanal appendages small, rounded, constricted basally. Inferior appendage, in lateral view, moderately elongate, narrow, nearly uniform in width, dorsally flexed near base, apex incurved and narrowed, in dorsal/caudal views, forming short, subtruncate, weakly bifid, projection; appendage, in dorsal or ventral views, strongly and uniformly curved, with short, rounded, setose, basomesal projection. Phallic apparatus with phallobase moderately elongate, tubular, with usual basodorsal expansion, dorsal margin somewhat projecting, but only weakly sclerotized, apicoventral margin slightly projecting, extending nearly straight; endotheca apparently without minute spines, but with two symmetrically positioned spines and an additional large, unpaired, lightly sclerotized, mesal spine, which is somewhat irregular, wider in lateral than in dorsal view; phallotremal sclerite complex composed of moderately elongate rod and ring structure, with narrow, paired, dorsolateral sclerites.

###### Distribution.

Ghana, Ivory Coast.

##### ﻿ Chimarra waensis

Taxon classificationAnimaliaTrichopteraPhilopotamidae

Gibon, 1985

D3930CE2-CC1F-58E7-917C-C21079DA1D26

Fig. 23A–E

Chimarra waensis Gibon, 1985: 26, figs 9–10.

###### Material examined.

Ghana – **Brong Ahafo Reg.** ● 1♀; Asubende, River Pru; 8°01'18"N, 1°01'58"W; 24 Nov. 1990; JS Amakye leg.; light trap; ZMBN. – **Northern Reg.** ● 1♀; Sabari, Oti River; 9°17'41"N, 0°14'43"E; 22–24 Nov. 1991; JS Amakye leg.; Malaise trap; ZMBN. – **Upper East Reg.** ● 1♀; Nangodi, Nangodi Bridge; 10°51'48"N, 0°39'36"W; 26 June 1993; JS Amakye leg.; light trap; ZMBN. – **Volta Reg.** ● 1♀; Wli, Agumatsa waterfall, station # 12; 7°07'29"N, 0°35'31"E; 16 Nov. 1993; J Kjærandsen leg.; light trap; ZMBN. – **Western Reg.** ● 2♀♀; Ankasa Game Production Reserve; 5°15'N, 2°37'W; 6–12 Dec. 1993; T Andersen & J Kjærandsen leg.; Malaise trap; ZMBN ● 1♀; same collection data as for preceding except 9 Dec. 1993; J Kjærandsen leg.; light trap; UMSP ● 1♂; same collection data as for preceding except 31 Mar. 1993; J Kjærandsen leg.; light trap; UMSP ● 1♀; same collection data as for preceding; ZMBN ● 7♀♀; same collection data as for preceding except 8–10 Dec. 1993; T Andersen & J Kjærandsen leg.; ZMBN.

###### Diagnosis.

**Figure 23.**
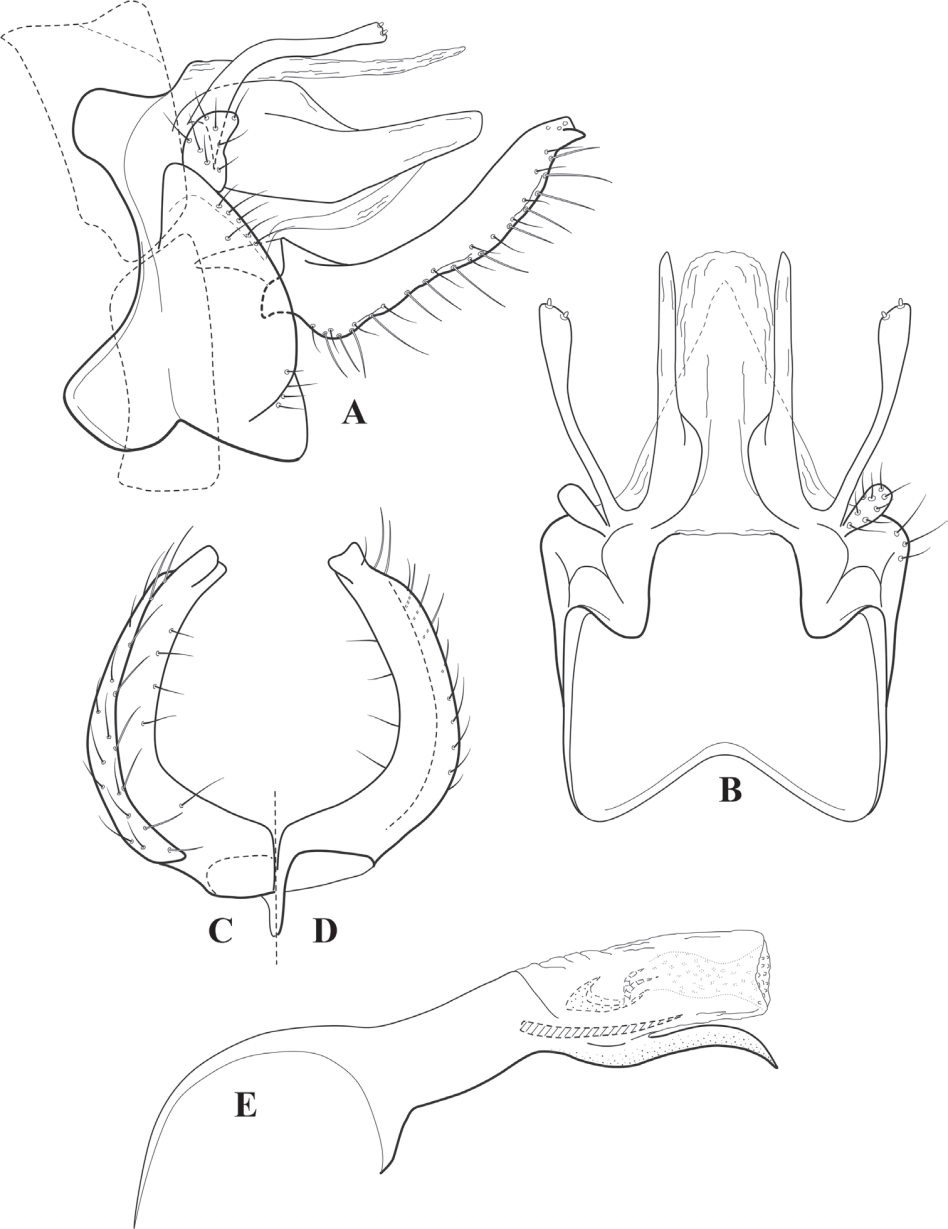
*Chimarrawaensis* Gibon, ♂ genitalia **A** lateral **B** dorsal, segments IX and X **C** inferior appendage, ventral **D** inferior appendage, dorsal **E** phallus, lateral.

Characters, in combination, that confirm the identification and can be used to distinguish *C.waensis* from other species in the subgroup include: length and position of digitate process of tergum X; general shape and length of inferior appendage; subtriangular shape of ventral process of tergum X; the single, moderately elongate phallic spine; and the curved, projecting apex of the phallobase. The anterior margin of segment IX, in the original illustration of *C.waensis* (Gibon 1985: fig. 10), is less sinuate than in our illustration. Particularly, the prominent dorsal apodeme is not featured. This is more likely a deficit in the illustration than a genuine difference, since the anterior contour of the segment, as illustrated in Fig. 23A, is not characteristic of any species of the *kenyana* subgroup. We consider the matching features sufficient to justify the use of the name *C.waensis* to identify our specimen.

Both *C.baculifera* Marlier and *C.camerunensis* Marlier are also very similar to *C.waensis*, particularly in the general shape of the inferior appendages, which are elongate and narrow, with a characteristic subtruncate apex. All of these species also have elongated, curved, sensilla-bearing processes on the lateral lobes of tergum X, and a similar, basally broad, subtriangular ventral process on segment IX. We initially considered synonymizing all of these species. Restraint in doing so was based on the very acutely angled anteroventral margin of segment IX in the original illustration of *C.baculifera*, and the very elongate phallic spine featured in the original illustration of *C.camerunensis*. Since these kinds of differences are not usually attributable to intraspecific variation, a more critical evaluation, provided by comparison of holotype specimens, ideally in the context of a formal revision of the entire subgroup, should probably precede any synonymy.

###### Redescription.

***Adult.*** Overall color (in alcohol) pale yellowish brown. Head elongate (postocular parietal sclerite slightly shorter than diameter of eye). Palps elongate; maxillary palp with 1^st^ segment very short (length subequal to width), 2^nd^ segment elongate (slightly longer than 3^rd^), apex with cluster of stiff setae, 3^rd^ segment moderately elongate (normal), 4^th^ segment short (~ 2× 1^st^), 5^th^ segment elongate (subequal to 3^rd^). Forewing length: male, 5.7 mm; female, 4.5–5.0 mm. Fore- and hind wings with forks I, II, III, and V present. Forewing with R_1_ distinctly sinuous, stem of Rs inflected at past midlength, with node extending into cell below, discoidal cell short, length slightly greater than width, basal fork not enlarged, forks I and II sessile, *r* crossvein diagonal, intersecting discoidal cell at approximately midlength, *r-m* crossvein of forewing diagonal, slightly proximal to *s*, *m* crossvein very faint, proximal to *r-m*, very near basal fork of M, 2A with crossvein (apparently forked apically to 1A and 3A). Hind wing with R_1_ fused to subcosta basally, both veins intersecting wing margin, discoidal cell short, forks I and II elongate, sessile. Foreleg with apical tibial spur short; male with foretarsi unmodified, claws small and symmetrical.

***Male genitalia*.** Segment VIII short, sternum and tergum subequal in length. Segment IX, in lateral view, relatively short, anterior margin distinctly, subangularly, produced in ventral ¼, dorsolaterally with very prominent, broadly rounded apodeme, nearly as projecting as ventral production, margin concave between; tergum, in dorsal view, very short and narrowly sclerotized, but continuous dorsally, or nearly so, forming excavation between apodemes; posterior margin obliquely and somewhat convexly widened from preanal appendage to ventral process; ventral process prominent, subtriangular, wide basally, only weakly projecting. Segment IX, in dorsal or ventral views, with anteroventral margin moderately, concavely excavated mesally. Lateral lobes of tergum X elongate, subtruncately rounded apically, somewhat dorsally produced in basal half, basodorsally with elongate, posteriorly oriented, digitate, sensilla-bearing process; lateral lobes, in dorsal view, subparallel; mesal lobe of tergum X membranous, approximately as long as lateral lobes. Preanal appendages short, constricted basally, knob-like. Inferior appendage, in lateral view, elongate, narrow, nearly uniform in width, distinctly dorsally flexed near base, apex slightly narrowed, forming subtruncate, weakly notched or bifid process; appendage, in dorsal or ventral views, more or less uniformly mesally curved, curvature moderate. Phallic apparatus with phallobase relatively short and tubular, with usual basodorsal expansion, apicoventral margin distinctly projecting and deflexed, apex acute and even more strongly ventrally curved; endotheca with single, moderately elongate spine; phallotremal sclerite complex composed of short rod and ring structure, with small, indistinct apicolateral sclerites.

###### Distribution.

Ghana, Ivory Coast.

#### The *leta* subgroup

**Included species.***Chimarraamakyei* sp. nov.; and *Chimarraleta* Mosely, 1936.

Characters tentatively used to define the group include an elongate, narrow tergum X, with preanal appendages flattened and fused basally, sensilla of tergum X on a rounded mesally directed process, nearly linear arrangement of the *s*, *r-m*, and *m* crossveins of the forewing, and lack of a basal inflection of the inferior appendages. The latter two characters are probably plesiomorphic and also occur in the *cara* subgroup; thus, they do not necessarily indicate a relationship between the two subgroups. They are, however, unusual characters within the *marginata* Group as a whole. Like most members of the *fallax* subgroup, the inferior appendages are mounted relatively high above the ventral process of segment IX, which, however, is short, rather than elongate, and tergum X has a pair of ventral sclerotized periphallic processes (or detached ventromesal lobes of tergum X, lacking sensilla). These similarities are likely convergent. Based on literature descriptions and illustrations, only *C.leta* and the following new species can be definitively placed in this subgroup.

##### ﻿ Chimarra amakyei sp. nov.

Taxon classificationAnimaliaTrichopteraPhilopotamidae

701827E8-CB35-5A25-8418-943E8DC98B0D

http://zoobank.org/7E3AB7DA-C910-461E-9057-754E790215CE

Fig. 24A–E

###### Type material.

***Holotype*.** Ghana – **Volta Reg.** ● ♂ (in alcohol); Wli, Agumatsa waterfall, station # 3; 7°07'29"N, 0°35'31"E; 10 Mar. 1993; JS Amakye & J Kjærandsen leg.; sweep net; UMSP 000550018. ***Paratypes*.** Ghana – **Volta Reg.** ● 3♂♂; same data as for holotype except station # 2^A^; 8–11 Mar. 1993; Malaise trap; ZMBN.

###### Additional material.

Ghana – **Volta Reg.** ● 2♀♀; Wli, Agumatsa waterfall, station # 1^A^; 7°07'29"N, 0°35'31"E; 12–21 Nov. 1993, J. Kjærandsen leg.; Malaise trap; ZMBN ● 1♀; same collection data as for previous except station # 2^B^; 5–14 Mar. 1993; JS Amakye & J. Kjærandsen leg.; ZMBN ● 2♀♀; same collection data as for previous except station # 5^A^; 10–13 Mar. 1993; ZMBN ● 1♀; same collection data as for previous except station # 5^D^; 9–12 Mar. 1993; ZMBN ● 1♀; same collection data as for previous except station # 5C; 12–15 Mar. 1993, J Kjærandsen & JS Amakye leg.; UMSP.

**Figure 24.**
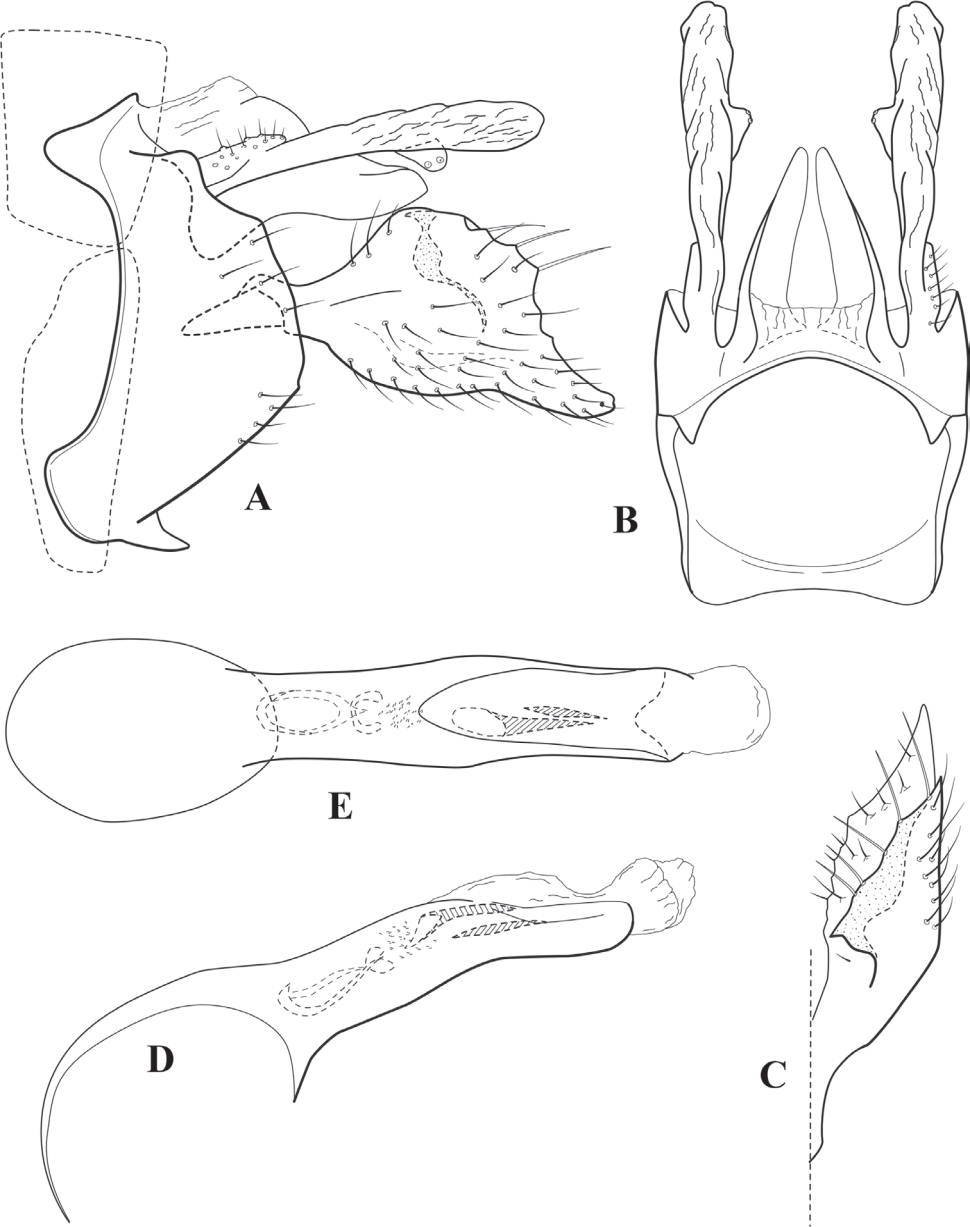
*Chimarraamakyei* sp. nov., ♂ genitalia **A** lateral **B** dorsal, segments IX and X **C** inferior appendage, dorsal **D** phallus, lateral **E** phallus, dorsal.

###### Diagnosis.

*Chimarraamakyei* sp. nov. is closely related to *Chimarraleta* Mosely. Like that species it has an elongate, narrow tergum X, with preanal appendages flattened and fused basally. It also has inferior appendages projecting approximately midlaterally, above a small basally located ventral process. It differs diagnostically in that the apices of inferior appendages are tapering and acute, rather than subtruncate.

###### Description.

***Adult.*** Overall color (in alcohol) yellowish brown; vertex of head dark brown, setal warts very pale. Head relatively short (postocular parietal sclerite short). Palps moderately elongate; maxillary palp with 1^st^ segment very short (approximately as long as wide), 2^nd^ segment relatively short (distinctly shorter than 3^rd^), apex with small cluster of stiff setae, 3^rd^ segment elongate, 4^th^ segment very short (shorter than 2^nd^), 5^th^ segment elongate (slightly longer than 3^rd^). Forewing length: male, 4.8–5.3 mm; female, 4.5–5.3 mm. Fore- and hind wings with forks I, II, III, and V present. Forewing with R_1_ sinuous, stem of Rs distinctly inflected at past midlength (without distinct node at inflection), basal fork of discoidal cell moderately enlarged, length of cell slightly greater than 2× width, forks I and II sessile, *r* crossvein strongly diagonal, intersecting discoidal cell at just past midlength, *s*, *r-m*, and *m* crossveins linear, s crossvein pigmented (like wing), *r-m* and *m* crossveins hyaline (*m* very indistinct), 2A with crossvein (apparently forked apically to 1A and 3A). Hind wing with R_1_ reduced, narrowly parallel to subcosta, fork I sessile, fork II subsessile, fork III distal and relatively wide, anal loop small. Forelegs with apical tibial spur short; male without enlarged tarsal claws, apical segments of tarsi narrow, claws very small and symmetrical.

***Male genitalia*.** Segment VIII with sternum very short, tergum moderately expanded dorsally (~ 2× length of sternum at base). Segment IX, in lateral view, relatively short, anterior margin concave, dorsally with broadly rounded apodeme, anteroventrally with short angular projection at approximately basal ¼; posterior margin moderately produced midlaterally, basoventrally with very short, subtriangular, posteriorly projected ventral process, segment expanded and sloping dorsal to process, with inferior appendages inserted high above process, approximately midlaterally; as viewed dorsally, with tergum very narrow, but continuous, sternum subtruncate, slightly concave mesally. Lateral lobes of tergum X elongate, rugose and club-like, with small rounded, mesally directed process bearing two sensilla at past midlength; dorsum of tergum X short, membranous, continuous with paired, sclerotized, apically rounded, periphallic processes surrounding phallic apparatus laterally and ventrally. Preanal appendages narrow and flattened, fused basally to lateral lobes. Inferior appendage without pronounced basal inflection, appendage narrow basally, dorsal margin with rounded expansion, tapering to acute, projecting apex; base of dorsal expansion with very short, sclerotized, mesally curved projection, continuous on mesal surface as sclerotized ridge. Phallic apparatus with relatively small, tubular phallobase with usual basodorsal expansion, apicodorsal margin with deep membranous invagination, ventral margin projecting, but without apicomesal projection; endotheca with two very small spines; phallotremal sclerite complex composed of reclinate, ring-like structure, with short rounded apicolateral projections.

###### Etymology.

*Chimarraamakyei*, named for Joseph S. Amakye, who helped collect much of the material represented in this paper, in recognition of his efforts.

#### The *mazumbai* subgroup

**Included species.***Chimarramazumbai* sp. nov.; *Chimarrausambara* sp. nov.; and *C.wliensis* sp. nov.

Three species are assigned to this new group, the two from Tanzania very evidently closely related and the third from Ghana more speculatively associated. All of the species have scabrous lobes associated to terga IX or X, and relatively short phalli with a prominent sclerotized ventral apex. The endotheca is relatively simple, short and untextured (without small spines). A single short spine is present in the endotheca, as well as a short phallotremal sclerite complex, composed of a short rod and ring structure. The ventral process of segment IX is also very short. Superficially, the species look very much like species in the *georgensis* Group, but venational characters, including the arrangement of the chord and the anal veins of the forewing, are typical of the *marginata* Group. It seems likely that the species included here represent a relatively basal lineage within this group.

##### ﻿ Chimarra mazumbai sp. nov.

Taxon classificationAnimaliaTrichopteraPhilopotamidae

4A8C0F4E-3BD9-5F3A-9862-D5E7989BA912

http://zoobank.org/A021CB84-FC3E-437E-A93E-14ECCCDEE586

Fig. 25A–D

###### Type material.

***Holotype*.** Tanzania – **Tanga Reg.** ● ♂ (in alcohol); West Usambara Mts., Mazumbai, Kaputu Stream; 4°48'S, 38°30'E; 29–30 Nov. 1990; T Andersen leg.; Malaise trap; UMSP 000550020. ***Paratypes*.** Tanzania – **Tanga Reg.** ● 1♂; same data as for holotype except 14–20 Nov. 1990; UMSP ● 21♂♂; same data as for holotype except 30 Oct. 1990–12 Feb. 1991; ZMBN.

###### Diagnosis.

*Chimarramazumbai* is very similar to *C.usambara* sp. nov. Both species have a pair of upturned, digitate processes on a short tergum X, and somewhat similar inferior appendages, as well as a relatively short segment IX, with a very small ventral process. *Chimarramazumbai* can be distinguished by the shape of its inferior appendage, which has its dorsal process shorter, and also by having the digitate processes of tergum X less closely apposed.

**Figure 25.**
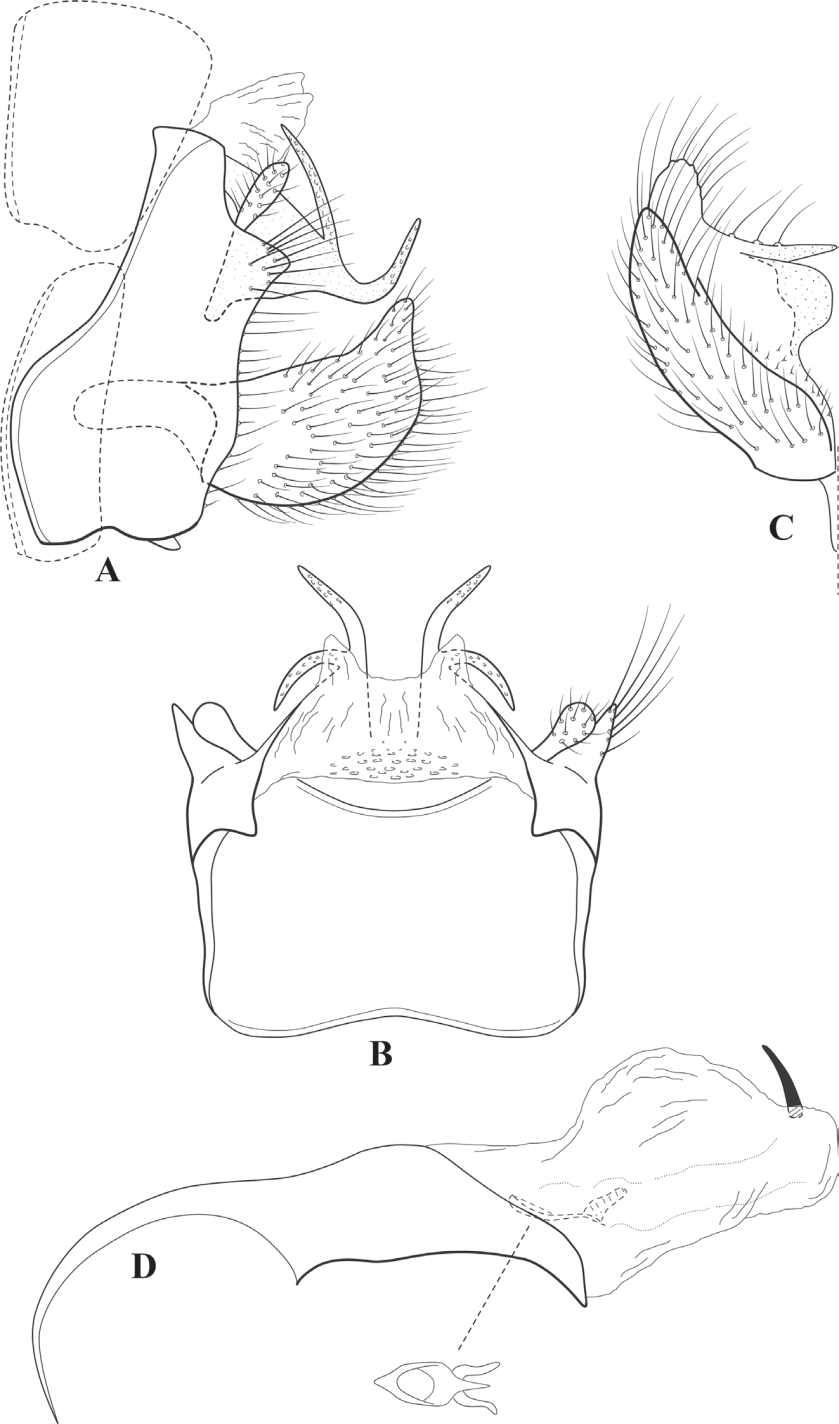
*Chimarramazumbai* sp. nov., ♂ genitalia **A** lateral **B** dorsal, segments IX and X **C** inferior appendage, ventral **D** phallus, lateral, with dorsal detail of phallotremal sclerite complex.

###### Description.

***Adult.*** Overall color (in alcohol) dark brown. Head moderately elongate (postocular parietal sclerite ~1/2 diameter of eye). Palps elongate, maxillary palp with 1^st^ segment short (length slightly greater than width), 2^nd^ segment very elongate, with approximately a dozen elongate apical setae, 3^rd^ segment elongate (subequal to 2^nd^), 4^th^ segment short (< 1/2 length of 3^rd^), 5^th^ segment elongate (slightly longer than 3^rd^). Forewing length: male, 6.5–8.0 mm. Fore- and hind wings with forks I, II, III, and V present. Forewing with R_1_ not, or only very weakly, sinuous, stem of Rs with relatively weak inflection in apical half, with node at inflection, extending into cell below, basal fork of discoidal cell slightly enlarged, fork nearly symmetric, discoidal cell elongate, length ~ 2 1/2× width, forks I and II distinctly subsessile, *r* crossvein intersecting discoidal cell at base of fork I, *r-m* crossvein continuous with *s*, *m* crossvein proximal to *s* and *r-m* crossveins, approximately midway between basal fork of M and *r-m* crossvein, *s* pigmented (like wing), *r-m* and *m* crossveins hyaline, very indistinct, 2A with crossvein (apparently forked apically to 1A and 3A). Hind wing with R_1_ fused to subcosta basally, both veins intersecting wing margin apically, forks I and II subsessile. Foreleg with apical tibial spur very short; male with foretarsi unmodified, claws small and symmetrical.

***Male genitalia*.** Segment VIII short ventrally, tergum ~ 2× as long dorsally. Segment IX, in lateral view, relatively short, anterior margin relatively weakly, sinuously, produced in ventral half, dorsolaterally with apodeme very small, almost absent; tergum, in dorsal view, obsolete between apodemes; posterior margin short dorsally, weakly produced below preanal appendages, nearly linear to ventral margin, ventral margin with very minute, short, ventrally projecting, process posteriorly. Segment IX, in dorsal or ventral views, with anteroventral margin subtruncate. Lateral lobes of tergum X very short, with pair of very narrow, digitate, dorsally projecting, apically acute processes, one apical and one at midlength, sensilla not apparent; mesal lobe of tergum X membranous, short, hardly projecting beyond base of lateral lobes. Preanal appendages short, rounded, knob-like, distinctly constricted basally. Inferior appendage, in lateral view, short, densely setose, strongly rounded basally, dorsally with short, rounded, dorsally flexed apex; in ventral view, with closely associated, narrow, acute, and rounded projections, visible on dorsomesal margin. Phallic apparatus with phallobase very short and tubular, with usual basodorsal expansion, apicoventral margin forming distinct, ventrally curved, projection; endotheca membranous and simple in structure, without minute spines, but with a single short apicomesal spine; phallotremal sclerite complex composed of very short rod and ring structure, with pair of small apical sclerites.

###### Etymology.

*Chimarramazumbai*, name used as a noun in apposition, for the name of the scenic type locality in the Usambara Mountains where the species was collected.

##### ﻿ Chimarra usambara sp. nov.

Taxon classificationAnimaliaTrichopteraPhilopotamidae

CDCCE086-CF40-542E-ADA2-D089366DD417

http://zoobank.org/6D9524AF-CC86-460C-B9E1-011AE3246730

Fig. 26A–E

###### Type material.

***Holotype*.** Tanzania – **Tanga Reg.** ● ♂ (in alcohol); East Usambara Mts, Sigi River, Amani; 21 Feb. 1959; 2.500 ft; MT Gillies leg.; INHSTrichoptera 50335. ***Paratypes*.** Tanzania – **Tanga Reg.** ● 1♀; same data as for holotype; INHS $ 1♂; Amani; 16 Nov. 1959; MT Gillies leg.; INHS.

###### Diagnosis.

*Chimarrausambara* is very similar to *C.mazumbai* sp. nov. and, like that species, has a short tergum X with a pair of upturned, spine-like processes. It is easily distinguished from *C.mazumbai* in that the spine-like processes of the tergum are more closely adpressed, and by the shape of its inferior appendage, which has a more distinctly defined and upturned, thumb-like dorsal process.

**Figure 26.**
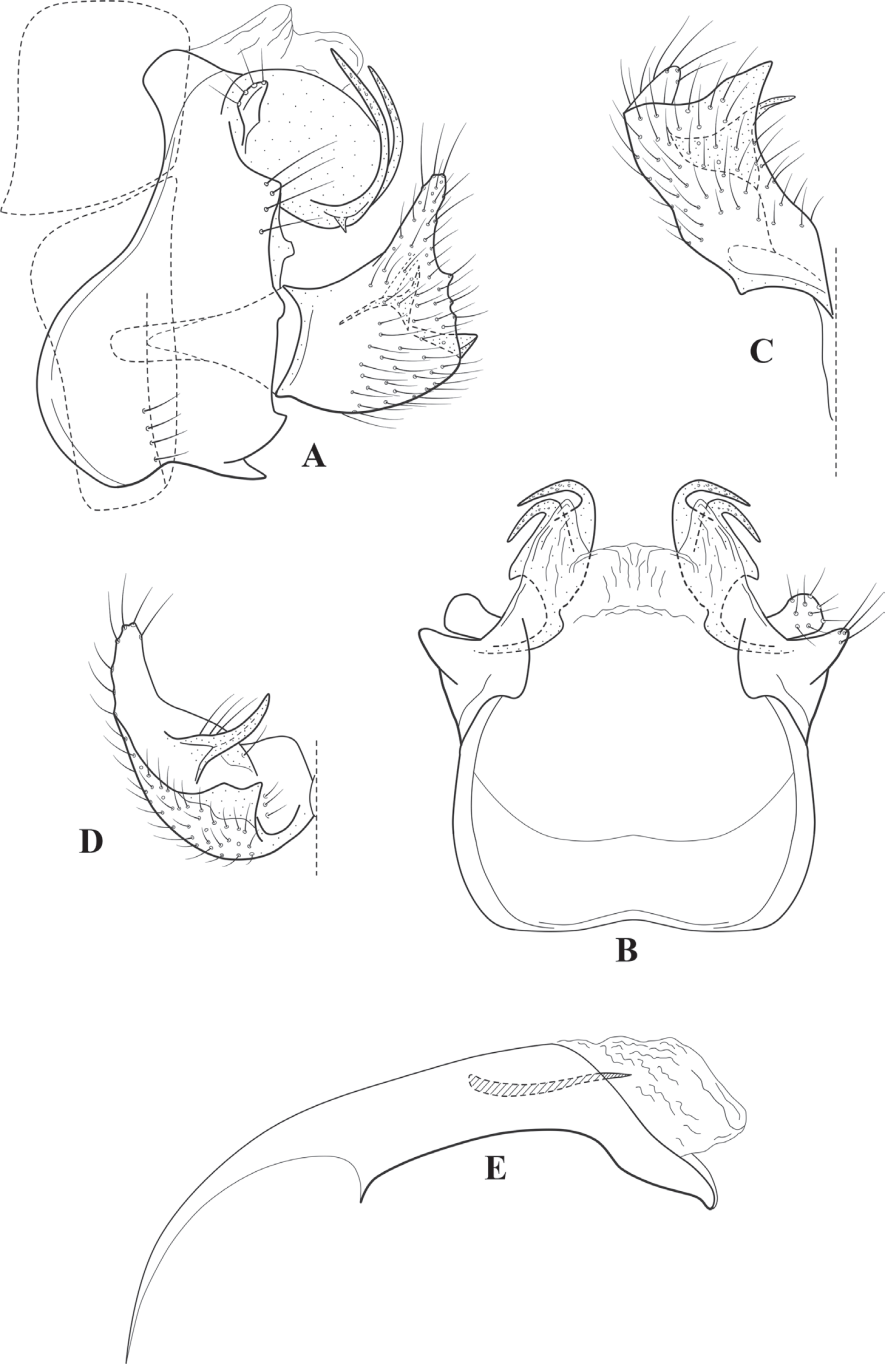
*Chimarrausambara* sp. nov., ♂ genitalia **A** lateral **B** dorsal, segments IX and X **C** inferior appendage, ventral **D** inferior appendage, caudal **E** phallus, lateral.

###### Description.

***Adult.*** Overall color (in alcohol) dark brown. Head moderately elongate (postocular parietal sclerite ~ 1/2 diameter of eye). Palps elongate, maxillary palp with 1^st^ segment short (length subequal to width), 2^nd^ segment very elongate, with approximately a dozen elongate apical setae, 3^rd^ segment moderately elongate (shorter than 2^nd^), 4^th^ segment short, 5^th^ segment elongate (subequal to 2^nd^). Forewing length: male, 4.7 mm; female, 5.0 mm. Fore- and hind wings with forks I, II, III, and V present. Forewing with R_1_ not, or only very weakly, sinuous, stem of Rs with relatively weak inflection in apical half, with node at inflection, extending into cell below, basal fork of discoidal cell enlarged, fork very asymmetric, discoidal cell with length ~ 2× its width, forks I and II slightly subsessile, *r* crossvein diagonal, intersecting discoidal cell at past midlength, just before fork I, *r-m* crossvein continuous with *s*, *m* crossvein proximal to *s* and *r-m* crossveins, approximately midway between basal fork of M and *r-m* crossvein, *s* pigmented (like wing), *r-m* and *m* crossveins hyaline, very indistinct, 2A with crossvein (apparently forked apically to 1A and 3A). Hind wing with R_1_ narrowly parallel to subcosta, forks I and II subsessile. Foreleg with apical tibial spur apparently absent; male with foretarsi unmodified, claws small and symmetrical.

***Male genitalia*.** Segment VIII short ventrally, tergum somewhat wider dorsally. Segment IX, in lateral view, relatively short, anterior margin relatively weakly, sinuously produced in ventral half, dorsolaterally with distinct rounded apodeme; tergum, in dorsal view, obsolete between apodemes; posterior margin short dorsally, weakly produced below preanal appendages, nearly linear to ventral margin, ventral margin with short, small, posteriorly projecting, ventral process. Segment IX, in dorsal or ventral views, with anteroventral margin subtruncate. Lateral lobes of tergum X very short and rounded, with pair of closely apposed and very narrow, digitate, dorsally projecting, recurved, apically acute processes from apicoventral margin, projections slightly scabrous, sensilla not apparent; mesal lobe of tergum X membranous, short, hardly projecting beyond base of lateral lobes. Preanal appendages short, rounded, knob-like, distinctly constricted basally. Inferior appendage, in lateral view, short, densely setose, strongly rounded basally, dorsally with thumb-like, dorsally flexed projection; in ventral view, with short, acute, mesally curved, apicoventral projection, only indistinctly visible in lateral view; in caudal view, with narrow spine-like projection visible on mesal surface. Phallic apparatus with phallobase very short and tubular, with usual basodorsal expansion, apicoventral margin forming distinct, ventrally curved, projection; endotheca membranous and simple in structure, without spines; phallotremal sclerite complex composed of relatively short, simple, rod and ring structure, with associated sclerites absent or not apparent.

###### Etymology.

*Chimarrausambara*, name used as an adjective, after the East Usambara Mountains of Tanzania, in which this species was collected.

##### ﻿ Chimarra wliensis sp. nov.

Taxon classificationAnimaliaTrichopteraPhilopotamidae

BD73B837-AFC7-54A1-8F74-862E433A573F

http://zoobank.org/D21E371E-DA8A-4AED-9A4E-7FBDA69776FC

Fig. 27A–E

###### Type material.

***Holotype*.** Ghana – **Volta Reg.** ● ♂ (in alcohol); Wli, Agumatsa waterfall, station # 2^A^; 7°07'29"N, 0°35'31"E; 8–11 Mar. 1993; JS Amakye & J Kjærandsen leg.; Malaise trap; UMSP 000550021. ***Paratypes*.** Ghana– **Volta Reg.** ● 1♂; same data as for holotype except station # 1^A^; 5–14 Mar. 1993; ZMBN ● 1♂; same data as for holotype except station # 3; 11–20 Nov. 1993; J Kjærandsen leg.; ZMBN. – **Western Reg.** ● 1♂; Ankasa Game Production Reserve; 5°15'N, 2°37'W; 12 Dec. 1993, T Andersen & J Kjærandsen leg.; sweep net; ZMBN.

###### Additional material.

Ghana – **Volta Reg.** ● 5♀♀; Wli, Agumatsa waterfall, station # 2^A^; 7°07'29"N, 0°35'31"E; 5–14 Mar. 1993; JS Amakye & J Kjærandsen leg.; Malaise trap; ZMBN ● 1♀; same collection data as for preceding except 8–11 Mar. 1993; UMSP ● 1♀; same collection data as for preceding except station # 2^B^; ZMBN ● 1♀; same collection data as for preceding except station # 3; 17 Nov. 1993; J Kjærandsen leg.; light trap; ZMBN.

**Figure 27.**
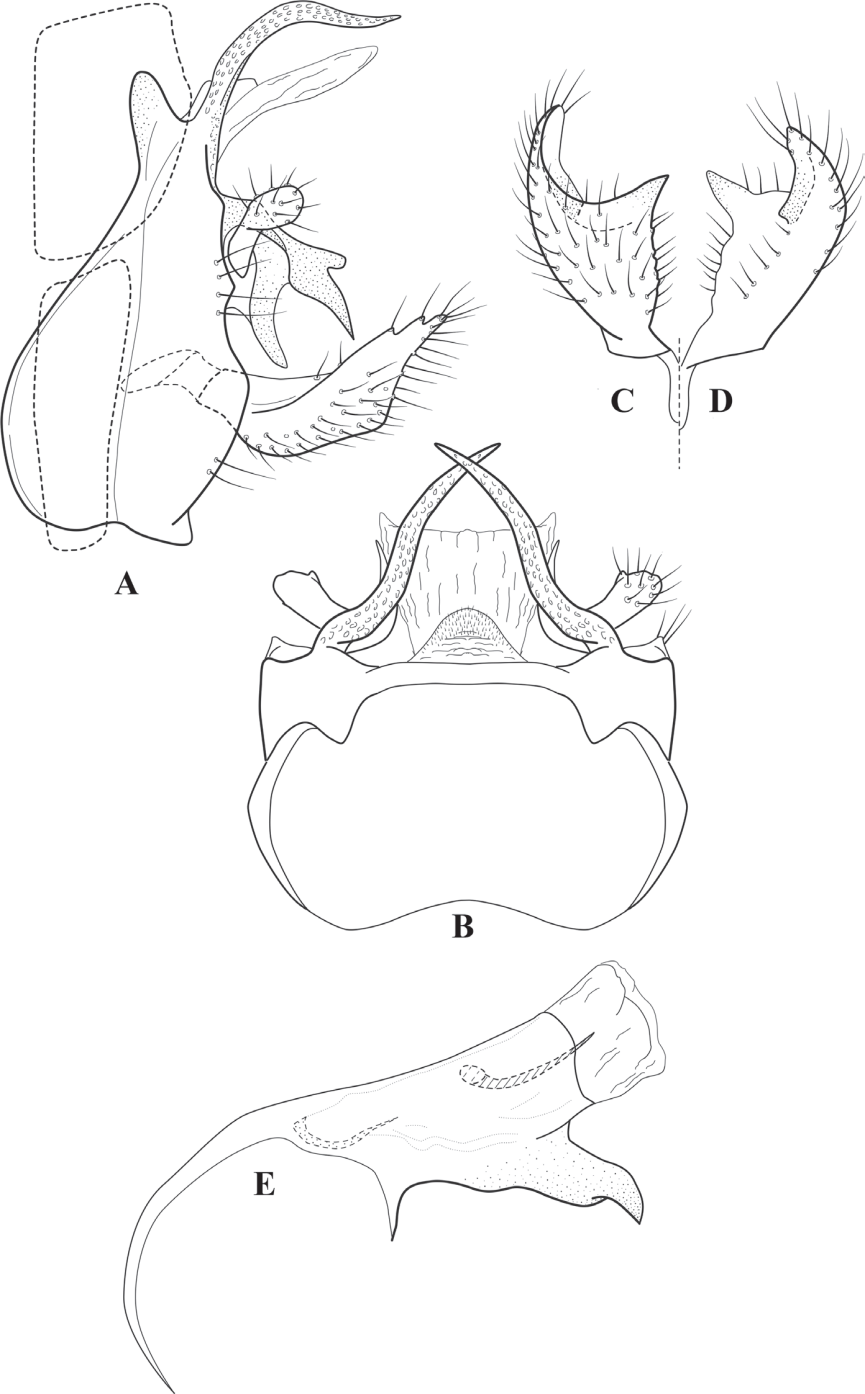
*Chimarrawliensis* sp. nov., ♂ genitalia **A** lateral **B** dorsal, segments IX and X **C** inferior appendage, ventral **D** inferior appendage, dorsal **E** phallus, lateral.

###### Diagnosis.

*Chimarrawliensis* is a very distinctive species, easily recognized by the curved, spine-like, and scabrous lateral lobes of tergum X and the shape of its inferior appendages, in addition to it venational attributes.

Superficial similarities would suggest a relationship to species in the *georgensis* Group, because of the scabrous dorsolateral processes on segment IX, as in the *evoluta* subgroup, and the sclerotized and divided lateral lobes of tergum X, as in the *georgensis* subgroup, as well as its rather simple and short phallobase, with a produced ventral apex. However, venational characters place this species within the *marginata* Group, since the anal veins have a distinct crossvein (2A apparently forked apically), the Rs vein of the forewing is distinctly curved, with the *s* crossvein pigmented (not hyaline), and the *m* crossvein is distinctly proximal to the *s* and *r-m* crossveins. In overall morphology the species is thus distinctive. It is conceivably related to *C.berghei* (Marlier), whose overall description makes it difficult to place; similarities to *C.wliensis* include, particularly, the arching dorsolateral lobes of tergum X; however, *C.berghei* differs significantly in the shape of its inferior appendages and it is possible that it is not very closely related.

###### Description.

***Adult.*** Overall color (in alcohol) medium brown, head and thorax not paler than body. Head elongate (postocular parietal sclerite nearly as long as diameter of eye). Palps elongate; maxillary palp with 1^st^ segment very short (approximately as long as wide), 2^nd^ segment elongate (subequal to 3^rd^), apex with numerous elongate, stiff setae, 3^rd^ segment elongate, 4^th^ segment short (~ 1/3 length of 3^rd^), 5^th^ segment elongate and narrow (subequal to 3^rd^). Forewing length: male, 5.7–6.5 mm; female, 6.5–7.5mm. Fore- and hind wings with forks I, II, III, and V present. Forewing with R_1_ somewhat curved, stem of Rs curved, bowed outward, without sclerotized node in cell below, basal fork of discoidal cell slightly enlarged, nearly evenly forked, length of cell ~ 2× width, forks I and II sessile, *r* crossvein diagonal, intersecting discoidal cell before fork, *s* crossvein pigmented, *r-m* and *m* hyaline, *s* and *r-m* crossveins continuous, *m* crossvein distinctly proximal; 2A with crossvein (apparently forked apically to 1A and 3A). Hind wing with R_1_ obsolete (or fused to subcosta), forks I and II subsessile. Forelegs with apical tibial spur very short; male with apical segments of foreleg small and thread-like, not enlarged, tarsal claws symmetrical.

***Male genitalia*.** Segment VIII short, tergum longer than sternum. Segment IX short, anterior margin expanded and rounded in ventral half, segment very short dorsally, anterodorsal margin with distinct rounded apodemes, posterodorsal margin with elongate, scabrous, posteriorly-curved, spine-like lateral processes, ventral process very short, subtriangular, more or less ventrally oriented, inferior appendages inserted distinctly above ventral margin of segment; as viewed dorsally, with tergum very narrow, but continuous, sternum short, broad, weakly concave mesally, scabrous dorsolateral processes of segment mesally curved, meeting mesally. Tergum X with mesal lobe membranous and with textured region at at base, lateral lobes (or periphalic processes?) strongly sclerotized and ventrally curved, divided apically into acute lobes, sensilla of lobes absent (or not evident). Preanal appendages short, knob-like, inserted membranously (not fused to segments IX or X). Inferior appendage with moderate basal inflection; as viewed laterally, more or less narrow, moderately elongate, apex acute; as viewed ventrally, with prominent, acute apicomesal projection (thus, ventral and apical projections subequal and separated by crescentic margin); mesal margin with short cusp, continuous with apical projection. Phallic apparatus with phallobase short and strongly sclerotized, with usual basodorsal expansion, apicoventral margin of phallobase projecting, sclerotized, acute, distinctly ventrally curved; endotheca apparently short, membranous, with single short spine; phallotremal sclerite complex composed of short rod and ring structure.

###### Etymology.

*Chimarrawliensis*, used as an adjective, meaning “from Wli”, for the site where the holotype of this species was collected.

#### The *minima* subgroup

**Included species.***Chimarraambaja* Mosely, 1939; *C.angolensis* Marlier, 1965; *C.antsymeloka* Gibon, 2015; *C.assambae* Gibon, 2015; *C.bertrandi* Scott, 1974; *C.callasae* Gibon, 1982; *C.cereris* Barnard, 1934; *C.cognata* Kimmins, 1957; *C.intexta* Mosely, 1931; *C.koualaeensis* Johanson & Mary, 2009; *C.loffae* Gibon, 2015; *C.lufirae* Jacquemart, 1961; *C.minima* Ulmer, 1907; *C.prodhoni* Gibon, 1985; *C.sanagae* Gibon, 2015; *C.sassandrae* Gibon, 1982; *C.toubaensis* Gibon, 1985; and *C.vulgaris* Gibon, 2015.

The *minima* subgroup was treated in a recent revision by Gibon (2015), and the reader is referred to that work for a comparative treatment and descriptions of the species. The only additional species assigned to the group is *C.koualaeensis* Johanson & Mary, 2009, due to its close morphological similarity. Four species of the subgroup were collected from Ghana and their distribution records are listed below. Illustrations of the species are included for comprehensive reasons.

##### ﻿ Chimarra callasae

Taxon classificationAnimaliaTrichopteraPhilopotamidae

Gibon, 1982

FBFB4B85-DEE0-5EC4-8BF5-96AA9581D339

Fig. 28A–G

Chimarra callasae Gibon, 1982: 75–76, figs 3, 12–15.
Chimarra callasae Gibon: Gibon 2015: 335, 338, 346, fig. 3A–B (distribution [table, map]: Mali, Guinea, Sierra-Leone).

###### Material examined.

Ghana – **Northern Reg.** ● 1♂1♀; Bamboi, Black Volta; 8°08'50"N, 2°02'40"W; 25 Apr. 1991; T Andersen & JS Amakye leg.; light trap; UMSP.

###### Diagnosis.

*Chimarracallasae* is most readily diagnosed from other species in the subgroup by the short and apically strongly out-turned and acute lateral lobes of tergum X, by the shape of the two phallic spines, which are short, symmetrical, and have their apices more or less bird-head shaped, and also by the general shape of the inferior appendages.

**Figure 28.**
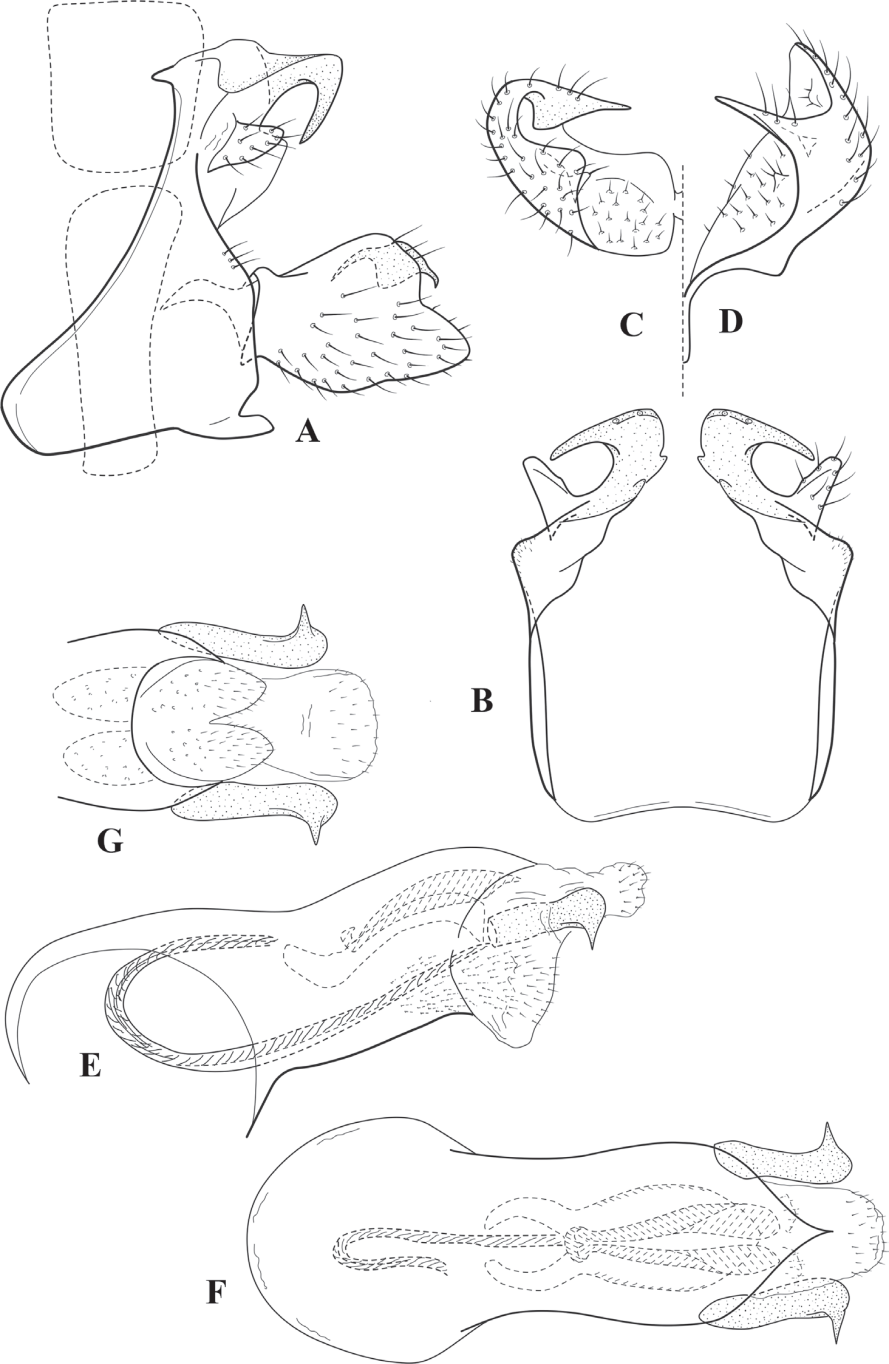
*Chimarracallasae* Gibon, ♂ genitalia **A** lateral **B** dorsal, segments IX and X **C** inferior appendage, caudal **D** inferior appendage, dorsal **E** phallus, lateral **F** phallus, dorsal **G** phallus apex, ventral

###### Distribution.

Ghana, Guinea, Mali, Sierra-Leone.

##### ﻿ Chimarra intexta

Taxon classificationAnimaliaTrichopteraPhilopotamidae

Mosely, 1931

24F8A36C-07C7-54FB-8AA2-328F94F91D42

Fig. 29A–F

Chimarrha intexta Mosely, 1931: 546–547, figs 6–9.
Chimarra intexta Mosely: Kimmins 1958: 359, 361, fig. 2 (distribution: Sierra Leone); Fischer 1961: 60; Gibon 1985: 25 (distribution: Ivory Coast); Gibon 2015: 335, 346 (distribution [table, map]: Sierra Leone, Ivory Coast, Guinea).

###### Material examined.

Ghana – **Central Reg.** ● 1♂; Kakum Forest Reserve; 5°21'N, 1°22'W; 8 Nov. 1994; T Andersen leg.; light trap; ZMBN ● 1♀; same collection data as for preceding; UMSP. – **Greater Accra Reg.** ● 1♂; Legon, Botanical Garden; 5°51'55"N, 0°11'15"W; 19 Nov. 1994; T Andersen leg.; light trap; ZMBN. – **Western Reg.** ● 1♂; Ankasa Game Production Reserve; 5°15'N, 2°37'W; 31 Mar. 1993; J Kjærandsen leg.; light trap; UMSP ● 1♂2♀♀; same collection data as for preceding except 5 Dec. 1993; T Andersen & J Kjærandsen leg.; ZMBN.

**Figure 29.**
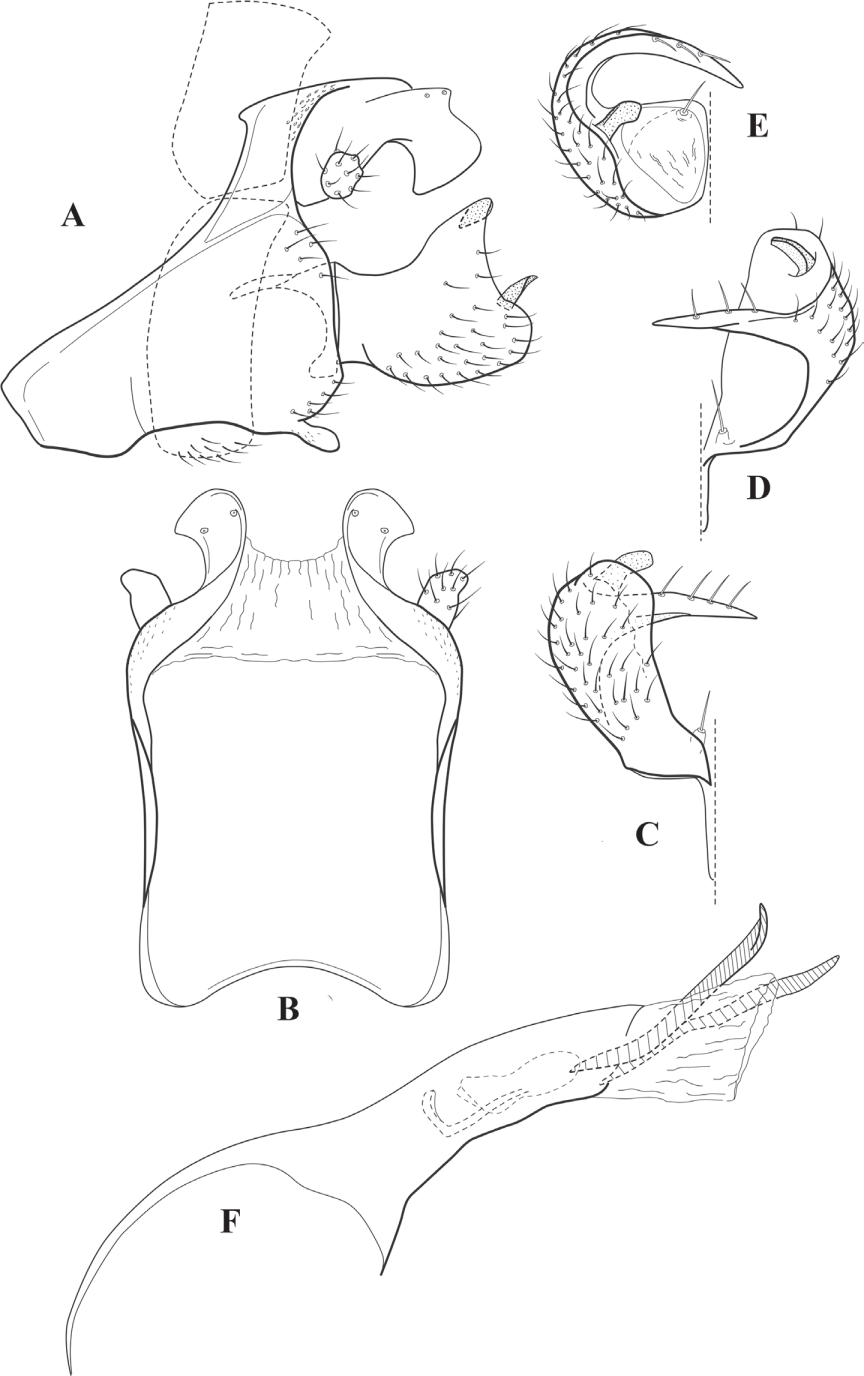
*Chimarraintexta* Mosely, ♂ genitalia **A** lateral **B** dorsal, segments IX and X **C** inferior appendage, ventral **D** inferior appendage, dorsal **E** inferior appendage, caudal **F** phallus, lateral.

###### Diagnosis.

*Chimarraintexta* is most readily diagnosed from other species of the subgroup by the shape and form of the lateral lobes of tergum X, which are short, weakly sclerotized, and have the out-turned lateral apices only weakly angulate, and also by the general form of the inferior appendages, which have the dorsal process strongly mesally curved, elongate, and acute apically, and also have a small tooth or cusp on the ventromesal surface, visible in lateral view.

###### Distribution.

Ghana, Guinea, Ivory Coast, Sierra-Leone.

##### ﻿ Chimarra minima

Taxon classificationAnimaliaTrichopteraPhilopotamidae

Ulmer, 1907

A4BC0ACA-7DC2-56D9-853C-5D7298BF5889

Fig. 30A–F

Chimarrha minima Ulmer, 1907: 43–44, fig. 64.
Chimarra minima Ulmer: Fischer 1961: 66; Gibon 2015: 335, 338, 348, fig. 3C–D (distribution, [table, map]: Togo, Ghana, Ivory Coast, Burkina Faso, Mali, Guniea, Cameroon).
Chimarra petri Gibbs, 1973: 369–371, figs 11–13, 21; Gibon 2015: 335 (as synonym of C.minima Ulmer).
Chimarra voltae Marlier, 1978: 288; Gibon 1985: 23 (as synonoym of C.petri Gibbs).

###### Material examined.

Ghana – **Brong Ahafo Reg.** ● 1♂1♀; Asubende, River Pru; 8°01'18"N, 1°01'58"W; 25 Nov. 1990; JS Amakye leg.; light trap; ZMBN. – **Northern Reg.** ● 7♂♂4♀♀; Bamboi, Black Volta; 8°08'50"N, 2°02'40"W; 25 Apr. 1991; JS Amakye leg.; light trap; ZMBN ● 1♀; same collection data as for preceding; UMSP ● 1♂; Sabari, Oti River; 9°17'41"N, 0°14'43"E; 10 Nov. 1993; T Andersen & J Kjærandsen leg.; light trap; ZMBN ● 1♂; same collection data as for preceding; UMSP.

**Figure 30.**
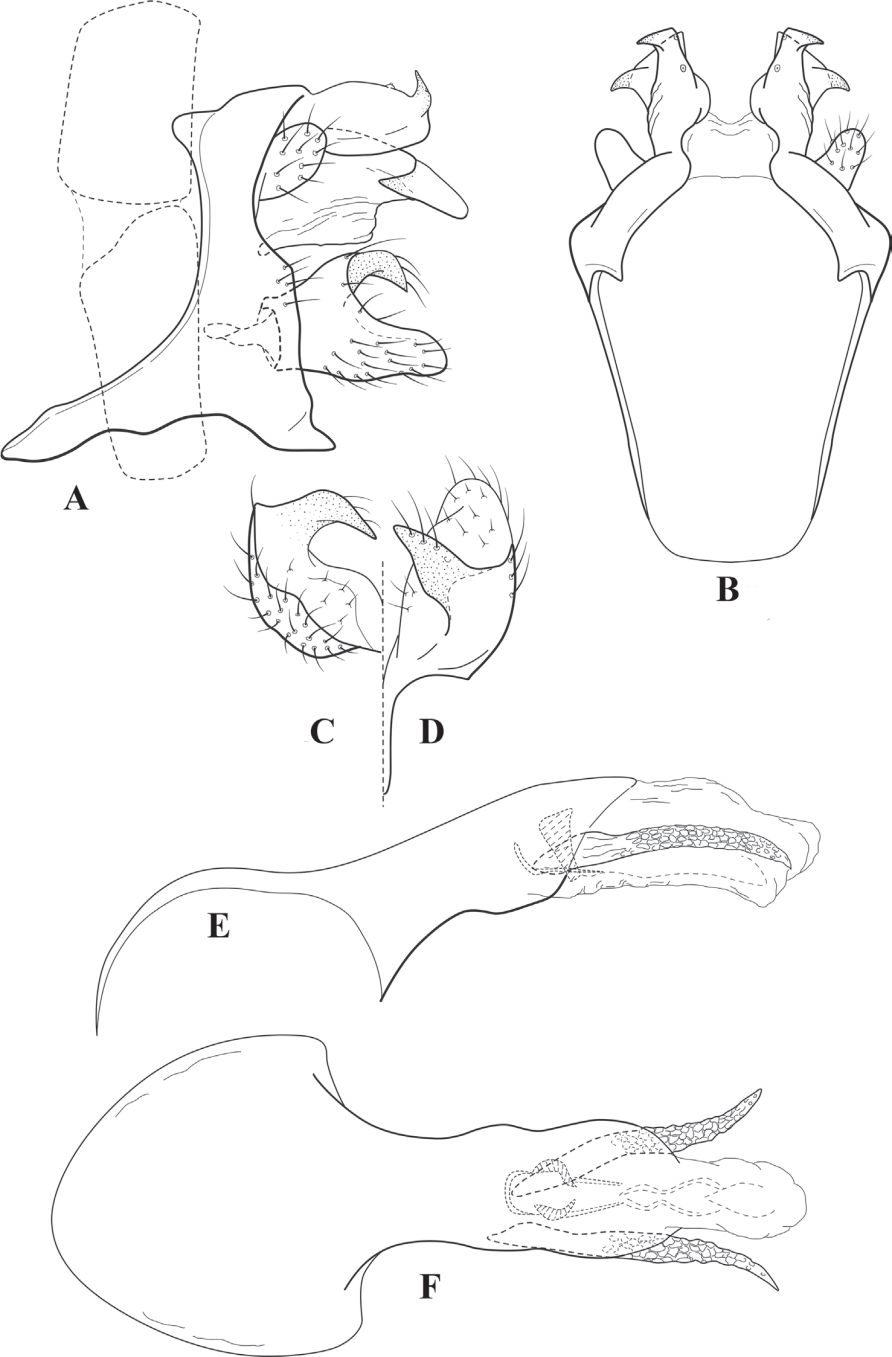
*Chimarraminima* Ulmer, ♂ genitalia **A** lateral **B** dorsal, segments IX and X **C** inferior appendage, caudal **D** inferior appendage, dorsal **E** phallus, lateral **F** phallus, dorsal.

###### Diagnosis.

*Chimarraminima* is a very distinctive species, easily diagnosed by the form of the lateral lobes of tergum X, each of which is divided into a dorsal and ventral lobe, each ending in an acute apical projection, that of the dorsal lobe directed upward and that of the lower lobe directed laterally. The form of the inferior appendage is also diagnostic in that the ventral part is relatively projecting and rounded apically, and the dorsal process is relatively basal, prominent, and posteromesally curved, thus forming a C-shaped dorsal projection in apposition to the ventral apex. The paired phallic spines are also unusual in having a distinctly reticulated structure.

###### Distribution.

Benin, Burkino Faso, Cameroon, Ghana, Guinea, Ivory Coast, Mali, Togo.

##### ﻿ Chimarra sassandrae

Taxon classificationAnimaliaTrichopteraPhilopotamidae

Gibon, 1982

BB830EA8-3E15-5F23-BAE6-E300A814AC2B

Fig. 31A–F

Chimarra sassandrae Gibon, 1982: 76, fig. 4, 10–11.
Chimarra sassandrae Gibon: Gibon 1985: 24 (distribution: Ivory Coast); Gibon 2015: 335, 338, 346, fig. 3E (distribution [table, map]: Ivory Coast, Guniea, Mali, Togo, Cameroon).

###### Material examined.

Ghana – **Brong Ahafo Reg.** ● 14♂♂25♀♀; Asubende, River Pru; 8°01'18"N, 1°01'58"W; 24–25 Feb. 1990; JS Amakye leg.; light trap; ZMBN ● 2♂♂; same collection data as for preceding except 18–19 Apr. 1991; Malaise trap; ZMBN. – **Northern Reg.** ● 12♂♂7♀♀; Bamboi, Black Volta; 8°08'50"N, 2°02'40"W; 25 Apr. 1991; T Andersen & JS Amakye leg.; light trap; ZMBN ● 9♂♂7♀♀; Sabari, Oti River; 9°17'41"N, 0°14'43"E; 27 Nov. 1990; JS Amakye leg.; light trap; ZMBN. – **Volta Reg.** ● 1♂; Hohoe, Matvin Hotel; 7°09'43"N, 0°28'31"E; 11 Nov. 1993; J Kjærandsen leg.; at light; ZMBN ● 1♂1♀; Kute, River Menu; 7°22'N, 0°36'E; 18 Nov. 1993; J Kjærandsen leg.; light trap; ZMBN ● 1♂; Wli, Agumatsa waterfall, station # 6; 7°07'29"N, 0°35'31"E; 20 Nov. 1993; J Kjærandsen leg.; light trap; UMSP ● 8♂♂7♀♀; same collection data as for preceding except station # 10; 19 Nov. 1993; ZMBN ● 8♂♂7♀♀; same collection data as for preceding except station # 12; 16 Nov. 1993; ZMBN ● 1♀; same collection data as for preceding; UMSP.

**Figure 31.**
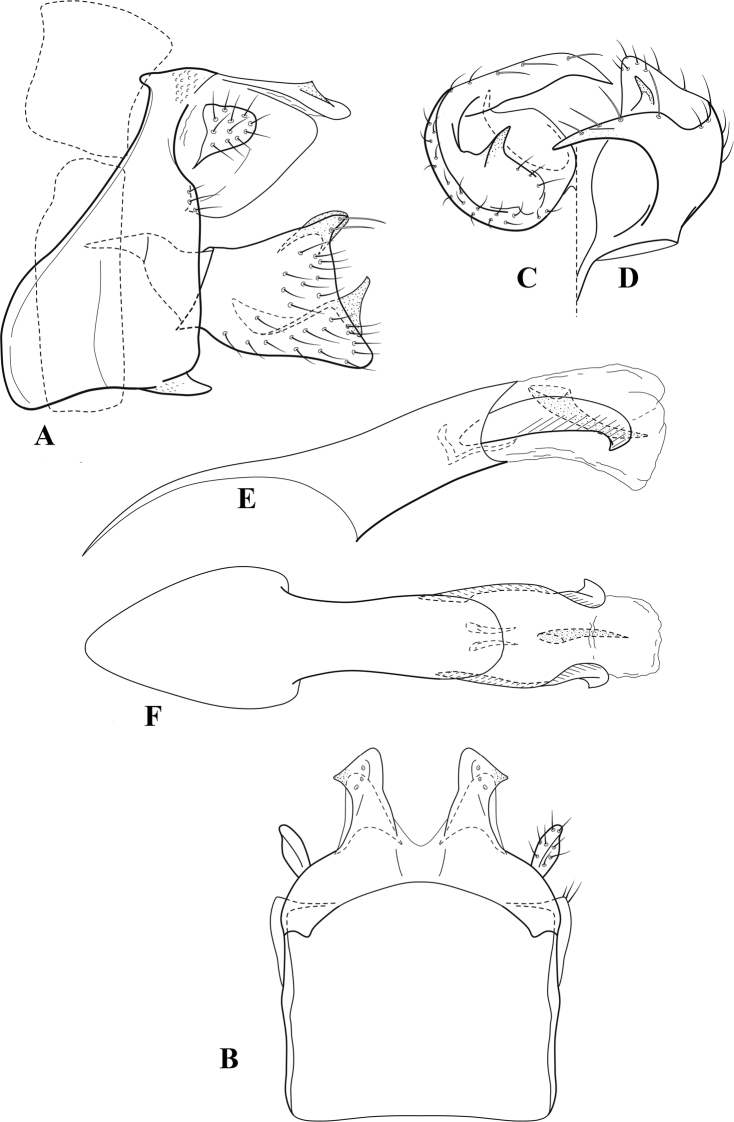
*Chimarrasassandrae* Gibon, ♂ genitalia **A** lateral **B** dorsal, segments IX and X **C** inferior appendage, caudal **D** inferior appendage, dorsal **E** phallus, lateral **F** phallus, dorsal.

###### Diagnosis.

*Chimarrasassandrae* has a general similarity to *C.intexta*, especially in the general attributes of the structure of tergum X and the inferior appendages. It can be diagnosed by details in both structures. The short, weakly sclerotized dorsal part of the lateral lobe of tergum X lies flatter and does not curve downward, and its apical projections are more acute. The inferior appendages have the dorsal projection even more strongly mesally curved and ventromesal tooth or cusp is more prominent and acute apically. This gives the inferior appendage, in lateral view, a distinctly more subquadrate appearance and this provides the most readily discernable diagnostic difference for the two species. Additionally, the anteroventral margin of segment IX is less produced than in *C.intexta* and the shape of the phallic spines is different.

###### Distribution.

Cameroon, Ghana, Guinea, Ivory Coast, Mali, Togo.

#### The *ruficeps* subgroup

**Included species.***Chimarrachechewa* Walhlberg, Espeland & Johanson, 2014; *C.circumverta* Wahlberg, Espeland & Johanson, 2014; *C.clara* Mosely, 1938; *C.cornuta* Jacquemart & Statzner, 1981 (homonym of *C.cornuta* Ross, 1959); *C.dulensis* sp. nov.; *C.fuscipes* Kimmins, 1958; *C.kibiensis* sp. nov.; *C.lwirona* Statzner, 1976; *C.minacis* sp. nov.; *C.ruficeps* Ulmer, 1914; *C.tangaensis* sp. nov.; and *C.uncata* Morse, 1974.

This subgroup is probably closely related to the *fallax* subgroup. Members of both subgroups have the ventral process of segment IX of males distinctly narrow and elongate (length at least 2× width at base, and usually much greater than this). The apex of this process in lateral view, in species of the *ruficeps* subgroup, is distinctly enlarged due to the presence of a cluster of small spines or thickened setae on its ventral margin. This compares to species in the *fallax* subgroup in which the apex, in lateral view, is either acute or without modified setation. At least for species in which the character is discussed, the color pattern in species of the *ruficeps* subgroup includes a yellowish head and thorax and contrastingly darker wings. Another distinguishing feature is in the structure of the lateral lobes of tergum X. In members of the *ruficeps* subgroup, the lateral lobes are incised apically into dorsal and ventral lobes; the ventral lobes may converge ventrally beneath the phallus, but are not fused basally, as the periphallic processes often are in members of the *fallax* subgroup. As compared to species in the *fallax* subgroup, the overall shape of segment IX is also different, usually with the anteroventral margin distinctly produced and invaginated or concave mesally, rather than only moderately produced and truncate or weakly invaginated mesally. Also, the inferior appendages do not emerge so far above the ventral process as they do in many members of the *fallax* subgroup. Finally, the ventral margin of segment VIII is not modified and projecting as it is in members of the *fallax* subgroup.

The differences characterizing the *fallax* and *ruficeps* subgroups may be difficult to assess from literature descriptions; it is possible that they are not completely consistent for all species of the subgroups. Assigning a species to one subgroup or the other is therefore sometimes problematic and, in some cases, may be equivocal. However, the overall subjective differences do seem to warrant the recognition of two subgroups.

*Chimarracornuta* Jacquemart & Statzner is a homonym of *C.cornuta* Ross from the New World and thus needs a new name. We prefer to defer this until the holotype is examined and a new illustration can be provided.

##### ﻿ Chimarra dulensis sp. nov.

Taxon classificationAnimaliaTrichopteraPhilopotamidae

BA742F46-ACC4-5E7A-80B2-C96A94925F2A

http://zoobank.org/EA7C917B-FEA2-41AC-A301-7AD5CF8E925C

Fig. 32A–D

###### Type material.

***Holotype*.** Tanzania – **Tanga Reg.** ● ♂ (in alcohol); West Usambara Mts, Dule; 4°51'S, 38°26'E; 26 Nov. 1990; T Andersen leg.; sweep net; UMSP 000550033.

###### Diagnosis.

*Chimarradulensis* most closely resembles *C.tangaensis* sp. nov., particularly in the overall shape of segment IX and shape of the inferior appendages, which are relatively short, with an acute dorsal apex. Both species also have an elongate, tube-shaped phallobase, with a projecting ventral apex and an endotheca with two elongate, symmetrical spines. The two species, however, are easily differentiated by the form of the lateral lobes of tergum X, which in *C.tangaensis* each have a dorsal spine-like projection, but in *C.dulensis* are simpler in form, elongate, with a more or less rounded, decurrent apex.

**Figure 32.**
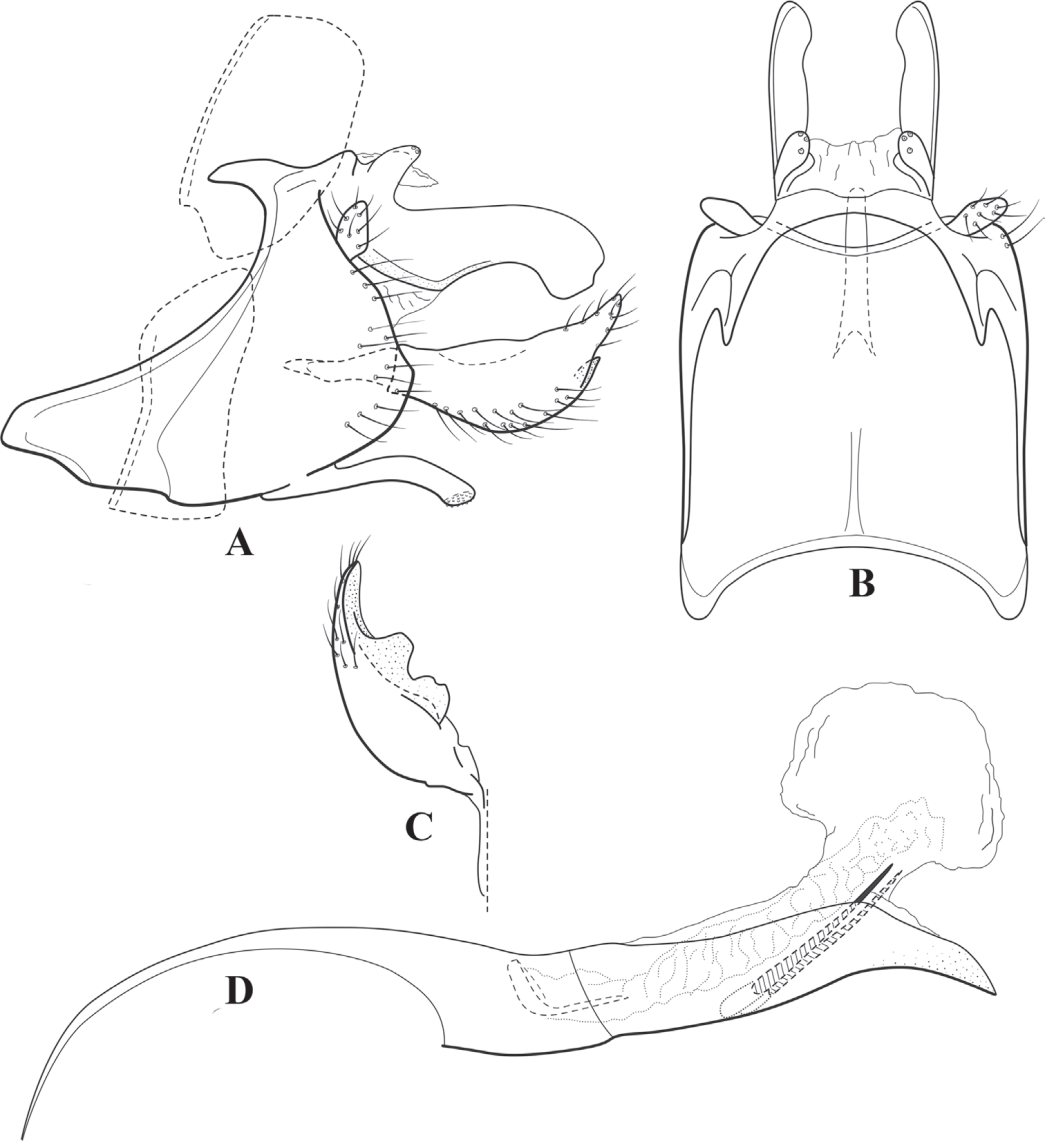
*Chimarradulensis* sp. nov., ♂ genitalia **A** lateral **B** dorsal, segments IX and X **C** inferior appendage, ventral **D** phallus, lateral.

###### Description.

***Adult.*** Overall color (in alcohol) medium brown. Head elongate (postocular parietal sclerite nearly equal to diameter of eye). Palps relatively elongate, maxillary palp with 1^st^ segment very short (slightly longer than wide), 2^nd^ segment short (~ 2× 1^st^), apex with small cluster of stiff setae, 3^rd^ segment elongate, almost 2× length of 2^nd^, 4^th^ segment short (subequal to 2^nd^), 5^th^ segment elongate (slightly longer than 3^rd^). Forewing length: male, 6.0 mm. Fore- and hind wing with forks I, II, III, and V present. Forewing with R_1_ slightly sinuous, stem of Rs with inflection at past midlength (with distinct node at inflection, almost appearing as crossvein), basal fork of discoidal cell somewhat enlarged, fork asymmetric, discoidal cell short, length ~ 1 1/2× width, forks I and II elongate, slightly subsessile, *r* crossvein diagonal, intersecting discoidal cell before fork I; *s*, *r-m*, and *m* crossveins co-linear, *s* pigmented (like wing), *r-m* and *m* crossveins hyaline, but distinct, 2A with crossvein (apparently forked apically to 1A and 3A). Hind wing with R_1_ fused to subcosta basally, both veins intersecting wing margin, fork I sessile, fork II subsessile. Forelegs with apical tibial spur distinct; male with foretarsi unmodified, claws small and symmetrical.

***Male genitalia.*** Segment VIII with sternum short, without ventromesal projection, tergum hardly longer. Segment IX, in lateral view, with anteroventral margin greatly, angularly, produced, anterodorsal margin with prominent apodeme, margin between strongly concave; dorsomesal margin of segment strongly concave, very short, but continuously sclerotized; segment, in lateral view, very short dorsally, posterior margin obliquely and almost linearly widened to inferior appendage, ventral margin rounded, ventral process emerging well below inferior appendages, very elongate, digitate, with apex rounded in lateral view, apex with short spines or setae; anteroventral margin of segment, in dorsal or ventral views, very strongly concave. Lateral lobes of tergum X relatively elongate, simple in structure, apices rounded and distinctly ventrally curved; each lobe with short rounded basodorsal projection with two small sensilla; mesal lobe of tergum X very short, membranous, hardly projecting beyond basal sensilla-bearing projections. Preanal appendages short and knob-like, weakly constricted basally. Inferior appendage relatively short, rounded basally, with only weak basal inflection, apex narrowed, subacute, strongly posteriorly projecting; in ventral view, with pair of sclerotized cusps on mesal margin. Phallic apparatus with phallobase elongate, tubular, with usual basodorsal expansion, apicoventral margin acute, distinctly projecting and somewhat downturned; endotheca membranous and apparently elongate, with two moderately elongate, slender, symmetrically positioned spines, membrane not noticeably textured; phallotremal sclerite complex composed of a moderate length rod-and-ring structure, without obvious apical sclerites.

###### Etymology.

*Chimarradulensis*, name used as an adjective, meaning “from Dule,” for the name of the town near which the type species was collected.

##### ﻿ Chimarra kibiensis sp. nov.

Taxon classificationAnimaliaTrichopteraPhilopotamidae

2994DCD6-9104-54AF-9994-62BC5F5B29AC

http://zoobank.org/F5993752-AC25-4017-AA14-7290CC5E8EF8

Fig. 33A–G

###### Type material.

***Holotype*.** Ghana – **Eastern Reg.** ● ♂ (in alcohol); Kibi, Subri stream; 6°10'N, 0°33'W; 5 Nov. 1993; J Kjærandsen leg.; light trap; UMSP 000550030.

###### Additional material.

Ghana – **Eastern Reg.** ● 1♀; Kibi, Subri stream; 6°10'N, 0°33'W; 5 Nov. 1993; J Kjærandsen leg.; light trap; UMSP.

###### Diagnosis.

*Chimarrakibiensis* is very similar to *C.minacis* sp. nov., as discussed in the diagnosis for that species, but the dorsal spine-like lobes of tergum X are shorter, and the ventral lobes of the tergum are longer, with the apices longer, more strongly ventrally curved, and also more sclerotized. Additionally, the inferior appendage in *C.kibiensis*, in lateral view, has an evident tooth on its posterior margin, whereas *C.minacis* has a pair of small mesal teeth or cusps; these are only readily evident in caudal view.

**Figure 33.**
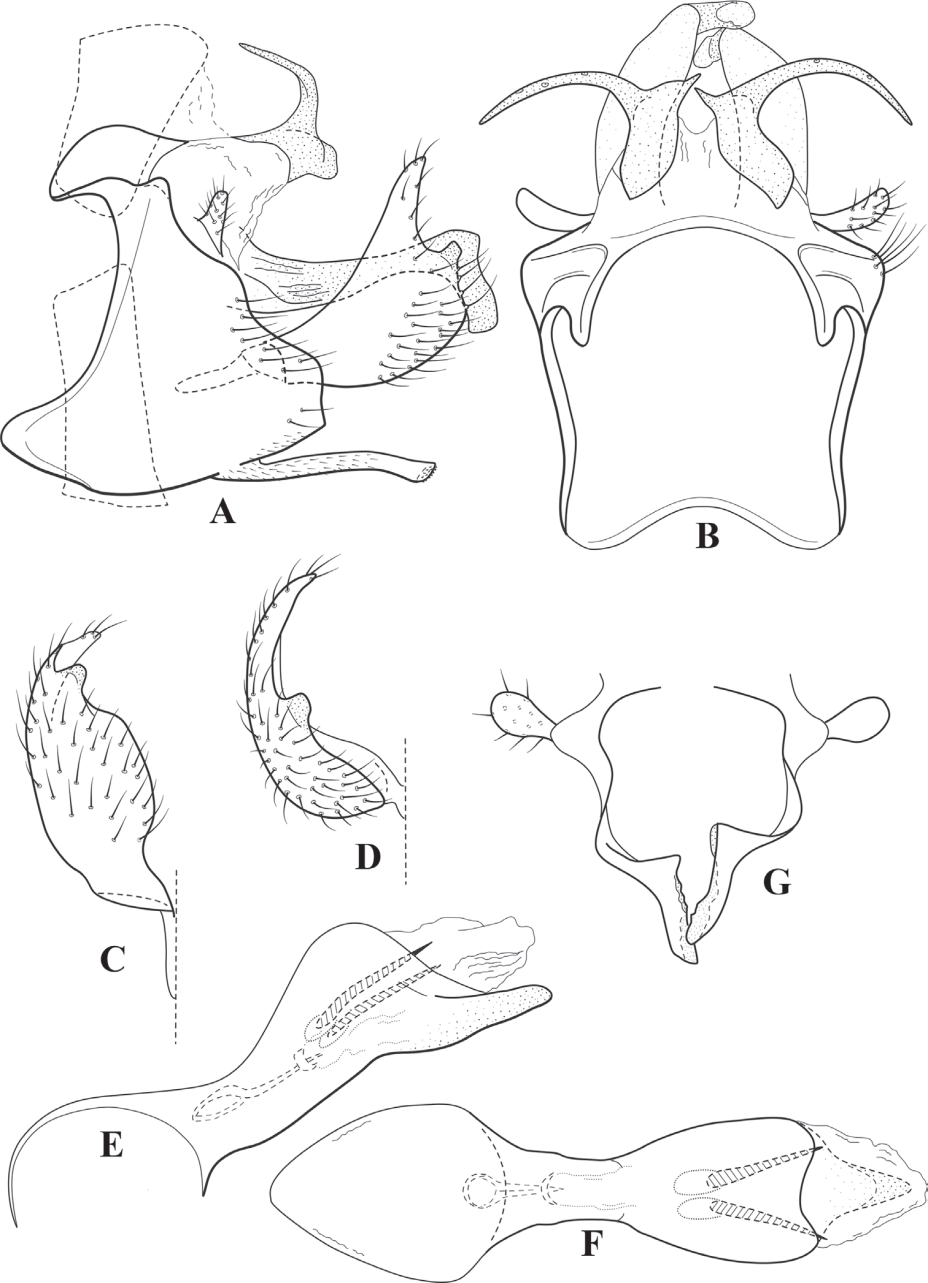
*Chimarrakibiensis* sp. nov., ♂ genitalia **A** lateral **B** dorsal, segments IX and X **C** inferior appendage, ventral **D** inferior appendage, caudal **E** phallus, lateral **F** phallus, dorsal **G** ventral lobes of tergum X, caudal.

###### Description.

***Adult.*** Overall color (in alcohol) nearly uniformly yellowish brown, spurs slightly darker. Head moderate in length (postocular parietal sclerite slightly > 1/2 diameter of eye). Palps moderately elongate; maxillary palp with 1^st^ segment very short (approximately as long as wide), 2^nd^ segment relatively short (< 3× 1^st^), apex with small cluster of stiff setae, 3^rd^ segment elongate, almost 2× length of 2^nd^, 4^th^ segment very short (shorter than 2^nd^), 5^th^ segment subequal to 3^rd^. Forewing length: male, 5.5 mm. Fore- and hind wing with forks I, II, III, and V present. Forewing with R_1_ sinuous, stem of Rs inflected at past midlength (with small node at inflection), basal fork of discoidal cell somewhat enlarged, fork slightly asymmetric, length of cell ~ 2× width, forks I and II subsessile, *r* crossvein diagonal, intersecting discoidal cell at approximately midlength, *s* and *r-m*, crossveins linear, *m* crossvein more proximal, *s* pigmented (like wing), *r-m* and *m* crossveins hyaline, 2A with crossvein (apparently forked apically to 1A and 3A). Hind wing with R_1_ narrowly parallel to subcosta, forks I and II subsessile. Forelegs with apical tibial spur distinct; male with foretarsi unmodified, claws small and symmetrical.

***Male genitalia.*** Segment VIII short, tergum not wider, sternum without ventromesal projection. Segment IX, in lateral view, with anteroventral margin distinctly produced, anterodorsal margin with broadly rounded apodeme, margin between strongly concave; dorsomesal margin of segment very short, but continuously sclerotized; posterior margin strongly produced in ventral half, strongly narrowed dorsally above inferior appendages, segment very short dorsally; ventral process emerging from ventral margin, very elongate, digitate, with apex rounded in lateral view, apex with short spines or setae; anteroventral margin of segment, in dorsal or ventral views, concave. Lateral lobes of tergum X each divided laterally into dorsal and ventral lobes, dorsal lobes strongly upturned and spine-like, recurved and very strongly sclerotized, especially compared to base, almost appearing as separate structures; ventral lobes very elongate and strongly sclerotized, with apices strongly, angularly downturned, apices of lobes rounded. Preanal appendages short and knob-like, constricted basally. Inferior appendage with pronounced basal inflection, dorsally with moderately elongate, tapering dorsal projection, apex subacute; posteromesal margin with prominent sclerotized cusp, readily visible in lateral view. Phallic apparatus with phallobase moderately elongate, with usual basodorsal expansion, apical half strongly flared and vase-like, apicoventrally with short rounded, sclerotized projection; endotheca with two moderately elongate, slender, symmetrically positioned spines, phallotremal sclerite complex composed of short rod and ring structure, with small apical sclerite.

###### Etymology.

*Chimarrakibiensis*, name used as an adjective (from Kibi), for the name of the town near where the type specimen was collected.

##### ﻿ Chimarra minacis sp. nov.

Taxon classificationAnimaliaTrichopteraPhilopotamidae

E014094C-761A-5D64-B248-73E1D78E9C28

http://zoobank.org/97E7A881-0991-47B9-840B-A7C27283BC77

Fig. 34A–E

###### Type material.

***Holotype*.** Ghana – **Volta Reg.** ● ♂ (in alcohol); Wli, Agumatsa waterfall, station # 3; 7°07'29"N, 0°35'31"E; 10 Mar. 1993; JS Amakye & J Kjærandsen leg.; light trap; UMSP 000550079. ***Paratypes*.** Ghana – **Volta Reg.** ● 1♂ (lacking abdomen); same data as for holotype except station # 2^B^; 5–8 Mar. 1993; Malaise trap; UMSP ● 3♂♂; same data as for holotype except 17 Nov. 1993; J Kjærandsen leg.; ZMBN ● 2♂♂; same data as for holotype except station # 6; 20 Nov. 1993; J Kjærandsen leg.; ZMBN.

**Figure 34.**
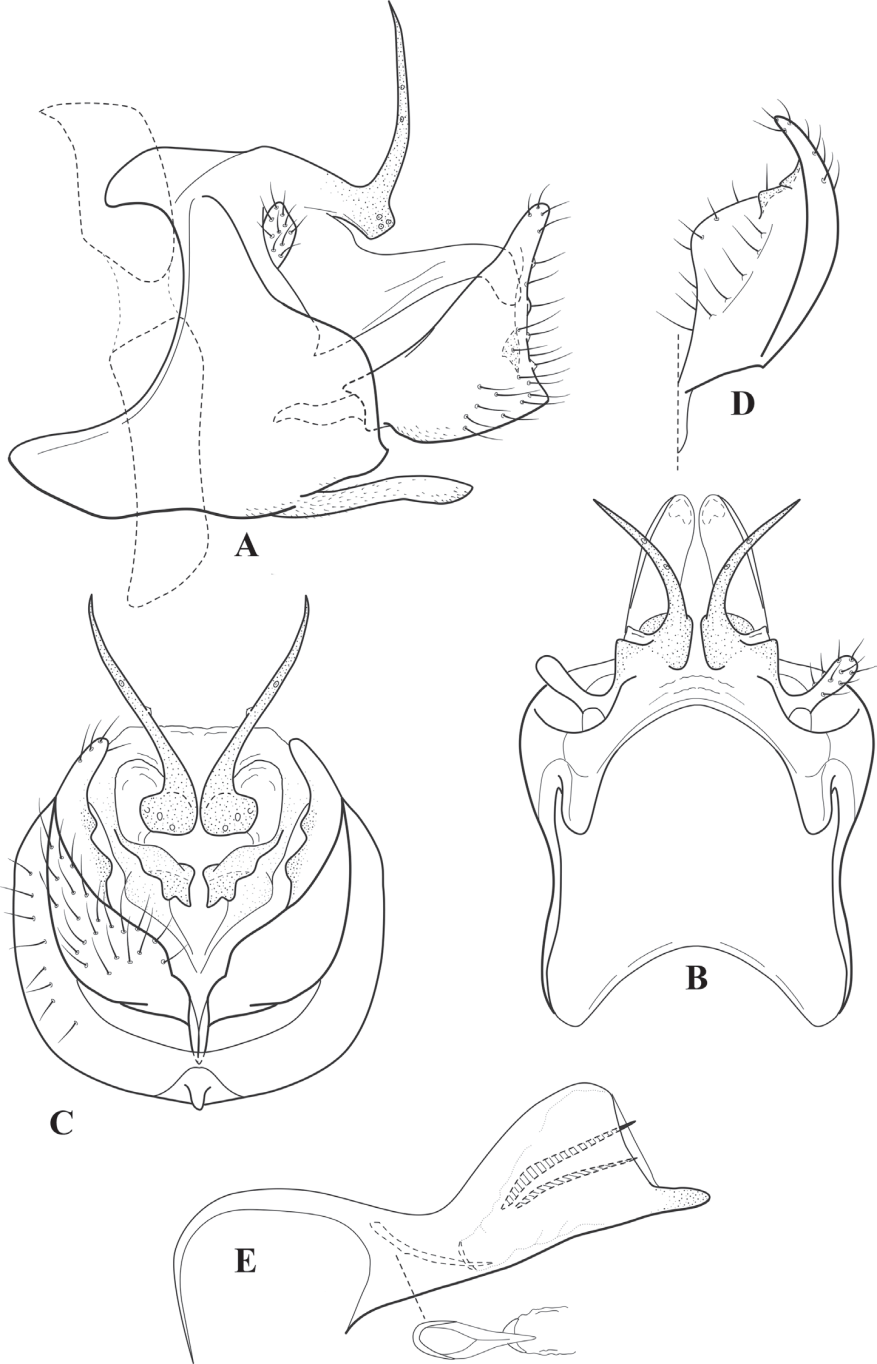
*Chimarraminacis* sp. nov., ♂ genitalia **A** lateral **B** dorsal, segments IX and X **C** caudal **D** inferior appendage, dorsal **E** phallus, lateral, with dorsal detail of phallotremal sclerite complex.

###### Diagnosis.

*Chimarraminacis* is very closely related to *C.kibiensis* sp. nov. The differences between the two are relatively minor, but distinctive. The decision to recognize them as different species is admittedly subjective. Although it is conceivable that they may eventually be shown to be forms of a single species, the use of names in the meantime is meant to draw attention to the distinctiveness of the forms. Both species are readily recognized by the strongly upturned dorsal spine-like lobes of tergum X, with the elongate and apically downturned ventral lobes of the same tergum. *Chimarracornuta* Jacquemart & Statzner also has spine-like lobes of tergum X, but in this species the lobes are not as upright, and the posterior margin of segment IX is not as produced in its ventral part as either of the two species discussed here. The primary difference of *C.minacis* from *C.kibiensis* is that the apices of the ventral lobes of tergum X are much more strongly developed and sclerotized in *C.kibiensis*, even resulting in some asymmetry of the lobes. Also, the sclerotized cusp or projection on the apical margin of the inferior appendage in *C.minacis* is not as strongly developed; notably it is not projecting or readily visible in lateral view; there is also a second small cusp found in *C.minacis*, not present in *C.kibiensis*.

###### Description.

***Adult.*** Overall color (in alcohol) nearly uniformly yellowish brown, spurs slightly darker. Head moderate in length (postocular parietal sclerite slightly > 1/2 diameter of eye). Palps moderately elongate; maxillary palp with 1^st^ segment very short (approximately as long as wide), 2^nd^ segment relatively short (< 3× 1^st^), apex with small cluster of stiff setae, 3^rd^ segment elongate, almost 2× length of 2^nd^, 4^th^ segment very short (shorter than 2^nd^), 5^th^ segment subequal to 3^rd^. Forewing length: male, 4.7–5.5 mm. Fore- and hind wing with forks I, II, III, and V present. Forewing with R_1_ sinuous, stem of Rs inflected at past midlength (with small node at inflection), basal fork of discoidal cell distinctly enlarged, fork slightly asymmetric, length of cell ~ 2× width, forks I and II subsessile, *r* crossvein diagonal, intersecting discoidal cell at approximately midlength, *s* and *r-m*, crossveins linear, *m* crossvein more proximal, *s* pigmented (like wing), *r-m* and *m* crossveins hyaline, 2A with crossvein (apparently forked apically to 1A and 3A). Hind wing with R_1_ narrowly parallel to subcosta, forks I and II subsessile. Forelegs with apical tibial spur distinct; male with foretarsi unmodified, claws small and symmetrical.

***Male genitalia.*** Segment VIII short, tergum not longer, sternum without ventromesal projection. Segment IX, in lateral view, with anteroventral margin greatly produced, anterodorsal margin with distinct and broadly rounded apodeme, margin between strongly concave; dorsomesal margin of segment very short, but continuously sclerotized; posterior margin strongly and truncately produced in ventral half, strongly narrowed dorsally above inferior appendages, segment very short dorsally; ventral process emerging from ventral margin, very elongate, digitate, with apex rounded in lateral view, apex with short spines or setae; anteroventral margin of segment, in dorsal or ventral views, very strongly concave. Lateral lobes of tergum X each divided laterally into dorsal and ventral lobes, dorsal lobes strongly upturned and spine-like, very strongly sclerotized, especially compared to base, almost appearing as separate structures; ventral lobes relatively elongate, with apices strongly, angularly downturned, apices of lobes rounded. Preanal appendages short and knob-like, constricted basally. Inferior appendage with pronounced basal inflection, dorsally with moderately elongate, tapering dorsal projection, apex subacute; posteromesal margin with a pair of small, sclerotized cusps, not or scarcely visible in lateral view. Phallic apparatus with phallobase moderately elongate, with usual basodorsal expansion, apical half strongly flared and vase-like, apicoventrally with short rounded, sclerotized projection; endotheca with two moderately elongate, slender, symmetrically positioned spines, membrane not noticeably textured, phallotremal sclerite complex composed of short rod and ring structure, with small apical sclerite.

###### Etymology.

*Chimarraminacis*, used as an adjective, from the Latin *minax*, meaning jutting out or threatening, for the upright spine-like processes on tergum X in this species.

##### ﻿ Chimarra tangaensis sp. nov.

Taxon classificationAnimaliaTrichopteraPhilopotamidae

1CDEC649-AAFB-5154-B8AB-817A90C66BCB

http://zoobank.org/C5D6476C-A2D5-4CA6-B8F4-D56548C5D090

Fig. 35A–E

###### Type material.

***Holotype*.** Tanzania – **Tanga Reg.** ● ♂ (in alcohol); West Usambara Mts., Mazumbai, Kaputu Stream; 4°48'S, 38°30'E; 4–13 Dec. 1990; T Andersen leg.; Malaise trap; UMSP 000550032. ***Paratypes*.** Tanzania – **Tanga Reg.** ● 5♂♂; same data as for holotype except 31 Oct. 1990–13 Jan. 1991; ZMBN.

###### Diagnosis.

*Chimarratangaensis* probably has its overall greatest similarity to *C.dulensis* sp. nov., particularly in the general shape of its inferior appendages, and in having an elongate, tubular phallobase with slender and symmetrically positioned phallic spines. It is easily distinguished by the more spine-like lateral lobes of tergum X. It is possible that the latter character reflects a closer relationship to the other new species of the *ruficeps* subgroup described here, which have the lobes even more dramatically developed into spine-like processes.

**Figure 35.**
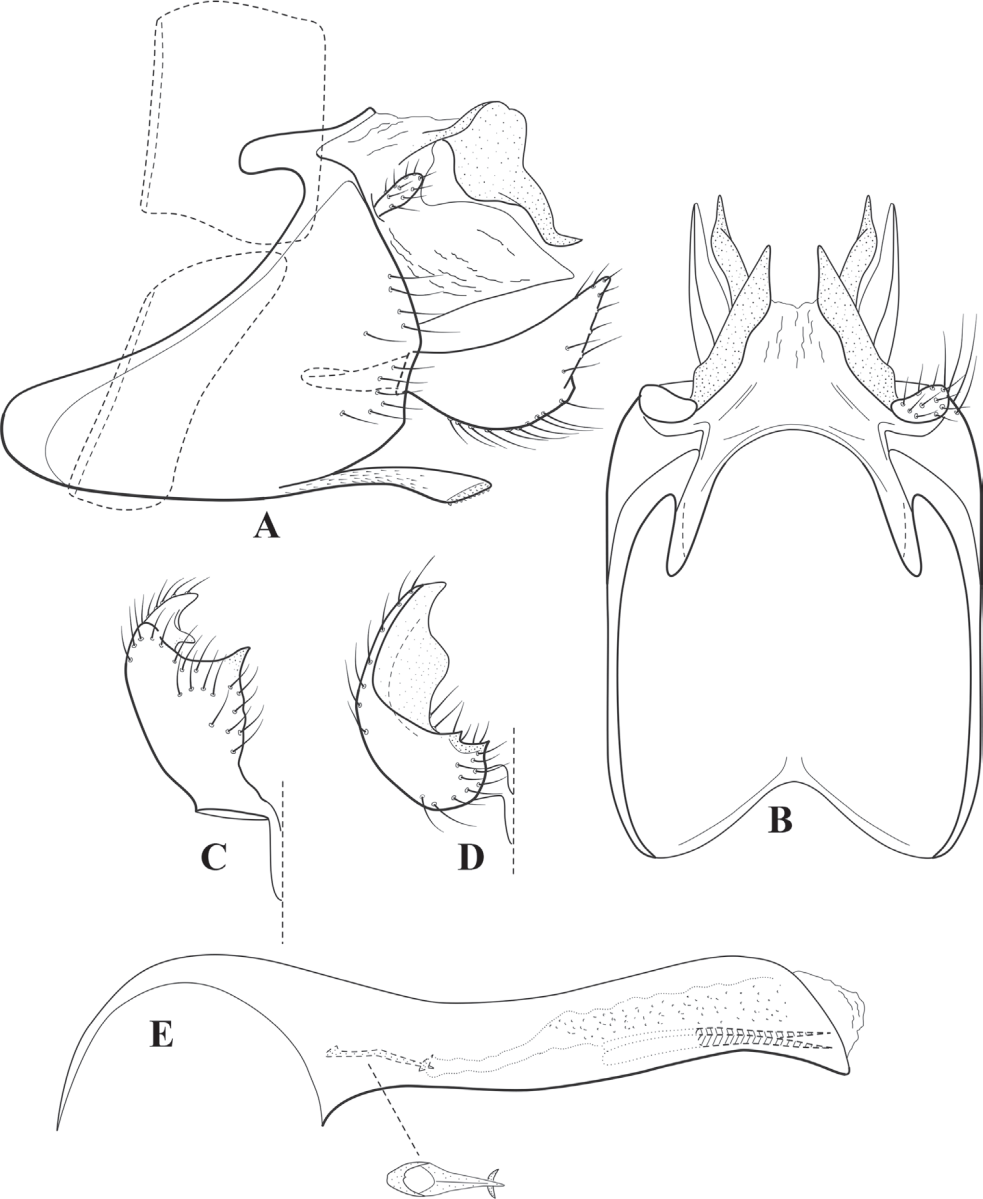
*Chimarratangaensis* sp. nov., ♂ genitalia **A** lateral **B** dorsal, segments IX and X **C** inferior appendage, ventral **D** inferior appendage, caudal **E** phallus, lateral, with dorsal detail of phallotremal sclerite complex.

###### Description.

***Adult.*** Overall color (in alcohol) medium brown. Head relatively elongate (length of postocular parietal sclerite nearly equal to diameter of eye). Palps moderately elongate, maxillary palp with 1^st^ segment very short (slightly longer than wide), 2^nd^ segment short (~ 2× 1^st^), apex with small cluster of stiff setae, 3^rd^ segment elongate, almost 2× length of 2^nd^, 4^th^ segment short (subequal to 2^nd^), 5^th^ segment elongate (slightly longer than 3^rd^). Forewing length: male, 7.5–8.5 mm. Fore- and hind wing with forks I, II, III, and V present. Forewing with R_1_ slightly sinuous, stem of Rs weakly inflected at past midlength (with indistinct node at inflection, almost appearing as crossvein), basal fork of discoidal cell somewhat enlarged, fork almost symmetric, discoidal cell elongate, length ~ 2 1/2× width, forks I and II slightly subsessile, *r* crossvein diagonal, intersecting discoidal cell at past midlength, before fork I, *s* and *r-m*, crossveins linear, *m* crossvein more proximal, ~ 1/2 way between basal fork of M and *r-m* crossvein, *s* pigmented (like wing), *r-m* and *m* crossveins hyaline, 2A with crossvein (apparently forked apically to 1A and 3A). Hind wing with R_1_ fused to subcosta basally, both veins intersecting wing margin, forks I and II slightly subsessile. Forelegs with apical tibial spur distinct; male with foretarsi unmodified, claws small and symmetrical.

***Male genitalia.*** Segment VIII with sternum short, without ventromesal projection, tergum slightly longer. Segment IX, in lateral view, with anteroventral margin greatly, subangularly, produced, anterodorsal margin with prominent apodeme, margin between strongly concave; dorsomesal margin of segment strongly concave, very short, but continuously sclerotized; segment, in lateral view, very short dorsally, posterior margin obliquely and almost linearly widened to inferior appendage, ventral margin rounded, ventral process emerging somewhat below inferior appendages, very elongate, digitate, with apex rounded in lateral view, apex with short spines or setae; anteroventral margin of segment, in dorsal or ventral views, very strongly concave. Lateral lobes of tergum X moderately elongate, divided into dorsal and ventral lobes, ventral lobes (possibly sclerotized extensions of periphallic membrane) relatively lightly sclerotized, wide basally, subacute apically, dorsal lobes more distinctly sclerotized, with rounded and projecting basodorsal process and posteriorly projecting apical process, narrowing to a somewhat spine-like apex, sensilla not apparent; mesal lobe of tergum X short, membranous, only extending to basodorsal processes of dorsal lobes of lateral lobes. Preanal appendages short and knob-like, weakly constricted basally. Inferior appendage relatively short, rounded basally, with moderate basal inflection, apex narrowed, acute, moderately posteriorly projecting; in caudal view, with rounded projection from dorsomesal margin. Phallic apparatus with phallobase elongate, tubular, with usual basodorsal expansion, apicoventral margin slightly projecting and somewhat downturned; endotheca membranous and apparently elongate, with two moderately elongate, slender, symmetrically positioned spines, phallotremal sclerite complex composed of moderately elongate rod and ring structure, with small apical sclerites.

###### Etymology.

*Chimarratangaensis*, used as an adjective, meaning “from Tanga,” for the region in Tanzania in which this species was collected.

#### Species not assigned to subgroup

##### ﻿ Chimarra multisensillata sp. nov.

Taxon classificationAnimaliaTrichopteraPhilopotamidae

2B0A19E1-5825-5BF3-94AF-62BF4DDC1E19

http://zoobank.org/5C9B0780-5053-4DAC-8E63-07D32C0F84BC

Fig. 36A–F

###### Type material.

***Holotype*.** Tanzania – **Tanga Reg.** ● ♂ (in alcohol); East Usambara Mts, Fanusi; 28 Feb. 1959; 1.000 ft; MT Gillies leg.; INHSTrichoptera 50336.

###### Diagnosis.

*Chimarramultisensillata* is a distinctive species, unlike any other described species, with several unusual characteristics. Its most diagnostic feature is the shape of the inferior appendage, which has a narrow, sclerotized, digitate projection on the dorsal margin at just past midlength, that is oriented more or less parallel to the appendage itself. Also distinctive is the overall form of the lateral lobes of tergum X, which are very elongate and simple in structure, with ~ 10 unraised sensilla scattered laterally along its length. Another unusual characteristic, for a species in the *marginata* Group, is the relatively elongate, tubular phallobase with two elongate, sclerotized, symmetrically arranged spines, possibly modified elements of the phallotremal sclerite complex. Finally, the shape of segment IX is also unusual in having is anterior margin uniformly concave and its ventral margin strongly produced, with lateral margins that are somewhat convergent, as viewed dorsally or ventrally.

**Figure 36.**
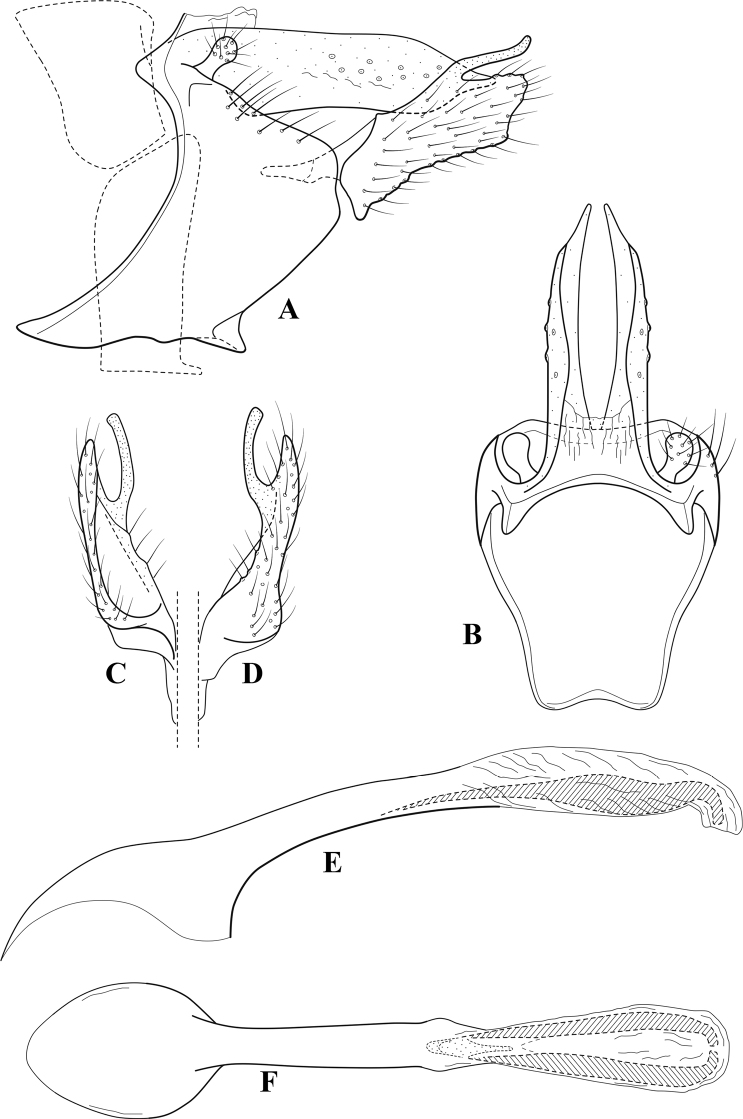
*Chimarramultisensillata* sp. nov., ♂ genitalia **A** lateral **B** dorsal, segments IX and X **C** inferior appendage, ventral **D** inferior appendage, dorsal **E** phallus, lateral **F** phallus, dorsal.

Because of having multiple sensilla on tergum X, rather than just two, as is typical of species in the *marginata* Group, one might question its placement in the group. Its venational characters, however, are typical of species in the *marginata* Group. Nevertheless, it probably represents a relatively basal species in the lineage.

###### Description.

***Adult.*** Overall color (in alcohol) yellowish brown. Head relatively short (postocular parietal sclerite < 1/2 diameter of eye). Palps moderately elongate, maxillary palp with 1^st^ segment short (length subequal to width), 2^nd^ segment short (~ 2× 1^st^), apex with small cluster of stiff setae, 3^rd^ elongate (> 2× as long as 2^nd^), 4^th^ segment short (subequal to 2^nd^), 5^th^ segment very elongate (longer than 3^rd^). Forewing length: male, 5.0 mm. Fore- and hind wings with forks I, II, III, and V present. Forewing with R_1_ distinctly sinuous, stem of Rs inflected at just past midlength, with small node at inflection, not extending into cell below, basal fork of discoidal cell enlarged, fork asymmetric, discoidal cell with length ~ 2× width, fork I sessile, fork II stalked, *r* crossvein diagonal, intersecting discoidal cell at past midlength, *r-m* crossvein continuous with *s*, *m* crossvein proximal to *s* and *r-m* crossveins, approximately midway between basal fork of M and *r-m* crossvein, *s* pigmented (like wing), *r-m* and *m* crossveins hyaline, very faint, 2A with crossvein (apparently forked apically to 1A and 3A). Hind wing with R_1_ fused to subcosta basally, both veins intersecting margin of wing, fork I sessile, fork II stalked. Foreleg with apical tibial spur distinct; male with foretarsi unmodified, segments very narrow, claws small and symmetrical.

***Male genitalia.*** Segment VIII with sternum relatively short, with short ventromesal projection, tergum approximately same length. Segment IX, in lateral view, relatively elongate, with anteroventral margin greatly produced, anterodorsal margin with small rounded apodeme, margin between strongly concave; dorsomesal margin of segment strongly concave, very short, but continuously sclerotized; segment, in lateral view, short dorsally, posterior margin greatly, convexly produced between preanal appendages and small, subtriangular ventral process, inferior appendage mounted at midheight; anteroventral margin of segment, in dorsal or ventral views, with lateral margins converging, mesal margin with weak concave emargination. Lateral lobes of tergum X elongate, parallel sided, tapering apically, simple in structure, lateral margin with ~ 10 unraised sensilla scattered along its length; mesal lobe of tergum X very short, membranous, much shorter than lateral lobes. Preanal appendages short and knob-like, distinctly constricted basally. Inferior appendage moderately elongate, projecting, without significant basal inflection, dorsal and ventral margins subparallel, with narrow, digitate, posteriorly projecting process at approximately midlength from dorsal margin, length of inferior appendage slightly > 2× its width, apex narrowing, rounded. Phallic apparatus with phallobase very elongate and narrowly tubular, with pair of very elongate, narrow sclerites, wider at midlength (possibly modified lateral sclerites of phallotremal sclerite complex), positioned apical to a more or less typical and moderately elongate rod and ring structure of phallotremal sclerite complex.

###### Etymology.

*Chimarramultisensillata*, name used as an adjective, for the relatively numerous sensilla on the lateral lobes of tergum X, a very unusual characteristic for a species in the *marginata* Group.

#### The *georgensis* Group

This group was designated by Blahnik et al. (2012), when describing a new species from Vietnam. The group is otherwise only definitively known from Africa. *Chimarrageorgensis*Barnard (1934), besides being the first described species in the group, is also the designated type species for the genus *Chimarrhafra*Lestage (1936), a genus synonynized with *Chimarra* by Ross (1956). Thus, *C.georgensis* has formal name priority over other species of the group, unless the genus *Vigarrha*, a monotypic genus from the Philippines, also synonymized under *Chimarra* by Ross (1956), can be demonstrated to belong to the group. Ross believed the two genera to be related. The issue is somewhat complicated and further discussed below in the characterization of the *georgensis* subgroup. African species of the group were listed by Gibon (2018) when describing a new species of the group from Madagascar. As recognized here, these species all belong to the *georgensis* subgroup. Another subgroup, the *evoluta* subgroup, is proposed in this paper, containing three described species and also additional species described herein.

The two subgroups of the *georgensis* Group are quite distinctive. The characters uniting them are a bit more difficult to characterize. Venational characters used to characterize the group as a whole, including the absence of a crossvein in the anal veins of the fore wing (both 2A and 3A looped to the 1A), a linear arrangement of the *s*, *r-m*, and *m* crossveins of the forewing, and a straight (or nearly straight) Rs vein in the forewing, are probably all plesiomorphic characters, at least with respect to other subgenera within *Chimarra*. Both subgroups are also characterized by a loss of the R_1_ vein of the hind wing (or its fusion to the subcosta). Thus, only a single vein of the two reaches the margin of the wing. However, this is not a unique character within *Chimarra*. It also characterizes most species of the subgenus Otarrha in the New World and seems to be a consistent character of the *minuta* Group of the Chimarra in Asia (whose relationship to the subgenus *georgensis* Group remains to be assessed). Since the subcosta and R_1_ veins of the hind wing run narrowly parallel in many species of *Chimarra*, its loss is to be expected, especially in smaller species. It also makes determination of the character state difficult. The recent addition of photographs of wings in some publications of new species of *Chimarra*, as for instance, Johanson and Espeland (2010), Johanson et al. (2011), Johanson and Oláh (2012), and Gibon (2015), reveals that the loss or fusion of the R_1_ vein in the hind wing characterizes many taxa in the *marginata* Group of the subgenus Chimarra, in addition to species of the *georgensis* Group, and also that the loss of fork I and fork III in the hind wing, either independently or together, is not uncommon. These are the most usual venational modifications occurring in the genus and seem to have occurred independently in various lineages or species. Nevertheless, loss of the R_1_ vein of the forewing may be a synapomorphy for the taxa placed in the *georgensis* Group. In addition to this admittedly homoplasious character, most species of both the *georgensis* and *evoluta* subgroups are characterized by a rather short phallobase that is anchored in place by a sclerotization of the periphallic membrane, forming a phallocrypt that holds the short phallobase in place (so that it is not easily removed). This is usually more evident in the *georgensis* subgroup. Members of both subgroups also have maxillary palps in which the terminal segment is unusually elongate. These characters, in combination, are suggestive enough to place both subgroups into a common taxon. We recognize that this placement deserves further evaluation.

#### The *georgensis* subgroup

**Included species.***Chimarraankylis* sp. nov.; *C.aurita* sp. nov.; *C.corneola* Blahnik, Arefina-Armitage & Armitage, 2012; *C.crescentis* sp. nov.; *C.furcata* Jacquemart, 1961; *C.georgensis* Barnard, 1934; *C.hoogstraali* Ross, 1956; *C.indicis* sp. nov.; *C.kabashana* (Marlier, 1943); *C.latidentis* sp. nov.; *C.leptodactylus* sp. nov.; *C.obuncata* sp. nov.; *C.polycentropoides* sp. nov.; *C.ralphi* sp. nov.; *C.serrella* sp. nov.; *C.triramosa* sp. nov.; *C.uncinata* sp. nov.; *C.vermitergata* sp. nov.; and *C.zombitsei* Gibon, 2018.

This subgroup has the R_1_ vein of the hind wing either completely obsolete, or present basally, but obsolete or fused to the subcosta apically. Notably, only one vein intersects the wing margin. The venation of the hind wing is otherwise complete for *Chimarra*, with forks I, II, III, and V present. However, usually fork III is relatively narrowly forked. All of the species seem to have a relatively short phallobase, with the ventral apex strongly projecting and downturned, and often modified. The most usual modification is for the apex to be bifid, or that is with a mesal invagination and lateral projections on either side. In the species from Tanzania, this ventral projection is apparently fused to the sclerotized periphallic membrane, and thus it is difficult to say whether this is actually a phallic structure or independently derived and fused periphallic processes. The former seems more likely. However, the situation is further complicated in that there is a sclerotized extension of the phallobase beyond this ventral projection, which may be due to a secondary sclerotization of the base of the endotheca in these species. All of the species in the *georgensis* subgroup from Ghana have males with enlarged and modified claws on the forelegs, whereas the four species from Tanzania placed in this subgroup have the claws small and unmodified. Since modified claws on the forelegs is a common feature throughout the genus *Chimarra*, including the *evoluta* subgroup, their absence in the species from Tanzania probably represents a loss. This character, in combination with the modified apex of the phallobase discussed above, can be taken as evidence for the monophyly of the Tanzanian species placed in this subgroup.

To date, only a single species of this subgroup has been reported from Asia (*Chimarracarneola* Blahnik, Arefina-Armitage & Armitage from Vietnam). However, *C.giacomazzoi*Malicky (2015a), described from Guangxi, China is very similar in morphology and probably also belongs here. Malicky stated, with the description of the species, that other species with a similar morphology exist, without stating their geographic location or whether they were currently described. *Chimarrapotamophila*Mey (1995), described from Mindoro in the Philippines, also shares some of the features of this group and at least a superficial similarity. Unfortunately, venational characters of the species were not discussed in its initial description. Ross (1956) stated that *Vigarrhatibialis*Navás (1922), described from Luron (Luzon?) in the Philippines was related to the *Chimarrhafra* group from Africa. However, he placed both in the genus *Chimarra* and never discussed the morphological characters that he used to make his assessment. If related, the name *Vigarrha* (and its type species) would have name precedence in referring to this group. *Vigarrhatibialis* was based on a female specimen and only a hind wing was illustrated (Navás 1922: fig. 1). A male of the species was later illustrated by Ulmer (1930: fig. 7), based on specimens from Leyte and Mindanao, rather than Luzon. Thus, it is difficult to know whether the male illustrated actually represents the same species described by Navás. As illustrated by Navás (1922: fig. 1) *Vigarrhatibialis* possesses the R_1_ vein in the hind wing but lacks fork III. Although the second character occurs in some species of the *evoluta* subgroup of Africa (with the additional loss of fork I), none of the known species of the *georgensis* Group have the R_1_ vein of the hind wing present. Moreover, loss of this vein was a primary character used by Lestage (1936) to define the genus *Chimarrhafra*. Based on this conflicting evidence, both the existence of the *georgensis* Group or subgroup in the Philippines, and its possible relationship to the genus *Vigarrha*, is inconclusive and needs further investigation.

##### ﻿ Chimarra ankylis sp. nov.

Taxon classificationAnimaliaTrichopteraPhilopotamidae

332C81DD-683C-5569-9095-599420F7E1DD

http://zoobank.org/EBBF7A2F-B3EE-4CF2-A487-805445ACCC82

Fig. 37A–F

###### Type material.

***Holotype*.** Tanzania –**Tanga Reg.** ● ♂ (in alcohol); West Usambara Mts, Mazumbai, Kaputu Stream; 4°48'S, 38°30'E; 26–29 Nov. 1990; T Andersen leg.; Malaise trap; UMSP 000550076. ***Paratypes*.** Tanzania –**Tanga Reg.** ● 10♂♂; same data as for holotype; UMSP ● 35♂♂; same data as for holotype except 10 Nov. – 3 Dec. 1990; ZMBN.

**Figure 37.**
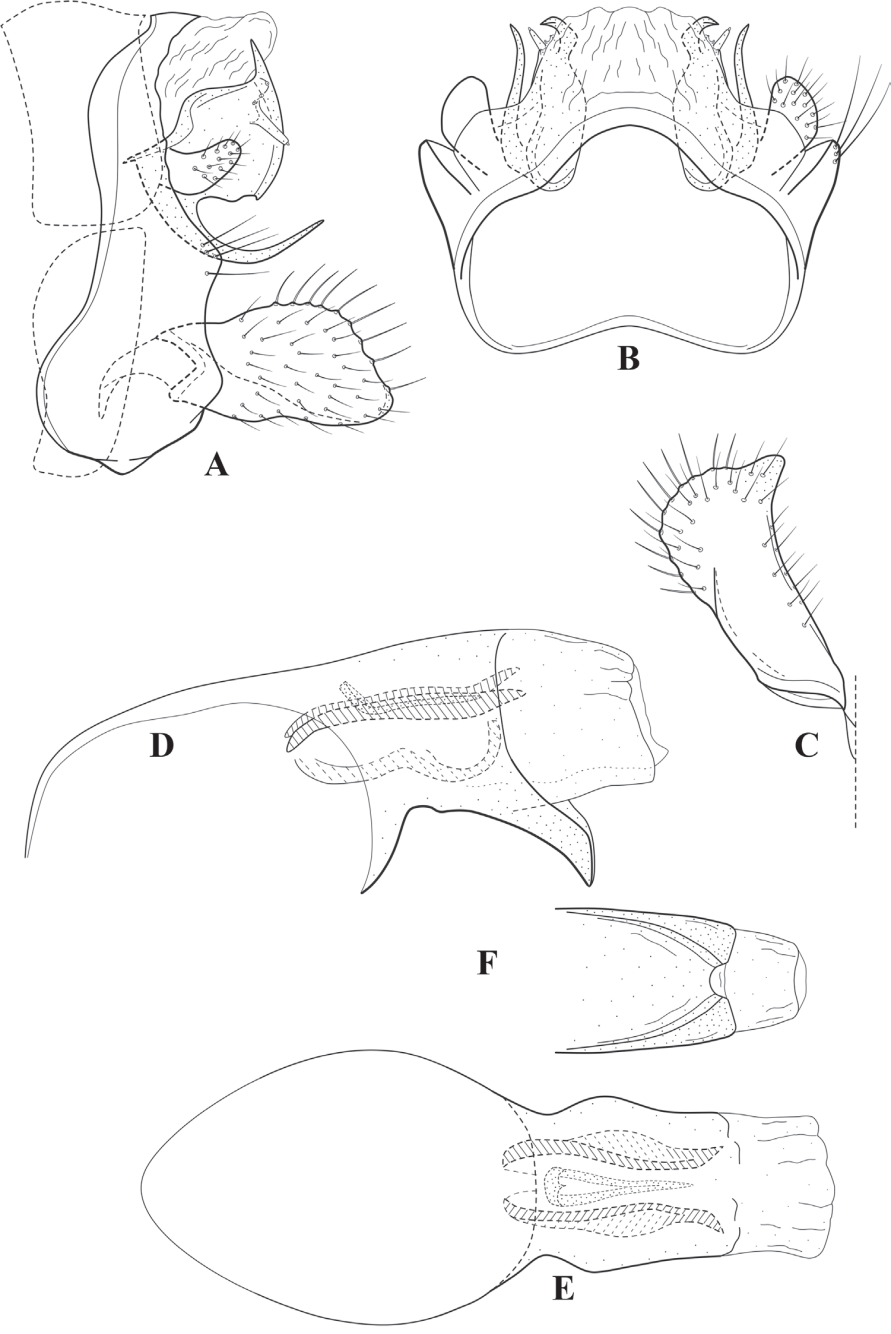
*Chimarraankylis* sp. nov., ♂ genitalia **A** lateral **B** dorsal, segments IX and X **C** inferior appendage, venral **D** phallus, lateral **E** phallus, dorsal **F** phallus apex, ventral.

###### Diagnosis.

*Chimarraankylis* is readily diagnosed by the distinctive characteristics of the lateral lobes of tergum X, which are very short and have both a short, acute, dorsally directed apical projection and a more prominent, acute, curved, basoventral projection. Also distinctive is a relatively elongate and very narrow lateral projection on each of the lateral lobes of tergum X, with an apical sensillum, in addition to several small sensilla basal to this structure. The inferior appendage is subovate, as viewed laterally, but has a short, subacute apex on its mesal surface as viewed ventrally. The phallobase, is both large and short, as is characteristic of the group, with its apicoventral projection relatively wide, sclerotized laterally, and with a small desclerotized notch mesally. The phallic spines, possibly actually components of a phallotremal sclerite complex, are relatively elongate and prominent.

###### Description.

***Adult.*** Overall color (in alcohol) dark brown. Head short (postocular parietal sclerite < 1/2 diameter of eye). Palps elongate; maxillary palp with 1^st^ segment short (slightly longer than wide), 2^nd^ segment moderately elongate (> 3× 1^st^), apex with cluster of ~ 8–10 stiff setae, 3^rd^ segment elongate (distinctly longer than 2^nd^), 4^th^ segment short (1/2 length of 2^nd^), 5^th^ segment very elongate and narrow (subequal to 3^rd^ and 4^th^ combined). Forewing length: male, 5.0–6.0 mm. Fore- and hind wings with forks I, II, III, and V present. Forewing with R_1_ straight, stem of Rs straight, basal fork of discoidal cell not enlarged, evenly forked, length of cell ~ 2× width, forks I and II sessile, *r* crossvein diagonal, intersecting discoidal cell before apical fork, *s*, *r-m*, and *m* crossveins linear and hyaline, both 2A and 3A looped to 1A (2A without apical fork). Hind wing with R_1_ obsolete or fused to subcosta, forks I, and II distinctly subsessile, anal loop small. Forelegs with apical tibial spur short; male with foretarsi modified, tarsal claws enlarged and asymmetrically developed.

***Male genitalia.*** Segment VIII very short, tergum slightly longer than sternum, sternum without posteroventral projection. Segment IX, in lateral view, very short, anteroventral margin only slightly expanded in ventral 1/3, dorsal margin without apodemes, sternum with very short, rounded ventral process from ventral margin, inferior appendages inserted near ventral margin; as viewed dorsally, with tergum very narrow, but continuous, sternum short, subtruncate, very shallowly emarginate mesally. Tergum X with mesal lobe short and membranous, lateral lobes very short and strongly sclerotized, each with short, acute apicodorsal projection and much larger, curved, basoventral, spine-like projection; preapical lateral margin with two or three small sensilla, one on a narrow digitate projection. Preanal appendages short and rounded, relatively prominent, constricted basally. Inferior appendage not or only weakly inflected basally, appendage relatively simple in structure, longer than wide, apicoventral margin somewhat projecting, forming subacute apicomesal projection, as viewed ventrally. Phallic apparatus with phallobase very short and wide, strongly sclerotized, with usual basodorsal expansion, securely anchored within segment by sclerotized periphallic membrane; apicoventral margin of phallobase very distinctly sclerotized and produced, downturned; apex, as viewed ventrally, relatively wide, with sclerotized lateral margins, apex subtruncate, only very shallowly emarginate mesally; endotheca simple and membranous. Armature of phallus relatively prominent, extending nearly length of phallobase, possibly entirely part of a modified phallotremal sclerite complex, composed of a moderate length rod-and-ring structure and enlarged pair of dorsolateral sclerites, appearing as pair of symmetrical spines.

###### Etymology.

*Chimarraankylis*, used as a noun in apposition, from the Greek word *ankylis*, a hook or barb, for the hooked ventral projection from the lateral lobes of tergum X in this species.

##### ﻿ Chimarra aurita sp. nov.

Taxon classificationAnimaliaTrichopteraPhilopotamidae

90896B79-D136-5424-BF14-D58722012E5E

http://zoobank.org/99E6C630-4634-4DF8-AC12-BA3543343DC1

Fig. 38A–F

###### Type material.

***Holotype*.** Ghana – **Western Reg.** ● ♂ (in alcohol); Ankasa Game Production Reserve; 5°15'N, 2°37'W; 6–12 Dec. 1993; T Andersen & J Kjærandsen leg.; Malaise trap; UMSP 000550034. ***Paratypes*.** Ghana – **Western Reg.** ● 2♂♂; same data as for holotype; ZMBN.

**Figure 38.**
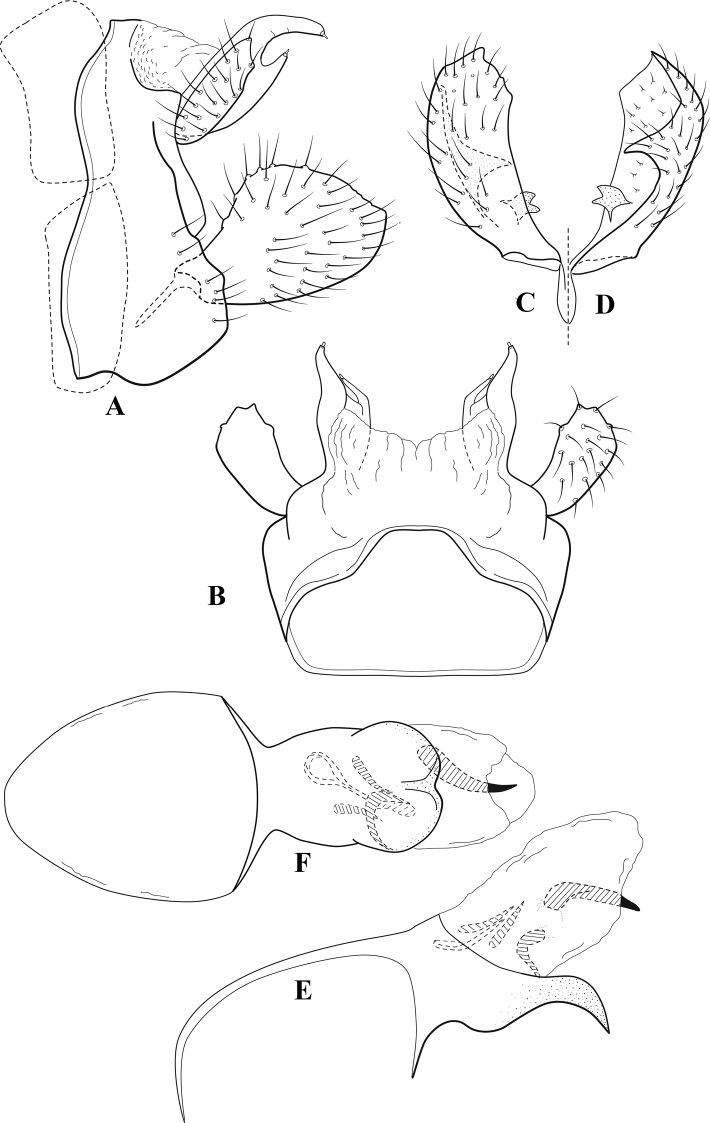
*Chimarraaurita* sp. nov., ♂ genitalia **A** lateral **B** dorsal, segments IX and X **C** inferior appendage, ventral **D** inferior appendage, dorsal **E** phallus, lateral **F** phallus, ventral.

###### Additional material.

Ghana– **Western Reg.** ● 9♀♀; Ankasa Game Production Reserve; 5°15'N 2°37'W; 6–12 Dec. 1993; T Andersen & J Kjærandsen leg.; Malaise trap; ZMBN ● 1♀; same collection data as for preceding; UMSP.

###### Diagnosis.

*Chimarraaurita* is distinctive because of its enlarged and ear-like preanal appendages. As in other species of the subgroup, *C.aurita* is most readily diagnosed by characters of the inferior appendages, tergum X and phallobase in combination. The inferior appendages are short and ovate, with a mesally curved and spine-like projection on its dorsal margin and a short bifid cusp on its basomesal margin. The short lateral lobes of tergum X each terminates in a pair of digitate, sensillum-bearing processes, a longer ventrally curved one on its dorsal margin and a much shorter, dorsally-projecting process on its ventral margin. The short phallobase has a decurrent apex, appearing spine-like in lateral view, but with the apex actually broad, as viewed ventrally, and not divided mesally (or at most only weaky notched).

###### Description.

***Adult.*** Overall color (in alcohol) light brown or yellowish brown, undersides and appendages paler, setal warts of head not contrasting. Head short and rounded (postocular parietal sclerite short). Palps elongate; maxillary palp with 1^st^ segment very short (approximately as long as wide), 2^nd^ segment moderate in length (~ 3× 1^st^), apex with cluster of ~ 8 stiff setae, 3^rd^ segment elongate, distinctly longer than 2^nd^, 4^th^ segment short (shorter than 2^nd^), 5^th^ segment very elongate and narrow (subequal to 3^rd^ and 4^th^ combined). Forewing length: male, 3.8–4.9 mm; female, 4.3–4.7 mm. Fore- and hind wings with forks I, II, III, and V present. Forewing with R_1_ straight, stem of Rs slightly inflected, basal fork of discoidal cell distinctly enlarged, evenly forked, length of cell ~ 2× width, forks I and II subsessile, *r* crossvein diagonal, intersecting discoidal cell before apical fork, *s*, *r-m*, and *m* crossveins linear and hyaline, both 2A and 3A looped to 1A (2A without apical fork). Hind wing with R_1_ evident basally, obsolete (or fused to subcosta) apically, forks I and II subsessile, fork III distal and relatively narrow, anal loop small. Forelegs with apical tibial spur short; male with modified tarsal claws, apical three segments of tarsi short and flattened, claws asymmetrical, outer one elongate and twisted.

***Male genitalia.*** Segment VIII very short, tergum approximately same length as sternum, sternum without posteroventral projection. Segment IX, in lateral view, relatively short, ventral margin somewhat projecting posteriorly, anteroventral margin only slightly expanded, constricted basally under sternum VIII, ventral process absent, dorsal margin without apodemes, inferior appendages inserted near ventral margin; as viewed dorsally, with tergum very narrow, but continuous, sternum short, subtruncate. Tergum X with mesal lobe short and membranous, lateral lobes short and divided apically into two digitate processes, each with single apical sensillum, dorsal process more elongate and slightly ventrally curved, ventral process very short. Preanal appendages prominent and moderately large, distinctly flattened, ear-shaped, slightly constricted basally, inserted membranously (not fused to segments IX or X). Inferior appendage without evident basal inflection; as viewed laterally, more or less ovate, subangulate apically, with short lateral setae and row of spaced, more elongate setae on dorsal margin; as viewed dorsally, with short, acute, sclerotized projection on dorsomesal margin, and prominent, apically forked cusp basoventrally on mesal surface. Phallic apparatus with phallobase very short and strongly sclerotized, with usual basodorsal expansion, securely anchored within segment by semi-sclerotized periphallic membrane (attached to lateral margin of segment IX), apicoventral margin of phallobase very distinctly sclerotized and produced, down-turned, apex produced into single acute mesal projection; endotheca short, membranous, with two short, curved, thick spines, one slightly larger than the other; phallotremal sclerite complex composed of short rod and ring structure, with pair of short apicolateral sclerites.

###### Etymology.

*Chimarraaurita*, used as an adjective, from the Latin *auritus*, or eared, for the large, ear-like preanal appendages of this species.

##### ﻿ Chimarra crescentis sp. nov.

Taxon classificationAnimaliaTrichopteraPhilopotamidae

DD52A851-6E13-505B-8EF8-D96B6352A3FE

http://zoobank.org/49EB1093-6FDF-4795-B9A2-3CE3A67CF65F

Fig. 39A–F

###### Type material.

***Holotype*.** Tanzania – **Tanga Reg.** ● ♂ (in alcohol); West Usambara Mts, Dule; 4°51'S, 38°26'E; 26 Nov. 1990; T Andersen leg.; sweep net; UMSP 000550035. ***Paratypes*.** Tanzania – **Tanga Reg.** ● 1♂; same data as for holotype; ZMBN ● 1♂; INHS.

**Figure 39.**
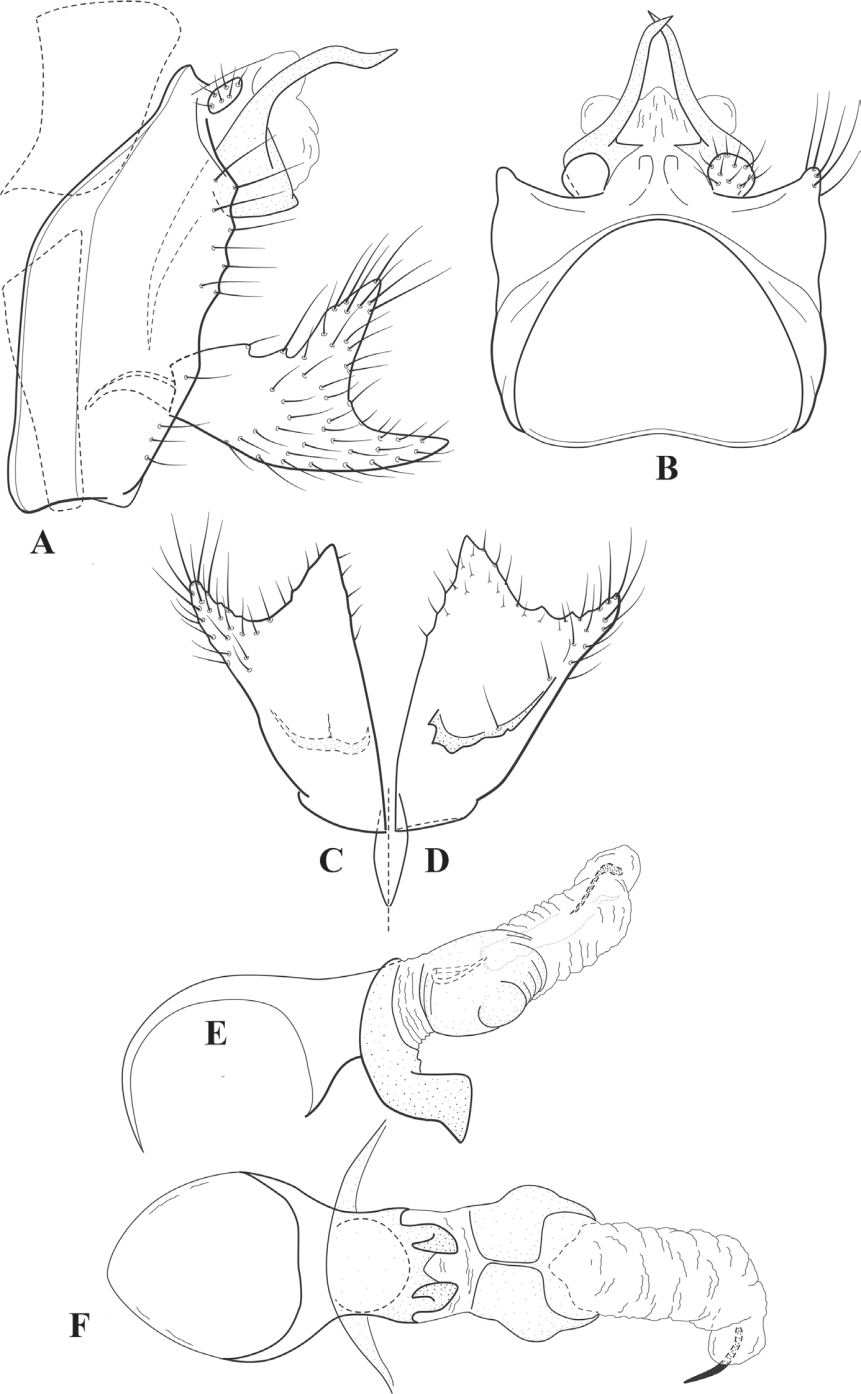
*Chimarracrescentis* sp. nov., ♂ genitalia **A** lateral **B** dorsal, segments IX and X **C** inferior appendage, ventral **D** inferior appendage, dorsal **E** phallus, lateral **F** phallus, ventral.

###### Diagnosis.

*Chimarracrescentis* is related to the other new species of this subgroup from Tanzania, resembling them in the general structure of the phallus. It is easily distinguished from any other species by the shape of its inferior appendages, which, in lateral view, have acute dorsal and ventral apices separated by a broadly crescentic invagination.

###### Description.

***Adult.*** Overall color (in alcohol) medium brown to yellowish brown, setal warts of head slightly paler, weakly contrasting. Head short (postocular parietal sclerite relatively short, shorter than eye). Palps elongate; maxillary palp with 1^st^ segment very short (approximately as long as wide), 2^nd^ segment short (~ 2× 1^st^), apex with cluster of ~ 8–10 stiff setae, 3^rd^ segment very elongate (> 2× 2^nd^), 4^th^ segment short (shorter than 2^nd^), 5^th^ segment elongate and narrow (slightly longer than 3^rd^). Forewing length: male, 4.7–5.0 mm. Fore- and hind wings with forks I, II, III, and V present. Forewing with R_1_ straight, stem of Rs straight, or nearly so, basal fork of discoidal cell slightly enlarged, evenly forked, length of cell ~ 2× width, fork I sessile, fork II slightly subsessile, *r* crossvein diagonal, intersecting discoidal cell before apical fork, *s*, *r-m*, and *m* crossveins linear and hyaline (*m* crossvein somewhat diagonal), both 2A and 3A looped to 1A (2A without apical fork). Hind wing with R_1_ evident basally, obsolete (or fused to subcosta) apically, forks I and II slightly subsessile, fork III distal and relatively wide, anal loop small. Forelegs with apical tibial spur short; male with tarsal claws not enlarged, claws symmetrical, tarsal segments narrow.

***Male genitalia.*** Segment VIII very short, tergum longer than sternum, dorsal margin slightly projecting, sternum without posteroventral projection. Segment IX, in lateral view, short, anteroventral margin only slightly expanded, dorsal margin without apodemes, sternum with very short, rounded ventral process from posterior margin, inferior appendages inserted near ventral margin; as viewed dorsally, with tergum very narrow, but continuous, sternum short, subtruncate. Tergum X with mesal lobe short and membranous, lateral lobes short and strongly sclerotized, each with rounded basal part and mesally curved, spine-like dorsal projection; sensilla of lobes absent (or not evident). Preanal appendages very short and rounded, slightly flattened, inserted membranously (not fused to segments IX or X). Inferior appendage not or only weakly inflected basally, appendage narrow basally, expanded apically to produce widely forked, subequal, acute, dorsal and ventral lobes; mesal surface with irregular sclerotized cusp in basal half, probably articulating with sclerotized ventral projection of phallobase. Phallic apparatus with phallobase very short and strongly sclerotized, with usual basodorsal expansion, securely anchored within segment by sclerotized periphallic membrane (and apparently fused to it); apicoventral margin of phallobase (or possibly projections from periphallic membrane) very distinctly sclerotized and produced, down-turned, apex divided mesally, apparently articulating with cusped projections of mesal surface of inferior appendages; phallic apparatus distal to sclerotized ventral projection (possibly modified endotheca), with lightly sclerotized membranous region and bulbous sclerotized projection with rounded apical lobes (appearing as extension of phallobase); endotheca with single short, curved apical spine; phallotremal sclerite complex composed of short rod and ring structure, rod very short.

###### Etymology.

*Chimarracrescentis*, used as an adjective, for the crescentic apex of the inferior appendages in this species (derived via OF from the Latin *cresco*, to grow, for the figure of the moon in its first or last quarter).

##### ﻿ Chimarra indicis sp. nov.

Taxon classificationAnimaliaTrichopteraPhilopotamidae

A65BB97D-4395-5E04-8A61-5E7A9EB9847F

http://zoobank.org/D31D52C1-11C9-464D-A98B-85A18BC69929

Fig. 40A–F

###### Type material.

***Holotype*.** Ghana – **Western Reg.** ● ♂ (in alcohol); Ankasa Game Production Reserve; 5°15'N, 2°37'W; 6–12 Dec. 1993; T Andersen & J Kjærandsen leg.; Malaise trap; UMSP 000550036. ***Paratypes*.** Ghana – **Western Reg.** ● 4♂♂; same data as for holotype; ZMBN. – **Central Reg.** ● 1♂; Kakum Forest Reserve; 5°21'N, 1°22'W; 8–15 Nov. 1994; T Andersen leg.; Malaise trap; ZMBN.

**Figure 40.**
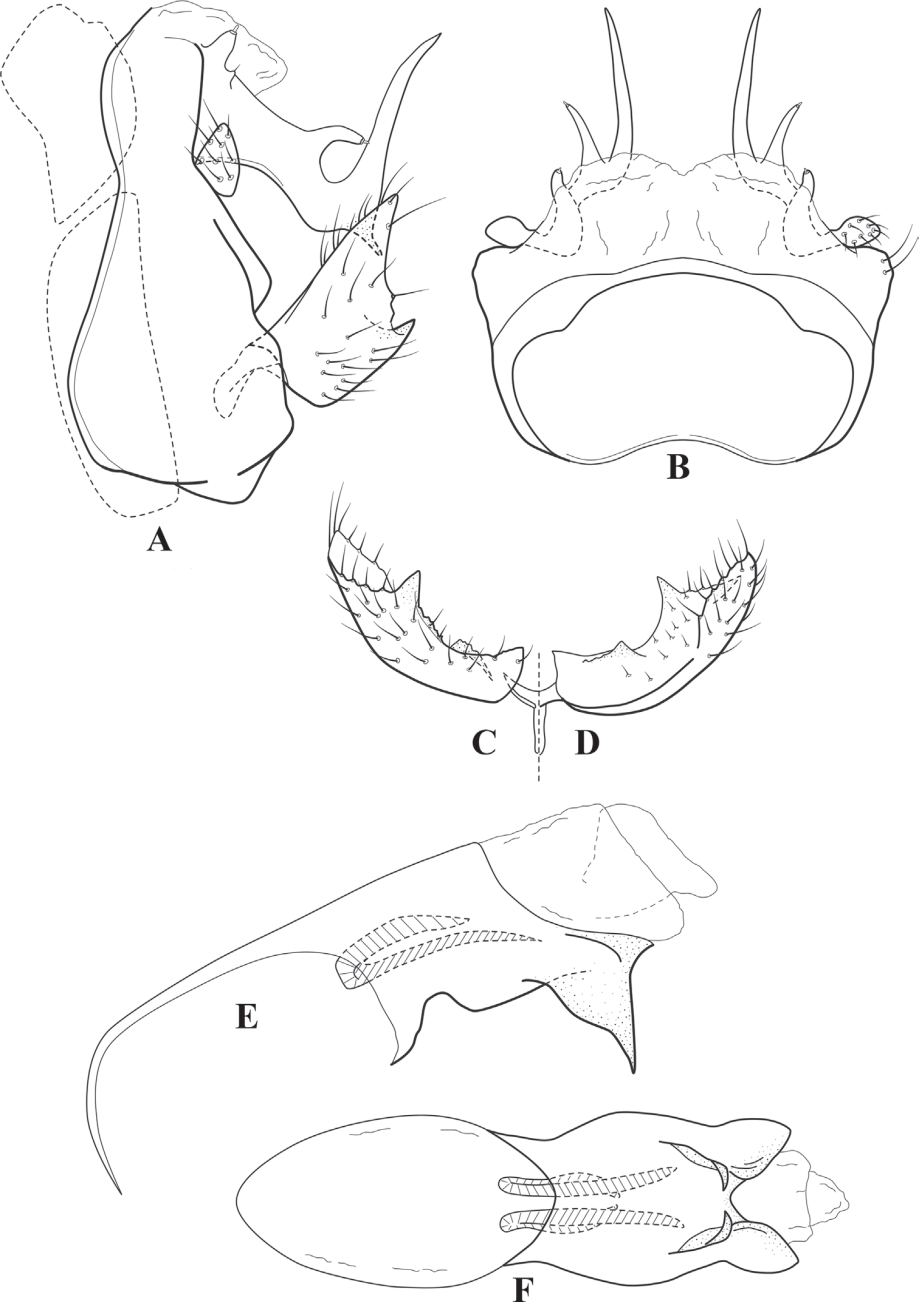
*Chimarraindicis* sp. nov., ♂ genitalia **A** lateral **B** dorsal, segments IX and X **C** inferior appendage, ventral **D** inferior appendage, dorsal **E** phallus, lateral **F** phallus, ventral.

###### Additional material.

Ghana – **Western Reg.** ● 1♀; Ankasa Game Production Reserve; 5°15'N, 2°37'W; 6–12 Dec. 1993; T Andersen & J Kjærandsen leg.; Malaise trap; UMSP.

###### Diagnosis.

*Chimarraindicis* appears to be somewhat similar to *C.georgenis* Barnard, particularly in a having an acute cusp on the ventral margin of the inferior appendage and in having each of the lateral lobes of tergum X with an elongate and short digitate process near its apex. A direct comparison is difficult because the genitalia of *C.georgensis* was drawn as fragmented parts. In general, *C.georgensis* is probably more directly related to *C.uncinata* sp. nov. and *C.serrella* sp. nov. because of the similarity in the structure of the phallobase in those species, with the apex decurrent and with two distinct apical points on each side and an acute projection on the dorsal margin where the apex begins to bend downward. Besides the differently formed apex of the phallobase in *C.indicis*, it also differs from *C.georgensis* in that the inferior appendage has an acute dorsal projection, and the lateral lobes of tergum X have a greater overall length, each lobe with two sensilla on short digitate processes, one basal and one curved and preapical, just before the elongate, acute, dorsally recurved apical projection.

###### Description.

***Adult.*** Overall color (in alcohol) light brown, undersides and appendages yellowish brown, vertex of head slightly darker, setal warts of head not distinctly contrasting. Head short (postocular parietal sclerite short). Palps elongate; maxillary palp with 1^st^ segment very short (approximately as long as wide), 2^nd^ segment moderately elongate (~ 3× 1^st^), apex with cluster of ~ 6 setae, 3^rd^ segment elongate, distinctly longer than 2^nd^, 4^th^ segment short (shorter than 2^nd^), 5^th^ segment very elongate and narrow (subequal to 3^rd^ and 4^th^ combined). Forewing length: male, 3.5–4.0 mm. Fore- and hind wings with forks I, II, III, and V present. Forewing with R_1_ straight, stem of Rs straight, or nearly so, basal fork of discoidal cell slightly enlarged, evenly forked, length of cell ~ 2× width, fork I subsessile, fork II sessile, *r* crossvein diagonal, intersecting discoidal cell near apical fork, *s*, *r-m*, and *m* crossveins linear and hyaline, both 2A and 3A looped to 1A (2A without apical fork). Hind wing with R_1_ obsolete (or fused to subcosta), forks I and II subsessile, fork III distal and relatively narrow, anal loop small. Forelegs with apical tibial spur very short; male with modified tarsal claws, apical three segments of tarsi short and flattened, claws asymmetrical, outer one elongate and twisted.

***Male genitalia.*** Segment VIII very short, tergum approximately same length as sternum, sternum without posteroventral projection. Segment IX, in lateral view, short, anteroventral margin only slightly expanded, dorsal margin without apodemes, ventral process very short, subtriangular, ventrally oriented, inferior appendages inserted near ventral margin; as viewed dorsally, with tergum very narrow, but continuous, sternum short, subtruncate. Tergum X with mesal lobe very short and membranous, lateral lobes projecting, distinctly sclerotized, produced apically into strongly dorsally curved spine-like projection (bent at approximately right angle), dorsal projection subequal to length of base before inflection, ventral margin of inflection with additional small spine; sensilla of lobes apparently reduced to two, on short nipple-like projections, one basodorsally and one dorsolaterally, before apical bend. Preanal appendages short and rounded, distinctly flattened, inserted membranously (not fused to segments IX or X). Inferior appendage with distinct basal inflection; as viewed laterally, narrow, with short, sclerotized projection from apex of ventral margin, appendage dorsally inflected and tapering from ventral projection, apex acute; as viewed ventrally, with acute sclerotized cusp on posterior margin (extended onto mesal surface), and additional small, irregular cusp basally on mesal surface. Phallic apparatus with phallobase very short and strongly sclerotized, with usual basodorsal expansion, securely anchored within segment by semi-sclerotized periphallic membrane (attached to lateral margin of segment IX), apicoventral margin of phallobase very distinctly sclerotized and produced, down-turned, apex divided into pair of ventrally projecting spine-like processes; endotheca short, membranous, internally with pair of prominent recurved sclerites (possibly modified elements of phallotremal sclerite complex).

###### Etymology.

*Chimarraindicis*, used as a noun in apposition, from the Latin *index*, a sign, token, or forefinger, for the notable upturned apex of tergum X in this species.

##### ﻿ Chimarra latidentis sp. nov.

Taxon classificationAnimaliaTrichopteraPhilopotamidae

7B0E6EA3-F991-5692-8BA2-E26FE80D6405

http://zoobank.org/3FAAEA83-9E81-41A9-9B86-8576F8A1F33A

Fig. 41A–F

###### Type material.

***Holotype*.** Tanzania – **Tanga Reg.** ● ♂ (in alcohol); West Usambara Mts, Mazumbai, Kaputu Stream; 4°48'S, 38°30'E; 27–28 Oct. 1990; T Andersen leg.; Malaise trap; UMSP 000550038. ***Paratypes*.** Tanzania – **Tanga Reg.** ● 34♂♂; same data as for holotype except 27 Oct. 1990–12 Feb. 1991; ZMBN ● 1♂; West Usambara Mts, Shokoi River; 4°46'S, 38°29'E; 24 Nov. 1990; T Andersen leg.; sweep net; UMSP.

**Figure 41.**
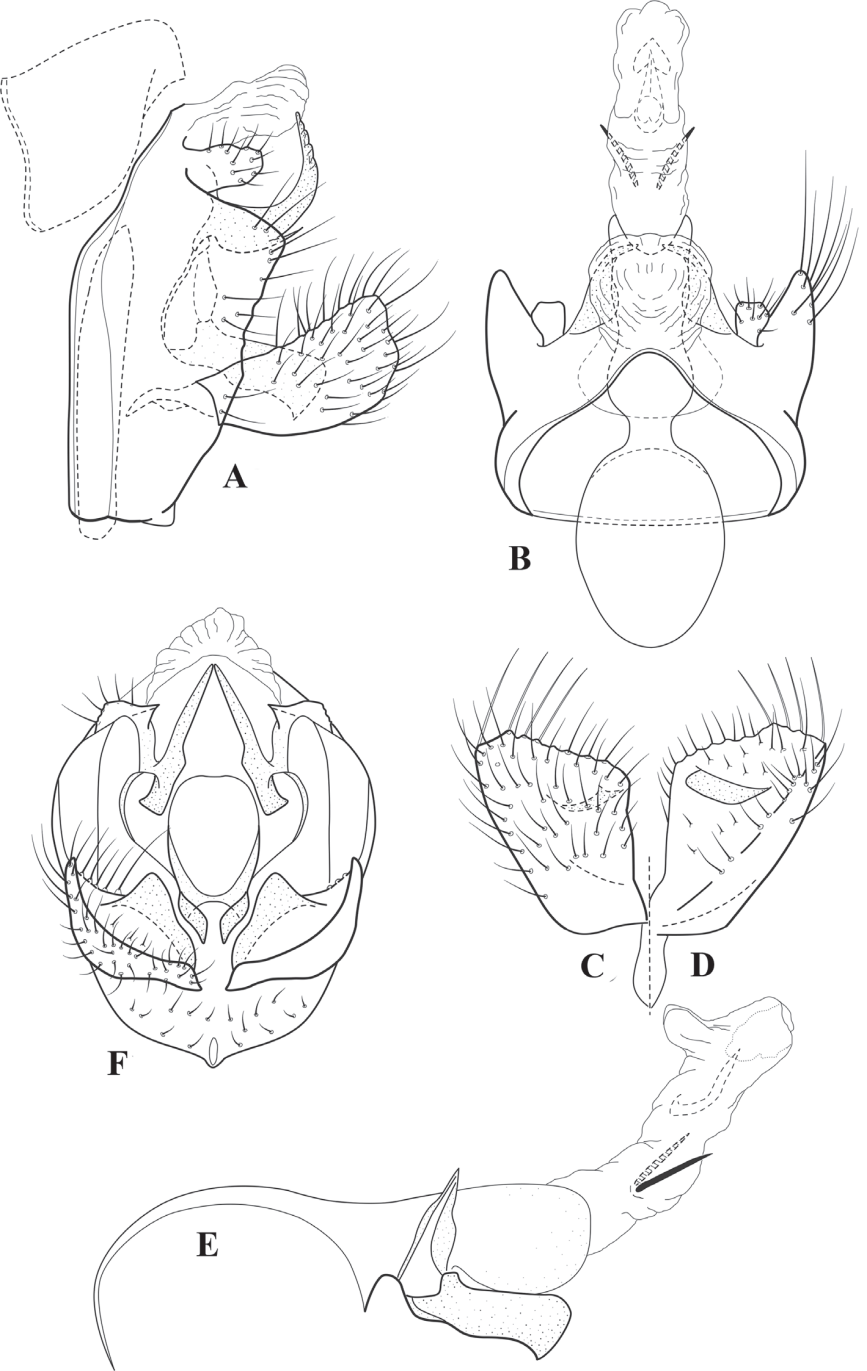
*Chimarralatidentis* sp. nov., ♂ genitalia **A** lateral **B** dorsal, segments IX, X, and phallus **C** inferior appendage, ventral **D** inferior appendage, dorsal **E** phallus, lateral **F** caudal.

###### Diagnosis.

*Chimarralatidentis* is closely related to *C.leptodactylus* sp. nov. and *C.vermitergata* sp. nov. It is most similar to *C.vermitergata* in having the lateral lobes of tergum X formed into upright, spine-like processes, without a separated ventral projection. At least in the holotype of *C.latidentis*, these lobes are thicker, undivided and have their apices somewhat scabrous. The mesal cusps of the inferior appendages in *C.latidentis* are also wider, larger, and more prominent than in *C.vermitergata*, and the basal sclerotized portion of the endotheca, posterior to the sclerotized ventral apex of the phallobase, is more rounded.

###### Description.

***Adult.*** Overall color (in alcohol) medium brown to yellowish brown, head darker (dark brown), setal warts of head paler and somewhat contrasting. Head short (postocular parietal sclerite relatively short, shorter than eye). Palps elongate; maxillary palp with 1^st^ segment very short (approximately as long as wide), 2^nd^ segment short (~ 2× 1^st^), apex with cluster of ~ 8 stiff setae, 3^rd^ segment very elongate (nearly 3× 2^nd^), 4^th^ segment short (shorter than 2^nd^), 5^th^ segment elongate and narrow (subequal to 3^rd^). Forewing length: male, 5.0–6.0 mm. Fore- and hind wings with forks I, II, III, and V present. Forewing with R_1_ straight, stem of Rs straight, or nearly so, basal fork of discoidal cell slightly enlarged, evenly forked, discoidal cell relatively elongate and narrow, length of cell nearly 3× width, fork I with long stem, fork II subsessile, *r* crossvein diagonal, intersecting discoidal cell near *s* crossvein, *s*, *r-m*, and *m* crossveins hyaline, *s* and *r-m* linear, *m* crossvein somewhat proximal and diagonal, both 2A and 3A looped to 1A (2A without apical fork). Hind wing with R_1_ obsolete (or fused to subcosta), fork I with very short stem, fork II subsessile, fork III distal and very narrow, anal loop small. Forelegs with apical tibial spur short; male with tarsal claws not enlarged, claws symmetrical, tarsal segments narrow.

***Male genitalia.*** Segment VIII with sternum very short, tergum ~ 2× as long, dorsal margin projecting, sternum without posteroventral projection. Segment IX, in lateral view, short, anteroventral margin only slightly expanded, anterodorsal margin without apodemes, posterior margin angularly projecting below preanal appendages, sternum with very short, rounded ventral process from posterior margin, inferior appendages inserted somewhat above ventral margin; as viewed dorsally, with tergum very narrow, but continuous (or nearly so), sternum very short, subtruncate. Tergum X with mesal lobe short and membranous, lateral lobes short and sclerotized, each modified into short, upturned spine-like projection from basoventral margin, apex of projection rugose; sensilla of lobes absent (or not evident). Preanal appendages very short and rounded, slightly flattened, inserted membranously (not fused to segments IX or X). Inferior appendage with weak basal inflection; as viewed laterally, short, with apicodorsal margin somewhat angulate and laterally projecting; as viewed ventrally, subtruncate apically, with mesal margins of opposite appendages proximate, then sharply bent; mesal surface with wide, sclerotized, tooth-like projection, apparently articulating with sclerotized ventral projection of phallobase. Phallic apparatus with phallobase very short and strongly sclerotized, with usual basodorsal expansion, securely anchored within segment by sclerotized periphallic membrane (and apparently fused to it); apicoventral margin of phallobase (or projections from periphallic membrane) very distinctly sclerotized and produced, down-turned, apex divided mesally, apparently articulating with tooth-like projections of mesal surface of inferior appendages; phallic apparatus distal to sclerotized ventral projection (possibly modified endotheca), forming short, sclerotized, bulbous extension of phallobase; endotheca approximately as long as phallobase, apicodorsally with pair of small membranous lobes, basally with pair of very short, symmetrically positioned spines; phallotremal sclerite complex composed of short rod and ring structure.

###### Etymology.

*Chimarralatidentis*, used as a noun in apposition, from the Latin *latus*, meaning broad or wide, and *dens*, a tooth, for the relatively large, wide tooth-like cusp on the mesal surface of the inferior appendage.

##### ﻿ Chimarra leptodactylus sp. nov.

Taxon classificationAnimaliaTrichopteraPhilopotamidae

6E86E64F-6229-5779-8036-6E7935BF7959

http://zoobank.org/6C5E426B-C19E-44CA-9FEF-52D5DBB4D3B3

Fig. 42A–F

###### Type material.

***Holotype*.** Tanzania – **Tanga Reg.** ● ♂ (in alcohol); West Usambara Mts, Mazumbai, Kaputu Stream; 4°48'S, 38°30'E; 29 Nov. – 3 Dec. 1990; T Andersen leg.; Malaise trap; UMSP 000550040. ***Paratypes*.** Tanzania – **Tanga Reg.** ● 2♂♂; same data as for holotype except 20–26. Nov. 1990; ZMBN ● 1♂; same data as for holotype except 3 Nov. 1990; sweep net; ZMBN ● 1♂; West Usambra Mts, Mgwashi, Shokoi River; 4°46'S, 38°29'E; 24 Nov. 1990; T Andersen leg.; sweep net; UMSP.

**Figure 42.**
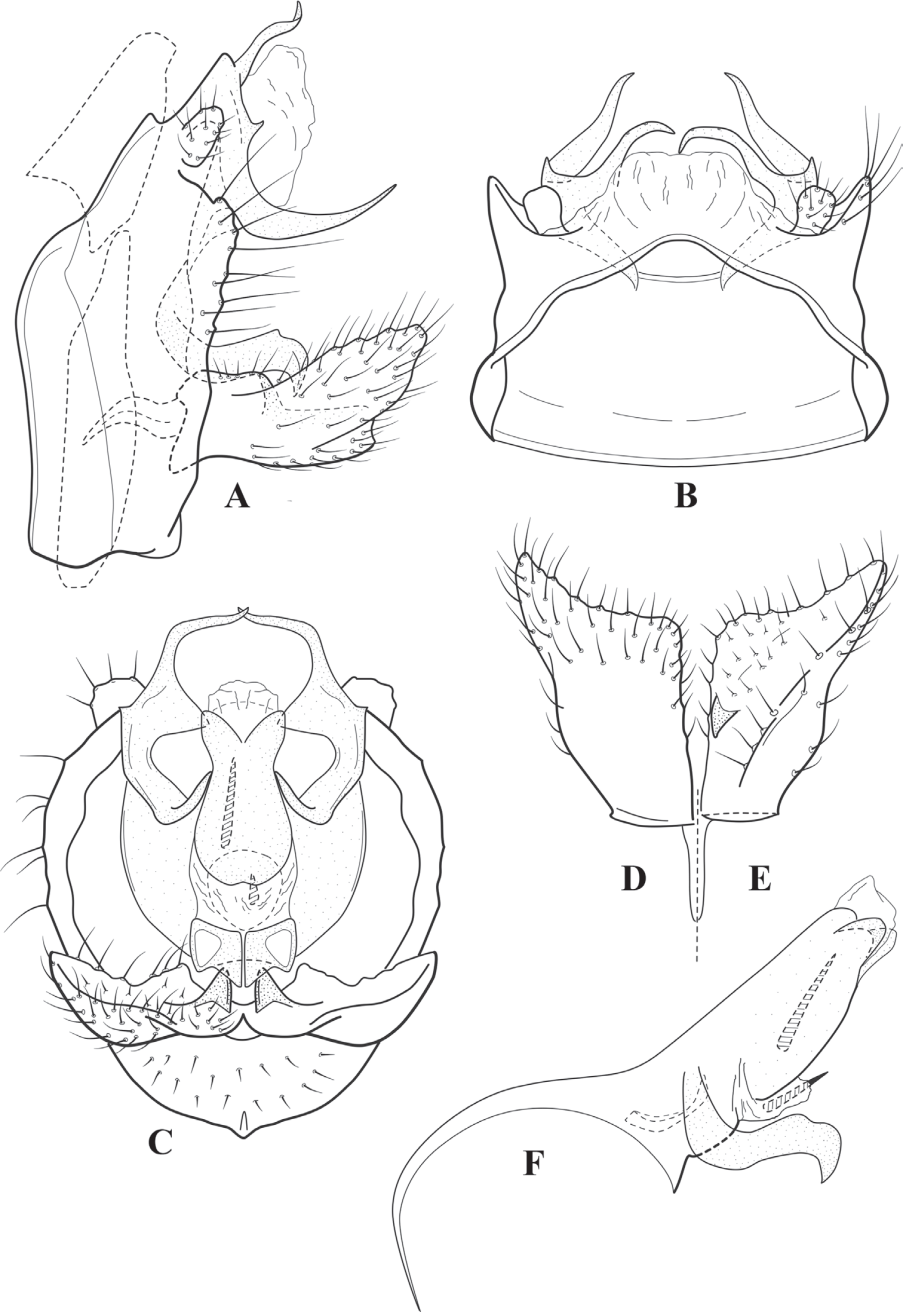
*Chimarraleptodactylus* sp. nov., ♂ genitalia **A** lateral **B** dorsal, segments IX and X **C** caudal **D** inferior appendage, ventral **E** inferior appendage, dorsal **F** phallus, lateral.

###### Diagnosis.

*Chimarraleptodactylus* is closely related to *C.latidentis* sp. nov. and *C.vermitergata* sp. nov. It is most readily diagnosed by the overall structure of the lateral lobes of tergum X, each of which has a narrow dorsomesal projection and a widely separated, acute, ventral projection, rather than a generally dorsally directed lobe or lobes.

###### Description.

***Adult.*** Overall color (in alcohol) medium brown, setal warts of head not contrasting. Head short (postocular parietal sclerite relatively short, shorter than eye). Palps elongate; maxillary palp with 1^st^ segment very short (approximately as long as wide), 2^nd^ segment short (~ 2× 1^st^), apex with cluster of ~ 8 stiff setae, 3^rd^ segment very elongate (~ 2× 2^nd^), 4^th^ segment short (shorter than 2^nd^), 5^th^ segment elongate and narrow (subequal to 3^rd^). Forewing length: male, 5.5–6.0 mm. Fore- and hind wings with forks I, II, III, and V present. Forewing with R_1_ straight, stem of Rs straight, or nearly so, basal fork of discoidal cell distinctly enlarged, evenly forked, length of cell slightly > 2× width, forks I and II sessile, *r* crossvein diagonal, intersecting discoidal cell near apical fork, *s*, *r-m*, and *m* crossveins linear and hyaline (*m* crossvein somewhat diagonal), both 2A and 3A looped to 1A (2A without apical fork). Hind wing with R_1_ evident basally, obsolete (or fused to subcosta) apically, forks I and II sessile, fork III distal and relatively narrow, anal loop small. Forelegs with apical tibial spur very short; male with tarsal claws not, or only slightly, enlarged, claws symmetrical, tarsal segments narrow.

***Male genitalia.*** Segment VIII short, tergum longer than sternum, dorsal margin projecting, sternum without posteroventral projection. Segment IX, in lateral view, very short, anteroventral margin only slightly expanded, dorsal margin without apodemes, sternum with very short, rounded ventral process from posterior margin, inferior appendages inserted near ventral margin; as viewed dorsally, with tergum very narrow, but continuous (or nearly so), sternum short, subtruncate. Tergum X with mesal lobe short and membranous, lateral lobes short and sclerotized, each divided basally into dorsal and ventral spine-like processes, ventral processes larger and posteriorly curved, with short spine-like projection from dorsal margin in basal half, dorsal ones mesally curved; sensilla of lobes very small and reduced in number (possibly only 2 on each side, on apical half of dorsal lobe). Preanal appendages very short and rounded, slightly flattened, inserted membranously (not fused to segments IX or X). Inferior appendage with weak basal inflection; as viewed laterally, with apices narrowed and laterally projecting; as viewed ventrally, with mesal margins of opposite appendages proximate, then sharply bent, with apices narrowing and laterally projecting; mesal surface with distinctly sclerotized, anteriorly projecting, spine-like projection in basal part, apparently articulating with sclerotized ventral projection of phallobase. Phallic apparatus with phallobase very short and strongly sclerotized, with usual basodorsal expansion, securely anchored within segment by sclerotized periphallic membrane (and apparently fused to it); apicoventral margin of phallobase (or projections from periphallic membrane) very distinctly sclerotized and produced, downturned, apex truncate and narrowly divided mesally, apparently articulating with spine-like projections of mesal surface of inferior appendages; phallic apparatus distal to sclerotized ventral projection (possibly modified endotheca), lightly sclerotized, tube-like, and narrowing, with short rounded apical lobes; endotheca with pair of short, asymmetrically positioned spines; phallotremal sclerite complex composed of short rod and ring structure.

###### Etymology.

*Chimarraleptodactylus*, used as a noun in apposition, from the Greek words *leptos*, meaning thin, fine, small, or slender, and *daktylos*, a finger, for the narrow dorsal projection from each of the lateral lobes of tergum X.

##### ﻿ Chimarra obuncata sp. nov.

Taxon classificationAnimaliaTrichopteraPhilopotamidae

B50A4158-81E2-5F3A-A3F1-C40515F8950A

http://zoobank.org/039AE4DE-6783-430F-84EA-11FEFD4D99D7

Fig. 43A–G

###### Type material.

***Holotype*.** Ghana – **Western Reg.** ● ♂ (in alcohol); Ankasa Game Production Reserve; 5°15'N, 2°37'W; 7–11 Dec. 1993; T Andersen & J Kjærandsen leg.; Malaise trap; UMSP 000550042. ***Paratype*.** Ghana – **Western Reg.** ● 1♂; same data as for holotype; ZMBN.

**Figure 43.**
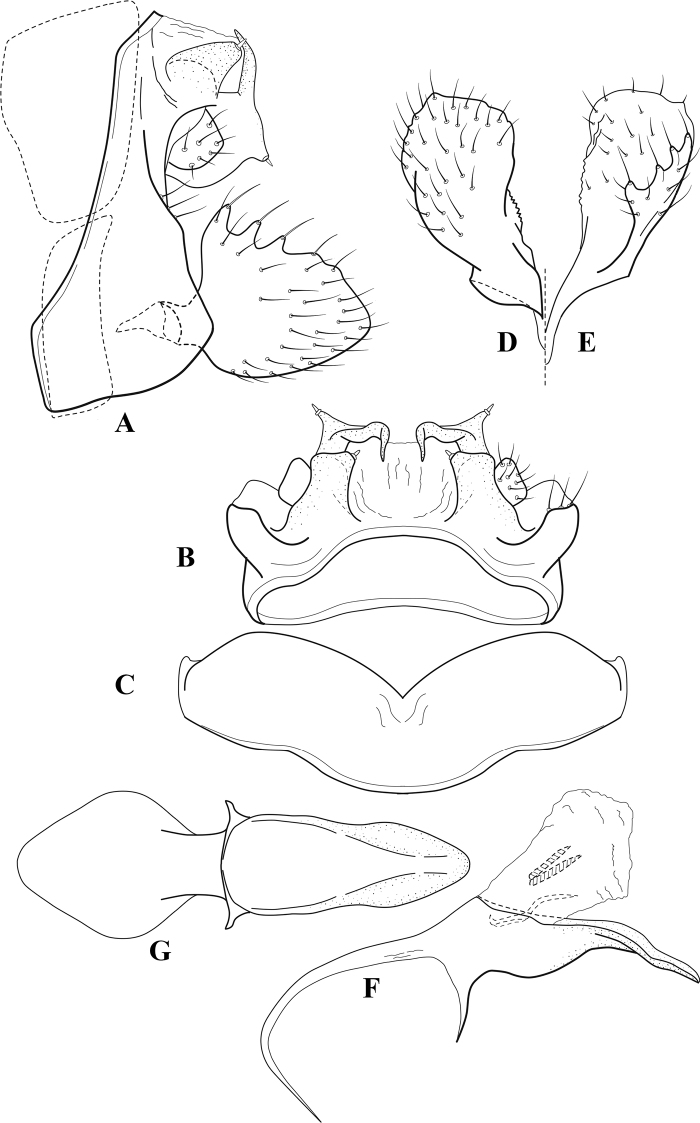
*Chimarraobuncata* sp. nov., ♂ genitalia **A** lateral **B** dorsal, segments IX and X **C** tergum VIII, dorsal **D** inferior appendage, ventral **E** inferior appendage, dorsal **F** phallus, lateral **G** phallus, ventral.

###### Additional material.

Ghana – **Western Reg.** ● 7♀♀; Ankasa Game Production Reserve; 5°15'N, 2°37'W; 6–12 Dec. 1993; T Andersen & J Kjærandsen leg.; Malaise trap; ZMBN.

###### Diagnosis.

*Chimarraobuncata* is easily identified by its short, rounded inferior appendages and the structure of tergum X, with its short sensillum-bearing processes. The structure of the phallobase, which is very short and has the apex tapered and subacute apically also distinguishes it from other species of the subgroup.

###### Description.

***Adult.*** Overall color (in alcohol) yellowish brown. Head short (postocular parietal sclerite short). Palps elongate; maxillary palp with 1^st^ segment very short (approximately as long as wide), 2^nd^ segment moderately elongate (distinctly shorter than 3^rd^), apex with cluster of stiff setae, 3^rd^ segment elongate, 4^th^ segment relatively short (shorter than 2^nd^), 5^th^ segment very elongate and narrow (subequal to 3^rd^ and 4^th^ combined). Forewing length: male, 3.5–3.8 mm; female, 3.7–4.5mm. Fore- and hind wings with forks I, II, III, and V present. Forewing with R_1_ straight, stem of Rs straight, or nearly so, basal fork of discoidal cell slightly enlarged, length of cell ~ 2× width, forks I and II subsessile, *r* crossvein diagonal, intersecting discoidal cell near apical fork, *s*, *r-m*, and *m* crossveins linear and hyaline, both 2A and 3A looped to 1A (2A without apical fork). Hind wing with R_1_ faintly evident basally, obsolete or fused to subcosta distally, forks I and II subsessile, fork III distal and narrow, anal loop small. Forelegs with apical tibial spur short; male with modified tarsal claws, apical 2 segments of tarsi enlarged and flattened, claws asymmetrical, outer one elongate and twisted.

***Male genitalia.*** Segment VIII with sternum very short, without posteroventral projection, tergum moderately expanded dorsally (~ 2× length of sternum at base), dorsomesally with broad V-shaped emargination from posterior margin. Segment IX, in lateral view, short, segment widest ventrally, nearly linearly narrowing dorsally from approximately ventral ¼, dorsum very short, without apodemes; posterior margin not or scarcely produced, ventral process absent, inferior appendages inserted near ventral margin; as viewed dorsally, with tergum very narrow, but continuous, sternum very short, subtruncate. Lateral lobes of tergum X very short, each produced as pair of short, rounded dorsal and ventral sensillum-bearing processes, basodorsal pair mound-like, with apical sensilla somewhat mesally oriented, ventral pair more elongate, with apicoventral sensillum of each on short nipple-like process and somewhat laterally oriented, dorsal margin of ventral processes each with curved, dorsally directed, spine-like projection, dorsal lobe of tergum X short, membranous. Preanal appendages short and rounded, not (or hardly) constricted basally, slightly flattened, inserted membranously (not fused to segments IX or X). Inferior appendage without basal inflection; as viewed laterally, very short, rounded, basodorsal margin with short nipple-like projections, each bearing stout apical seta, mesal surface without projections. Phallic apparatus with phallobase very short, with usual basodorsal expansion, apicoventral margin very distinctly sclerotized and produced, somewhat downturned, subacutely narrowed, as viewed dorsally or ventrally; endotheca short, membranous, with two very small spines; phallotremal sclerite complex composed of short, reclinate ring and short rod.

###### Etymology.

*Chimarraobuncata*, used as an adjective, from the Latin *obuncus*, meaning bent in or hooked, for the acute dorsal process of the very short tergum X in this species, which is dorsally projected and strongly mesally hooked inward.

##### ﻿ Chimarra polycentropoides sp. nov.

Taxon classificationAnimaliaTrichopteraPhilopotamidae

5A61A397-21D0-56A9-89C4-DFF828E9176D

http://zoobank.org/4B2DF6B6-A576-4D3E-94BB-D6ECD44D13BC

Fig. 44A–E

###### Type material.

***Holotype*.** Democratic Republic Of The Congo ● ♂ (in alcohol); S slope of Mt Kahuzi; 5 Sept. 1957; 1.900 m a.s.l.; ES Ross & RE Leech leg.; INHSTrichoptera 50340.

**Figure 44.**
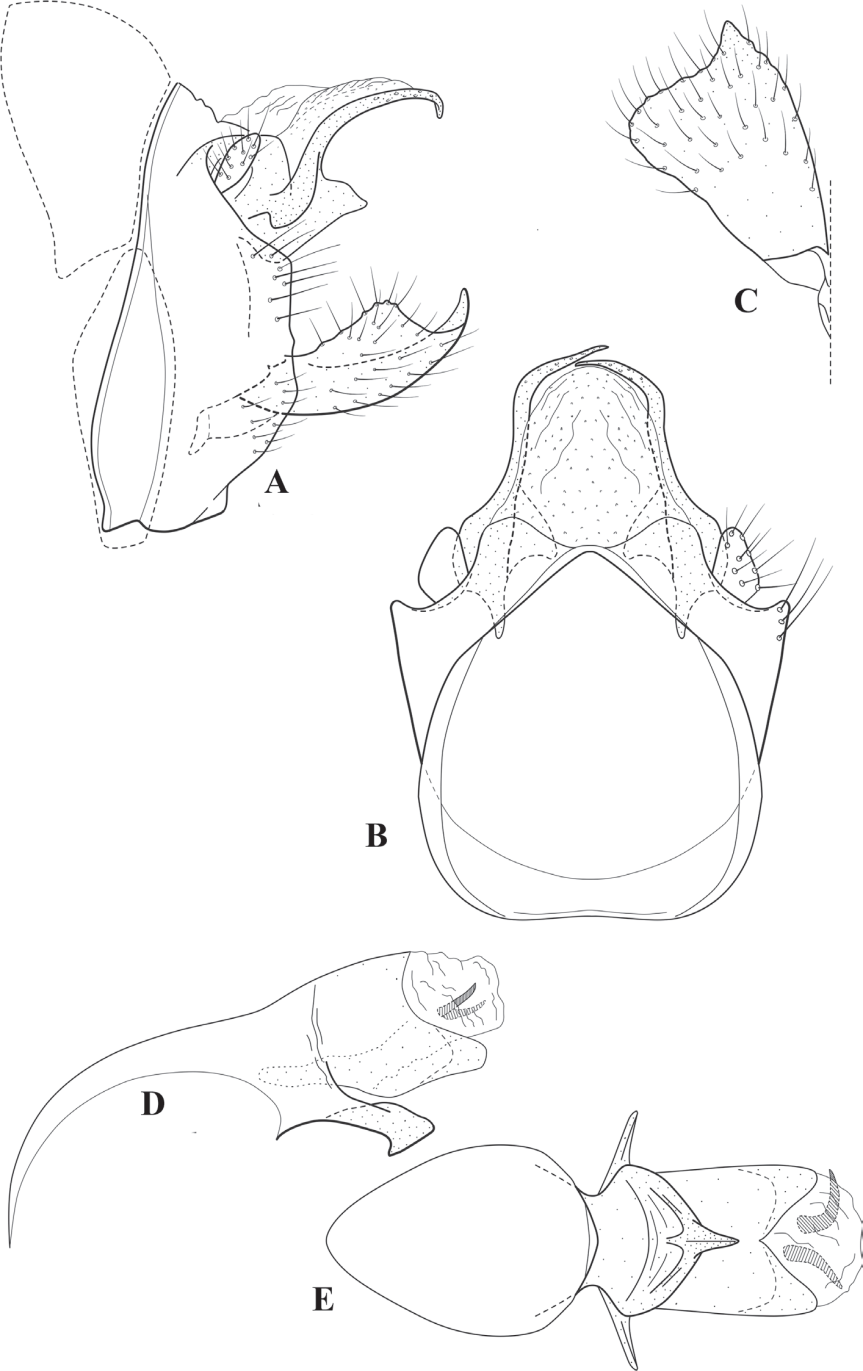
*Chimarrapolycentropoides* sp. nov., ♂ genitalia **A** lateral **B** dorsal, segments IX and X **C** inferior appendage, ventral **D** phallus, lateral **E** phallus, ventral.

###### Diagnosis.

*Chimarrapolycentropoides*, while similar in general aspects to other species of the *georgensis* subgroup, is distinctive because of its arched and spine-like lateral lobes of tergum X, and in having the ventromesal apex of the inferior appendage acutely produced, appearing as an upturned projection in lateral view. The general structure of the phallus and the absence of modified tarsal claws suggests a relationship to the species in the subgroup from Tanzania.

###### Description.

***Adult.*** Overall color (in alcohol) dark brown. Head short (postocular parietal sclerite ~ 1/2 diameter of eye). Palps elongate; maxillary palp with 1^st^ segment very short (approximately as long as wide), 2^nd^ segment short (~ 2× 1^st^), apex with cluster of ~ 8–10 stiff setae, 3^rd^ segment very elongate (almost 3× 2^nd^), 4^th^ segment short (shorter than 2^nd^), 5^th^ segment elongate and narrow (slightly longer than 3^rd^). Forewing length: male, 6.7 mm. Fore- and hind wings with forks I, II, III, and V present. Forewing with R_1_ straight, stem of Rs straight, or nearly so, basal fork of discoidal cell slightly enlarged, evenly forked, length of cell ~ 2× width, forks I and II sessile, *r* crossvein diagonal, intersecting discoidal cell before apical fork, *s*, *r-m*, and *m* crossveins linear and hyaline (*m* crossvein very slightly proximal), both 2A and 3A looped to 1A (2A without apical fork). Hind wing with R_1_ evident basally, obsolete (or fused to subcosta) apically, forks I and II sessile, anal loop small. Forelegs with apical tibial spur distinct; male with foretarsi not, or very little, modified, claws small and symmetrical.

***Male genitalia.*** Segment VIII with sternum very short, without posteroventral projection, tergum moderately expanded dorsally. Segment IX, in lateral view, short, anterior margin nearly linear, without dorsolateral apodemes; segment very short dorsally, widening below preanal appendages, ventral process very small, subtriangular, not projecting, emerging from base of segment below inferior appendages; as viewed dorsally, with tergum very narrow, but continuous, anterior margin of sternum subtruncate. Lateral lobes of tergum X modified into elongate, arched, spine-like processes, apices slightly scabrous, ventral margin developed into short projecting lobe; mesal lobe of tergum X membranous, slightly textured, nearly as long as lateral lobes. Preanal appendages short, rounded, slightly constricted basally. Inferior appendage, in lateral view, without basal inflection, dorsal margin weakly produced at midlength, apex acutely projecting and upturned; as viewed ventrally, subquadrate, moderately widened apically, with apicomesal margin acutely produced. Phallic apparatus with phallobase very short, with usual basodorsal expansion, apicoventral margin very distinctly sclerotized, not bifid, forming a short, acute, preapical projection from ventral margin, as viewed laterally; endotheca, apparently, with basal part forming a weakly sclerotized extension of phallobase, apical membrane very short, with pair of short, symmetric spines; phallotremal sclerite complex not evident.

###### Etymology.

*Chimarrapolycentropoides*, name used as an adjective, for resemblance of this species to those in the genus *Polycentropus*, due to the similarity of the lateral lobes of tergum X to the dorsal spine-like structures of some species in that genus.

##### ﻿ Chimarra ralphi sp. nov.

Taxon classificationAnimaliaTrichopteraPhilopotamidae

56B733A1-2A28-5C4A-9E1B-FA1B41808039

http://zoobank.org/44E09354-0A8F-4932-BABA-7ADE04C845AC

Fig. 45A–F

###### Type material.

***Holotype*.** Ghana – **Volta Reg.** ● ♂ (in alcohol); Wli, Agumatsa waterfall, station # 6; 7°07'29"N, 0°35'31"E; 11 Mar. 1993; JS Amakye & J Kjærandsen leg.; light trap; UMSP 000550041. ***Paratypes*.** Ghana – **Western Reg.** ● 2♂♂; Ankasa Game Production Reserve; 5°15'N, 2°37'W; 6–12 Dec. 1993; T Andersen & J Kjærandsen leg.; Malaise trap; ZMBN.

**Figure 45.**
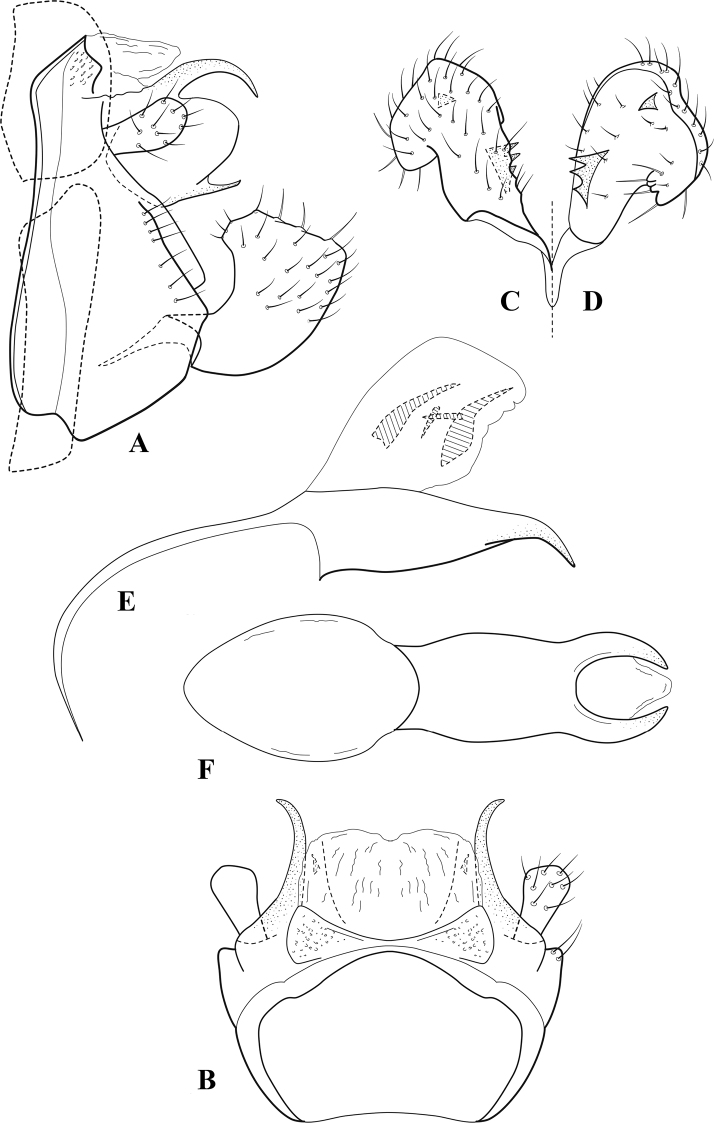
*Chimarraralphi* sp. nov., ♂ genitalia **A** lateral **B** dorsal, segments IX and X **C** inferior appendage, ventral **D** inferior appendage, dorsal **E** phallus, lateral **F** phallus, ventral.

###### Additional material.

Ghana – **Volta Reg.** ● 1♀; Wli, Agumatsa waterfall, station # 6; 7°07'29"N, 0°35'31"E; 11–20 Nov. 1993; J Kjærandsen leg.; Malaise trap; ZMBN. – **Western Reg.** ● 2♀♀; Ankasa Game Production Reserve; 5°15'N, 2°37'W; 6–12 Dec. 1993; T Andersen & J Kjærandsen leg.; Malaise trap; ZMBN ● 1♀; same collection data as for preceding; UMSP.

###### Diagnosis.

*Chimarraralphi* is diagnosed by the unusual shape of its inferior appendages, each of which is very short, with the base rounded and with a short blunt, recurved projection on its dorsal margin; by the shape of the lateral lobes of tergum X, which are short and rounded apically, each with a short, ventrally curved spine-like projection on its dorsal margin and even shorter spine-like projection from its ventral margin; and by the form of the phallobase, with its apex deeply divided mesally and rather weakly down-curved.

###### Description.

***Adult.*** Overall color (in alcohol) light brown or yellowish brown, undersides and appendages paler, setal warts of head not contrasting. Head short and rounded (postocular parietal sclerite very short). Palps moderately elongate; maxillary palp with 1^st^ segment very short (approximately as long as wide), 2^nd^ segment relatively short (slightly greater than 2× width), apex with cluster of ~ 8 stiff setae, 3^rd^ segment elongate (distinctly longer than 2^nd^), 4^th^ segment short (subequal to 2^nd^), 5^th^ segment elongate and narrow (subequal to 3^rd^ and 4^th^ combined). Forewing length: male, 3.2–4.0 mm; female, 4.0–4.5 mm. Fore- and hind wings with forks I, II, III, and V present. Forewing with R_1_ straight, stem of Rs very slightly inflected, without node, basal fork of discoidal cell distinctly enlarged, evenly forked, length of cell ~ 2× width, forks I and II both subsessile, *r* crossvein diagonal, intersecting discoidal cell near apical fork, *s*, *r-m*, and *m* crossveins linear and hyaline, both 2A and 3A looped to 1A (2A without apical fork). Hind wing with R_1_ obsolete (or fused to subcosta), forks I and II subsessile, fork III distal and relatively narrow, anal loop small. Forelegs with apical tibial spur short; male with modified tarsal claws, apical three segments of tarsi short and flattened, claws asymmetrical, outer one elongate and twisted.

***Male genitalia.*** Segment VIII very short, tergum only slightly longer than sternum, sternum without posteroventral projection. Segment IX, in lateral view, relatively short, ventral margin distinctly projecting posteriorly, anteroventral margin only slightly expanded, constricted basally under sternum VIII, ventral process absent, dorsal margin without apodemes, inferior appendages inserted near ventral margin; as viewed dorsally, with tergum very narrow, but continuous, sternum short, subtruncate. Tergum X with mesal lobe short and membranous, lateral lobes short and rounded apically, lightly sclerotized, except ventral margin with more strongly sclerotized, spine-like process at approximately midlength and dorsal margin with projecting, sclerotized, hooked, spine-like process; sensilla of lobes apparently reduced to two on each side, on mesal surface of dorsal process, one basally and one at approximately midlength. Preanal appendages relatively short and knob-like (constricted basally), distinctly flattened, inserted membranously (not fused to segments IX or X). Inferior appendage with rounded basal inflection; as viewed laterally, short, more or less ovate, with short rounded dorsal projection at approximately midlength, basal inflection of projection very strong (nearly perpendicular), mesal margin of appendage with two distinct sclerotized cusps, one preapically and very small, the other basoventrally and somewhat larger. Phallic apparatus with phallobase very short and strongly sclerotized, with usual basodorsal expansion, securely anchored within segment by semi-sclerotized periphallic membrane (attached to lateral margin of segment IX), apicoventral margin of phallobase very distinctly sclerotized and produced, down-turned, apex with deep, concave mesal excavation, producing paired apical lobes; endotheca short, membranous, with pair of short, stout, sclerotized spines; phallotremal sclerite complex indistinct (not figured), apparently composed of reclinate ring and short rod, with pair of small apical spines.

###### Etymology.

*Chimarraralphi*, name used as an adjective in the genitive case and translated as Ralph’s *Chimarra*, in honor of Ralph Holzenthal, the subject of this commemorative issue and in recognition of his many contributions to the systematics of Trichoptera, including many collaborations with both authors.

##### ﻿ Chimarra serrella sp. nov.

Taxon classificationAnimaliaTrichopteraPhilopotamidae

3F3E5DBE-BE63-5EE3-BE51-B13370AB5139

http://zoobank.org/2523BCC1-2198-43C3-AA13-634041FE0CAA

Fig. 46A–F

###### Type material.

***Holotype*.** Ghana – **Western Reg.** ● ♂ (in alcohol); Ankasa Game Production Reserve; 5°15'N, 2°37'W; 31 Mar. 1993; JS Amakye & J Kjærandsen leg.; light trap; UMSP 000550043. ***Paratypes*.** Ghana – **Western Reg.** ● 8♂♂; same data as for holotype except 6–12 Dec. 1993; T Andersen & J Kjærandsen leg.; Malaise trap; ZMBN.

**Figure 46.**
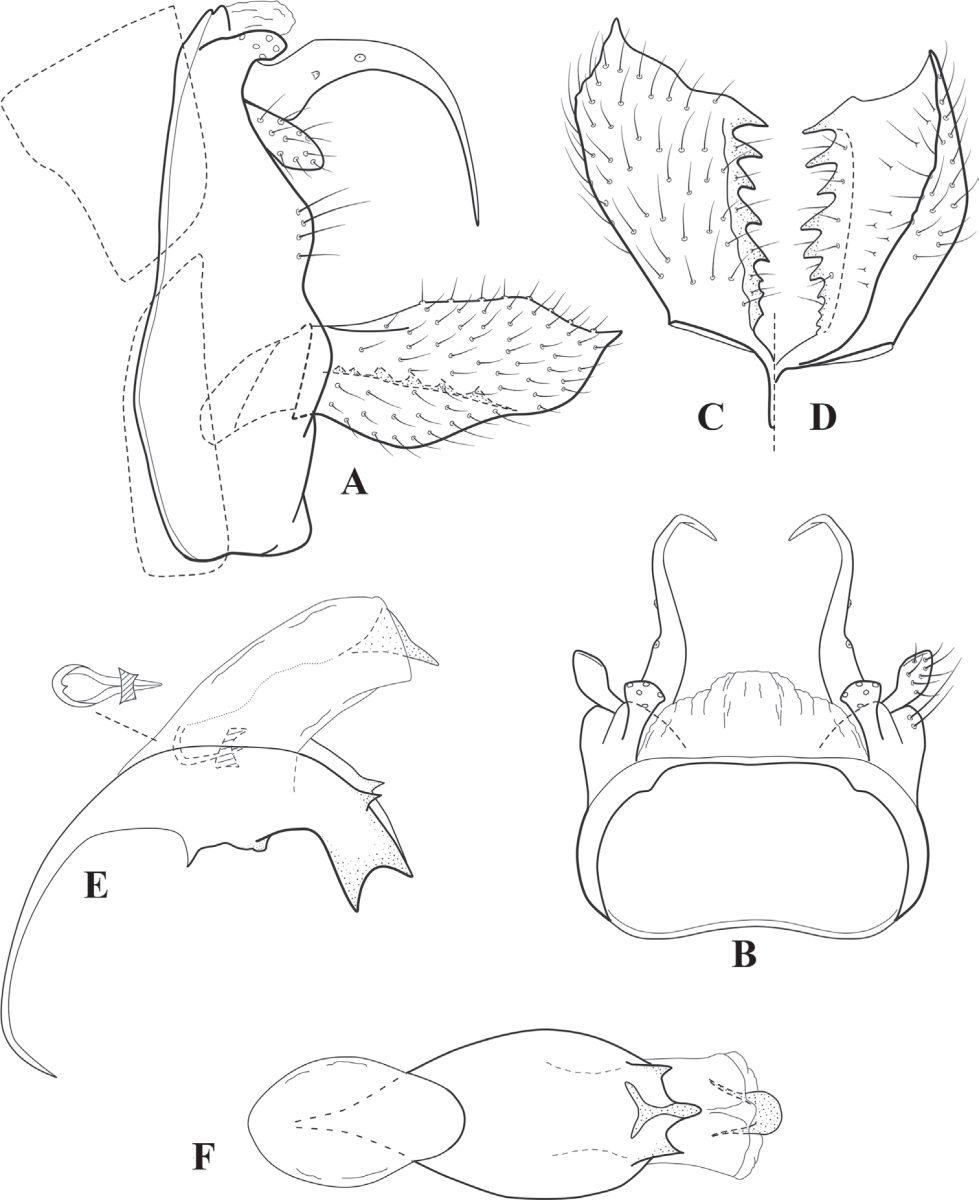
*Chimarraserrella* sp. nov., ♂ genitalia **A** lateral **B** dorsal, segments IX and X **C** inferior appendage, ventral **D** inferior appendage, dorsal **E** phallus, lateral, with dorsal detail of phallotremal sclerite complex **F** phallus, ventral.

###### Additional material.

Ghana – **Western Reg.** ● 9♀♀; Ankasa Game Production Reserve; 5°15'N, 2°37'W; 6–12 Dec. 1993; T Andersen & J Kjærandsen leg.; Malaise trap; ZMBN ● 1♀; same collection data as for preceding; UMSP.

###### Diagnosis.

*Chimarraserrella* is very similar to *C.uncinata* sp. nov. and the two undoubtedly constitute a pair of closely related sister species. The species resemble each other in the distinctive shape of the apex of the phallobase, and in the general shape of their inferior appendages, which are short, linear, and acute apically, as viewed laterally, but have the apex obliquely subtruncate, as viewed ventrally, with the ventromesal margin distinctly serrate. Neither species has cusps or projections on the mesal surface of the inferior appendages. The most distinctive difference is in the shape of tergum X, which has its apex upturned and hooked in *C.uncinata*, and very narrow and strongly downturned in *C.serrella*. The apex of the inferior appendage, in lateral view, is also more acute in *C.serrella* than in *C.uncinata*.

###### Description.

***Adult.*** Overall color (in alcohol) light brown, underside and appendages yellowish brown, setal warts of head not distinctly contrasting. Head short (postocular parietal sclerite short). Palps elongate; maxillary palp with 1^st^ segment very short (approximately as long as wide), 2^nd^ segment moderate (~ 3× 1^st^), apex with cluster of ~ 6–8 stiff setae, 3^rd^ segment elongate, 4^th^ segment short (shorter than 2^nd^), 5^th^ segment elongate and narrow (subequal to 3^rd^ and 4^th^ combined). Forewing length: male, 3.5–4.0 mm; female, 3.7–4.4 mm. Fore- and hind wings with forks I, II, III, and V present. Forewing with R_1_ straight, stem of Rs straight, or nearly so, basal fork of discoidal cell slightly enlarged, evenly forked, length of cell ~ 2× width, fork I subsessile, fork II sessile, *r* crossvein diagonal, intersecting discoidal cell before apical fork, *s*, *r-m*, and *m* crossveins linear and hyaline, both 2A and 3A looped to 1A (2A without apical fork). Hind wing with R_1_ obsolete (or fused to subcosta), forks I and II subsessile, fork III distal and relatively narrow, anal loop small. Forelegs with apical tibial spur very short; male with modified tarsal claws, apical three segments of tarsi short and flattened, claws asymmetrical, outer one elongate and twisted.

***Male genitalia.*** Segment VIII short, tergum longer than sternum, sternum without posteroventral process. Segment IX, in lateral view, very short, anteroventral margin only slightly projecting, dorsal margin without apodemes, but with pair of short, rounded, multi-sensillate projections from posterior margin, ventral process very short, subtriangular, not or scarcely projecting, inferior appendages inserted slightly above ventral margin of segment; as viewed dorsally, with tergum very narrow, but continuous, sternum short, subtruncate. Tergum X with mesal lobe very short and membranous, lateral lobes short and distinctly sclerotized, produced apically into tapering, ventrally recurved, spine-like projections; sensilla of lobes very small, reduced in number, possibly only two, on basal part of tergum. Preanal appendages short, oblong, somewhat ventrally projecting, inserted membranously (not fused to segments IX or X). Inferior appendage with very weak basal inflection; as viewed laterally, more or less ovate, acutely angulate apically, with short lateral setae, setae slightly longer and spaced on dorsal margin; as viewed ventrally, with apex obliquely truncate, with longitudinal ridge near mesal margin, mesal margin distinctly serrate. Phallic apparatus with phallobase very short and strongly sclerotized, with usual basodorsal expansion, securely anchored within segment by semi-sclerotized periphallic membrane (attached to lateral margin of segment IX), apicoventral margin of phallobase very distinctly sclerotized and produced, down-turned, apex produced into short apical spine-like processes, ventral one weakly divided, dorsolateral margin of apex with additional short spine-like projection on each side; endotheca short, membranous, with small, lightly sclerotized apical spine, apex bluntly rounded, as viewed ventrally; phallotremal sclerite complex composed of short rod and ring structure, with indistinct apicolateral sclerite.

###### Etymology.

*Chimarraserrella*, used as a noun in apposition, from the Latin diminutive for *serra*, a saw, in reference to the very serrate ventromesal margin of the inferior appendages in this species.

##### ﻿ Chimarra triramosa sp. nov.

Taxon classificationAnimaliaTrichopteraPhilopotamidae

5487BDF9-B52D-576A-BD57-971EFB27FC3A

http://zoobank.org/E9134C4F-47C0-40FF-8CF9-66987226CAF1

Fig. 47A–E

###### Type material.

***Holotype*.** Ghana – **Volta Reg.** ● ♂ (in alcohol); Wli, Agumatsa waterfall, station # 12^A^; 7°07'29"N, 0°35'31"E; 13–16 Mar.1993; JS Amakye & J Kjærandsen leg.; Malaise trap; UMSP 000550045. ***Paratypes*.** Ghana – **Volta Reg.** ● 3♂♂; same data as for holotype except station # 5^A^; 4–13 Mar. 1993; ZMBN ● 1♂; same data as for holotype except station # 5^B^; 10–13 Mar. 1993; ZMBN ● 2♂♂; same data as for holotype except station # 6^B^; 12–15 Mar. 1993; ZMBN ● 1♂; same data as for holotype except station # 7^A^; 10–13 Mar. 1993; ZMBN ● 3♂♂; same data as for holotype except station # 8^A^; 7–13 Mar. 1993; ZMBN ● 3♂♂; same data as for holotype except station # 8^B^; ZMBN ● 3♂♂; same data as for holotype except station # 9^A^; 10–13 Mar. 1993; ZMBN ● 13♂♂; same data as for holotype except station # 9^B^; 4–13 Mar. 1993; ZMBN ● 281♂♂; same data as for holotype except station # 10^A^; ZMBN ● 10♂♂; same data as for holotype except station # 11^A^; ZMBN ● 5♂♂; same data as for holotype except station # 12^A^; 7–13 Mar. 1993; ZMBN ● 2♂♂; same data as for holotype except station # 12^B^; 7–16 Mar. 1993; ZMBN ● 7♂♂; same data as for holotype except station # 6; 11 Mar. 1993; light trap; ZMBN ● 286♂♂; same data as for holotype except station # 10; 8 Mar. 1993; light trap; ZMBN ● 3♂♂; same data as for holotype except 19 Nov. 1993; J Kjærandsen leg.; light trap; ZMBN ● 45♂♂; same data as for holotype except 8 Mar. 1993; sweep net; ZMBN. – **Eastern Reg.** ● 2♂♂; Boti Falls; 6°11'40"N, 0°13'05"W; 28 Oct. – 4 Nov. 1994; T Andersen leg.; Malaise trap; ZMBN ● 4♂♂; Kibi, Subri stream; 6°10'N 0°33'W; 5 Nov. 1993; J Kjærandsen leg.; light trap; ZMBN.

**Figure 47.**
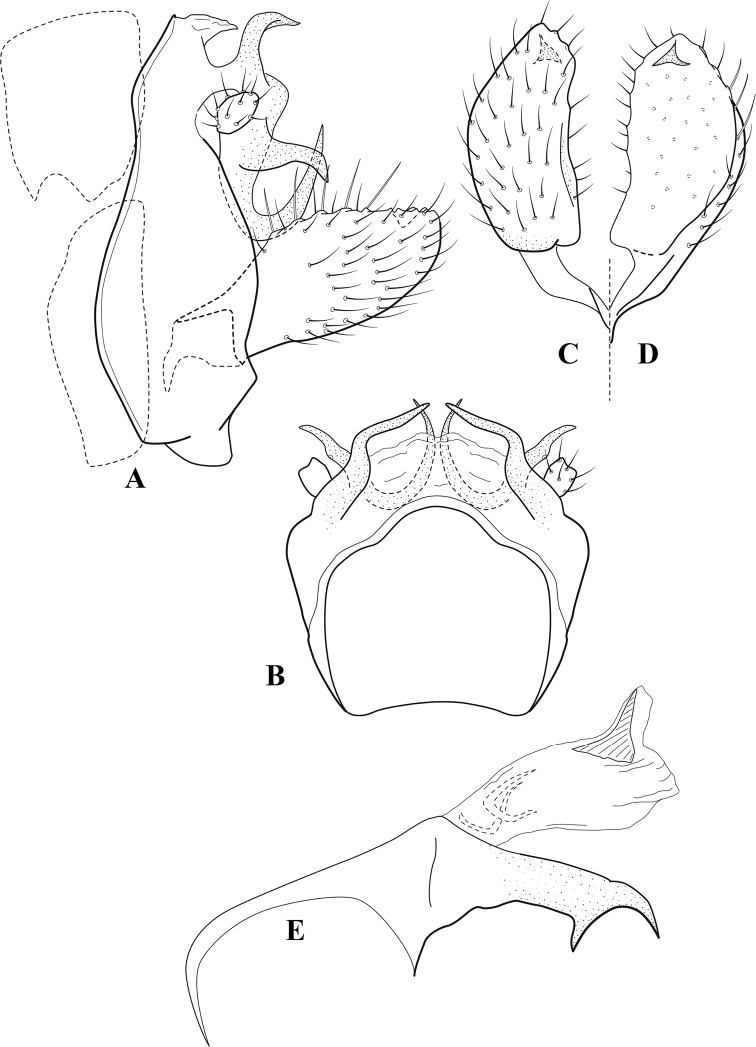
*Chimarratriramosa* sp. nov., ♂ genitalia **A** lateral **B** dorsal, segments IX and X **C** inferior appendage, ventral **D** inferior appendage, dorsal **E** phallus, lateral.

###### Additional material.

Ghana – **Eastern Reg.** ● 4♀♀; Kibi, Subri stream; 6°10'N, 0°33'W; 5 Nov. 1993; J Kjærandsen leg.; light trap; ZMBN. – **Volta Reg.** ● 2♀♀; Wli, Agumatsa waterfall, station # 5^B^; 7°07'29"N, 0°35'31"E; 10–13 Mar. 1993; JS Amakye & J Kjærandsen leg.; Malaise trap; ZMBN ● 3♀♀; same collection data as for preceding except station # 6^B^; 12–15 Mar. 1993; ZMBN ● 2♀♀; same collection data as for preceding except station # 8^B^; 7–13 Mar. 1993; ZMBN ● 2♀♀; same collection data as for preceding except station # 9^A^; 10–13 Mar. 1993; ZMBN ● 6♀♀; same collection data as for preceding except station # 9^B^; 4–13 Mar. 1993; ZMBN ● 230♀♀; same collection data as for preceding except station # 10^A^; ZMBN ● 2♀♀; same collection data as for preceding except station # 10^B^; 10–13 Mar. 1993; ZMBN ● 6♀♀; same collection data as for preceding except station # 11^A^; 4–13 Mar. 1993; ZMBN ● 2♀♀; same collection data as for preceding except station # 12^A^; 7–13 Mar. 1993; ZMBN ● 2♀♀; same collection data as for preceding except station # 12^B^; 7–16 Mar. 1993; ZMBN ● 70♀♀; same collection data as for preceding except station # 10; 8 Mar. 1993; light trap; ZMBN ● 1♀; same collection data as for preceding except station # 12; 16 Nov. 1993; J Kjærandsen leg.; ZMBN ● 1♀; same collection data as for preceding except 19 Nov. 1993; UMSP.

###### Diagnosis.

*Chimarratriramosa* is most readily diagnosed by its ovate inferior appendages, each with a short preapical projection on its mesal surface; lateral lobes of tergum X, each of which is divided into three pairs of short, curved spine-like projections; and especially by the short phallobase, with a decurved and mesally divided ventral apex, each half of which, in turn, has its apex crescentic, forming an additional two apical projections, in lateral view.

###### Description.

***Adult.*** Overall color (in alcohol) light brown, underside and appendages yellowish brown, setal warts of head pale, contrasting. Head short (postocular parietal sclerite short). Palps elongate; maxillary palp with 1^st^ segment very short (approximately as long as wide), 2^nd^ segment moderately elongate (slightly shorter than 3^rd^), apex with cluster of ~ 6–8 stiff setae, 3^rd^ segment elongate, 4^th^ segment short (shorter than 2^nd^), 5^th^ segment very elongate and narrow (slightly shorter than 3^rd^ and 4^th^ combined); both sets of palps with evident longitudinal row of more elongate setae on dorsomesal surface, graded and shortened on apical segments. Forewing length: male, 3.2–4.0 mm; female, 4.0–5.0 mm. Fore- and hind wings with forks I, II, III, and V present. Forewing with R_1_ straight, stem of Rs straight, or nearly so, basal fork of discoidal cell slightly enlarged, evenly forked, length of cell ~ 2× width, forks I and II subsessile, *r* crossvein diagonal, intersecting discoidal cell near apical fork, *s*, *r-m*, and *m* crossveins linear and hyaline, both 2A and 3A looped to 1A (2A without apical fork). Hind wing with R_1_ obsolete (or fused to subcosta), forks I and II subsessile, fork III distal and relatively narrow, anal loop small. Forelegs with apical tibial spur very short; male with modified tarsal claws, apical three segments of tarsi short and flattened, claws asymmetrical, outer one elongate and twisted.

***Male genitalia.*** Segment VIII very short, tergum slightly longer than sternum, sternum without posteroventral projection. Segment IX, in lateral view, very short, anteroventral margin only slightly expanded, dorsal margin without apodemes, ventral process very short, subtriangular, more or less ventrally oriented, inferior appendages inserted near ventral margin; as viewed dorsally, with tergum very narrow, but continuous, sternum short, subtruncate. Tergum X with mesal lobe very short and membranous, lateral lobes very short and strongly sclerotized, somewhat variable in structure, each produced into three more or less spine-like lobes, one curved ventrally, with apex recurved dorsally, one intermediate and laterally curved lobe, often subtruncate apically, and one dorsomesally curved lobe; sensilla of lobes very small and reduced in number (possibly only two, one on posterobasal margin of dorsal lobe, other either apically or preapically on lateral lobe). Preanal appendages short and rounded, slightly flattened, inserted membranously (not fused to segments IX or X). Inferior appendage without basal inflection; as viewed laterally, ovately rounded, subtruncate apically, with short lateral setae and row of spaced, more elongate setae on dorsal margin; mesal surface with distinctly sclerotized, short, preapical spine-like projection. Phallic apparatus with phallobase very short and strongly sclerotized, with usual basodorsal expansion, securely anchored within segment by semi-sclerotized periphallic membrane (attached to lateral margin of segment IX), apicoventral margin of phallobase very distinctly sclerotized and produced, down-turned, apex divided mesally, with each half of divided apex produced into pair of apical and preapical spine-like processes (thus with four apical spine-like projections); endotheca short, membranous, with pair of apical, lightly sclerotized, subtriangular spines (possibly connected mesally); phallotremal sclerite complex composed of short rod and ring structure, with indistinct lateral sclerite.

###### Etymology.

*Chimarratriramosa*, used as an adjective, from the Latin *ramus*, or branch, for the 3-branched tergum X of this species, which is one of its most defining characters.

##### ﻿ Chimarra uncinata sp. nov.

Taxon classificationAnimaliaTrichopteraPhilopotamidae

F078B73C-8C48-5513-8BCC-FE345FD551FE

http://zoobank.org/F8D2E59B-0617-4308-9DD0-32517239ACB4

Fig. 48A–F

###### Type material.

***Holotype*.** Ghana – **Volta Reg.** ● ♂ (in alcohol); Wli, Agumatsa waterfall, station # 12^A^; 7°07'29"N, 0°35'31"E; 7–16 Mar. 1993; JS Amakye & J Kjærandsen leg.; Malaise trap; UMSP 000550047. ***Paratypes*.** Ghana – **Volta Reg.** ● 1♂; same data as for holotype; ZMBN ● 1♂; same data as for holotype except station # 10^B^; 10–13 Mar. 1993; ZMBN ● 1♂; same data as for holotype except station # 12^B^; 13–16 Mar. 1993; ZMBN.

**Figure 48.**
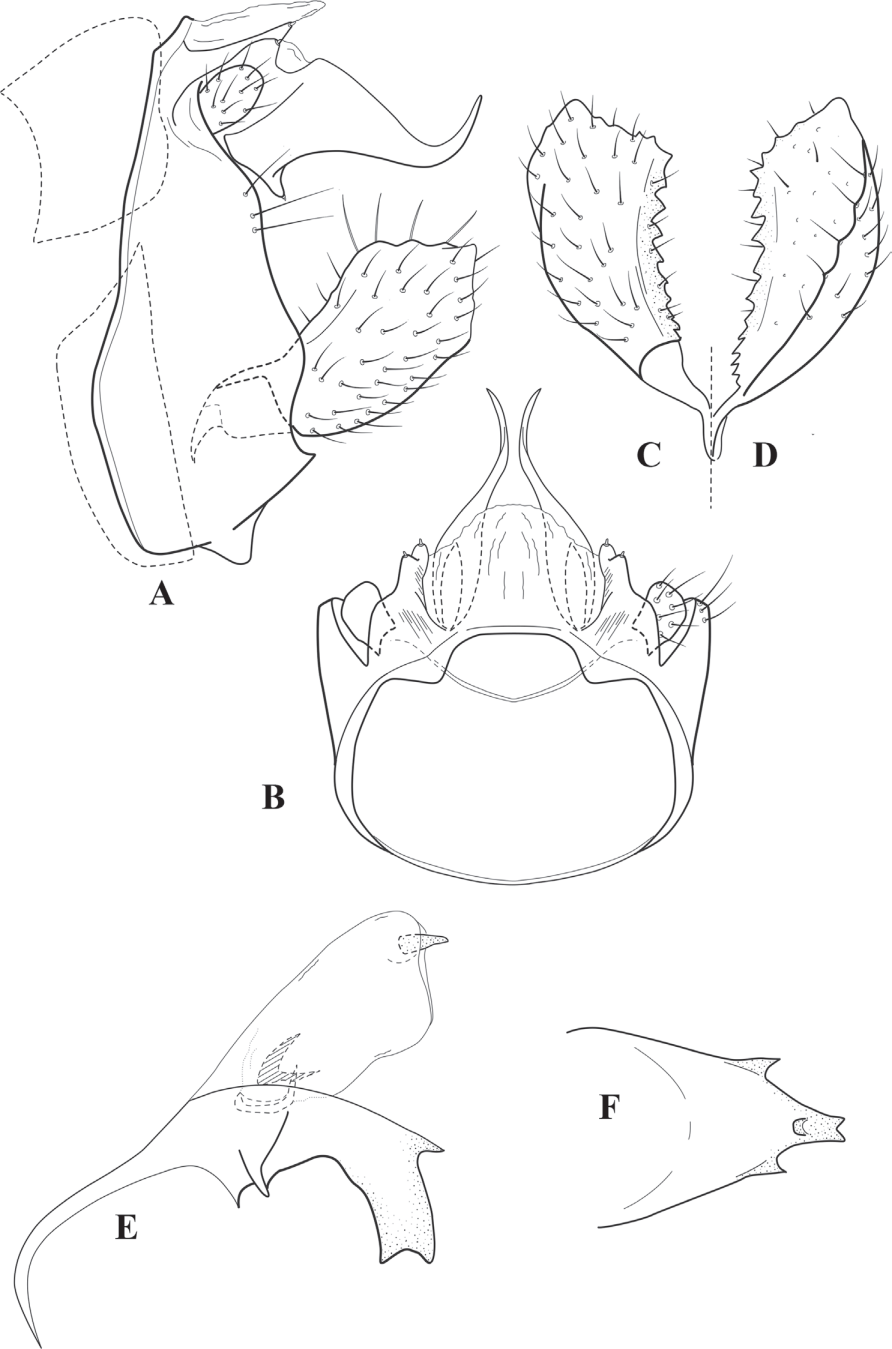
*Chimarrauncinata* sp. nov., ♂ genitalia **A** lateral **B** dorsal, segments IX and X **C** inferior appendage, ventral **D** inferior appendage, dorsal **E** phallus, lateral **F** phallus apex, ventral.

###### Additional material.

Ghana – **Volta Reg.** ● 1♀; Volta Region, Wli, Agumatsa waterfall, station # 12^A^; 7°07'29"N, 0°35'31"E; 7–10 Mar. 1993, JS Amakye & J Kjærandsen leg.; Malaise trap; UMSP.

###### Diagnosis.

*Chimarrauncinata* is most readily diagnosed, in combination, by its short inferior appendages, with the ventromesal margins very irregular and serrate, lateral lobes of tergum X, each of which has its apex hook-like, upturned and acute and also has short sensillate lobes on the basodorsal and basoventral margins, and by the form of the phallobase, which is short and has its apex sharply decurrent, with the apex bifid in both lateral and caudal views and with an additional short spine on each side of the dorsal margin just prior to the decurrent apex. It is most similar to *C.serrella* sp. nov., which also has the ventromesal margin of the inferior appendages serrate, but differs in the structure of tergum X.

###### Description.

***Adult.*** Overall color (in alcohol) light brown, underside and appendages yellowish brown, vertex of head darker, setal warts of head pale, contrasting. Head short (postocular parietal sclerite short). Palps elongate; maxillary palp with 1^st^ segment very short (approximately as long as wide), 2^nd^ segment moderately elongate (slightly shorter than 3^rd^), apex with cluster of ~ 6–8 stiff setae, 3^rd^ segment elongate, 4^th^ segment short (shorter than 2^nd^), 5^th^ segment very elongate and narrow (slightly shorter than 3^rd^ and 4^th^ combined). Forewing length: male, 3.2–3.8 mm. Fore- and hind wings with forks I, II, III, and V present. Forewing with R_1_ straight, stem of Rs straight, or nearly so, basal fork of discoidal cell slightly enlarged, evenly forked, length of cell ~ 2× width, fork I subsessile, fork II sessile, *r* crossvein diagonal, intersecting discoidal cell near apical fork, *s*, *r-m*, and *m* crossveins linear and hyaline, both 2A and 3A looped to 1A (2A without apical fork). Hind wing with R_1_ obsolete (or fused to subcosta), forks I and II subsessile, fork III distal and relatively narrow, anal loop small. Forelegs with apical tibial spur very short; male with modified tarsal claws, apical three segments of tarsi short and flattened, claws asymmetrical, outer one elongate and twisted.

***Male genitalia.*** Segment VIII very short, tergum slightly longer than sternum, sternum without posteroventral projection. Segment IX, in lateral view, very short, anteroventral margin only slightly expanded, dorsal margin without apodemes, ventral process very short, subtriangular, more or less ventrally oriented, inferior appendages inserted near ventral margin; as viewed dorsally, with tergum very narrow, but continuous, sternum short, subtruncate. Tergum X with mesal lobe very short and membranous, lateral lobes short and distinctly sclerotized, produced apically into dorsally recurved spine-like projections; sensilla of lobes very small, reduced in number, on short nipple-like basal projections, one dorsally with two or three sensilla and one ventrally with single apical sensillum. Preanal appendages short and rounded, distinctly flattened, inserted membranously (not fused to segments IX or X). Inferior appendage with very weak basal inflection; as viewed laterally, more or less ovate, subangulate apically, with short lateral setae and row of spaced, more elongate setae on dorsal margin; as viewed ventrally, with longitudinal ridge near mesal margin, mesal margin distinctly serrate. Phallic apparatus with phallobase very short and strongly sclerotized, with usual basodorsal expansion, securely anchored within segment by semi-sclerotized periphallic membrane (attached to lateral margin of segment IX), apicoventral margin of phallobase very distinctly sclerotized and produced, down-turned, apex produced into short apical and preapical spine-like processes, apical one weakly divided, dorsolateral margin of apex with additional short spine-like projection on each side; endotheca short, membranous, with very small, lightly sclerotized apical spine; phallotremal sclerite complex composed of short rod and ring structure, with indistinct lateral sclerite.

###### Etymology.

*Chimarrauncinata*, used as an adjective, from the Latin *uncus*, or hook, for the hooked apex of tergum X in this species, which is a useful identifying character.

##### ﻿ Chimarra vermitergata sp. nov.

Taxon classificationAnimaliaTrichopteraPhilopotamidae

C90A6EA4-FAB7-54A5-A7E4-C7F492DB5D2F

http://zoobank.org/3AFD8372-63B3-433C-9B04-D72CDD682FDE

Fig. 49A–E

###### Type material.

***Holotype*.** Tanzania – **Tanga Reg.** ● ♂ (in alcohol); West Usambara Mts, Gologolo; 4°41'S, 38°13'E; 25 Nov. 1990; T Andersen leg.; sweep net; UMSP 000550049. ***Paratypes*.** Tanzania – **Tanga Reg.** ● 1♂; same data as for holotype; ZMBN ● 1♂; West Usambara Mts, Mazumbai, Kaputu Stream; 4°48'S, 38°30'E; 4–13 Jan. 1991; T Andersen leg.; Malaise trap; ZMBN ● 2♂♂; same collection data as for preceding except 5 Nov. 1990; sweep net; ZMBN.

**Figure 49.**
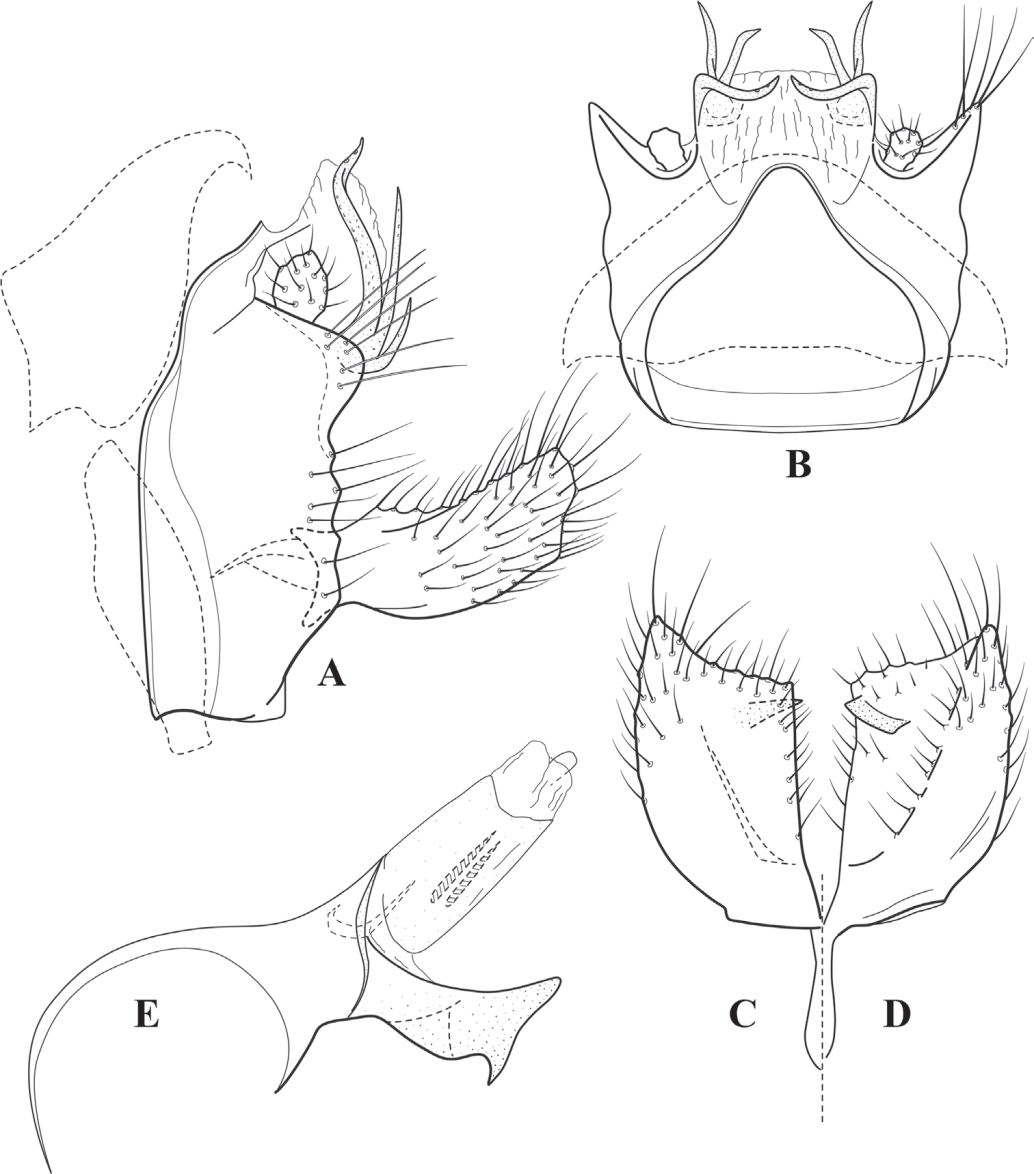
*Chimarravermitergata* sp. nov., ♂ genitalia **A** lateral **B** dorsal, segments IX and X **C** inferior appendage, ventral **D** inferior appendage, dorsal **E** phallus, lateral.

###### Diagnosis.

*Chimarravermitergata* has an overall similarity to both *C.leptodactylus* sp. nov. and *C.latidentis* sp. nov. It is most similar to *C.latidentis*; only a direct comparison of the genitalia offers convincing evidence that they are different species. The most evident difference, as apparent from the accompanying illustrations, is in the more divided and less scabrous lateral lobe of tergum X in *C.vermitergata*. However, this is a relatively minor difference, and it is difficult to know how constant this character may be from the limited material available. A synopsis of the differences between *C.vermitergata* and *C.latidentis* include: a somewhat more elongate inferior appendage, with a shorter, less prominent cusp on the mesal surface; structural details of the lateral lobes of tergum X, which have the spine-like basal projections narrow and divided in *C.vermitergata* and with the apices less evidently scabrous than in *C.latidentis*; and a phallic apparatus with a narrow, tube-like, and lightly sclerotized basal portion of the endotheca apical to the deflexed and paired ventral projections of the phallobase, rather than one that is short and bulbous. In combination, these differences provide sufficient evidence that the two should be considered different species.

###### Description.

***Adult.*** Overall color (in alcohol) medium brown to yellowish brown, head slightly darker, setal warts of head not, or hardly, contrasting. Head short (postocular parietal sclerite relatively short, shorter than eye). Palps elongate; maxillary palp with 1^st^ segment very short (approximately as long as wide), 2^nd^ segment short (~ 2× 1^st^), apex with cluster of ~ 8 stiff setae, 3^rd^ segment very elongate (> 2× 2^nd^), 4^th^ segment short (shorter than 2^nd^), 5^th^ segment elongate and narrow (slightly longer than 3^rd^). Forewing length: male, 7.0 mm. Fore- and hind wings with forks I, II, III, and V present. Forewing with R_1_ straight, stem of Rs straight, or nearly so, basal fork of discoidal cell slightly enlarged, evenly forked, discoidal cell slightly longer than 2× width, forks I and II sessile, *r* crossvein diagonal, intersecting discoidal cell just before fork I, *s*, *r-m*, and *m* crossveins more or less linear and hyaline (*m* crossvein somewhat diagonal), both 2A and 3A looped to 1A (2A without apical fork). Hind wing with R_1_ evident basally, obsolete (or fused to subcosta) apically, fork I sessile, fork II slightly subsessile, fork III distal and relatively wide, anal loop small. Forelegs with apical tibial spur short; male with tarsal claws not enlarged, claws symmetrical, tarsal segments narrow.

***Male genitalia.*** Segment VIII with sternum very short, tergum ~ 2× as long, dorsal margin projecting, sternum without posteroventral projection. Segment IX, in lateral view, short, anteroventral margin only slightly expanded, anterodorsal margin without apodemes, posterior margin angularly projecting below preanal appendages, sternum with very short, subtriangular ventral process from posterior margin, inferior appendages inserted somewhat above ventral margin; as viewed dorsally, with tergum very narrow, but continuous (or nearly so), sternum very short, subtruncate. Tergum X with mesal lobe short and membranous, lateral lobes short and sclerotized, each modified into several narrow, upturned spine-like projections, dorsal ones longest, mesally curved and with two sensilla near apex. Preanal appendages short and rounded, slightly flattened, inserted membranously (not fused to segments IX or X). Inferior appendage with weak basal inflection; as viewed laterally, short, with apicodorsal margin somewhat angulate and laterally projecting; as viewed ventrally, subtruncate apically, with mesal margins of opposite appendages proximate, then sharply bent; mesal surface with sclerotized, anteriorly projecting, cusp-like projection, apparently articulating with sclerotized ventral projection of phallobase. Phallic apparatus with phallobase very short and strongly sclerotized, with usual basodorsal expansion, securely anchored within segment by sclerotized periphallic membrane (and apparently fused to it); apicoventral margin of phallobase (or projections from periphallic membrane) very distinctly sclerotized and produced, down-turned, apex divided mesally, apparently articulating with spine-like projections of mesal surface of inferior appendages; phallic apparatus distal to sclerotized ventral projection (possibly modified endotheca), forming narrow sclerotized tube, apparently as extension of phallobase; endotheca with pair of very short, symmetrically positioned spines; phallotremal sclerite complex composed of short rod and ring structure.

###### Etymology.

*Chimarravermitergata*, used as an adjective, from the Latin *vermis*, a worm, and *tergum*, a back, for the narrow, irregular, and worm-like divisions of tergum X of this species.

#### The *evoluta* subgroup

**Included species.***Chimarraaciculata* Morse, 1974; *C.evoluta* Kimmins, 1957; *C.foliata* Kimmins, 1959; *C.giboni* sp. nov.; *C.lobulata* sp. nov.; *C.mgwashi* sp. nov.; *C.parafoliata* sp. nov.; and *C.pectinella* sp. nov.

This subgroup is distinguished from the *georgensis* subgroup by its more reduced venation (absence of forks I and III in the hind wing, in addition to the fused R_1_ and subcosta veins), and by the presence of elongate and often scabrous dorsal processes from the dorsal margin of segment IX. Since tergum X is fused with and continuous with the posterior margin of segment IX, it is conceivable that these processes actually have their origin as a basally divided lobe from the lateral lobes of tergum X. The apparent lateral lobes of tergum X are simpler in overall structure than in species of the *georgensis* subgroup, lacking the scabrous or acute and divided lobes present in this subgroup, and generally with two rather evident sensilla on each lobe, one apical and one preapical.

##### ﻿ Chimarra giboni sp. nov.

Taxon classificationAnimaliaTrichopteraPhilopotamidae

FE8810C0-98A3-5573-BEEC-9DF53A37B265

http://zoobank.org/2DD1B8CE-7038-4454-8F5F-07A2662A70CD

Fig. 50A–D

###### Type material.

***Holotype*.** Ghana – **Western Reg.** ● ♂ (in alcohol); Ankasa Game Production Reserve; 5°15'N, 2°37'W; 6–12 Dec. 1993; T Andersen & J Kjærandsen leg.; Malaise trap; UMSP 000550050. ***Paratypes*.** Ghana – **Western Reg.** ● 4♂♂; same data as for holotype; ZMBN. – **Central Reg.** ● 3♂♂; Kakum Forest Reserve; 5°21'N, 1°22'W; 8–15 Nov. 1994; T Andersen leg.; Malaise trap; ZMBN.

**Figure 50.**
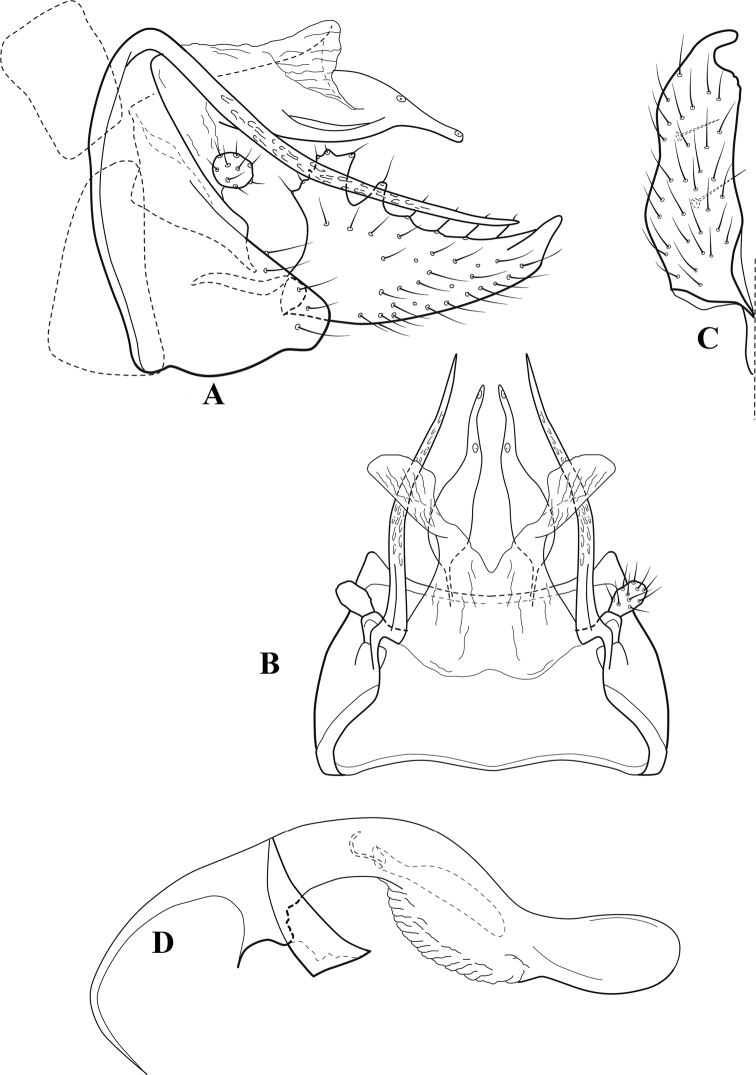
*Chimarragiboni* sp. nov., ♂ genitalia **A** lateral **B** dorsal, segments IX and X **C** inferior appendage, ventral **D** phallus, lateral.

###### Additional material.

Ghana – **Central Reg.** ● 9♀♀; Kakum Forest Reserve; 5°21'N, 1°22'W; 8–15 Nov. 1994; T Andersen leg.; Malaise trap; ZMBN ● 1♀; same collection data as for preceding; UMSP. – **Western Reg.** ● 69♀♀; Ankasa Game Production Reserve; 5°15'N, 2°37'W; 6–12 Dec. 1993; T Andersen & J Kjærandsen leg.; Malaise trap; ZMBN ● 1♀; same collection data as for preceding; UMSP.

###### Diagnosis.

This species is probably most closely related to *Chimarrafoliata* Kimmins and *C.parafoliata* sp. nov., resembling both in having foliate basal projections on the lateral lobes of tergum X and in having the posterior margin of segment IX strongly produced at the level of the inferior appendage. It differs in the much more elongate and apically projecting inferior appendages, without a mesal tooth or cusp, and in that the posterior projection of segment IX is at, or nearly at, the ventral margin of the segment.

###### Description.

***Adult.*** Overall color (in alcohol) yellowish brown, vertex of head slightly darker. Head short (postocular parietal sclerite short). Palps elongate; maxillary palp with 1^st^ segment very short (approximately as long as wide), 2^nd^ segment moderately elongate (~ 3× 1^st^), apex with cluster of ~ 8–10 stiff setae, 3^rd^ segment moderately elongate, slightly longer than 2^nd^, 4^th^ segment short (~ ½ length of 2^nd^), 5^th^ segment very elongate and narrow (subequal to 3^rd^ and 4^th^ combined). Forewing length: male, 3.2–4.0 mm; female, 3.5–4.5 mm. Forewing forks I, II, III, and V present; hind wing with forks II and V only. Forewing with R_1_ nearly straight, stem of Rs very weakly inflected, basal fork of discoidal cell slightly enlarged, evenly forked, length of cell ~ 2× width, forks I and II sessile, *r* crossvein not evident, *s*, *r-m*, and *m* crossveins linear and hyaline, both 2A and 3A looped to 1A (2A without apical fork). Hind wing with R_1_ obsolete (or fused to subcosta), fork II sessile, anal loop small. Forelegs with apical tibial spur short; male with modified tarsal claws, apical three segments of tarsi short and flattened, claws asymmetrical, outer one elongate and twisted.

***Male genitalia.*** Segment VIII short, sternum without posteroventral projection. Segment IX, in lateral view, with anterior margin nearly straight, ventral margin very distinctly expanded, forming projection point for attachment of inferior appendages, segment narrowing and convergent dorsally, dorsal margin without apodemes, but with paired, elongate, narrow, scabrous, posteroventrally directed processes from posterolateral margin, apices of processes acute, ventral process of segment apparently obsolete; as viewed dorsally, with tergum discontinuous mesally, posterior processes widely separated basally. Tergum X with membranous mesal lobe, divided mesally, lateral lobes with expanded foliate basal lobes and narrow, projecting apices; sensilla two on each lobe, one preapical and one apical, on narrow projection. Preanal appendages small and knob-like, rounded, constricted basally, inserted membranously (not fused to segments IX or X). Inferior appendage, as viewed laterally, relatively elongate, subtriangular, with basodorsal expansion, gradually narrowing to acute apex, without cusp or tooth on mesal margin; as viewed ventrally, with apex narrowed and mesally curved. Phallic apparatus with phallobase short, well anchored within segment by semi-sclerotized periphallic membrane (attached to lateral margin of segment IX); apex of phallobase and endotheca not well demarcated, endotheca apparently weakly sclerotized, forming looped structure with rounded apex; phallotremal sclerite complex small and indistinct, composed of short rod and ring structure and weakly sclerotized apical structure.

###### Etymology.

We take pleasure in naming this species *Chimarragiboni* for François-Marie Gibon, in recognition of his many contributions to the taxonomy of Trichoptera in Africa and Madagascar, and especially the genus *Chimarra*.

##### ﻿ Chimarra lobulata sp. nov.

Taxon classificationAnimaliaTrichopteraPhilopotamidae

397D0AD7-5771-5E59-9957-C3FE830740CB

http://zoobank.org/6E004EBF-33C6-4C0C-A2AA-5D8F25118D37

Fig. 51A–F

###### Type material.

***Holotype*.** Ghana – **Western Reg.** ● ♂ (in alcohol); Ankasa Game Production Reserve; 5°15'N, 2°37'W; 6–12 Dec. 1993; T Andersen & J Kjærandsen leg.; Malaise trap; UMSP 000550052.

**Figure 51.**
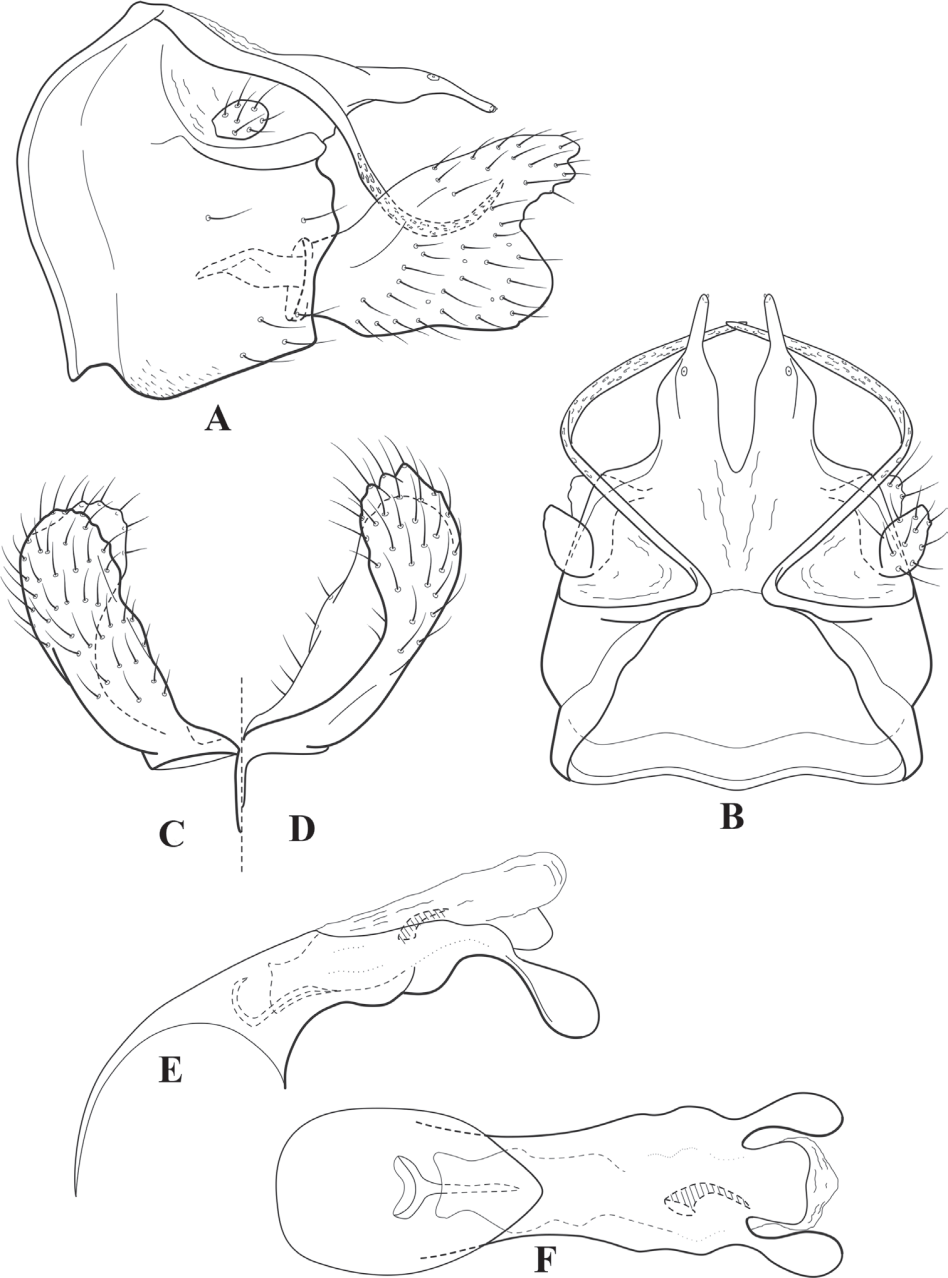
*Chimarralobulata* sp. nov., ♂ genitalia **A** lateral **B** dorsal, segments IX and X **C** inferior appendage, ventral **D** inferior appendage, dorsal **E** phallus, lateral **F** phallus, ventral.

###### Additional material.

Ghana – **Western Reg.** ● 2♀♀; Ankasa Game Production Reserve; 5°15'N, 2°37'W; 6–12 Dec. 1993; T Andersen & J Kjærandsen leg.; Malaise trap; ZMBN ● 1♀; same collection data as for preceding; UMSP.

###### Diagnosis.

*Chimarralobulata* is closely related to *C.pectinella* sp. nov., as evidenced by the shape of segment IX, including the mesally proximate posterior processes, shape of tergum X, and the paired apicoventral lobes of the phallobase. It differs diagnostically in the much more prominent apicolateral lobes of the phallobase, as well as in the shape of the interior appendages, which have the posterodorsal margin projecting and lack cusps on the mesal margin.

###### Description.

***Adult.*** Overall color (in alcohol) light brown to yellowish brown, head slightly darker, setal warts of head pale, contrasting. Head relatively short (postocular parietal sclerite short), slightly flattened. Palps elongate; maxillary palp with 1^st^ segment very short (approximately as long as wide), 2^nd^ segment moderately elongate (~ 3× 1^st^, slightly shorter than 3^rd^), apex with cluster of ~ 8 stiff setae, 3^rd^ segment moderately elongate, 4^th^ segment short (~ 1/2 length of 2^nd^), 5^th^ segment very elongate and narrow (slightly longer than 3^rd^ and 4^th^ combined). Forewing length: male, 4.0 mm; female, 4.0–4.3 mm. Forewing forks I, II, III, and V present; hind wing with forks II and V only. Forewing with R_1_ nearly straight, stem of Rs weakly, but distinctly inflected, basal fork of discoidal cell enlarged, asymmetrically forked, length of cell slightly > 2× width, fork I subsessile, fork II sessile, *r* crossvein diagonal, intersecting discoidal cell near apical fork, *s*, *r-m*, and *m* crossveins linear and hyaline, both 2A and 3A looped to 1A (2A without apical fork). Hind wing with R_1_ obsolete (or fused to subcosta), fork II sessile, anal loop small. Forelegs with apical tibial spur short; male with modified tarsal claws, apical three segments of tarsi short and flattened, claws asymmetrical, outer one elongate and twisted.

***Male genitalia.*** Segment VIII moderate in length, tergum slightly longer than sternum, sternum without posteroventral projection. Segment IX, in lateral view, relatively long, narrowed dorsally at about level of preanal appendages, ventral margin not, or hardly, expanded, dorsal margin without apodemes, but with paired, elongate, narrow, scabrous, posteroventrally-directed processes from posterior margin, apices of processes acute, ventral process absent; as viewed dorsally, with tergum discontinuous mesally, posterior processes proximate mesally, bowed outward, sternum subtruncate. Tergum X without evident mesal lobe, lateral lobes divided mesally, moderately elongate and narrow, with narrow, projecting apex; sensilla probably only two on each lobe, one apical and the other preapical. Preanal appendages very small and rounded, inserted membranously (not fused to segments IX or X). Inferior appendage, as viewed laterally relatively short and wide, without distinct basal inflection, apicodorsal margin distinctly projecting; as viewed caudally, with slight mesal curvature, apex rounded, mesal surface without cusp, but ventromesal margin distinctly sclerotized. Phallic apparatus with phallobase relatively short, lightly sclerotized, with usual basodorsal expansion, apparently well anchored within segment by semi-sclerotized periphallic membrane (attached to lateral margin of segment IX), apicoventral margin of phallobase sclerotized, with lobate, ventrally projecting lobes, mesal margin between lobes slightly projecting and truncate; endotheca with short, curved spine; phallotremal sclerite complex large, composed of relatively elongate rod and ring structure, with lightly sclerotized apical structure.

###### Etymology.

*Chimarralobulata*, used as an adjective, from the Latin *lobus*, a rounded projection or protuberance, and referring to the lobulate apex of the phallobase in this species.

##### ﻿ Chimarra mgwashi sp. nov.

Taxon classificationAnimaliaTrichopteraPhilopotamidae

89B8FF3F-ED6C-52C6-8978-08DA739919EA

http://zoobank.org/6FDC2AA0-73A5-43EA-87C3-BC6889272C62

Fig. 52A–E

###### Type material.

***Holotype*.** Tanzania – **Tanga Reg.** ● ♂ (in alcohol); West Usambara Mts, Mgwashi, Shokoi River; 4°46'S, 38°29'E; 24 Nov. 1990; T Andersen leg.; sweep net; UMSP 000550054.

**Figure 52.**
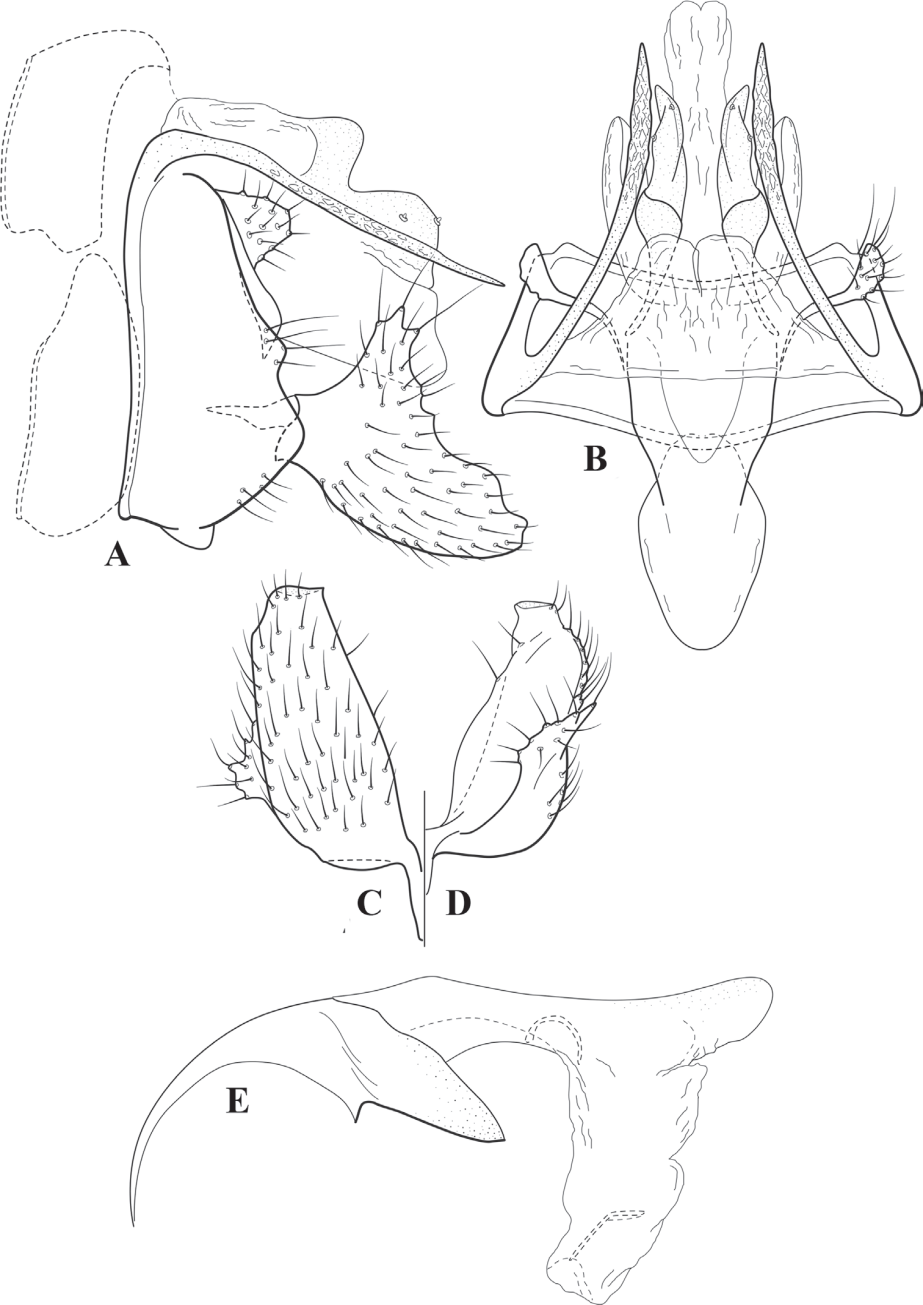
*Chimarramgwashi* sp. nov., ♂ genitalia **A** lateral **B** dorsal, segments IX and X and base of phallus **C** inferior appendage, ventral **D** inferior appendage, dorsal **E** phallus, lateral.

###### Diagnosis.

*Chimarramgwashi* sp. nov. is most similar to *C.aciculata* Morse and *C.evoluta* Kimmins, particularly in the general shape of segment IX and inferior appendages. Diagnostic differences from *C.aciculata* include the overall shape of the inferior appendage, in lateral view, which in *C.mgwashi* has its dorsal process more basal and not hooked or curved mesally, and the shape of the phallobase, which, in *C.mgwashi*, is very short and obscured by the strongly sclerotized lateral projections of the phallocrypt. The most useful diagnostic feature separating *C.mgwashi* from *C.evoluta* is the shape of the apex of the inferior appendage in ventral view, which is subtruncate in *C.mgwashi*, but narrowed and mesally hooked in *C.evoluta*.

###### Description.

***Adult.*** Overall color (in alcohol) dark brown. Head short (postocular parietal sclerite short, < 1/2 diameter of eye). Palps elongate, maxillary palp with 1^st^ segment very short (slightly longer than wide), 2^nd^ segment moderate (~ 2× 1^st^), apex with cluster of ~ 8–10 stiff setae, 3^rd^ segment elongate (~ 1½ × 2^nd^), 4^th^ segment short (~ 1/2 length of 3^rd^), 5^th^ segment very elongate and narrow (subequal to 3^rd^ and 4^th^ combined). Forewing length: male, 5.2 mm. Forewing forks I, II, III, and V present; hind wing with forks II and V only. Forewing with R_1_ nearly straight, stem of Rs very weakly inflected in middle, basal fork of discoidal cell enlarged, evenly forked, length of cell ~ 2½ × width, forks I and II sessile, *r* crossvein not evident, *s*, *r-m*, and *m* crossveins linear and hyaline, both 2A and 3A looped to 1A (2A without apical fork). Hind wing with R_1_ obsolete (or fused to subcosta), fork II slightly subsessile, anal loop small. Forelegs with apical tibial spur short; male with foretarsi modified, tarsal claws enlarged and asymmetrically developed.

***Male genitalia.*** Segment VIII short, sternum without posteroventral projection. Segment IX, in lateral view, very short, with anterior margin nearly straight, without dorsolateral apodemes, posterior margin somewhat expanded at attachment point of inferior appendages; dorsal margin with paired, elongate, narrow, scabrous, posteroventrally directed processes from anterolateral margin, apices of processes acute; ventral process of segment from ventral margin, very small, rounded, ventrally directed; as viewed dorsally, with tergum discontinuous mesally, posterior processes widely separated basally. Tergum X with relatively short membranous mesal lobe, divided mesally, lateral lobes, as viewed laterally, with dorsal margin more sclerotized, forming two rounded projections, the more distal one with two sensilla. Preanal appendages rounded, mound-like, fused basally. Inferior appendage, as viewed laterally, moderately elongate, narrowing apically, with short, tapering, basodorsal expansion; as viewed dorsally, without cusp or tooth on mesal margin, apex truncate. Phallic apparatus with phallobase very short, with usual basodorsal expansion, well anchored within segment by sclerotized periphallic membrane (attached to lateral margin of segment IX), appearing as sclerotized lateral wings, ventral apex of phallobase not projecting, continuous with endotheca; endotheca membranous, with slightly sclerotized dorsal lobe, phallic spines apparently absent; phallotremal sclerite complex composed of short, rather indistinct, rod and ring structure.

###### Etymology.

*Chimarramgwashi*, used as a noun in apposition, and named for the site in Tanzania where the holotype specimen was collected.

##### ﻿ Chimarra parafoliata sp. nov.

Taxon classificationAnimaliaTrichopteraPhilopotamidae

98FE273A-102A-5DD0-90B3-4AABCC4F2314

http://zoobank.org/4BFE5A74-ABBC-4E77-BB6D-2A2B80E54C7D

Fig. 53A–F

###### Type material.

***Holotype*.** Ghana – **Eastern Reg.** ● ♂ (in alcohol); Kibi, Subri stream; 6°10'N, 0°33'W; 4 Feb. 1993; J Kjærandsen leg.; at light; UMSP 000550067.

**Figure 53.**
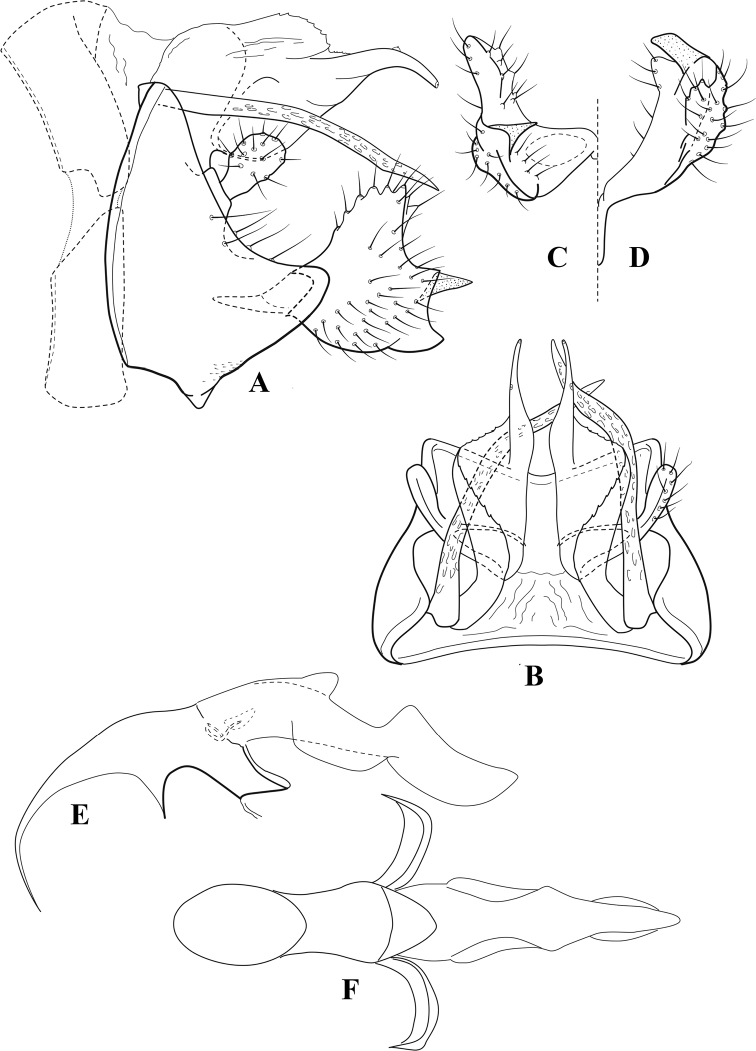
*Chimarraparafoliata* sp. nov., ♂ genitalia **A** lateral **B** dorsal, segments IX and X **C** inferior appendage, caudal **D** inferior appendage, dorsal **E** phallus, lateral **F** phallus, ventral.

###### Diagnosis.

This species is undoubtedly closely related to *Chimarrafoliata* Kimmins and *C.giboni* sp. nov. as evidenced by its foliate dorsal lobes of tergum X. It is most similar to *C.foliata* in the overall structure of its inferior appendages and in the general shape of segment IX, which has its posteroventral margin expanded at the level of the inferior appendages, and by the general lobate and semi-sclerotized structure of the endotheca. It differs diagnostically in the shape of its inferior appendages, which have a broader basodorsal lobe and also an acute ventral apex, as viewed laterally. The semi-sclerotized endotheca also seems to be somewhat different in shape, more elongate in *C.parafoliata* than in *C.foliata*, but the constancy and significance of this difference is difficult to assess.

###### Description.

***Adult.*** Overall color (in alcohol) yellowish brown, head slightly darker, setal warts of head pale, contrasting. Head short (postocular parietal sclerite short). Palps elongate; maxillary palp with 1^st^ segment very short (approximately as long as wide), 2^nd^ segment moderately elongate (~ 3× 1^st^), apex with cluster of ~ 8–10 stiff setae, 3^rd^ segment moderately elongate, slightly longer than 2^nd^, 4^th^ segment short (~ 1/2 length of 2^nd^), 5^th^ segment very elongate and narrow (subequal to 3^rd^ and 4^th^ combined). Forewing length: male, 4.0 mm. Forewing forks I, II, III, and V present; hind wing with forks II and V only. Forewing with R_1_ nearly straight, stem of Rs weakly inflected, basal fork of discoidal cell distinctly enlarged, evenly forked, length of cell slightly > 2× width, forks I and II sessile, *r* crossvein not evident, *s*, *r-m*, and *m* crossveins linear and hyaline, both 2A and 3A looped to 1A (2A without apical fork). Hind wing with R_1_ obsolete (or fused to subcosta), fork II sessile, anal loop small. Forelegs with apical tibial spur short; male with modified tarsal claws, apical three segments of tarsi short and flattened, claws asymmetrical, outer one elongate and twisted.

***Male genitalia.*** Segment VIII short, dorsal margin of tergum slightly expanded, sternum without posteroventral projection. Segment IX, in lateral view, with anterior margin nearly straight, ventral margin very distinctly expanded at level of inferior appendages, segment narrowing and convergent dorsally, dorsal margin without apodemes, but with paired, elongate, narrow, scabrous, posteriorly-directed processes from posterolateral margin, apices of processes acute, ventral process very short, ventrally projecting; as viewed dorsally, with tergum discontinuous mesally, posterior processes widely separated basally, mesally curved apically, sternum short, subtruncate. Tergum X with short membranous mesal lobe, lateral lobes with expanded foliate basal lobes and narrow, projecting apices; sensilla probably only two on each lobe, one apical and one preapical on narrow apical projection. Preanal appendages moderately large and knob-like, distinctly flattened, constricted basally, inserted membranously (not fused to segments IX or X). Inferior appendage, as viewed laterally, relatively short, convexly rounded basally, dorsally with rounded projection with marginal setae, apically with short, angular projections on ventral margin and midlaterally, the latter with sclerotized cusp projecting from mesal margin. Phallic apparatus with phallobase short, ventral apex short and deflexed, rounded apically, well anchored within segment by semi-sclerotized periphallic membrane (attached to lateral margin of segment IX); endotheca elongate, lightly sclerotized, with evident structure including dorsal, lateral, and apical lobes; phallotremal sclerite complex indistinct, apparently composed of short rod and ring structure.

###### Etymology.

*Chimarraparafoliata*, the species name meaning alongside or next to, because of the great similarity of this species to *C.foliata* Kimmins.

##### ﻿ Chimarra pectinella sp. nov.

Taxon classificationAnimaliaTrichopteraPhilopotamidae

2C184F30-BCD5-5005-89A2-187549C7223F

http://zoobank.org/78E8A2D4-C9C6-4C10-8B2E-3F9829E988C8

Fig. 54A–F

###### Type material.

***Holotype*.** Ghana – **Central Reg.** ● ♂ (in alcohol); Kakum Forest Reserve; 5°21'N, 1°22'W; 8–15 Nov. 1994; T Andersen leg.; Malaise trap; UMSP 000550061.

**Figure 54.**
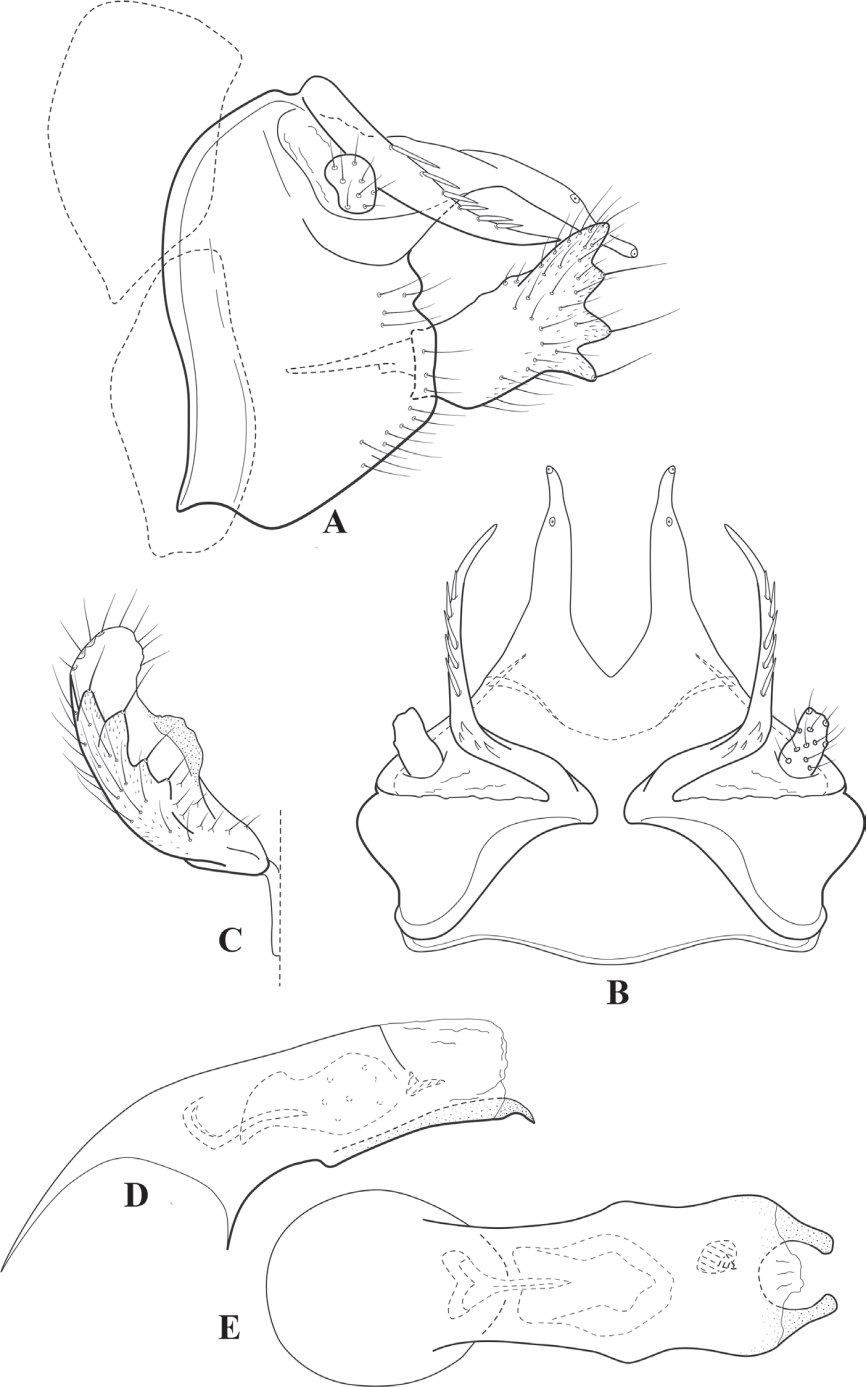
*Chimarrapectinella* sp. nov., ♂ genitalia **A** lateral **B** dorsal, segments IX and X **C** inferior appendage, ventral **D** phallus, lateral **E** phallus, ventral.

###### Additional material.

Ghana – **Central Reg.** ● 2♀♀; Kakum Forest Reserve; 5°21'N, 1°22'W; 8–15 Nov. 1994; T Andersen leg.; Malaise trap; ZMBN ● 1♀; same collection data as for preceding; UMSP.

###### Diagnosis.

*Chimarrapectinella* is probably closest to *C.lobulata* sp. nov., as evidenced by the similarity in the shapes of segment IX and tergum X of both species. Both species also have the dorsal processes of segment IX very narrowly separated mesally. *Chimarrapectinella* differs in the shape of its inferior appendages, with the setae on the apical margin on almost lobe-like projections, and by having a comb-like row of spines on the dorsal processes of segment IX, which, unlike *C.lobulata* lack a scabrous surface texture. It also differs in the shorter, smaller, and less ventrally curved apicoventral lobes of the phallobase, and by having a distinct cusp or tooth on the mesal surface of the inferior appendages.

###### Description.

***Adult.*** Overall color (in alcohol) yellowish brown, head slightly darker, setal warts of head pale, contrasting. Head short (postocular parietal sclerite short). Palps elongate; maxillary palp with 1^st^ segment very short (approximately as long as wide), 2^nd^ segment moderately elongate (~ 3× 1^st^, slightly shorter than 3^rd^), apex with cluster of ~ 8 stiff setae, 3^rd^ segment moderately elongate, 4^th^ segment short (~ ½ length of 2^nd^), 5^th^ segment very elongate and narrow (slightly longer than 3^rd^ and 4^th^ combined). Forewing length: male, 4.0 mm; female, 4.5–4.8 mm. Forewing forks I, II, III, and V present; hind wing with forks II and V only. Forewing with R_1_ nearly straight, stem of Rs weakly inflected, basal fork of discoidal cell distinctly enlarged, evenly forked, length of cell slightly > 2× width, fork I subsessile, fork II sessile, fork III with veins crossed (both forewings of male, possibly aberration, female with normal fork), *r* crossvein diagonal, intersecting discoidal cell near apical fork, *s*, *r-m*, and *m* crossveins linear and hyaline, both 2A and 3A looped to 1A (2A without apical fork). Hind wing with R_1_ obsolete (or fused to subcosta), fork II sessile, anal loop small. Forelegs with apical tibial spur short; male with modified tarsal claws, apical three segments of tarsi short and flattened, claws asymmetrical, outer one elongate and twisted.

***Male genitalia.*** Segment VIII moderate in length, tergum slightly longer than sternum, sternum without posteroventral projection. Segment IX, in lateral view, relatively long, narrowed dorsally at approximately level of preanal appendages, ventral margin only slightly expanded, dorsal margin without apodemes, but with paired, elongate, narrow, posteriorly directed processes from posterior margin, each with row of short spines on dorsal margin, apices of processes acute, ventral process absent; as viewed dorsally, with tergum discontinuous mesally, posterior processes proximate mesally, bowed outward, sternum short, subtruncate. Tergum X without evident mesal lobe, lateral lobes divided mesally, moderately elongate and narrow, with narrow, projecting apex; sensilla probably only two on each lobe, one apical and the other preapical. Preanal appendages very small and rounded, inserted membranously (not fused to segments IX or X). Inferior appendage with only weak basal inflection, widened apically, apical margin with short nipple-like projections, each with elongate seta; as viewed caudally, with slight mesal curvature, apex rounded, mesal surface with distinctly sclerotized cusp. Phallic apparatus with phallobase moderately elongate, lightly sclerotized, with usual basodorsal expansion, apparently well anchored within segment by semi-sclerotized periphallic membrane (attached to lateral margin of segment IX), apicoventral margin of phallobase sclerotized and slightly projecting, mesal margin with U-shaped invagination, producing short paired, sclerotized processes; endotheca with very short spine; phallotremal sclerite complex large, composed of relatively elongate rod and ring structure, with lightly sclerotized apical structure.

###### Etymology.

*Chimarrapectinella*, used as an adjective and derived from the Latin *pecten*, a comb, in reference to the row of comb-like spines on the dorsolateral lobes of segment IX in this species.

#### Species not assigned to subgroup

##### ﻿ Chimarra agumatsa sp. nov.

Taxon classificationAnimaliaTrichopteraPhilopotamidae

BA890B78-11C9-50D0-BBA8-F77E41EEEBDC

http://zoobank.org/71853F1C-53D9-43C0-AC0D-652E87E34878

Fig. 55A–F

###### Type material.

***Holotype*.** Ghana – **Volta Reg.** ● ♂ (in alcohol); Wli, Agumatsa waterfall, station # 2^B^; 7°07'29"N, 0°35'31"E; 5–8 Mar. 1993; JS Amakye & J Kjærandsen leg.; Malaise trap; UMSP 000550055. ***Paratypes*.** Ghana – **Volta Reg.** ● 2♂♂; same data as for holotype except 8–11 Mar. 1993; ZMBN ● 6♂♂; same data as for holotype except station # 1^B^; 5–14 Mar. 1993; ZMBN ● 2♂♂; same data as for holotype except station # 5^C^; 6–9 Mar. 1993; ZMBN ● 1♂; same data as for holotype except station # 6; 11–20 Nov. 1993; J Kjærandsen leg.; ZMBN.

**Figure 55.**
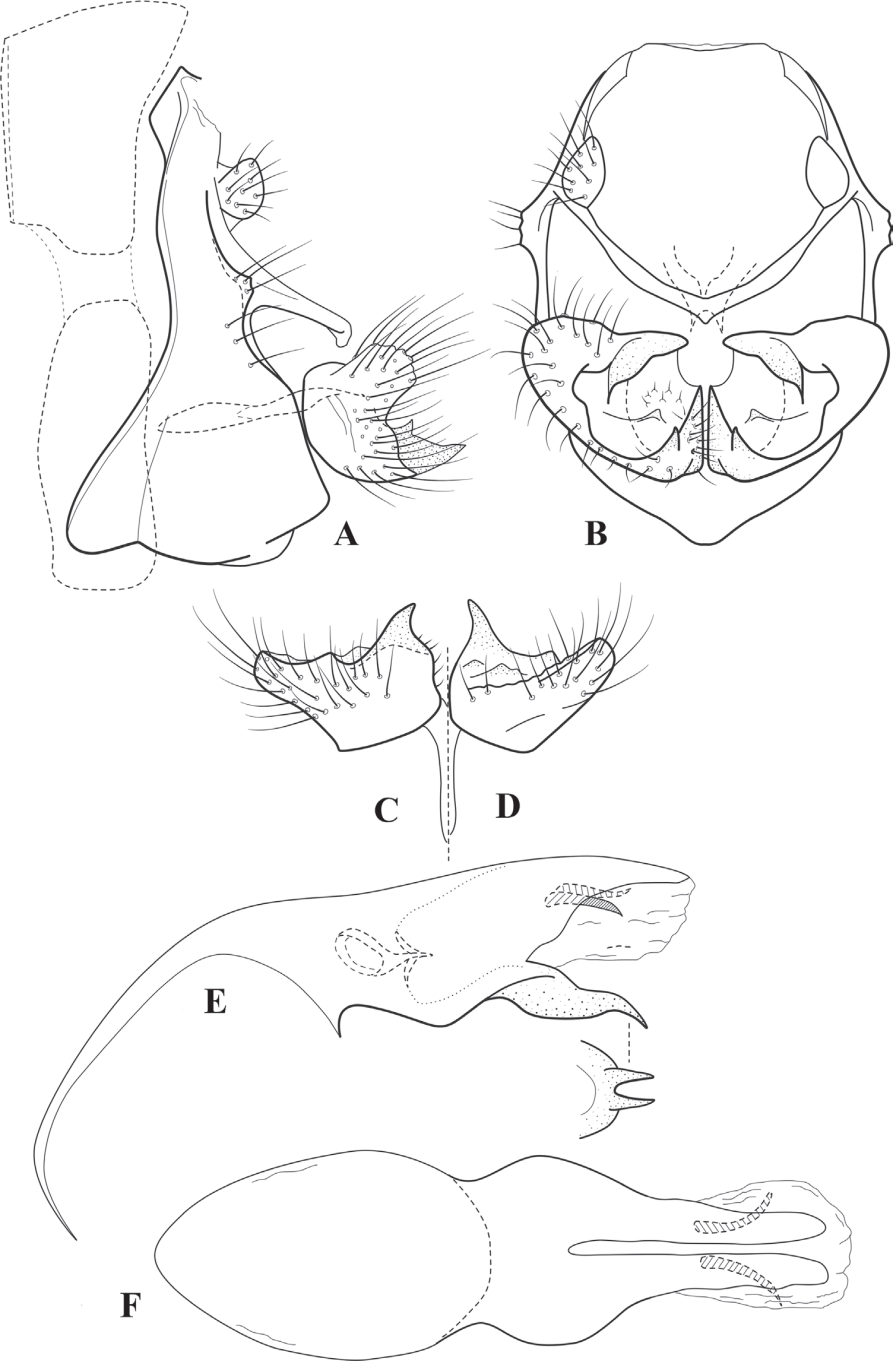
*Chimarraagumatsa* sp. nov., ♂ genitalia **A** lateral **B** caudal **C** inferior appendage, ventral **D** inferior appendage, dorsal **E** phallus, lateral, with ventral view of phallobase apex **F** phallus, dorsal.

###### Additional material.

Ghana – **Volta Reg.** ● 1♀ Wli, Agumatsa waterfall, station # 6; 7°07'29"N, 0°35'31"E; 11–20 Nov. 1993; J Kjærandsen leg.; Malaise trap; ZMBN ● 1♀; same collection data as for preceding; UMSP.

###### Diagnosis.

*Chimarraagumatsa* shows its relationship to species of the *georgensis* Group in having an elongate apical segment of its maxillary palps and in its primitive venation (straight Rs vein of the forewing, linear, unpigmented chord, and absence of a “fork” or crossvein in the anal veins). It would appear to have its greatest affinity to members of the *georgensis* subgroup, especially in having the phallobase relatively short, with its apex somewhat bifid. However, it is distinctive in a number of ways, including the absence of a tergum X, loss of fork III in the hind wing, and in having the phallus less sclerously anchored than is typical in members of the *georgensis* Group in general. For this reason, we have left the species unassigned to subgroup.

*Chimarraagumatsa* is easily diagnosed by the characters discussed above, in addition to the distinctive shape of its inferior appendages, which are very short, with both the ventral and dorsal margins incurved. It is apparently most similar to *C.ino* Marlier, whose inferior appendages have more or less the same structure but have the projections from the ventral margin more elongate and projecting. Both species lack fork III in the hind wing. The quality of the original illustration of *C.ino* make other characters difficult to compare.

###### Description.

***Adult.*** Overall color (in alcohol) yellowish brown, vertex of head slightly darker, appendages yellowish. Head moderately elongate (postocular parietal nearly as long as diameter of eye). Palps elongate; maxillary palp with 1^st^ segment very short (approximately as long as wide), 2^nd^ segment relatively short (~ 3× length of 1^st^), apex with cluster of ~ 8–10 stiff setae, 3^rd^ segment elongate, almost 2× length of 2^nd^, 4^th^ segment short (slightly shorter than 2^nd^), 5^th^ segment very elongate (nearly length of 3^rd^ and 4^th^ combined). Forewing length: male, 4.0–5.0 mm; female, 4.0 mm. Forewing forks I, II, III, and V present; hind wing with forks I, II and V. Forewing with R_1_ nearly straight, basal fork of discoidal cell slightly enlarged, evenly forked, length of cell ~ 2× width, fork I subsessile, fork II sessile, *r* crossvein intersecting discoidal cell at past midlength, *s*, *r-m*, and *m* crossveins linear and hyaline, both 2A and 3A looped to 1A (2A without apical fork). Hind wing with R_1_ obsolete (or fused to subcosta), forks I and II sessile, anal loop small. Forelegs with apical tibial spur short; male with modified tarsal claws, apical three segments of tarsi short and flattened, claws asymmetrical, outer one elongate and twisted.

***Male genitalia.*** Segment VIII short, sternum without posteroventral projection. Segment IX, in lateral view, subtriangular, anteroventral and postroventral margins both moderately produced, strongly converging dorsally, dorsal margin obsolete, or nearly so, anterior margin without apodemes. Ventral process of segment greatly reduced, rounded, ventrally projecting, width at base greater than length. Tergum X apparently absent, but with narrow, converging lateral processes below preanal appendages, subtending phallus and fused apicomesally. Preanal appendages small and knob-like, rounded, slightly constricted basally, apparently fused to segment IX. Inferior appendage very short, with dorsal margin enrolled and rounded, as viewed laterally, forming a projecting cusp on the mesal surface; ventral margin of appendage projecting, acute, and mesally curved, forming short spine-like projection on mesal margin. Phallic apparatus with phallobase relatively short, not (or not evidently) anchored by sclerotized periphallic membrane; ventral apex of phallobase, as viewed laterally, projecting and acute, as viewed ventrally, weakly bifid apically; dorsal margin of phallobase, as viewed laterally, distinctly projecting, subequal in length to ventral projection, but with mesal margin forming an elongate, narrow, desclerotized strip over much of its length, as viewed dorsally; endotheca apparently short and simple in structure, with a pair of short, curved, symmetrically oriented phallic spines; phallotremal sclerite complex small and indistinct, composed of short rod and ring structure.

###### Etymology.

*Chimarraagumatsa*, the name considered a noun in apposition, for the name of the scenic waterfalls near which the holotype specimen was collected.

##### ﻿ Chimarra kjaerandseni sp. nov.

Taxon classificationAnimaliaTrichopteraPhilopotamidae

612BA1AC-B859-56F6-89EE-46FE05B1C4EB

http://zoobank.org/611EDC60-4653-4ECD-9B39-E6B05AE840D7

Fig. 56A–G

###### Type material.

***Holotype*** Ghana – **Volta Reg.** ● ♂ (in alcohol); Wli, Agumatsa waterfall, station # 5^C^; 7°07'29"N, 0°35'31"E; 9–12 Mar. 1993; JS Amakye & J Kjærandsen leg.; Malaise trap; UMSP 000550057.

**Figure 56.**
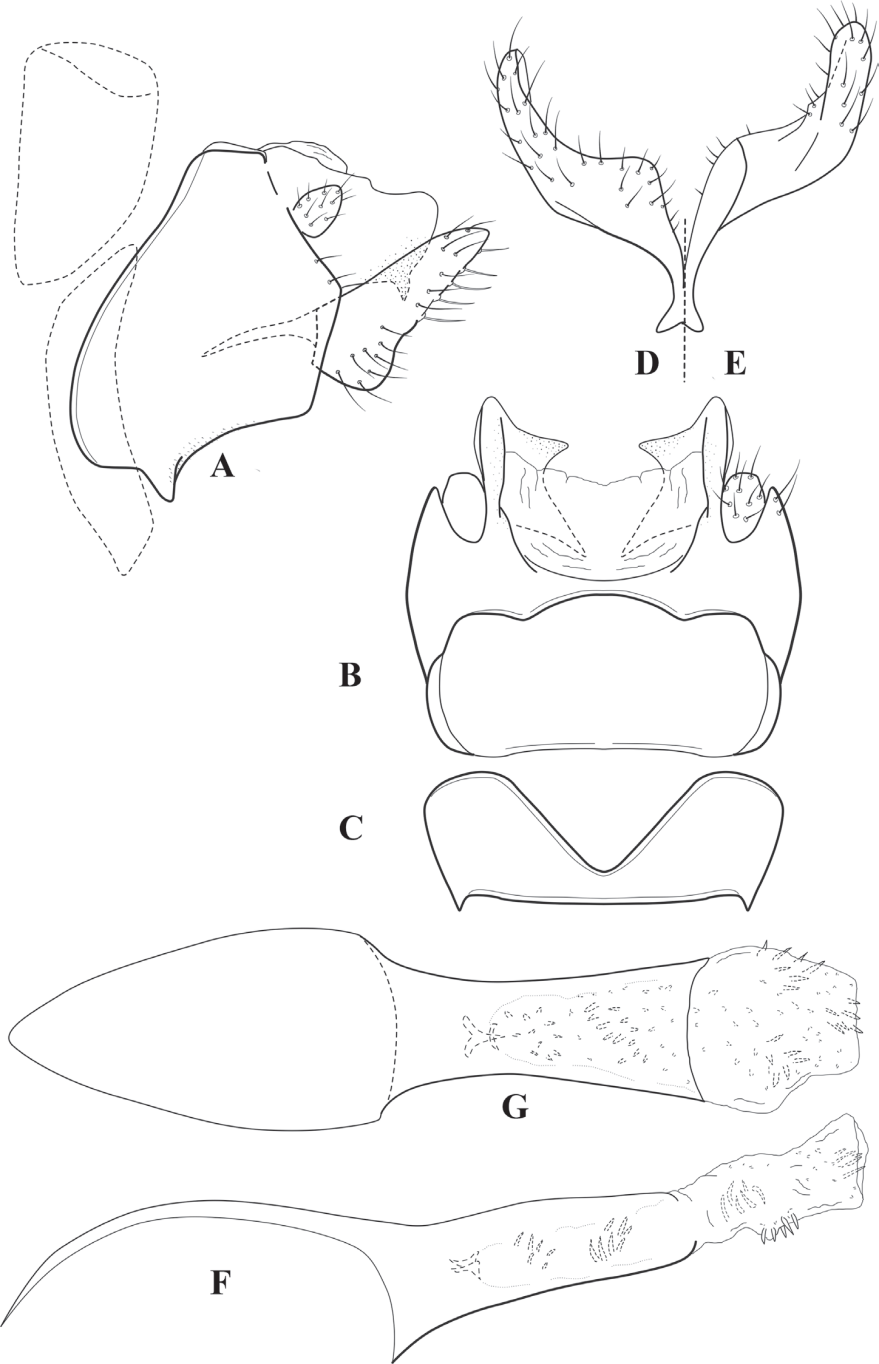
*Chimarrakjaerandseni* sp. nov., ♂ genitalia **A** lateral **B** dorsal, segments IX and X **C** tergum VIII, dorsal **D** inferior appendage, ventral **E** inferior appendage, dorsal **F** phallus, lateral **G** phallus, dorsal.

###### Additional material.

Ghana – **Volta Reg.** ● 1♀; Wli, Agumatsa waterfall, station # 6; 7°07'29"N, 0°35'31"E; 20 Nov. 1993; J Kjærandsen leg.; light trap; UMSP.

###### Diagnosis.

*Chimarrakjaerandseni* is a unique and enigmatic species, very different from the other species placed in the *georgensis* Group, but sharing some of the diagnostic characters, including a maxillary palp with a relatively elongate terminal segment and primitive venational characters, including a forewing with a straight Rs vein, a linear, unpigmented chord, and absence of a “fork” or crossvein in the anal veins. Like the species in the *evoluta* subgroup, it lacks fork I and III in the hind wing. However, none of the genitalic characters are particularly suggestive of a relationship with this subgroup, since it lacks either the elongate processes from the dorsal margin of segment IX or modified ventral apex of the phallobase that characterize other species in the subgroup. Because of this, and despite the very suggestive hind wing venational loss characters, we prefer to consider this species unassigned to subgroup in the *georgensis* Group.

General diagnostic characters of the species include the general shape of segment IX, which is relatively elongate and lacks anterodorsal apodemes, the ventrally projecting ventral process of the same segment, the simple lateral lobes of tergum X, and the short curved inferior appendages. Additionally, the numerous small spines in the phallus, its relative length, absence of a projecting ventral apex on the phallobase, as well as the relatively desclerotized posteromesal margin of segment VIII are also all useful diagnostic characters, not found in any other species of the *georgensis* Group.

###### Description.

***Adult*.** Overall color (in alcohol) yellowish brown, vertex of head slightly darker, appendages yellowish. Head relatively short (postocular parietal sclerite ~ 1/2 length of eye). Palps relatively elongate; maxillary palp with 1^st^ segment short and stout (approximately as long as wide), 2^nd^ segment moderately elongate (~ 3× 1^st^), apex with cluster of 6–8 stiff setae, 3^rd^ segment slightly longer than 2^nd^, 4^th^ segment short (~ 1/2 length of 3^rd^), 5^th^ segment relatively elongate and very narrow (somewhat shorter than 3^rd^ and 4^th^ combined). Forewing length: male, 4.0 mm. Forewing with forks I, II, III, and V present; hind wing with forks II and V only. Forewing with Rs straight, or nearly so, basal fork of discoidal cell slightly enlarged, evenly forked, length of cell ~ 2× width, fork I subsessile, II sessile, *r* crossvein not evident, *s*, *r-m*, and *m* crossveins linear and hyaline, both 2A and 3A looped to 1A (2A without apical fork). Hind wing with R_1_ obsolete (or fused to subcosta), fork II slightly subsessile, anal loop small. Forelegs with apical tibial spur very short; male with modified tarsal claws, apical tarsal segment enlarged and flattened, claws asymmetrical, outer one elongate and slightly twisted.

***Male genitalia.*** Segment VIII short, sternum without posteroventral projection, tergum slightly longer, with very distinct membranous posteromesal invagination. Segment IX, in lateral view, relatively elongate ventrally, anteroventral margin moderately expanded, posterior margin subparallel to anterior margin to point just above inferior appendage, then angularly narrowing dorsally; as viewed dorsally, with dorsal margin short, but continuous, anteroventral margin subtruncate. Ventral process of segment IX ventrally projecting, subtriangular, closer to anterior than posterior margin. Lateral lobes of tergum short and broad, subparallel, widely separated dorsally, with membranous lobe between, lobes converging anteroventrally, apices of lobes each with angular, beak-like, ventral projection, sensilla absent or indistinct. Preanal appendages prominent, rounded and knob-like, slightly constricted basally, apparently fused to segment IX. Inferior appendage, as viewed laterally, short and simple in shape, distinctly inflected basally, longer than wide, slightly tapering, apex rounded, mesally curved as viewed dorsally or ventrally, without cusp or tooth on mesal margin. Phallic apparatus with phallobase moderately elongate, tubular, without distinctly sclerotized periphallic membrane, ventral apex of phallobase not projecting. Endotheca at least moderately elongate, textured with small spine-like projections and several clusters of short spines, phallotremal sclerite complex small and indistinct, forming short rod and ring structure.

###### Etymology.

We are pleased to name this species for Jostein Kjærandsen, who participated in the collecting expedition that generated much of the material that the current study is based on, in addition to doing an initial sorting of the material and initiating the study.

## Supplementary Material

XML Treatment for Chimarra bispinosa

XML Treatment for Chimarra calundoensis

XML Treatment for Chimarra dybowskina

XML Treatment for Chimarra elga

XML Treatment for Chimarra fallax

XML Treatment for Chimarra jacquemarti

XML Treatment for Chimarra lanceolata

XML Treatment for Chimarra robynsi

XML Treatment for Chimarra togoana

XML Treatment for Chimarra akana

XML Treatment for Chimarra eshowensis

XML Treatment for Chimarra krugeri

XML Treatment for Chimarra morogoroensis

XML Treatment for Chimarra pedaliotus

XML Treatment for Chimarra szunyoghyi

XML Treatment for Chimarra tanzaniensis

XML Treatment for Chimarra triangularis occidentalis

XML Treatment for Chimarra waensis

XML Treatment for Chimarra amakyei

XML Treatment for Chimarra mazumbai

XML Treatment for Chimarra usambara

XML Treatment for Chimarra wliensis

XML Treatment for Chimarra callasae

XML Treatment for Chimarra intexta

XML Treatment for Chimarra minima

XML Treatment for Chimarra sassandrae

XML Treatment for Chimarra dulensis

XML Treatment for Chimarra kibiensis

XML Treatment for Chimarra minacis

XML Treatment for Chimarra tangaensis

XML Treatment for Chimarra multisensillata

XML Treatment for Chimarra ankylis

XML Treatment for Chimarra aurita

XML Treatment for Chimarra crescentis

XML Treatment for Chimarra indicis

XML Treatment for Chimarra latidentis

XML Treatment for Chimarra leptodactylus

XML Treatment for Chimarra obuncata

XML Treatment for Chimarra polycentropoides

XML Treatment for Chimarra ralphi

XML Treatment for Chimarra serrella

XML Treatment for Chimarra triramosa

XML Treatment for Chimarra uncinata

XML Treatment for Chimarra vermitergata

XML Treatment for Chimarra giboni

XML Treatment for Chimarra lobulata

XML Treatment for Chimarra mgwashi

XML Treatment for Chimarra parafoliata

XML Treatment for Chimarra pectinella

XML Treatment for Chimarra agumatsa

XML Treatment for Chimarra kjaerandseni
